# Do reporting guidelines have an impact? Empirical assessment of changes in reporting before and after the PRISMA extension statement for network meta-analysis

**DOI:** 10.1186/s13643-021-01780-9

**Published:** 2021-09-10

**Authors:** Areti Angeliki Veroniki, Sofia Tsokani, Stella Zevgiti, Irene Pagkalidou, Katerina-Maria Kontouli, Pinar Ambarcioglu, Nikos Pandis, Carole Lunny, Adriani Nikolakopoulou, Theodoros Papakonstantinou, Anna Chaimani, Sharon E. Straus, Brian Hutton, Andrea C. Tricco, Dimitris Mavridis, Georgia Salanti

**Affiliations:** 1 Department of Primary Education, School of Education, University of Ioannina, Ioannina, Greece; 2 Knowledge Translation Program, Li Ka Shing Knowledge Institute, St. Michael’s Hospital, Toronto, ON Canada; 3 Department of Hygiene, Social-Preventive Medicine and Medical Statistics, Medical School, Aristotle University of Thessaloniki, Thessaloniki, Greece; 4 Department of Biostatistics, Faculty of Veterinary Medicine, Mustafa Kemal University, Tayfur Sökmen Kampüsü 31060, Antakya, Hatay Turkey; 5 Department of Orthodontics and Dentofacial Orthopedics, Dental School/Medical Faculty, University of Bern, Bern, Switzerland; 6 Cochrane Hypertension Review Group and the Therapeutics Initiative, University of British Columbia, Vancouver, Canada; 7 Institute of Medical Biometry and Statistics, Faculty of Medicine and Medical Center, University of Freiburg, Freiburg, Germany; 8 Institute of Social and Preventive Medicine, University of Bern, Bern, Switzerland; 9 Université de Paris, Research Center of Epidemiology and Statistics Sorbonne Paris Cité (CRESS UMR1153), INSERM, INRA, Paris, France; 10 Cochrane France, Paris, France; 11 Department of Geriatric Medicine, University of Toronto, Toronto, ON Canada; 12 Ottawa Hospital Research Institute, Ottawa, ON Canada; 13 University of Ottawa School of Epidemiology and Public Health, Ottawa, ON Canada; 14 Epidemiology Division, Dalla Lana School of Public Health, University of Toronto, Toronto, ON Canada; 15 Paris Descartes University, Sorbonne Paris CitéFaculté de Médecine, Paris, France

**Keywords:** Multiple treatment meta-analysis, PRISMA-NMA, Systematic review, Reporting

## Abstract

**Background:**

The Preferred Reporting Items for Systematic Reviews and Meta-Analyses (PRISMA) extension statement for network meta-analysis (NMA) published in 2015 promotes comprehensive reporting in published systematic reviews with NMA. PRISMA-NMA includes 32 items: 27 core items as indicated in the 2009 PRISMA Statement and five items specific to the reporting of NMAs. Although NMA reporting is improving, it is unclear whether PRISMA-NMA has accelerated this improvement. We aimed to investigate the impact of PRISMA-NMA and highlight key items that require attention and improvement.

**Methods:**

We updated our previous collection of NMAs with articles published between April 2015 and July 2018. We assessed the completeness of reporting for each NMA, including main manuscript and online supplements, using the PRISMA-NMA checklist. The PRISMA-NMA checklist originally includes 32 total items (i.e. a 32-point scale original PRISMA-NMA score). We also prepared a modified version of the PRISMA-NMA checklist with 49 items to evaluate separately at a more granular level all multiple-content items (i.e. a 49-point scale modified PRISMA-NMA score). We compared average reporting scores of articles published until and after 2015.

**Results:**

In the 1144 included NMAs the mean modified PRISMA-NMA score was 32.1 (95% CI 31.8–32.4) of a possible 49-excellence-score. For 1-year increase, the mean modified score increased by 0.96 (95% CI 0.32 to 1.59) for 389 NMAs published until 2015 and by 0.53 (95% CI 0.02 to 1.04) for 755 NMAs published after 2015. The mean modified PRISMA-NMA score for NMAs published after 2015 was higher by 0.81 (95% CI 0.23 to 1.39) compared to before 2015 when adjusting for journal impact factor, type of review, funding, and treatment category. Description of summary effect sizes to be used, presentation of individual study data, sources of funding for the systematic review, and role of funders dropped in frequency after 2015 by 6–16%.

**Conclusions:**

NMAs published after 2015 more frequently reported the five items associated with NMA compared to those published until 2015. However, improvement in reporting after 2015 is compatible with that observed on a yearly basis until 2015, and hence, it could not be attributed solely to the publication of the PRISMA-NMA.

**Supplementary Information:**

The online version contains supplementary material available at 10.1186/s13643-021-01780-9.

## Background

The Preferred Reporting Items for Systematic Reviews and Meta-Analyses (PRISMA) statement was developed to promote comprehensive reporting in published systematic reviews with narrative summary and pairwise meta-analysis to increase transparency and reproducibility [1]. The PRISMA statement, published in 2009, was initially designed for systematic reviews and pairwise meta-analyses of healthcare interventions and has been widely used by reviewers and journals [2]. The statement was updated to PRISMA 2020 to reflect recent advances in the methods of systematic reviews [3]. It is of critical importance to report sufficient and accessible information so that research can be reproduced, which can help avoiding biased recommendations and distort health-care decision making [4, 5].

The PRISMA extension for network meta-analysis (NMA), published in 2015, was prompted by empirical research showing that reporting of NMA was problematic [6]. Our previous scoping reviews including 456 NMAs published until 2015 showed that only a quarter of NMAs were of high methodological quality and that half of the NMAs had failed to report the prerequisite assumptions of evidence synthesis, although both reporting and methodology were found to be improving over time [7, 8]. The PRISMA extension provides guidance for reporting of systematic reviews with NMA and highlights their key reporting components, aiming to improve primarily reporting and indirectly the conduct of reviews with NMA. There are five additional items in the PRISMA extension to NMA: description of methods used to explore network geometry, description of methods used to assess inconsistency, presentation of network diagram, brief overview of network characteristics, and description of results from investigations of inconsistency.

There are few empirical studies that have evaluated the completeness of reporting of NMAs since the publication of the PRISMA extension. The assessment of reporting of 21 systematic reviews with NMAs published until 2017 using the PRISMA-NMA checklist showed that reporting was low in the dental care field [9]. Tonin et al. [10] assessed the extend of compliance with PRISMA (for NMAs published before 2015) and PRISMA-NMA (for NMAs published between 2015 and end of 2016) in 477 NMAs of pharmacological treatments and showed minor improvement in reporting according to the PRISMA score. A scoping review of 89 NMAs with complementary and alternative medicines published up until 2018 showed that the PRISMA-NMA guideline was overall adequately adopted through key reporting items such as the existence of a protocol, exploring network geometry, and risk of bias assessment were often missing (up to 65%) [11].

As previous empirical research has suggested that reporting of NMAs is improving over time, it is unclear whether the PRISMA-NMA statement has accelerated this improvement. Our objective was to empirically assess whether the PRISMA-NMA statement had an important impact on the completeness of reporting by comparing NMA articles of randomised controlled trials (RCTs) between two time periods (2013–2015 and 2016–2018). We also aim to investigate publication features (such as journal characteristics or the existence of a protocol) and network characteristics (such as type of interventions compared) that might modify the completeness of reporting. We additionally sought to highlight key items that require further attention and potential improvement moving forward.

## Methods

### Eligibility criteria and study selection

We updated our previous collection of NMAs with articles published between April 2015 and July 2018 using the same search strategy and inclusion criteria as described in our previously published reviews [7, 8, 12]. In brief, networks were eligible if they included RCTs only, included at least four treatment nodes in the network, they had conducted any form of valid indirect comparison or NMA, and the number of studies was larger than the number of treatments compared (see also Appendix 1). In the present study, we included articles published between 2013 and 2018, to have an equal chronological timeframe before and after the PRISMA-NMA publication (published in June 2015).

### Data abstraction

We developed a predefined data abstraction form in REDCap [13]. We included first author’s name, publication year, journal name, and country of corresponding author in the abstracted data. We denoted each journal’s impact factor as indicated in the Web of Science (year 2019). Impact factors for journals not included in this list were obtained from the relevant journals’ official website. If an impact factor was not available for year 2019, it was retrieved from a previous year. We grouped NMAs according to the type of treatment comparisons that were presented (pharmacological vs placebo, pharmacological vs pharmacological or non-pharmacological vs any intervention) [12]. If a network included pharmacological interventions and a placebo or control, then it was classified as pharmacological vs placebo/control comparison type. Networks with pharmacological treatments but no placebo or control were categorised as a pharmacological vs pharmacological comparison type. Networks including at least one non-pharmacological treatment were classified as non-pharmacological vs any intervention comparison type. We classified NMAs according to the structure of the network (i.e. open networks vs networks with at least one closed loop of evidence) and the type of analyses presented (Bayesian, frequentist, or both). We also categorised NMAs according to their type of funding, irrespective of authors’ funding, as industry-sponsored, publicly sponsored, mixed-funded, non-sponsored studies, and funding not reported.

We assessed the completeness of reporting for each NMA, including main manuscript and online supplements, using the PRISMA-NMA checklist, which includes 32 items in total: 27 core items as indicated in the core 2009 PRISMA Statement and five additional items (S1-S5) specific to the reporting of NMAs. We also prepared a modified version of the PRISMA-NMA checklist such that multiple items could be listed an evaluated separately at a more granular level, which resulted in 49 items (Appendix Table 1); this included for example two separate terms for systematic review and NMA/related form of meta-analysis in the title, instead of a single item. We assigned each component a ‘yes’ (1 point) or ‘no’ (0 points) depending on whether it was reported. We ended up with a scale of 32 points for the original PRISMA-NMA items (termed ‘original PRISMA-NMA score’) and a scale of 49 points for the modified checklist (‘modified PRISMA-NMA score’).

### Statistical analysis

We compared reporting scores (both original and modified PRISMA-NMA scores, as described in the data abstraction section) between NMAs published in the interval January 2013 to December 2015 and January 2016 to December 2018. We performed a descriptive analysis for the PRISMA-NMA items (reporting percentage per item) and presented the percentage of studies with adequate reporting for each item prior to and after the PRISMA-NMA publication. We evaluated whether there was a total improvement in reporting over publication year using the Cox and Stuart trend test (null hypothesis: there is not a monotonic trend) in the *trend* library in R [14].

We compared reporting scores between Cochrane NMAs, non-Cochrane NMAs with a protocol, and non-Cochrane NMAs without a protocol. In non-Cochrane NMAs, we considered a protocol to be available only when this was reported in the manuscript (including registration in PROSPERO). Reporting scores were additionally compared between journals endorsing the original PRISMA and journals that did not recommend using PRISMA in their submission guidelines, as reported in http://www.prisma-statement.org/Endorsement/PRISMAEndorsers. We calculated the mean percent score difference between 2013 and 2015 and 2016 and 2018 along with a 95% confidence interval (CI) per journal with impact factor > 10. We also calculated the mean and median scores for each scale, along with the 95% CI or interquartile range (IQR), respectively.

We conducted a univariable regression analysis assessing the overall impact of year of publication as a dichotomous variable until vs. after 2015 on the PRISMA-NMA score. We also performed two univariable regression analyses to assess the impact of year of publication on the PRISMA-NMA score, for studies published before and after 2015, separately. Similarly, we performed a univariable regression for studies published until and after the PRISMA-NMA publication focusing only on the NMA specific items S1-S5 (min score 0, max score 5). To evaluate jointly the influence of the journal impact factor, year of publication, treatment type (pharmacological vs. non-pharmacological), funding type (industry or mixed vs. other), and review type (review with protocol vs. review without protocol) on reporting scores, we performed a multivariable regression analysis model. In case the impact factor was not available for a journal, we considered it as a zero value. We labelled the treatment type pharmacological when the network was categorised as pharmacological vs placebo/control or as pharmacological vs pharmacological comparison type, and the non-pharmacological treatment type when the network was categorised as non-pharmacological vs any. A network had an industry or mixed funding type when at least one of the sponsors for the review itself was industry. Each review was also classified depending on the protocol availability. We decided to use a binary categorisation of each covariate to improve power in our analysis. We also performed a multivariable regression using the same covariates apart from year of publication, which was considered as a dichotomous variable until vs. after 2015, as a subsequent sensitivity analysis. For our analyses, we used both original and modified PRISMA-NMA scores, a significance level of 5%, and the R software version 3.6.2 (R Development Core Team 2019) [15].

## Results

### Literature search

**Fig. 1:**
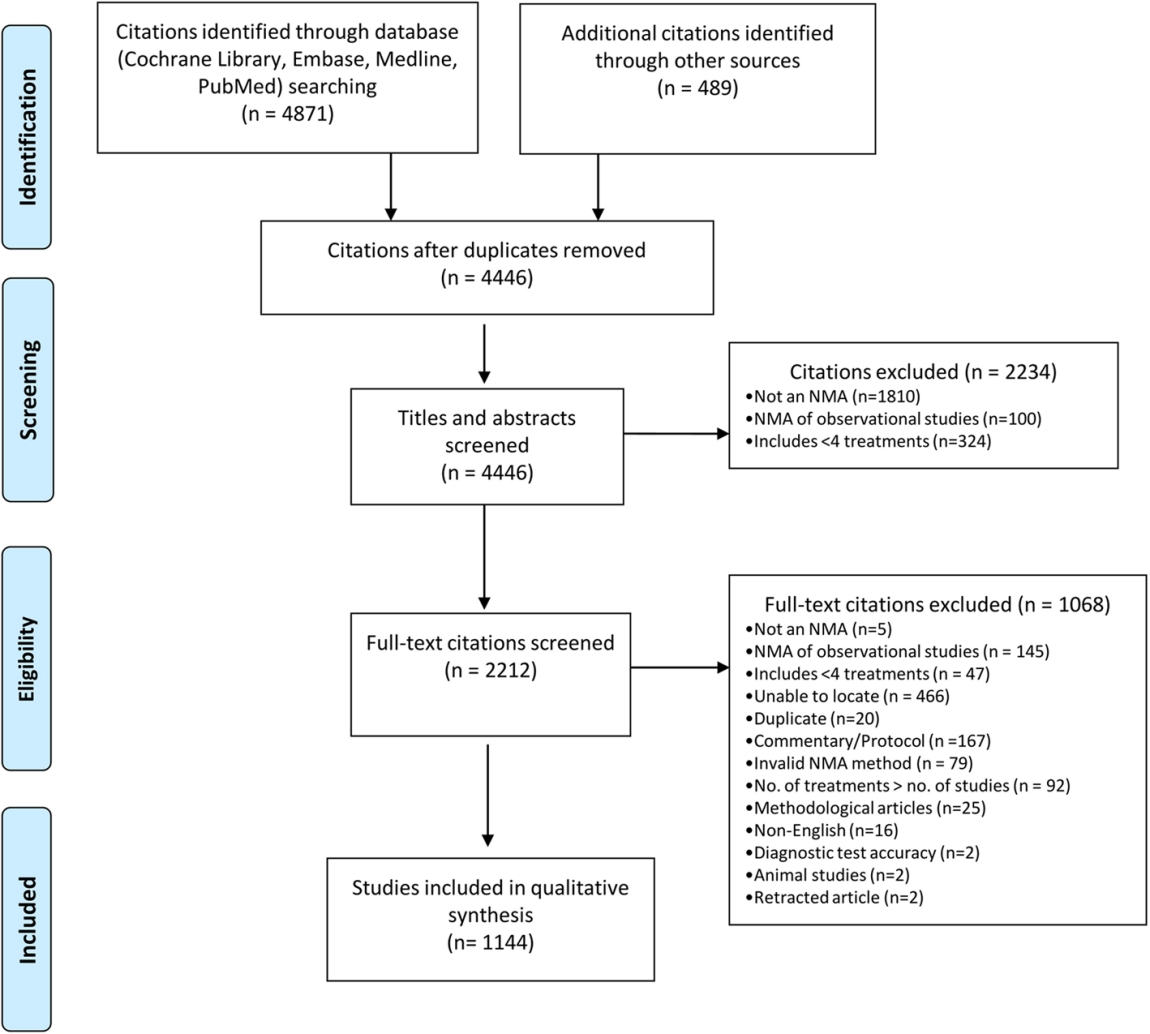
Flow diagram of the selection process for the included systematic reviews and network meta-analyses

The updated literature search yielded 4871 citations (Fig. 1). We included an additional of 489 citations published between 2013 and 2018 from supplementary sources and our published scoping reviews [7, 8, 12]. After de-duplication (i.e. multiple publications of the same systematic review and NMA), we screened 4446 unique titles and abstracts and 2212 full-text citations. Overall, we included 1144 NMAs that fulfilled the eligibility criteria (Appendix Table 2).

In the following, NMA reporting is presented according to the modified PRISMA-NMA score. Results according to the original PRISMA-NMA score are presented in the supplementary files.

### Study and method characteristics

**Fig. 2:**
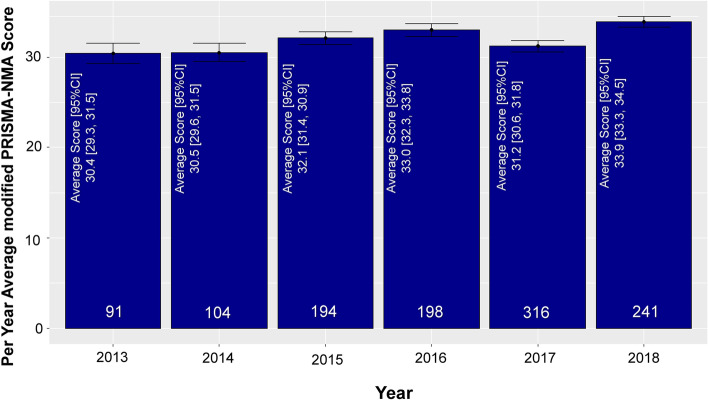
Number of systematic reviews and network meta-analyses, and per-paper average modified PRISMA-NMA score published between 2013 and 2018 ‘*’ denotes that the search was performed up to July 2018, and thus only 7 months of that year are reflected in this graph. Error bars parallel to the *y*-axis represent the uncertainty of the modified PRISMA-NMA score

The number of NMAs published by year between 2013 and 2018 along with the per-year average score across years is shown in Fig. 2 (see also Appendix Fig. 1 for the original PRISMA-NMA score). The highest mean reporting score was observed in 2018, whereas the 66% of the NMAs (755 of 1144 NMAs) were published in 2016–2018. The majority of the corresponding authors had an affiliation with China (357, 31%), the USA (184, 16%), and the UK (164, 14%) (Table 1, Appendix Fig. 2). Of the 450 journals included in our database, only 33 had already adopted the original PRISMA guidance. NMAs were published in journals with a median impact factor of 3.74 (IQR 2.69–5.81; Appendix Fig. 3). Among the included reviews, 801 (70%) NMAs employed a Bayesian hierarchical approach alone (779, 68%) or in addition to a frequentist approach (22, 2%). Most networks included pharmacological treatments only or in addition to a placebo/control treatment (907, 79%). We identified 33 Cochrane reviews with a protocol (3% of the networks), 280 non-Cochrane reviews with a protocol (24% of the networks), and 831 non-Cochrane reviews without a protocol (73% of the networks).

**Table 1:** Characteristics of NMAs published between 2013 and 2018

Published in journals endorsing NMA	NMAs published between 2013 and 2015			NMAs published between 2016 and 2018			Total		
	**Yes**	**No**	**Total**	**Yes**	**No**	**Total**	**Yes**	**No**	**Total**
	88 (23%)	301 (77%)	389	82 (11%)	673 (89%)	755	170 (15%)	974 (85%)	1144
**Ten most prevalent countries of corresponding author: frequency (%)**									
China	17 (22%)	60 (78%)	77	21 (8%)	256 (92%)	277	38 (11%)	316 (89%)	354
USA	17 (22%)	61 (78%)	78	12 (11%)	94 (89%)	106	29 (16%)	155 (84%)	184
UK	16 (22%)	58 (78%)	74	13 (14%)	77 (86%)	90	29 (18%)	135 (82%)	164
Canada	10 (29%)	24 (71%)	34	8 (20%)	32 (80%)	40	18 (24%)	56 (76%)	74
Italy	5 (16%)	26 (84%)	31	4 (13%)	28 (88%)	32	9 (14%)	54 (86%)	63
Korea (South)	1 (25%)	3 (75%)	4	3 (10%)	28 (90%)	31	4 (11%)	31 (89%)	35
Germany	3 (20%)	12 (80%)	15	1 (6%)	16 (94%)	17	4 (13%)	28 (88%)	32
France	2 (20%)	8 (80%)	10	2 (17%)	10 (83%)	12	4 (18%)	18 (82%)	22
Switzerland	4 (40%)	6 (60%)	10	3 (30%)	7 (70%)	10	7 (35%)	13 (65%)	20
Japan	2 (40%)	3 (60%)	5	0 (0%)	15 (100%)	15	2 (10%)	18 (90%)	20
**Ten most prevalent journals: frequency (%)**									
Plos One	31 (100%)	NA	31	21 (100%)	NA	21	52 (100%)	NA	52
Oncotarget	NA	4 (100%)	4	NA	39 (100%)	39	NA	43 (100%)	43
Medicine	NA	9 (100%)	9	NA	33 (100%)	33	NA	42 (100%)	42
Cochrane Database Of Systematic Reviews	NA	16 (100%)	16	NA	17 (100%)	17	NA	33 (100%)	33
Scientific Reports	NA	2 (100%)	2	NA	25 (100%)	25	NA	27 (100%)	27
Current Medical Research And Opinion	NA	11 (100%)	11	NA	10 (100%)	10	NA	21 (100%)	21
BMJ	16 (100%)	NA	16	5 (100%)	NA	5	21 (100%)	NA	21
Health Technology Assessment	NA	8 (100%)	8	NA	11 (100%)	11	NA	19 (100%)	19
Alimentary Pharmacology & Therapeutics	8 (100%)	NA	8	10 (100%)	NA	10	NA	18 (100%)	18
Clinical Therapeutics	NA	7 (100%)	7	NA	8 (100%)	8	NA	15 (100%)	15
**Type of review: frequency (%)**									
Non-Cochrane review without protocol	70 (23%)	236 (77%)	306	42 (8%)	483 (92%)	525	112 (13%)	719 (87%)	831
Non-Cochrane review with protocol	18 (27%)	49 (73%)	67	40 (19%)	173 (81%)	213	58 (21%)	222 (79%)	280
Cochrane review	0 (0%)	16 (100%)	16	0 (0%)	17 (100%)	17	0 (0%)	33 (100%)	33
**Type of treatment group: frequency (%)**									
Pharmacological vs Placebo	44 (20%)	171 (80%)	215	50 (14%)	315 (86%)	365	94 (16%)	486 (84%)	580
Pharmacological vs Pharmacological	27 (26%)	77 (74%)	104	16 (7%)	207 (93%)	223	43 (13%)	284 (87%)	327
Non-pharmacological vs Any treatment	17 (24%)	53 (76%)	70	16 (10%)	151 (90%)	167	33 (14%)	204 (86%)	237
**Shape of network: frequency (%)**									
Full shaped with at least one closed loop	71 (22%)	245 (78%)	316	69 (11%)	573 (89%)	642	140 (15%)	818 (85%)	958
Open shaped with no closed loops	17 (24%)	55 (76%)	72	13 (12%)	94 (88%)	107	30 (17%)	149 (83%)	179
Unclear	0 (0%)	1 (100%)	1	0 (0%)	6 (100%)	6	0 (0%)	7 (100%)	7
**Presentation results: frequency (%)**^a^									
Presentation of NMA results									
Ranking statistics	46 (25%)	138 (75%)	363	58 (11%)	494 (89%)	552	104 (14%)	632 (86%)	736
Forest plot	48 (24%)	149 (76%)	197	60 (12%)	449 (88%)	509	108 (15%)	598 (85%)	706
League tables	37 (23%)	124 (77%)	161	48 (10%)	419 (90%)	467	85 (14%)	543 (86%)	628
**Analysis setting: frequency (%)**									
Bayesian	67 (24%)	215 (76%)	272	56 (11%)	441 (89%)	497	123 (16%)	656 (84%)	779
Frequentist	19 (19%)	79 (81%)	98	26 (11%)	209 (89%)	235	45 (14%)	288 (86%)	333
Both	0 (0%)	1 (100%)	1	0 (0%)	21 (100%)	21	0 (0%)	22 (100%)	22
Unclear	2 (25%)	6 (75%)	8	0 (0%)	2 (100%)	2	2 (20%)	8 (80%)	10
**Bayesian analysis settings: frequency (%)**^a^									
Bayesian setting									
Reported prior distributions	37 (24%)	117 (76%)	154	22 (12%)	169 (88%)	191	59 (17%)	286 (83%)	345
Model fit assessment	40 (26%)	112 (74%)	152	21 (11%)	169 (89%)	190	61 (18%)	281 (82%)	342
Used different priors as additional analyses	5 (28%)	13 (72%)	18	4 (24%)	13 (76%)	17	9 (26%)	26 (74%)	35
**Additional analyses: frequency (%)**^a^									
Additional NMA analyses									
Subgroup and/or sensitivity analysis	50 (28%)	131 (72%)	181	46 (15%)	266 (85%)	312	96 (19%)	397 (81%)	493
Meta-regression	18 (26%)	52 (74%)	70	18 (17%)	87 (83%)	105	36 (21%)	139 (79%)	175
Alternative treatment formulations in the network	13 (42%)	18 (58%)	31	3 (10%)	26 (90%)	29	16 (27%)	44 (73%)	60
**Funding: frequency (%)**									
Publicly sponsored	27 (21%)	104 (79%)	131	29 (11%)	238 (89%)	267	56 (14%)	342 (86%)	398
Funding source not reported	8 (9%)	82 (91%)	90	14 (6%)	210 (94%)	224	22 (7%)	292 (93%)	314
Non-sponsored	34 (37%)	59 (63%)	93	26 (15%)	143 (85%)	169	60 (23%)	202 (77%)	262
Industry-sponsored	16 (24%)	52 (76%)	68	11 (13%)	72 (87%)	83	27 (18%)	124 (82%)	151
Mixed-funding	3 (43%)	4 (57%)	7	2 (17%)	10 (83%)	12	5 (26%)	14 (74%)	19

^a^The total number of NMAs does not add up to 1144 as each article might pertain to more than one category

*NMA* network meta-analysis

Five in six networks included at least one closed loop of evidence (958, 84%). A total of 398 NMAs (35%) were publicly funded, but funding was not reported in 314 (27%) NMAs. The most popular journals in our NMA database were PLOS ONE (*n* = 52, 5%) followed by Oncotarget (43; 4%) and Medicine (42; 4%).

### Comprehensiveness of reporting in network meta-analyses

#### Reporting score overall, until and after 2015

The modified PRISMA-NMA of 49 items had a mean score 32.1 (95% CI 31.8–32.4; Appendix Fig. 4a; Appendix Fig. 5a). The mean score of NMAs published until and after 2015 were 31.3 (95% CI 30.8–31.8) and 32.6 (95% CI 32.2–33.0) (Appendix Figs. 4b and 5b). Although reporting score increased per year across NMAs, the increase was small and not statistically significant (trend test *p*-value = 0.480, Appendix Fig. 6).

Findings from univariable regression analysis showed that after 2015 reporting of NMAs improved by an average score of 1.25 (95% CI 0.59 to 1.91; Table 2). Univariable regression analysis showed that the PRISMA-NMA score until 2015 is positively associated with year, and for 1-year increase the score increases by 0.96 items (95% CI 0.32 to 1.59). For NMAs published after 2015, the score increases by 0.53 (95% CI 0.02 to 1.04) for 1-year increase. Focusing only on the NMA-specific items S1-S5, the speed of improvement was higher before the publication of the PRISMA-NMA guidelines (average per-year score increase in items S1–S5: 2013–2015 0.32 95% CI 0.14 to 0.49; 2016–2018 0.22 95% CI 0.11 to 0.33; Appendix Table 3).

**Table 2:** Univariable and multivariable regression using the modified PRISMA-NMA

Covariates	Interpretation of the coefficient	Coefficient (95% CI)	Sample size
**Univariable analyses and subgroups**			
**Published after 2015 vs until 2015**	Average increase in the score after 2015	1.25 (0.59, 1.91)	Before 2015: 389 After 2015: 755
**Year of publication, subgroup: only NMAs published before 2015**	Average increase in the score per year	0.96 (0.32, 1.59)	
**Year of publication, subgroup: only NMAs published after 2015**	Average increase in the score per year	0.53 (0.02, 1.04)	
**Multivariable analyses with year as a continuous variable**			
**Year of publication**	Average increase in the score per year	0.34 (0.16, 0.52)	Year 2013: 91 (reference group) Year 2014: 104 Year 2015: 194 Year 2016: 198 Year 2017: 316 Year 2018: 241
**Treatment type**	Average increase in the score if network includes pharmacological treatments	− 0.66 (− 1.34, 0.02)	Pharmacological treatments: 907 Non-pharmacological treatments (reference group): 237
**Funding type**	Average increase in the score if non-sponsored/publicly sponsored	1.34 (0.56, 2.11)	Non-sponsored/publicly sponsored/not reported: 974 Industry/mixed sponsored (reference group): 170
**Review type**	Average increase in the score if protocol is not available/reported	− 5.12 (− 5.74, − 4.49)	With protocol (reference group): 313 Without protocol: 831
**Impact factor**	Average increase in the score per impact factor increase (1 unit)	0.10 (0.07, 0.13)	
**Multivariable analyses with year as a dichotomous variable**			
**Year of publication**	Average increase in the score per year	0.81 (0.23, 1.39)	Before 2015 (reference group):389 After 2015:755
**Treatment type**	Average increase in the score if network includes pharmacological treatments	− 0.72 (− 1.40, − 0.04)	Pharmacological treatments: 907 Non-pharmacological treatments (reference group): 237
**Funding type**	Average increase in the score if non-sponsored/publicly sponsored	1.36 (0.58, 2.14)	Non-sponsored/publicly sponsored/Not reported: 974 Industry/mixed sponsored (reference group): 170
**Review type**	Average increase in the score if protocol is not available/reported	− 5.18 (− 5.8, − 4.55)	With protocol (reference group): 313 Without protocol: 831
**Impact factor**	Average increase in the score per impact factor increase (1 unit)	0.10 (0.06, 0.13)	

*CI* confidence interval

#### Factors that impact on reporting

**Fig. 3:**
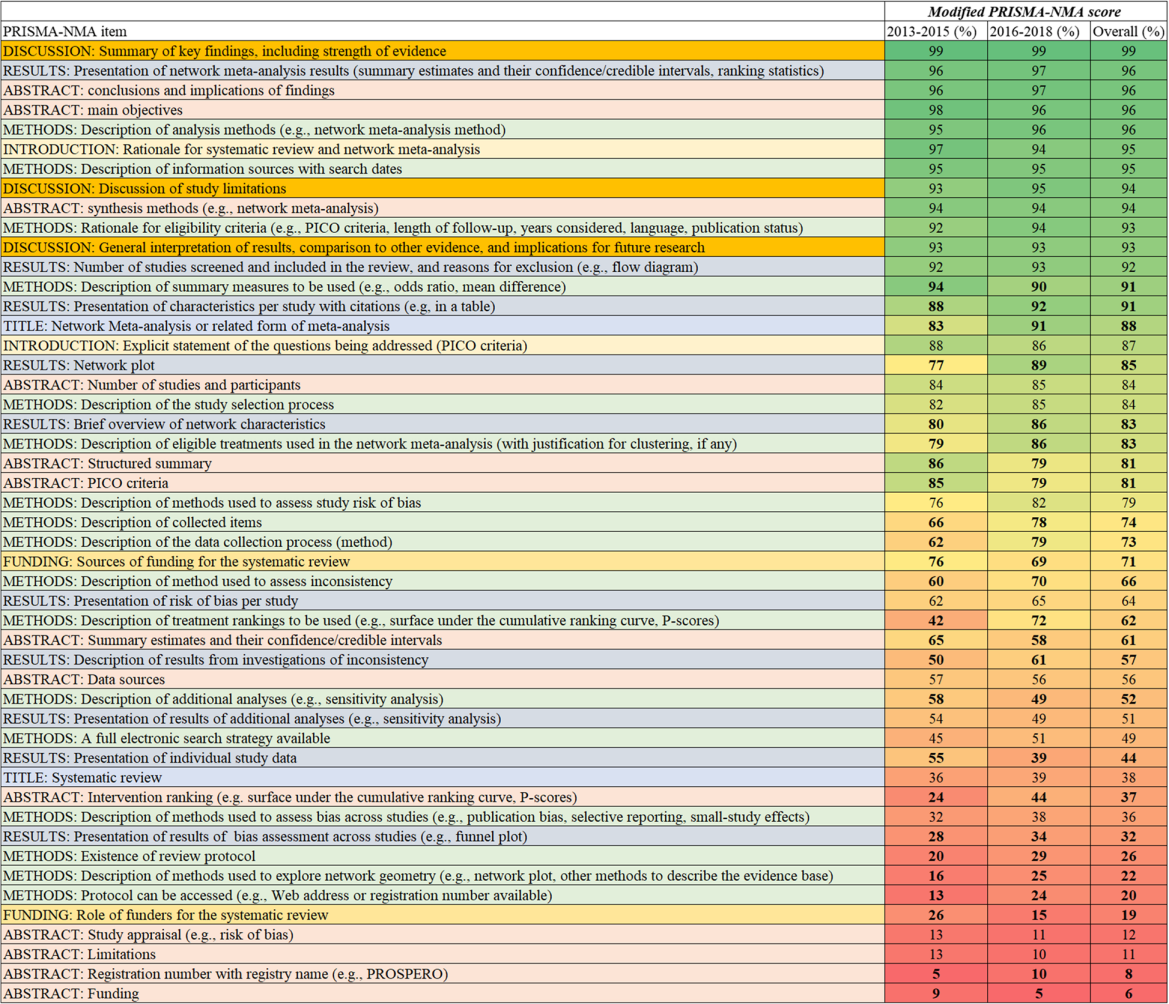
Plot of the percentage of adequately reporting the 49 modified PRISMA-NMA items overall and according to publication interval 2013–2015 and 2016–2018. PRISMA items are ordered from least to most well reported irrespective publication year. Statistically significant differences are indicated with a bold font. Each cell is coloured according to the reporting using the transformation of three colours: red (0%), yellow (50%), and green (100%)

On average, articles published in journals endorsing the original PRISMA had higher modified score (mean 34.5, 95% CI 33.8–35.2) compared with articles published in journals not explicitly endorsing PRISMA (median 31.7, IQR 21.4–32.0), yet this is not substantial (Fig. 3c; Appendix Fig. 4c). Overall, reporting of NMAs has been improved within each journal after 2015 (Appendix Table 4). Reporting did not vary substantially across continents (Appendix Fig. 7(c)(d)). Reporting differed across the types of reviews, with Cochrane reviews being associated with the highest scores (Cochrane reviews: mean 36.8, 95% CI 35.6–38.0; non-Cochrane reviews with protocol: mean 36.2, 95% CI 35.7–36.7; non-Cochrane reviews without a protocol median 30.6, 95% CI 29.8–30.4, Appendix Fig. 7(i)(j)).

Findings from multivariable regression analysis including year as a continuous variable showed that there was an increase in PRISMA score per year by 0.34 items (95% CI increase 0.16 to 0.52) when adjusting for journal impact factor, type of review, funding, and treatment types being compared in the network. Reporting was analogous to the journal impact factor (average score-increase 0.10 items, 95% CI increase 0.07 to 0.13). A positive association was also observed with publicly/non-sponsored NMAs (average score-increase 1.34 items, 95% CI increase 0.56 to 2.11; mean score: 32.5, 95% CI 32.2–32.8). Findings from multivariable regression analysis including year as a binary variable (before and after 2015) were in agreement with the multivariable regression and the year as a continuous variable, and suggested that there was an improvement in PRISMA score after 2015 by 0.81 items (95% CI increase 0.23 to 1.39) when adjusting for journal impact factor, type of review, funding, and treatment types being compared in the network. Conversely, a negative association was observed between the PRISMA-NMA score and reviews without a protocol (average score-decrease 5.18 items, 95% CI decrease 4.55 to 5.80; mean score 30.6, 95% CI 30.2–30.9), and networks including pharmacological treatments (average score-decrease 0.71 items 95% CI decrease 0.04 to 1.40; mean score 31.8, 95% CI 31.4–32.1; Table 2; Appendix Table 3).

#### PRISMA-NMA items that improved and items that still need attention

The percentage of adequately reported items before and after the PRISMA-NMA statement publication is presented in Fig. 3. Reporting was improved in 23 (47%) items, including the five items (S1-S5) specific to the reporting of the synthesis in NMAs. Overall, of the 958 with at least one closed loop, 693 networks (72%) reported consistency investigation, and this has improved over the years (range 52–78% NMAs). After 2015, improvement was also observed in items about the overview of the network and study characteristics.

However, reporting of several other items has shown little or no improvement after 2015, while adequate reporting of some items has declined, signalling a need for further attention. Description of summary effect sizes to be used, presentation of individual study data, sources of funding for the systematic review, and role of funders dropped in frequency after 2015 by 6–16% (Fig. 3). In particular, the role of funders for the systematic review was consistently missing across years for 71–88% NMAs (Appendix Table 5). A total of 294 NMAs (26%) reported the existence of a protocol, of which 229 (78%) reported a registry or a web site where a protocol could be accessed, and 79 (34%) of these NMAs reported this information in the abstract as well. Details on methods to assess bias across studies and on conducting additional analyses were underreported across all years.

## Discussion

Our findings suggest that key reporting items are missing in the majority of published NMAs. While minor improvements in the reporting of some elements were noted following PRISMA-NMA, other elements also experienced reductions. In total, reporting has improved after 2015 in 47% of the modified PRISMA-NMA items, but it has also deteriorated in 49% of the core items. Improvement was observed in items about the NMA synthesis, overview of the network and study characteristics, while deterioration was noticed in the description of summary effect sizes to be used, presentation of individual study data, sources of funding for the systematic review, and role of funders.

An explanation of the decrease in inadequately reported items may be restriction in the word count required by most journals. Also, some of the required details may be reported in the study’s protocol. Another key item that was inadequately reported was funding status. Journal guidelines highlight funding as crucial information to be reported in a paper; however, we noticed that mainly the author funding is reported and not funding for the review itself. Funding for the systematic review and role of funders are consistently underreported, which is a cause of concern. Presentation of individual study data is another item that has worsened after 2015. This may be because larger and more complex networks are being structured compared to past years or because of authors’ desire to retain ownership of the data, given the large efforts to compile the data sets, and to potentially publish new work after additional evidence (e.g. treatments) emerge.

Key factors that may impact the reporting were the journal’s impact factor, funding type, year of publication, type of review, and treatment category included in the network. In particular, newer and publicly sponsored NMAs of non-pharmacological therapies with a protocol, and published in high impact factor journals, were associated with better reporting. Our results showed that reviews with a protocol, and particularly Cochrane reviews, were associated with higher PRISMA-NMA scores.

Overall, reporting is adequate but not high (mean PRISMA-NMA score 32.1; 95% CI 31.8–32.4; max 49). Authors of NMAs showed a steep improvement in earlier years (2013–2015), but it stabilises after the PRISMA-NMA guideline publication. The improvement continues to exist throughout the years, but the speed of improvement is lower between 2016 and 2018. This suggests that overall, the PRISMA-NMA guidance has not importantly affected reporting in new NMAs compared to older NMAs. This may be because NMA authors in the earlier years 2013–2015 already followed existing guidelines for standards of conduct of NMA through the International Society for Pharmacoeconomics and Outcomes Research (ISPOR) tools [16, 17] and National Institute for Clinical Excellence (NICE) Decision Support Unit’s Evidence Synthesis Technical Support Documents (TSDs) documents [18]. However, we observed improvement in the five items (S1–S5) specific to the reporting of the synthesis section of an NMA after 2015, ranging between 4 and 12%. The improvement observed in reporting might be attributed at least in part to PRISMA-NMA, but may also be due to additional factors, such as the increase in registering or publishing of peer-reviewed protocols; protocol existence in NMAs has increased from 15–39% between 2013 and 2018. Our analysis showed that there is a slight improvement in reporting in the year 2016 compared to the year 2017 (2016: mean modified PRISMA-NMA score 33.0, 95% CI 32.3–33.8; 2017: mean modified PRISMA-NMA score 31.2, 95% CI 30.6–31.8). This may be due to the impact factor of the journal that NMAs were published in. The median impact factor of the journals the NMAs were published in 2016 was 3.87 (IQR 2.49–5.56), whereas in 2017 was 3.50 (IQR 2.63–5.16) (Appendix Fig. 8).

In our database of NMAs, the PRISMA-NMA guideline is only endorsed by 7% of the journals in which the NMAs were published. This highlights the need for journals publishing systematic reviews and NMAs to adopt the PRISMA-NMA guidelines to improve reporting, and to request the checklist upon a manuscript submission. Based on our findings, we provide recommendations to update the PRISMA-NMA statement to facilitate its use by systematic reviewers, journal editors, and peer reviewers (see the section “Conclusions and recommendations for practice”).

To the best of our knowledge, this is the largest review assessing the PRISMA extension guideline for NMA in more than 1000 systematic reviews and NMAs. Our findings are aligned with previous findings by Hutton et al*.* [11], who evaluated 89 NMAs of non-pharmacological therapies; Tonin et al. [10] who assessed 477 NMAs of pharmacological treatments; and Lee and Shin [9] who assessed 21 NMAs in dental care. In agreement with assessments in systematic reviews and meta-analyses, reporting in the 27 core PRISMA items was suboptimal [2]. A previous assessment on reporting of pairwise systematic reviews and meta-analyses on nursing interventions in patients with Alzheimer’s disease before and after PRISMA publication showed an improvement in the average core PRISMA items from 17.11 to 20.83 score [19]. Our findings about PRISMA-NMA are not limited to a specific disease area and showed that the average core PRISMA items score did not importantly change before (19.58 items) and after 2015 (19.88 items).

In addition to assessing the PRISMA-NMA items in the included systematic reviews, we explored factors that play a key role in reporting of NMA. In agreement with Zarin et al. [7], we found that the prerequisite assumptions are not always considered; 28% of NMAs (265 of 958 NMAs with a closed loop) did not report an assessment for consistency in their methods. However, similar to Petropoulou et al.’s [8] findings, reporting improved a bit over the years.

A limitation of our study is that in our assessment we considered that a PRISMA component was reported only if relevant information was present in the underlying section of the manuscript, as indicated in the PRISMA-NMA guideline. Also, protocols were not assessed for reporting relevant details, since the PRISMA-NMA guideline refers only to the final manuscript for NMAs. In our study, we have not explored differences in NMAs pointing authors to the relevant protocols for methods details and the remaining NMAs. However, in our assessment, we considered all available supplementary files and appendices. Although we may have missed some details reported in the protocol, we expect that this could not importantly impact our results. Also, an important unmeasured confounder in reporting may have been journals with no word count restrictions, but we have not assessed this further. Another potential limitation is that our literature search was conducted up to July 2018, and we may have missed recently published NMAs that were reported well. Also, the impact of the PRISMA-NMA guideline may not immediately be seen in the reporting of published NMAs and may take more time to start using it. However, this is the largest NMA database that assessed reporting, and we expect that no major differences would be seen in our results regarding the overall trend in reporting. A risk of confounding may be associated with our results between industry-sponsored studies and pharmacological treatments. We found that both factors were associated with a decrease in reporting (of the 170 industry-sponsored NMAs, 162 [95%] included pharmacological interventions only [with or without a placebo] in the network). We used a binary system (presence/absence) for the PRISMA-NMA items, but this may not be the best approach to assess adequacy of reporting. For example, authors may report that transitivity was assessed but without providing more details on this.

### Conclusions and recommendations for practice

NMAs published after 2015 more frequently reported the five items associated with NMA (i.e. description of methods to explore network geometry, description of methods to assess inconsistency, network plot presentation, brief overview of network characteristics, description of results from investigations of inconsistency). However, several important items are underreported and the yearly improvement in reporting is small.

In conducting this research, we chose to split certain PRISMA-NMA items into more specific items for evaluation, moving from 32 to 49 items. This allowed us to highlight crucial aspects of NMA that were or were not reported across the years. To this end, we suggest that the PRISMA-NMA checklist be updated using the 49 items instead of the initially suggested 32 items. This will provide more in-depth guidance to review authors, reviewers, editors, and readers for adequate reporting in NMA. The 49 items are listed in Appendix Table 1. Clarifying the information presented in the PRISMA-NMA 32 items into 49 different items may increase word count, but will enhance transparency of reporting. Online appendix files can also be used for additional and supporting information of the systematic review and NMA.

The original or modified PRISMA-NMA guidelines should be used extensively by review authors and be adopted by a wider range of journals. Journals editors, peer-reviewers, and systematic review authors should use the PRISMA-NMA list on a regular basis to write evaluate and publish results from NMA, paying special attention to items that are still underreported as highlighted in Fig. 3.

## Supplementary Information

**Additional file 1: Appendix 1**. Eligibility criteria, screening, study selection, and data abstraction. Appendix Tables. Appendix Figures.

**Additional file 2:** References of included studies.

## Data Availability

The datasets used and/or analysed during the current study are available from the corresponding author on reasonable request.
