# Correction: Do reporting guidelines have an impact? Empirical assessment of changes in reporting before and after the PRISMA extension statement for network meta-analysis

**DOI:** 10.1186/s13643-022-01988-3

**Published:** 2022-06-03

**Authors:** Areti Angeliki Veroniki, Sofia Tsokani, Stella Zevgiti, Irene Pagkalidou, Katerina-Maria Kontouli, Pinar Ambarcioglu, Nikos Pandis, Carole Lunny, Adriani Nikolakopoulou, Theodoros Papakonstantinou, Anna Chaimani, Sharon E. Straus, Brian Hutton, Andrea C. Tricco, Dimitris Mavridis, Georgia Salanti

**Affiliations:** 1 Department of Primary Education, School of Education, University of Ioannina, Ioannina, Greece; 2 Knowledge Translation Program, Li Ka Shing Knowledge Institute, St. Michael’s Hospital, Toronto, ON Canada; 3 Department of Hygiene, Social-Preventive Medicine and Medical Statistics, Medical School, Aristotle University of Thessaloniki, Thessaloniki, Greece; 4 Department of Biostatistics, Faculty of Veterinary Medicine, Mustafa Kemal University, Tayfur Sökmen Kampüsü, 31060 Antakya, Hatay Turkey; 5 Department of Orthodontics and Dentofacial Orthopedics, Dental School/Medical Faculty, University of Bern, Bern, Switzerland; 6 Cochrane Hypertension Review Group and the Therapeutics Initiative, University of British Columbia, Vancouver, Canada; 7 Institute of Medical Biometry and Statistics, Faculty of Medicine and Medical Center, University of Freiburg, Freiburg, Germany; 8 Institute of Social and Preventive Medicine, University of Bern, Bern, Switzerland; 9 Université de Paris, Research Center of Epidemiology and Statistics Sorbonne Paris Cité (CRESS UMR1153), INSERM, INRA, Paris, France; 10 Cochrane France, Paris, France; 11 Department of Geriatric Medicine, University of Toronto, Toronto, ON Canada; 12 Ottawa Hospital Research Institute, Ottawa, ON Canada; 13 University of Ottawa School of Epidemiology and Public Health, Ottawa, ON Canada; 14 Epidemiology Division, Dalla Lana School of Public Health, University of Toronto, Toronto, ON Canada; 15 Paris Descartes University, Sorbonne Paris CitéFaculté de Médecine, Paris, France

**Correction: Syst Rev 10, 246 (2021)**

10.1186/s13643-021-01780-9

Following publication of the original article [1], the authors identified an error in Fig. 3. The correct figure is given below.

**Fig. 3:**
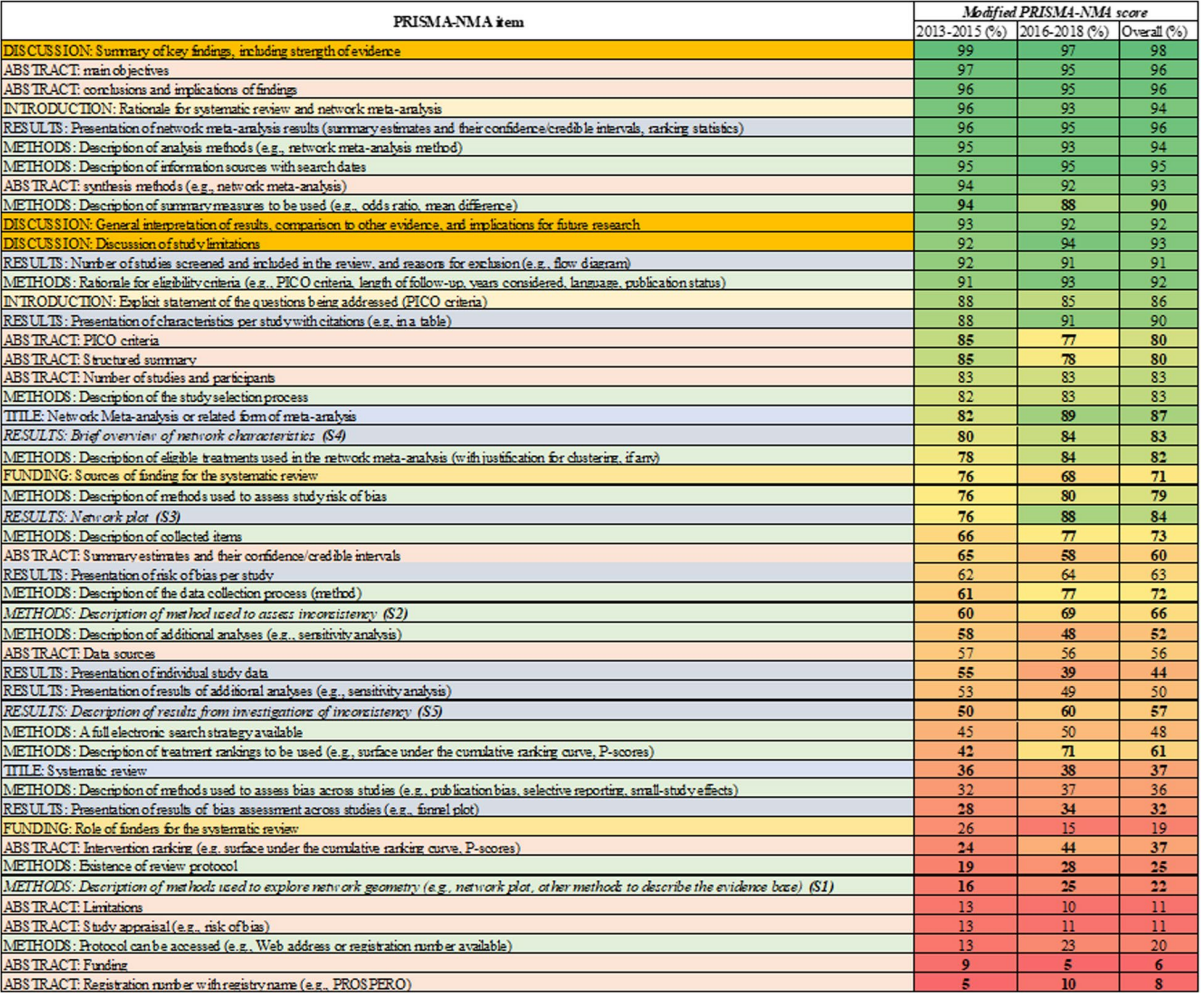
Plot of the percentage of adequately reporting the 49 modified PRISMA-NMA items overall and according to publication interval 2013–2015 and 2016–2018. PRISMA items are ordered from least to most well reported irrespective publication year. Statistically significant differences are indicated with a bold font. Each cell is coloured according to the reporting using the transformation of three colours: red (0%), yellow (50%), and green (100%)
