# Supplementary material for: Do reporting guidelines have an impact? Empirical assessment of changes in reporting before and after the PRISMA extension statement for network meta-analysis
Source: Syst Rev. 2021 Sep 10;10:246. doi: 10.1186/s13643-021-01780-9 (PMC8434710; doi:10.1186/s13643-021-01780-9)

Additional file 1

Appendix 1. Eligibility criteria, screening, study selection, and data abstraction 2

Appendix Table 1. *Original* and *Modified* PRISMA-NMA items 4

Appendix Table 2. List of included studies 5

Appendix Figure 1. Number of systematic reviews and network meta-analyses, and per-paper average *original* PRISMA-NMA score published between 2013 and 2018.............................5

Appendix Figure 2. Geographic heat map with published NMAs between 2013 and 2018 6

Appendix Figure 3. Distribution of journal impact factor 6

Appendix Figure 4. Distribution of *modified* PRISMA-NMA score: a) overall, b) before and after 2015, and c) per journals endorsing or not the PRISMA guidelines. 7

Appendix Figure 5. Distribution of *original* PRISMA-NMA score: a) overall, b) before and after 2015, and c) per journals endorsing or not the PRISMA guidelines 7

Appendix Figure 6. Distribution of *modified* PRISMA-NMA score per year of publication ...8

Appendix Table 3. Univariable and multivariable regression using the *original* PRISMA-NMA score...9

Appendix Table 4. Assessment of reporting quality per journal with impact factor >10........11

Appendix Figure 7. PRISMA-NMA score per study characteristics......................................12

Appendix Table 5. Assessment of reporting of NMAs using the *modified* PRISMA-NMA per year of publication 15

Appendix Figure 8. Distribution of journal impact factor in NMAs published in 2016 and 2017..18

Appendix 1. Eligibility criteria, screening, study selection, and data abstraction

We searched MEDLINE, EMBASE and the Cochrane Database of Systematic Reviews from inception until July, 2018 for network meta-analyses (NMAs) of randomized controlled trials (RCTs). An expert librarian compiled the literature search, which was peer-reviewed by a second librarian using the Peer Review of Electronic Search Strategies (PRESS) checklist [40]. The final search strategy for the MEDLINE database can be found in our previously published reviews.(1-3)

We included NMAs that compared at least four interventions (i.e., different drugs or other medical treatments, or different schedules, doses or formulations of the same treatment) including placebo, no treatment, waiting list or other control interventions. NMAs were eligible if they had conducted a valid statistical method for indirect comparisons (e.g., adjusted indirect comparison method (4)) or NMAs (e.g., hierarchical models). Published NMAs written in English were included. We excluded studies that applied an unadjusted indirect comparison,(5) a diagnostic test accuracy NMA, a NMA in animals, a NMA of non-randomized studies. We also excluded NMAs in which the number of trials was smaller than the number of interventions. In the present study, we restricted to articles published between 2013 and 2018, for an equal timeframe before and after the PRISMA-NMA publication.(6)

After a pilot-test of the eligibility criteria on a random sample of 30 articles, two reviewers (ST, SZ, IP) independently screened titles and abstracts, and similarly for relevant full-text papers. Conflicts were resolved by a third reviewer (AAV). Screening was performed using the online tool Synthesi.SR.(7)

A predefined data abstraction form was developed in REDCap.(8) Data abstraction was performed by a single reviewer (ST, SZ, IP, KMK, PA, NP, CL), and then data were checked by a second reviewer.

References

1. Petropoulou M, Nikolakopoulou A, Veroniki AA, Rios P, Vafaei A, Zarin W, et al. Bibliographic study showed improving statistical methodology of network meta-analyses published between 1999 and 2015. Journal of clinical epidemiology. 2017;82:20-8.
2. Zarin W, Veroniki AA, Nincic V, Vafaei A, Reynen E, Motiwala SS, et al. Characteristics and knowledge synthesis approach for 456 network meta-analyses: a scoping review. BMC medicine. 2017;15(1):3.
3. Nikolakopoulou A, Chaimani A, Veroniki AA, Vasiliadis HS, Schmid CH, Salanti G. Characteristics of networks of interventions: a description of a database of 186 published networks. PloS one. 2014;9(1):e86754.

4. Bucher HC, Guyatt GH, Griffith LE, Walter SD. The results of direct and indirect treatment comparisons in meta-analysis of randomized controlled trials. J Clin Epidemiol. 1997;50(6):683-91.
5. Song F, Loke YK, Walsh T, Glenny AM, Eastwood AJ, Altman DG. Methodological problems in the use of indirect comparisons for evaluating healthcare interventions: survey of published systematic reviews. BMJ. 2009;338:b1147.
6. Hutton B, Salanti G, Caldwell DM, Chaimani A, Schmid CH, Cameron C, et al. The PRISMA extension statement for reporting of systematic reviews incorporating network meta-analyses of health care interventions: checklist and explanations. Annals of internal medicine. 2015;162(11):777-84.
7. Knowledge Translation Program. Synthesi.SR. Toronto, Ontario: Li Ka Shing Knowledge Institute, St. Michael's Hospital; 2014. Available from: http://www.breakthroughkt.ca/login.php.
8. Papakonstantinou T. nmadata: R package for accessing redcap database of network meta-analyses hosted by ISPM University of Bern. Available at https://github.com/esm-ispm-unibe-ch/nmadata.,.

Appendix Table 1. *Original* and *Modified* PRISMA-NMA items

* Sub-items were not included in the *original* or *modified* PRISMA-NMA score

PRISMA STATEMENT Domains	Original PRISMA-NMA (Scored 1,if item reported-Max score 32)	Item Description	Modified PRISMA-NMA (Scored 1, if item reported-Max score 49)
**TITLE**			
Title **(OP1)**	1, if both terms reported	Systematic review **(MP1)**	1
		NMA/related form of MA **(MP2)**	1
**ABSTRACT**			
Structured summary **(OP2)**	1, if there is a structured summary, regardless the items included in the summary	Structured summary **(MP3)**	1
		Main objectives **(MP4)**	1
		Data sources **(MP5)**	1
		PICO **(MP6)**	1
		Study appraisal (e.g., risk of bias) **(MP7)**	1
		Synthesis methods e.g. NMA **(MP8)**	1
		Number of studies and participants **(MP9)**	1
		Summary estimates and their CIs/CrIs **(MP10)**	1
		Treatment ranking **(MP11)**	1
		Limitations **(MP12)**	1
		Conclusions and implications of findings **(MP13)**	1
		Funding **(MP14)**	1
		Protocol registration number with registry name (e.g., PROSPERO) **(MP15)**	1
**INTRODUCTION**			
Rationale **(OP3)**	1	Rationale for systematic review and NMA **(MP16)**	1
Objectives **(OP4)**	1	Explicit statement of the questions being addressed (PICOS) **(MP17)**	1
**METHODS**			
Protocol and registration **(OP5)**	1, if the existence of protocol is reported	Existence of review protocol **(MP18)**	1
		Protocol can be accessed (e.g., web address or registration number available) **(MP19)**	1
Eligibility criteria **(OP6)**	1, if the eligibility criteria are reported	Rationale for eligibility criteria (e.g., PICOS, length of follow-up, years considered, language, publication status) **(MP20)**	1
		Description of eligible treatments used in the NMA (with justification for clustering, if any) **(MP21)**	1
Information sources **(OP7)**	1	Description of information sources with search dates **(MP22)**	1
Search **(OP8)**	1	A full electronic search strategy available **(MP23)**	1
Study selection **(OP9)**	1	Description of the study selection process **(MP24)**	1
Data collection process **(OP10)**	1	Description of the data collection process (method) **(MP25)**	1
Data items **(OP11)**	1	Description of collected items **(MP26)**	1
Geometry of the network ***(OPS1)***	1	*Description of methods used to explore network geometry (e.g., network plot, other methods to describe the evidence base)* ***(MP27) - S1***	1
Risk of bias within individual studies **(OP12)**	1	Description of methods used to assess study risk of bias **(MP28)**	1
Summary measures **(OP13)**	1, if the summary measures used are reported	Description of summary measures to be used (e.g., OR, RR, MD, SMD) **(MP29)**	1
		Description of treatment rankings to be used (e.g., SUCRA, P-scores) **(MP30)**	1
Planned methods of analysis **(OP14)**	1	Description of analysis e.g., NMA method **(MP31)**	1
Assessment of inconsistency ***(OPS2)***	1	*Description of method used to assess inconsistency* ***(MP32) - S2***	1
Risk of bias across studies **(OP15)**	1	Description of methods used to assess bias across studies (e.g., publication bias, selective reporting, small study effects) **(MP33)**	1
Additional analyses **(OP16)**	1	Description of additional analyses (e.g., sensitivity analysis) **(MP34)**	1
	*Subitems for item "Planned methods of analysis"**	*Description of analysis e.g. NMA method (Handling of multi-arm trials)*	*Subitems for "Planned methods of analysis"**
		Description of analysis e.g. NMA method (Selection of variance structure)	
		Description of analysis e.g. NMA method (If Bayesian analysis, selection of prior distributions in Bayesian analyses)	
		Description of analysis e.g. NMA method (If Bayesian analysis, assessment of model fit)	
	*Subitems for item "Additional Analyses"**	*Description of additional analyses (e.g., sensitivity analysis (Sensitivity or subgroup analyses)*	*Subitems for item "Additional Analyses"**
		Description of additional analyses (e.g., sensitivity analysis (Meta-regression analyses)	
		Description of additional analyses (e.g., sensitivity analysis (Alternative formulations of the treatment network)	
		Description of additional analyses (e.g., sensitivity analysis (If Bayesian analysis, use of alternative priors)	
**RESULTS**			
Study selection **(OP17)**	1	Number of studies screened and included in the review, and reasons for exclusion (e.g., flow diagram) **(MP35)**	1
Presentation of network structure (	1	*Network plot* ***(MP36) - S3***	1
Summary of network geometry ***(OI***	1	*Brief overview of network characteristics* ***(MP37) - S4***	1
Study characteristics **(OP18)**	1	Presentation of characteristics per study with citations (e.g, in a table) **(MP38)**	1
Risk of bias within studies **(OP19)**	1	Presentation of risk of bias per study **(MP39)**	1
Results of individual studies **(OP20)**	1	Presentation of individual study data **(MP40)**	1
Synthesis of results **(OP21)**	1	Presentation of NMA summary estimates and CIs/CrIs **(MP41)**	1
Exploration for inconsistency ***(OPS***	1	*Description of results from investigations of inconsistency* ***(MP42) - S5***	1
Risk of bias across studies **(OP22)**	1	Presentation of results of bias assessment across studies (e.g., funnel plot) **(MP43)**	1
Results of additional analyses **(OP2**	1	Presentation of results of additional analyses (e.g., sensitivity analysis) **(MP44)**	1
	*Subitems for item "Results of individual studies"**	*Presentation of individual study data (summary data for each intervention group (e.g, 2x2 table)*	*Subitems for item "Results of individual studies"**
		Presentation of individual study data (effect estimates and confidence intervals (e.g, inverse variance data)	
	*Subitems for item "Synthesis of results"**	*Presentation of NMA summary estimates and CIs/CrIs (league tables)*	*Subitems for item "Synthesis of results"**
		Presentation of NMA summary estimates and CIs/CrIs (forest plots)	
		Presentation of NMA summary estimates and CIs/CrIs (ranking statistics (e.g., SUCRAs, rankograms, rank-heat plot)	
**DISCUSSION**			
Summary of evidence **(OP24)**	1	Summary of key findings, including strength of evidence **(MP45)**	1
Limitations **(OP25)**	1	Discussion of study limitations **(MP46)**	1
Conclusions **(OP26)**	1	General interpretation of results, comparison to other evidence, and implications for future research **(MP47)**	1
**FUNDING**			
Funding **(OP27)**	1, if funding source is reported	Sources of funding for the systematic review **(MP48)**	1
		Role of funders for the systematic review **(MP49)**	1
	*Subitems for item "Funding"**	*Role of funders for the systematic review (funding has been received from manufacturers of treatments in the*	*Subitems for item "Funding"**
		Role of funders for the systematic review (some of the authors are content experts with professional conflicts of interest that could affect use of treatments in the network	

Appendix Table 2. List of included studies
See Supplementary file 2

Appendix Figure 1. Number of systematic reviews and network meta-analyses, and per-paper average *original* PRISMA-NMA score published between 2013 and 2018.
'*' denotes that the search was performed up to July 2018, and thus only 7 months of that year are reflected in this graph. Error bars parallel to the y-axis represent the uncertainty of the *original* PRISMA-NMA score.

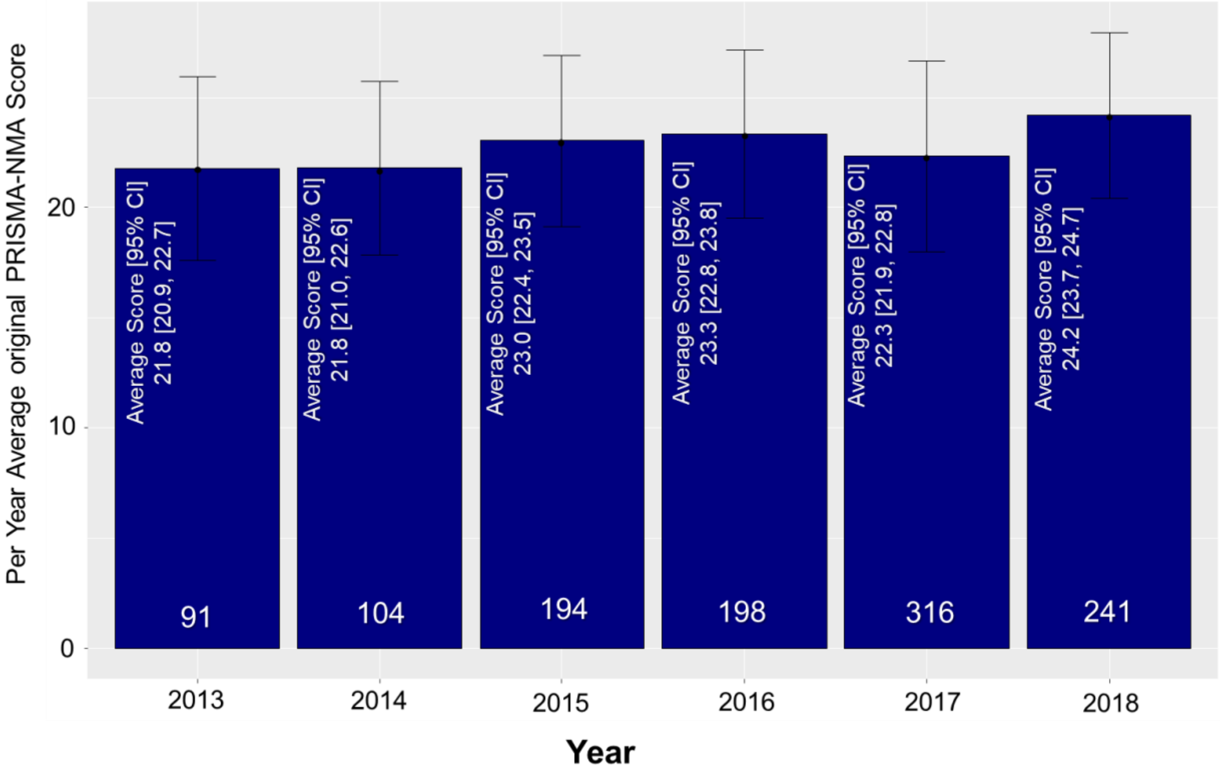

Appendix Figure 2. Geographic heat map with published NMAs between 2013 and 2018

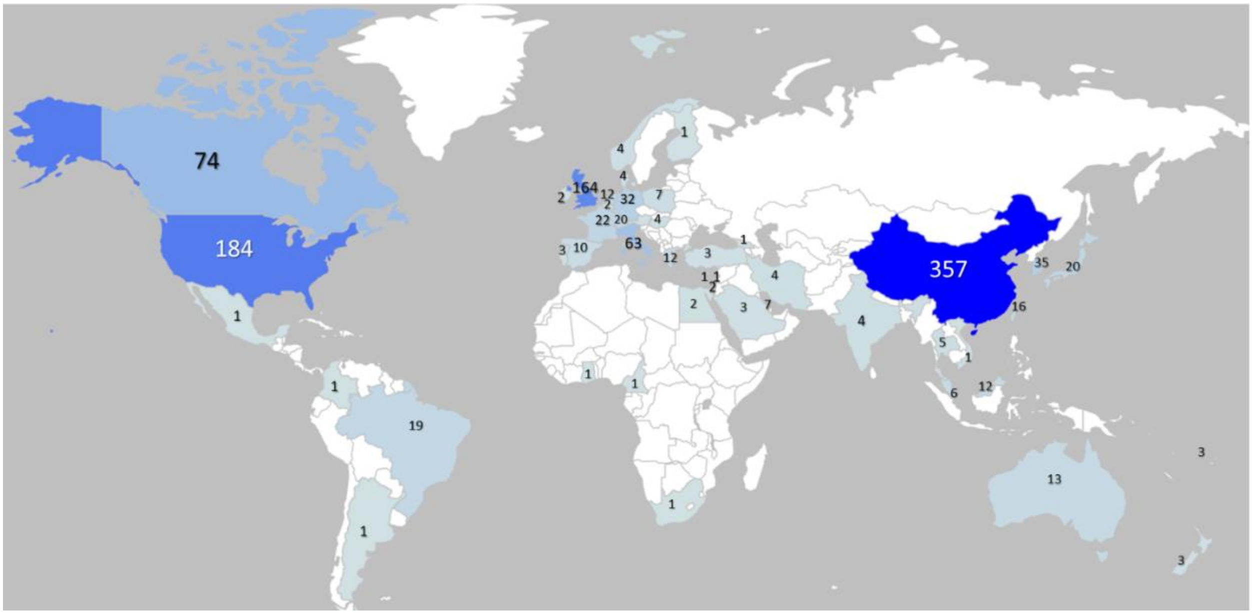

Appendix Figure 3. Distribution of journal impact factor

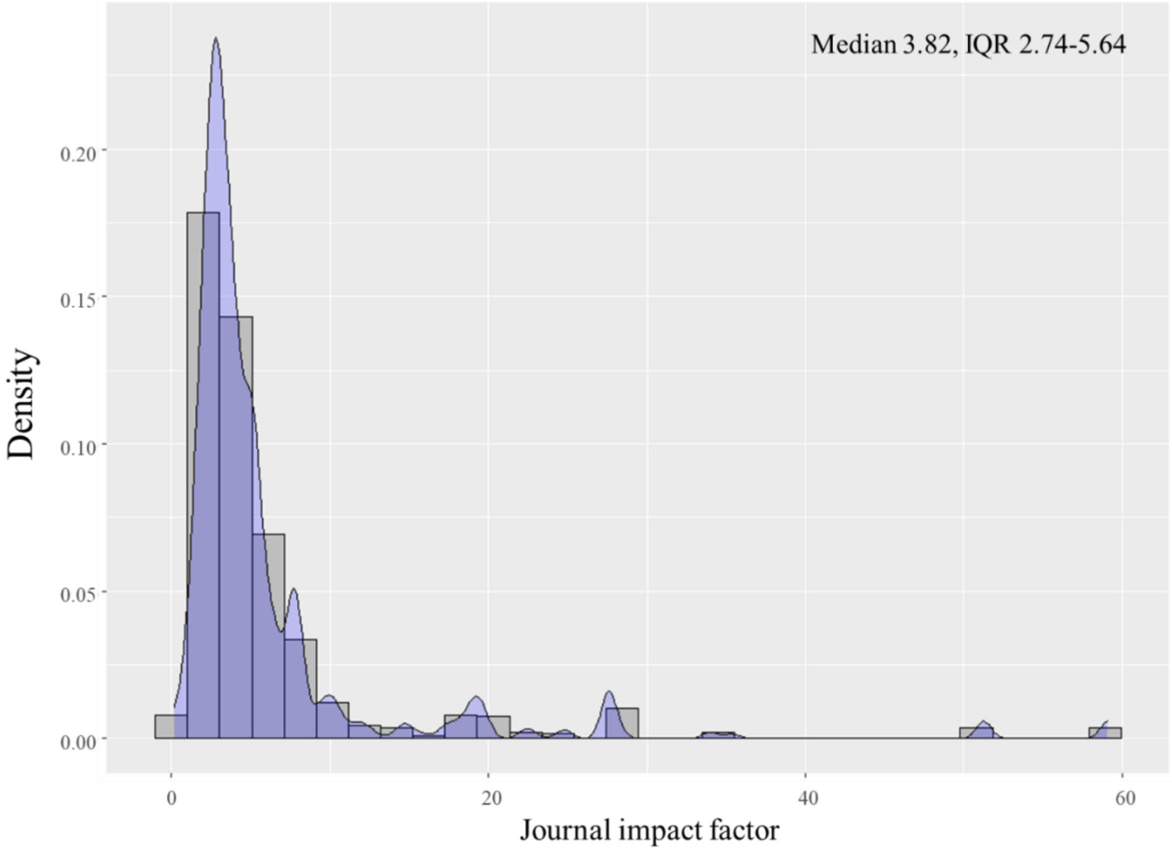

Appendix Figure 4. Distribution of *modified* PRISMA-NMA score: a) overall, b) before and after 2015, and c) per journals endorsing or not the PRISMA guidelines.

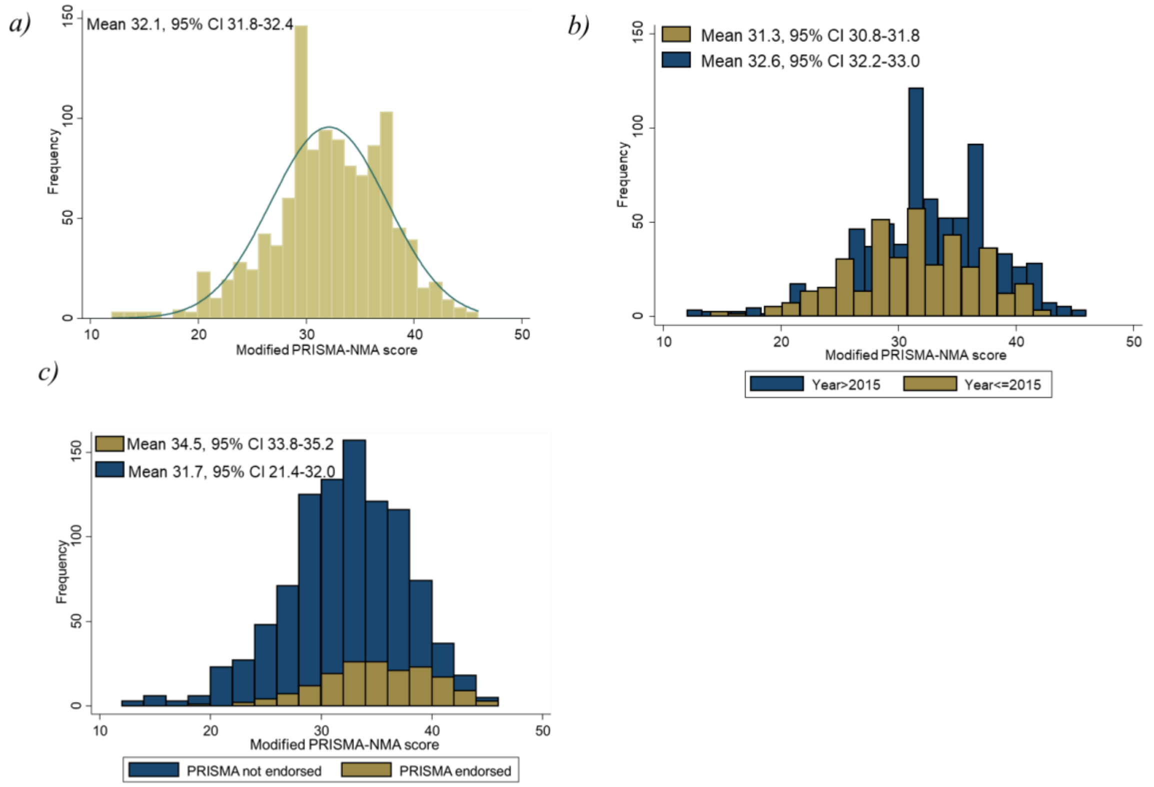

Appendix Figure 5. Distribution of *original* PRISMA-NMA score: a) overall, b) before and after 2015, and c) per journals endorsing or not the PRISMA guidelines

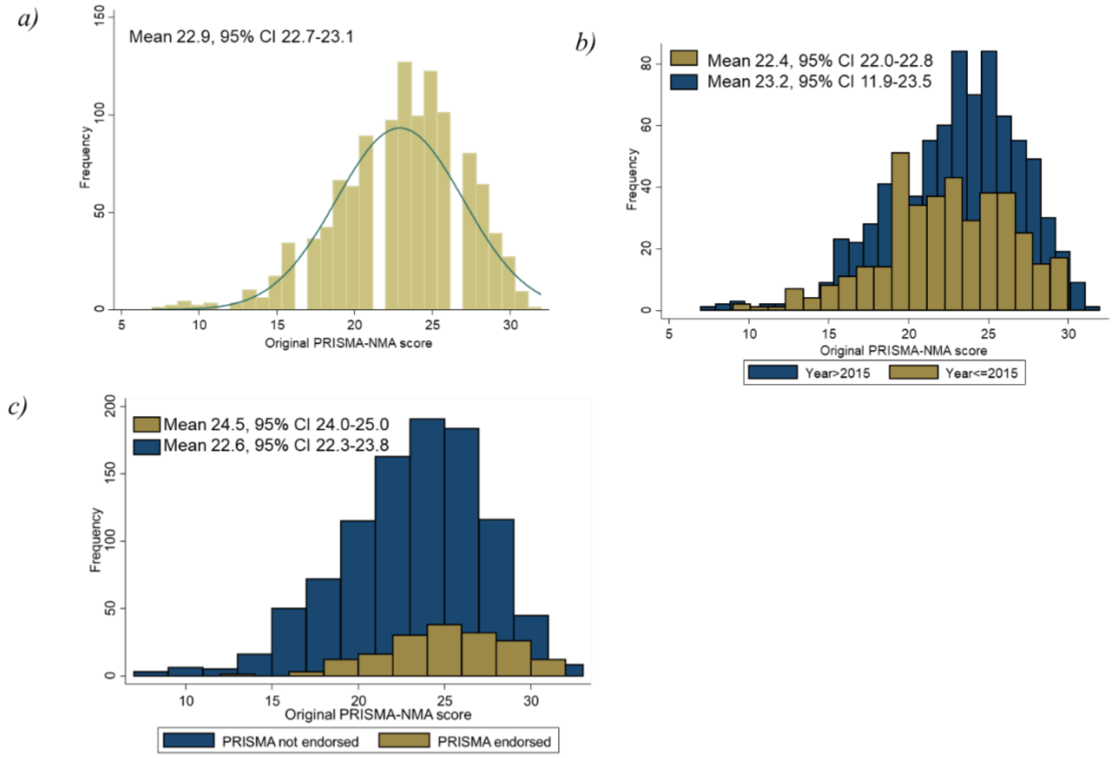

Appendix Figure 6. Distribution of *modified* PRISMA-NMA score per year of publication

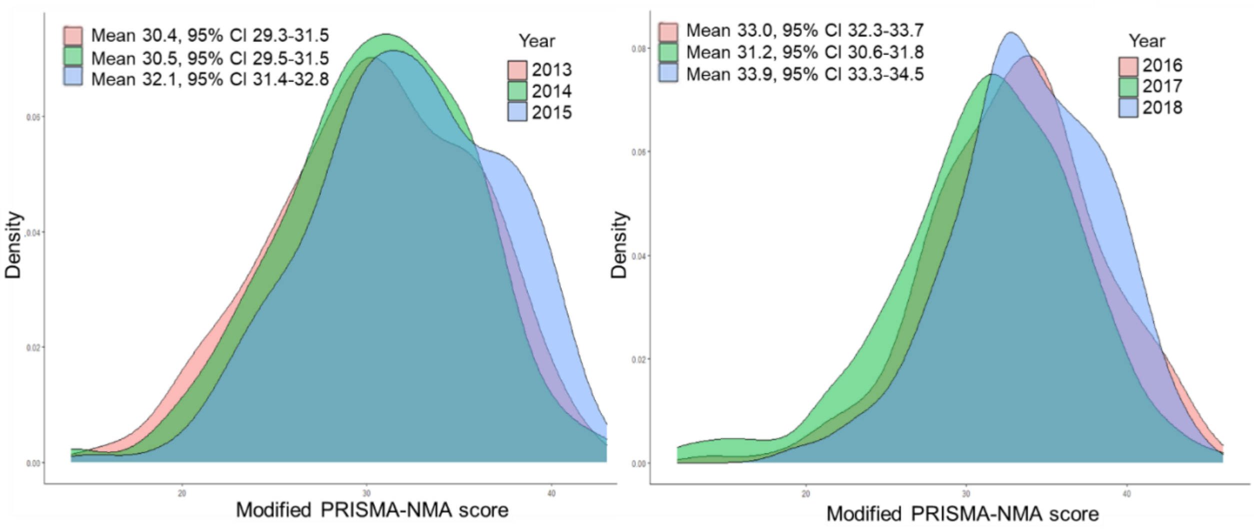

Appendix Table 3. Univariable and multivariable regression using the *original* PRISMA-NMA score

Covariates	Interpretation of the coefficient	Coefficient (95% CI)		Sample size
		Original PRISMA-NMA Score	S1-S5 Score	
**Univariable analyses and subgroups**				
**Published after 2015 vs until 2015**	Average increase in the score after 2015	0.69 (0.21, 1.18)	0.32 (0.14, 0.49)	Before 2015: 389<br><br>After 2015: 755
**Year of publication, subgroup: only NMAs published before 2015**	Average increase in the score per year	0.48 (0.10, 0.86)	0.22 (0.11, 0.33)	
**Year of publication, subgroup: only NMAs published after 2015**	Average increase in the score per year	0.77 (0.28, 1.28)	0.48 (0.32, 0.64)	
**Multivariable analyses with year as a continuous variable**				
**Year of publication**	Average increase in the score per year	0.23 (0.09, 0.37)	0.18 (0.13, 0.23)	Year 2013 (reference group): 91<br>Year 2014: 104<br>Year 2015: 194<br>Year 2016: 198<br>Year 2017: 316<br>Year 2018: 241
**Treatment type**	Average increase in the score if network includes pharmacological treatments	-0.49 (-1.02, 0.04)	-0.16 (-0.34, 0.02)	Pharmacological treatments: 907<br><br>Non-pharmacological treatments (reference group): 237
**Funding type**	Average increase in the score if non-sponsored/publicly-sponsored	0.99 (0.38, 1.59)	0.49 (0.28, 0.7)	Non-sponsored/publicly-sponsored/Not reported: 974<br><br>Industry/Mixed sponsored (reference group): 170
**Review type**	Average increase in the score if protocol is not available/reported	-3.44 (-3.92, -2.95)	-0.28 (-0.45, -0.11)	With protocol (reference group): 313<br><br>Without protocol: 831
**Impact factor**	Average increase in the score per	0.06 (0.04, 0.09)	0.02 (0.01, 0.03)	

	impact factor increase (1 unit)			
**Multivariable analyses with year as a dichotomous variable**				
**Year of publication**	Average increase in the score after 2015	0.46 (0.01, 0.92)	0.44 (0.28, 0.60)	Before 2015(reference group):389<br>After 2015:755
**Treatment type**	Average increase in the score if network includes pharmacological treatments	-0.52 (-1.06, 0.00)	-0.19 (-0.37, 0.00)	Pharmacological treatments: 907<br><br>Non-pharmacological treatments (reference group): 237
**Funding type**	Average increase in the score if non-sponsored/publicly-sponsored	1.01 (0.40, 1.62)	0.50 (0.29, 0.71)	Non-sponsored/publicly-sponsored/Not reported: 974<br><br>Industry/Mixed sponsored (reference group): 170
**Review type**	Average increase in the score if protocol is not available/reported	-3.49 (-3.97, -3.00)	-0.31 (-0.48, -0.14)	With protocol (reference group): 313<br><br>Without protocol: 831
**Impact factor**	Average increase in the score per impact factor increase (1 unit)	0.06 (0.03, 0.09)	0.02 (0.01, 0.03)	

Appendix Table 4. Assessment of reporting quality per journal with impact factor >10

In **bold** we highlight journals that endorse the PRISMA checklist

Journal (Impact Factor >10)	Total # of NMAs	Percentage of reported items in NMAs published <=2015 (mean)	Percentage of reported items in NMAs published >2015 (mean)	Mean Percentage Difference (95% CI)	Percentage of reported items in NMAs published <=2015 (mean)	Percentage of reported items in NMAs published >2015 (mean)	Mean Percentage Difference (95% CI)
		Original PRISMA-NMA score			*Modified* PRISMA-NMA score		
**Lancet (60.392)**	**8**	**78%**	**87%**	**9% (-5%, 23%)**	**76%**	**84%**	**8% (-3%, 21%)**
**Jama-Journal Of The American Medical Association (45.54)**	**8**	**73%**	**85%**	**12% (6%, 19%)**	**67%**	**80%**	**13% (8%, 17%)**
World Psychiatry (40.595)	3	72%	83%	11%*	65%	77%	12%*
Lancet Oncology (33.752)	2	81%	NA	NA	72%	NA	NA
Journal of Clinical Oncology (32.956)	1	NA	56%	NA	NA	55%	NA
**Bmj (30.223)**	**21**	**79%**	**88%**	**9% (1%, 15%)**	**71%**	**82%**	**11% (4%, 19%)**
Lancet Diabetes & Endocrinology (25.34)	1	78%	NA	NA	76%	NA	NA
Jama Oncology (24.799)	4	72%	66%	-6%*	65%	62%	-3%*
Circulation (23.603)	1	75%	NA	NA	67%	NA	NA
European Heart Journal (22.673)	3	NA	57%	NA	NA	50%	NA
Annals Of Internal Medicine (21.317)	11	81%	85%	4% (-6%, 15%)	79%	80%	1% (-6%,8%)
Journal Of The American College Of Cardiology (20.589)	4	64%	69%	5%*	56%	59%	3%*
Gut (19.819)	3	NA	84%	NA	NA	76%	NA
Annals Of Oncology (18.274)	1	NA	65%	NA	NA	67%	NA
European Urology (17.947)	5	69%	72%	3% (-19%, 25%)	63%	64%	1% (-15%, 17%)
Intensive Care Medicine (17.679)	4	66%	70%	4%*	61%	65%	4%*
Jama Psychiatry (17.471)	2	NA	77%	NA	NA	74%	NA
Gastroenterology (17.373)	5	86%	86%	0% (-12%, 11%)	76	78%	2% (-8%, 12%)
Lancet Psychiatry (16.209)	2	NA	84%	NA	NA	85%	NA
Lancet Hiv (14.813)	3	78%	81%	3%*	69%	76%	7%*
Lancet Gastroenterology & Hepatology (14.789)	1	NA	75%	NA	NA	69%	NA
Hepatology (14.679)	2	81%	84%	3%*	73%	73%	0%*
Jama Surgery (13.625)	1	NA	91%	NA	NA	82%	NA
Jama Neurology (13.608)	1	75%	NA	NA	65%	NA	NA
Journal Of Thoracic Oncology (13.357)	2	50%	34%	-16%*	45%	31%	-14%*
Jama Cardiology (12.794)	1	NA	88%	NA	NA	78%	NA
British Journal Of Sports Medicine (12.022)	4	59%	85%	26%*	55%	78%	23%*
European Journal Of Heart Failure (11.627)	1	53%	NA	NA	53%	NA	NA
Jnci-Journal Of The National Cancer Institute (11.577)	4	75%	80%%	5%*	69%	71%	2%*
Ageing Research Reviews (10.616)	1	NA	8%	NA	NA	78%	NA
**Plos Medicine (10.5)**	**2**	**72%**	**97%**	**25***	**69%**	**92%**	**23%***
Journal Of Allergy And Clinical Immunology (10.228)	1	66%	NA	NA	57%	NA	NA
American Journal Of Gastroenterology (10.171)	2	NA	77%	NA	NA	67%	NA
Annals Of Surgery (10.13)	2	66%	56%	-1%*	61%	53%	-8%*
Clinical Cancer Research (10.107)	1	41%	NA	NA	41%	NA	NA

* Inadequate number of studies per group to calculate a 95% confidence interval

Appendix Figure 7. PRISMA-NMA score per study characteristics

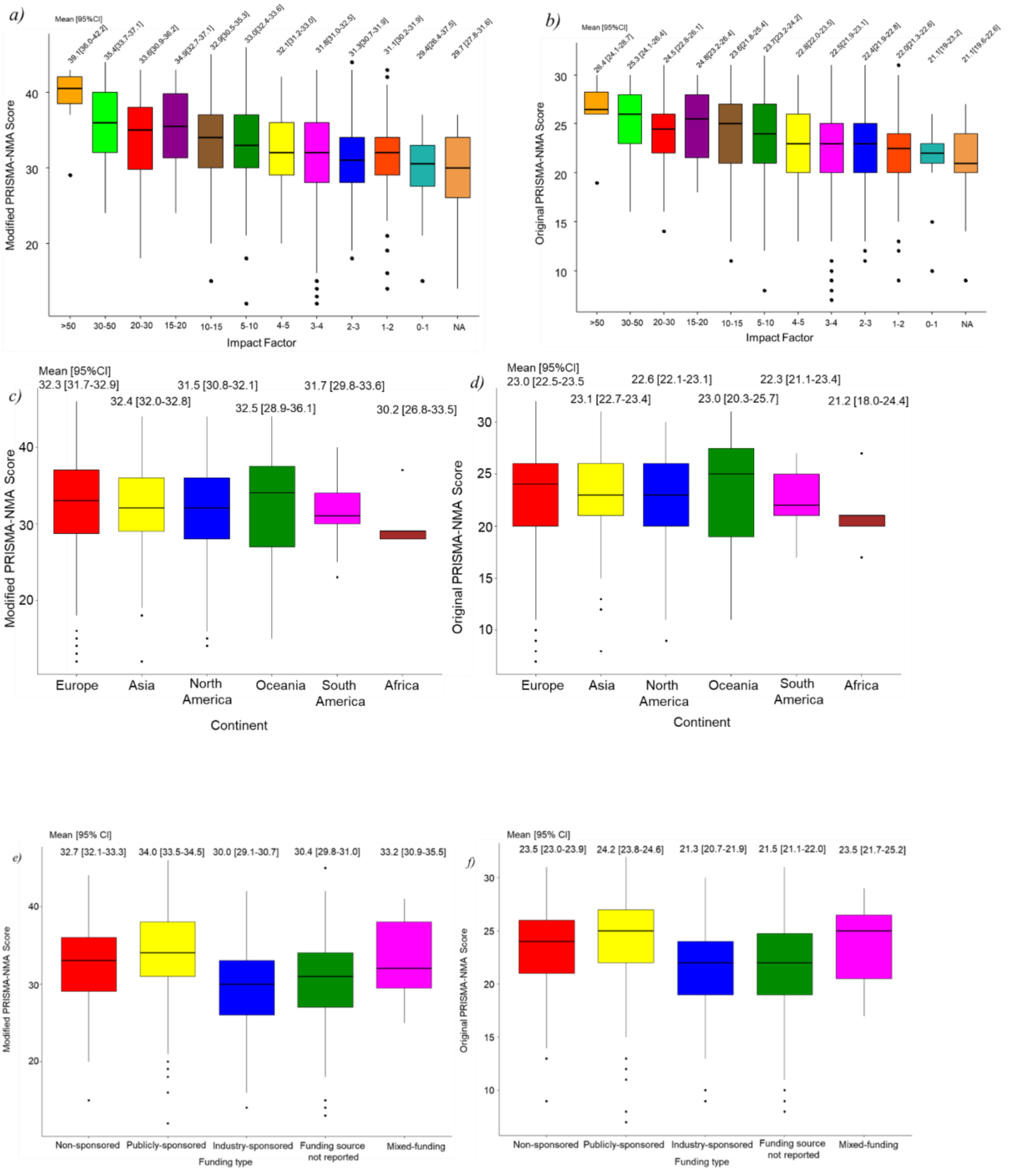

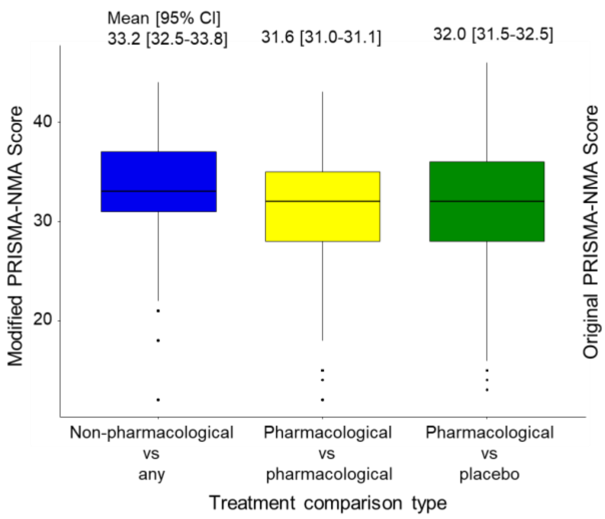

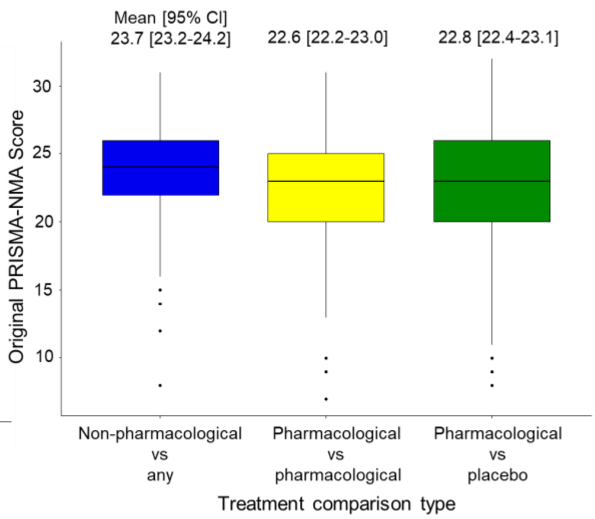

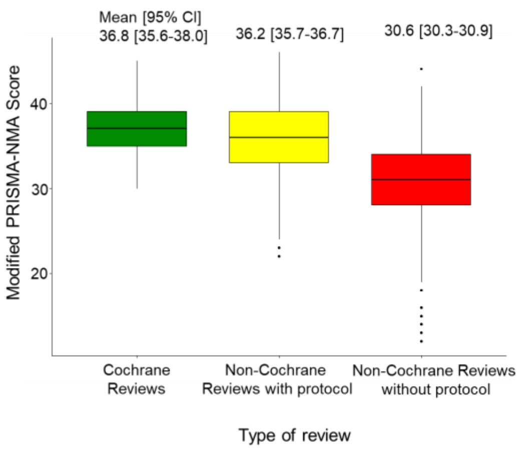

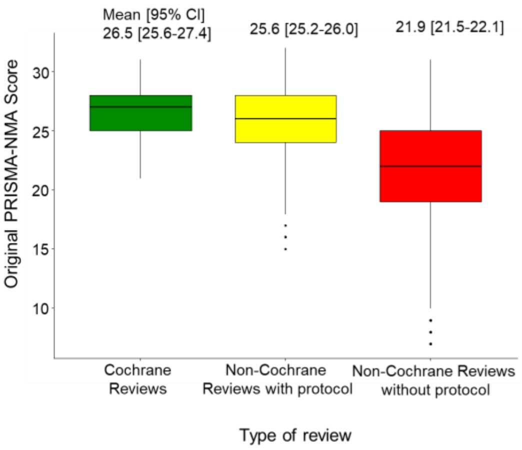

Dichotomous variables as included in the multivariable regression analysis

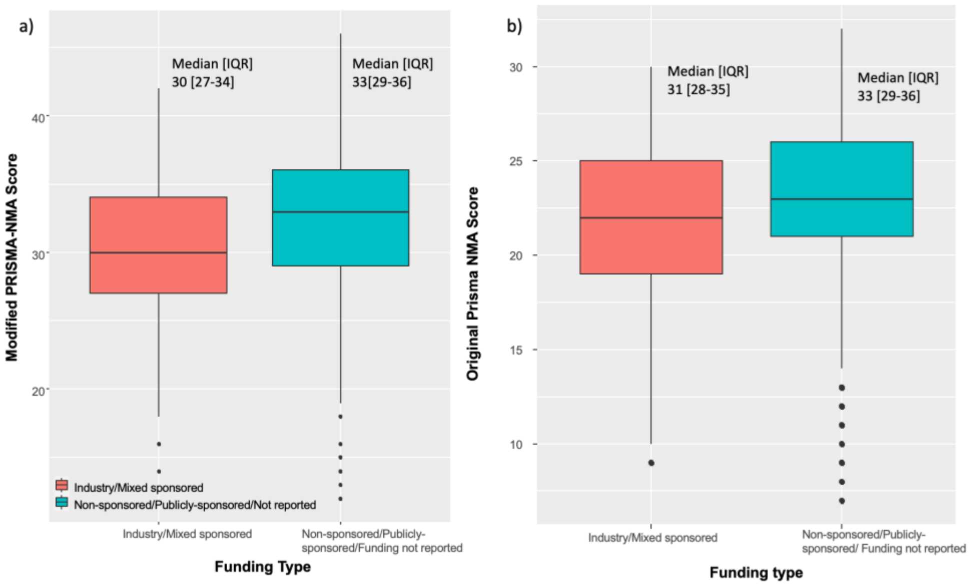

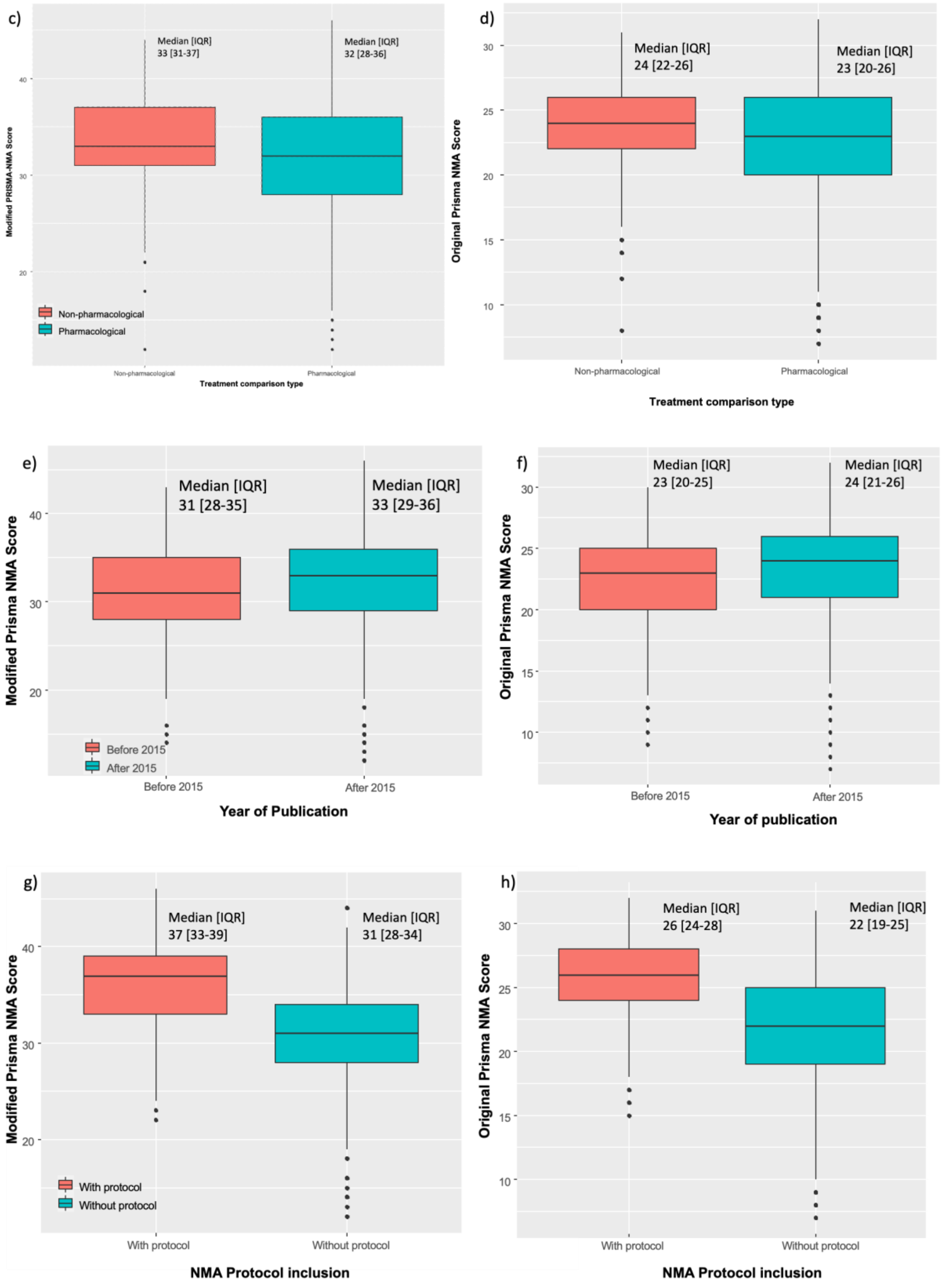
c)
Median [IQR]
33 [31-37]
Median [IQR]
32 [28-36]
Modified PRISMA-NMA Score
40
30
20
Non-pharmacological
Pharmacological
Non-pharmacological
Pharmacological
Treatment comparison type
d)
Median [IQR]
24 [22-26]
Median [IQR]
23 [20-26]
Original Prisma NMA Score
30
25
20
15
10
Non-pharmacological
Pharmacological
Treatment comparison type
e)
Median [IQR]
31 [28-35]
Median [IQR]
33 [29-36]
Modified Prisma NMA Score
40
30
20
Before 2015
After 2015
Before 2015
After 2015
Year of Publication
f)
Median [IQR]
23 [20-25]
Median [IQR]
24 [21-26]
Original Prisma NMA Score
30
25
20
15
10
Before 2015
After 2015
Year of publication
g)
Median [IQR]
37 [33-39]
Median [IQR]
31 [28-34]
Modified Prisma NMA Score
40
30
20
With protocol
Without protocol
With protocol
Without protocol
NMA Protocol inclusion
h)
Median [IQR]
26 [24-28]
Median [IQR]
22 [19-25]
Original Prisma NMA Score
30
25
20
15
10
With protocol
Without protocol
NMA Protocol inclusion

Appendix Table 5. Assessment of reporting of NMAs using the *modified* PRISMA-NMA per year of publication

Year of publication	**2013**	**2014**	**2015**	**2016**	**2017**	**2018**
**PRISMA ITEMS**	**# articles (%)**	**# articles (%)**	**# articles (%)**	**# articles (%)**	**# articles (%)**	**# articles (%)**
**TITLE**						
Systematic review	25 (27%)	29 (28%)	85 (44%)	83 (42%)	113 (36%)	99 (41%)
NMA/related form of MA	73 (80%)	84 (81%)	165 (85%)	174 (88%)	291 (92%)	222 (92%)
**ABSTRACT**						
Structured summary	83 (91%)	95 (91%)	156 (80%)	165 (83%)	247 (78%)	186 (77%)
Main objectives	89 (98%)	101 (97%)	190 (98%)	192 (97%)	299 (95%)	236 (98%)
Data sources	50 (55%)	57 (55%)	114 (59%)	120 (61%)	159 (50%)	148 (61%)
PICO	65 (71%)	93 (89%)	174 (90%)	175 (88%)	204 (65%)	215 (89%)
study appraisal (e.g., risk of bias)	12 (13%)	10 (10%)	29 (15%)	15 (8%)	31 (10%)	35 (15%)
synthesis methods (e.g. NMA)	84 (92%)	100 (96%)	182 (94%)	186 (94%)	288 (91%)	235 (98%)
Number of studies and participants	78 (86%)	90 (87%)	158 (81%)	158 (80%)	265 (84%)	214 (89%)
summary estimates and their CIs/CrIs	65 (71%)	72 (69%)	117 (60%)	122 (62%)	186 (59%)	136 (56%)
treatment ranking	22 (24%)	19 (18%)	51 (26%)	104 (53%)	115 (36%)	117 (49%)
limitations	7 (8%)	11 (11%)	32 (16%)	23 (12%)	26 (8%)	26 (11%)
conclusions and implications of findings	84 (92%)	104 (100%)	186 (96%)	192 (97%)	308 (97%)	233 (97%)
funding	9 (10%)	9 (9%)	19 (10%)	20 (10%)	9 (3%)	6 (2%)
registration number with registry name (e.g. PROSPERO)	3 (3%)	4 (4%)	12 (6%)	26 (13%)	28 (9%)	22 (9%)
**INTRODUCTION**						
Rationale for systematic review and NMA	85 (93%)	100 (96%)	192 (99%)	180 (91%)	297 (94%)	234 (97%)
Explicit statement of the questions being addressed (PICOS)	70 (77%)	94 (90%)	179 (92%)	185 (93%)	229 (72%)	238 (99%)
**METHODS**						
Existence of review protocol	15 (16%)	16 (15%)	45 (23%)	57 (29%)	68 (22%)	93 (39%)
Protocol can be accessed (e.g., Web address or registration number available)	11 (12%)	8 (8%)	30 (15%)	49 (25%)	60 (19%)	71 (29%)
rationale for eligibility criteria (e.g., PICOS, length of follow-up, years considered, language, publication status)	82 (90%)	96 (92%)	179 (92%)	187 (94%)	293 (93%)	231 (96%)
Description of eligible treatments used in the NMA (with justification for clustering, if any)	74 (81%)	82 (79%)	150 (77%)	176 (89%)	272 (86%)	199 (83%)
Description of information sources with search dates)	88 (97%)	97 (93%)	187 (96%)	191 (96%)	299 (95%)	225 (93%)
A full electronic search strategy available	36 (40%)	47 (45%)	92 (47%)	117 (59%)	150 (47%)	116 (48%)

Description of the study selection process	72 (79%)	79 (76%)	170 (88%)	180 (91%)	259 (82%)	200 (83%)
Description of the data collection process (method)	61 (67%)	68 (65%)	111 (57%)	181 (91%)	231 (73%)	183 (76%)
Description of collected items)	57 (63%)	66 (63%)	136 (70%)	174 (88%)	230 (73%)	185 (77%)
Description of methods used to explore network geometry (e.g., network plot, other methods to describe the evidence base)	12 (13%)	10 (10%)	41 (21%)	45 (23%)	76 (24%)	71 (29%)
Description of methods used to assess study risk of bias	67 (74%)	79 (76%)	152 (78%)	160 (81%)	249 (79%)	208 (86%)
Description of summary measures to be used (e.g., OR, RR, MD, SMD)	85 (93%)	98 (94%)	183 (94%)	181 (91%)	285 (90%)	212 (88%)
Description of treatment rankings to be used (e.g., SUCRA, P-scores)	33 (36%)	36 (35%)	95 (49%)	138 (70%)	222 (70%)	185 (77%)
Description of analysis methods (e.g., NMA method)	85 (93%)	99 (95%)	187 (96%)	185 (93%)	307 (97%)	235 (98%)
Description of method used to assess inconsistency	47 (52%)	54 (52%)	133 (69%)	126 (64%)	214 (68%)	188 (78%)
Description of methods used to assess bias across studies (e.g., publication bias, selective reporting, small study effects)	27 (30%)	26 (25%)	72 (37%)	59 (30%)	104 (33%)	124 (51%)
Description of additional analyses (e.g., sensitivity analysis)	51 (56%)	61 (59%)	113 (58%)	111 (56%)	123 (39%)	138 (57%)
**RESULTS**						
# of studies screened and included in the review, and reasons for exclusion (e.g., flow diagram)	86 (95%)	95 (91%)	178 (92%)	183 (92%)	288 (91%)	229 (95%)
Network plot	68 (75%)	69 (66%)	161 (83%)	168 (85%)	284 (90%)	221 (92%)
Brief overview of network characteristics	72 (79%)	73 (70%)	166 (86%)	168 (85%)	258 (82%)	221 (92%)
Presentation of characteristics per study with citations (e.g., in a table)	80 (88%)	95 (91%)	170 (88%)	185 (93%)	282 (89%)	229 (95%)
Presentation of risk of bias per study	51 (56%)	56 (54%)	134 (69%)	134 (68%)	193 (61%)	166 (69%)
Presentation of individual study data	52 (57%)	62 (60%)	101 (52%)	59 (30%)	125 (40%)	112 (46%)
Presentation of NMA results (summary estimates and CIs/CrIs, ranking statistics)	88 (97%)	99 (95%)	188 (97%)	194 (98%)	301 (95%)	238 (99%)
Description of results from investigations of inconsistency	40 (44%)	44 (42%)	111 (57%)	112 (57%)	192 (61%)	156 (65%)
Presentation of results of bias assessment across studies (e.g., funnel plot)	20 (22%)	26 (25%)	63 (32%)	55 (28%)	106 (34%)	98 (41%)
Presentation of results of additional analyses (e.g., sensitivity analysis)	44 (48%)	56 (54%)	108 (56%)	106 (54%)	126 (40%)	142 (59%)
**DISCUSSION**						
Summary of key findings, including strength of evidence	91 (100%)	104 (100%)	192 (99%)	196 (99%)	314 (99%)	238 (99%)

Discussion of study limitations	85 (93%)	93 (89%)	182 (94%)	186 (94%)	301 (95%)	233 (97%)
General interpretation of results, comparison to other evidence, and implications for future research	90 (99%)	103 (99%)	169 (87%)	182 (92%)	301 (95%)	220 (91%)
**FUNDING**						
Sources of funding for the systematic review	71 (78%)	84 (81%)	141 (73%)	139 (70%)	216 (68%)	169 (70%)
Role of funders for the systematic review	21 (23%)	23 (22%)	57 (29%)	39 (20%)	39 (12%)	38 (16%)
**Total # of articles**	**91**	**104**	**194**	**198**	**316**	**241**

Abbreviations: CI, confidence interval; CrI, credible interval NMA, network meta-analysis

Appendix Figure 8. Distribution of journal impact factor in NMAs published in 2016 and 2017

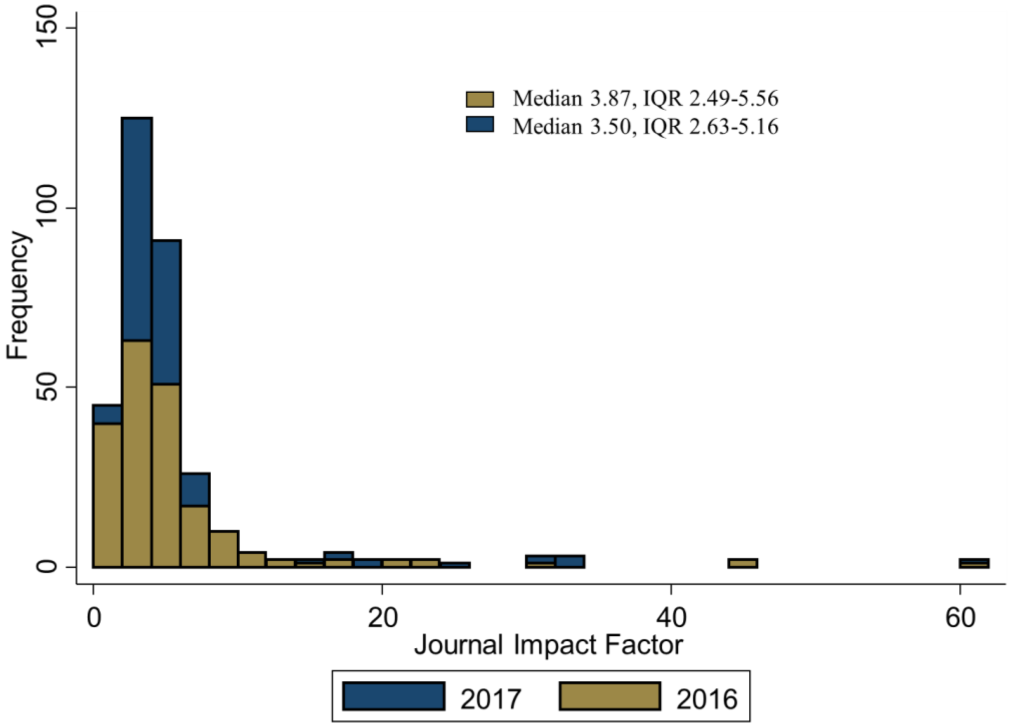

Supplement: Supplementary file 1 — Additional file 1: Appendix 1. Eligibility criteria, screening, study selection, and data abstraction. Appendix Tables. Appendix Figures. [file 13643_2021_1780_MOESM1_ESM.pdf]
