# Supplementary material for: Do reporting guidelines have an impact? Empirical assessment of changes in reporting before and after the PRISMA extension statement for network meta-analysis
Source: Syst Rev. 2021 Sep 10;10:246. doi: 10.1186/s13643-021-01780-9 (PMC8434710; doi:10.1186/s13643-021-01780-9)
Supplement: Supplementary file 2 — Additional file 2: References of included studies. [file 13643_2021_1780_MOESM2_ESM.docx]

# **Additional file 2: References of included studies**

1. Akshintala, V. S. et al. Systematic review with network meta-analysis: pharmacological prophylaxis against post-ERCP pancreatitis. Alimentary pharmacology & therapeutics 38, 1325-1337, doi:10.1111/apt.12534 (2013).
2. Alfirevic, Z. et al. Labour induction with prostaglandins: a systematic review and network meta-analysis. BMJ (Clinical research ed.) 350, h217, doi:10.1136/bmj.h217 (2015).
3. Ashby, R. L. et al. VenUS IV (Venous leg Ulcer Study IV) - compression hosiery compared with compression bandaging in the treatment of venous leg ulcers: a randomised controlled trial, mixed-treatment comparison and decision-analytic model. Health technology assessment (Winchester, England) 18, 1-293, v-vi, doi:10.3310/hta18570 (2014).
4. Assiri, A., Al-Majzoub, O., Kanaan, A. O., Donovan, J. L. & Silva, M. Mixed treatment comparison meta-analysis of aspirin, warfarin, and new anticoagulants for stroke prevention in patients with nonvalvular atrial fibrillation. Clinical therapeutics 35, 967-984 e962, doi:10.1016/j.clinthera.2013.05.011 (2013).
5. Baji, P. et al. Efficacy and safety of infliximab-biosimilar compared to other biological drugs in rheumatoid arthritis: a mixed treatment comparison. The European journal of health economics : HEPAC : health economics in prevention and care 15 Suppl 1, S53-64, doi:10.1007/s10198-014-0594-4 (2014).
6. Baji, P. et al. Comparative efficacy and safety of biosimilar infliximab and other biological treatments in ankylosing spondylitis: systematic literature review and meta-analysis. The European journal of health economics : HEPAC : health economics in prevention and care 15 Suppl 1, S45-52, doi:10.1007/s10198-014-0593-5 (2014).
7. Bangalore, S., Amoroso, N., Fusaro, M., Kumar, S. & Feit, F. Outcomes with various drug-eluting or bare metal stents in patients with ST-segment-elevation myocardial infarction: a mixed treatment comparison analysis of trial level data from 34 068 patient-years of follow-up from randomized trials. Circulation. Cardiovascular interventions 6, 378-390, doi:10.1161/CIRCINTERVENTIONS.113.000415 (2013).
8. Bangalore, S. et al. Bare metal stents, durable polymer drug eluting stents, and biodegradable polymer drug eluting stents for coronary artery disease: mixed treatment comparison meta-analysis. BMJ (Clinical research ed.) 347, f6625, doi:10.1136/bmj.f6625 (2013).
9. Bangalore, S., Toklu, B. & Feit, F. Outcomes with coronary artery bypass graft surgery versus percutaneous coronary intervention for patients with diabetes mellitus: can newer generation drug-eluting stents bridge the gap? Circulation. Cardiovascular interventions 7, 518-525, doi:10.1161/CIRCINTERVENTIONS.114.001346 (2014).
10. Bangalore, S. et al. Anticoagulant therapy during primary percutaneous coronary intervention for acute myocardial infarction: a meta-analysis of randomized trials in the era of stents and P2Y12 inhibitors. BMJ (Clinical research ed.) 349, g6419, doi:10.1136/bmj.g6419 (2014).
11. Bannuru, R. R. et al. Comparative effectiveness of pharmacologic interventions for knee osteoarthritis: a systematic review and network meta-analysis. Annals of internal medicine 162, 46-54, doi:10.7326/M14-1231 (2015).
12. Barth, J. et al. Comparative efficacy of seven psychotherapeutic interventions for patients with depression: a network meta-analysis. PLoS medicine 10, e1001454, doi:10.1371/journal.pmed.1001454 (2013).
13. Benedetto, U. et al. Searching for the second best graft for coronary artery bypass surgery: a network meta-analysis of randomized controlled trialsdagger. European journal of cardio-thoracic surgery : official journal of the European Association for Cardio-thoracic Surgery 47, 59-65; discussion 65, doi:10.1093/ejcts/ezu111 (2015).
14. Biondi-Zoccai, G. et al. Nephropathy after administration of iso-osmolar and low-osmolar contrast media: evidence from a network meta-analysis. International journal of cardiology 172, 375-380, doi:10.1016/j.ijcard.2014.01.075 (2014).
15. Biondi-Zoccai, G. et al. Comparative effectiveness of novel oral anticoagulants for atrial fibrillation: evidence from pair-wise and warfarin-controlled network meta-analyses. HSR proceedings in intensive care & cardiovascular anesthesia 5, 40-54 (2013).
16. Bodalia, P. N. et al. Comparative efficacy and tolerability of anti-epileptic drugs for refractory focal epilepsy: systematic review and network meta-analysis reveals the need for long term comparator trials. British journal of clinical pharmacology 76, 649-667, doi:10.1111/bcp.12083 (2013).
17. Bow, E. J. et al. Systematic review and mixed treatment comparison meta-analysis of randomized clinical trials of primary oral antifungal prophylaxis in allogeneic hematopoietic cell transplant recipients. BMC infectious diseases 15, 128, doi:10.1186/s12879-015-0855-6 (2015).
18. Braun, S. R., Gregor, B. & Tran, U. S. Comparing bona fide psychotherapies of depression in adults with two meta-analytical approaches. PloS one 8, e68135, doi:10.1371/journal.pone.0068135 (2013).
19. Brown, T. et al. Clinical effectiveness and cost-effectiveness of first-line chemotherapy for adult patients with locally advanced or metastatic non-small cell lung cancer: a systematic review and economic evaluation. Health technology assessment (Winchester, England) 17, 1-278, doi:10.3310/hta17310 (2013).
20. Bulluck, H., Kwok, C. S., Ryding, A. D. & Loke, Y. K. Safety of short-term dual antiplatelet therapy after drug-eluting stents: An updated meta-analysis with direct and adjusted indirect comparison of randomized control trials. International journal of cardiology 181, 331-339, doi:10.1016/j.ijcard.2014.12.037 (2015).
21. Buti, J., Baccini, M., Nieri, M., La Marca, M. & Pini-Prato, G. P. Bayesian network meta-analysis of root coverage procedures: ranking efficacy and identification of best treatment. Journal of clinical periodontology 40, 372-386, doi:10.1111/jcpe.12028 (2013).
22. Cahill, K., Stevens, S., Perera, R. & Lancaster, T. Pharmacological interventions for smoking cessation: an overview and network meta-analysis. The Cochrane database of systematic reviews, Cd009329, doi:10.1002/14651858.CD009329.pub2 (2013).
23. Caldeira, D., Pinto, F. J. & Ferreira, J. J. Dyspnea and reversibility profile of P2Y(1)(2) antagonists: systematic review of new antiplatelet drugs. American journal of cardiovascular drugs : drugs, devices, and other interventions 14, 303-311, doi:10.1007/s40256-014-0071-6 (2014).
24. Cameron, C. et al. Systematic review and network meta-analysis comparing antithrombotic agents for the prevention of stroke and major bleeding in patients with atrial fibrillation. BMJ open 4, e004301, doi:10.1136/bmjopen-2013-004301 (2014).
25. Carroll, C. et al. Clinical effectiveness and cost-effectiveness of minimally invasive techniques to manage varicose veins: a systematic review and economic evaluation. Health technology assessment (Winchester, England) 17, i-xvi, 1-141, doi:10.3310/hta17480 (2013).
26. Carter, P. et al. A Mediterranean diet improves HbA1c but not fasting blood glucose compared to alternative dietary strategies: a network meta-analysis. Journal of human nutrition and dietetics : the official journal of the British Dietetic Association 27, 280-297, doi:10.1111/jhn.12138 (2014).
27. Caruba, T. et al. Treatment for stable coronary artery disease: a network meta-analysis of cost-effectiveness studies. PloS one 9, e98371, doi:10.1371/journal.pone.0098371 (2014).
28. Castellucci, L. A. et al. Efficacy and safety outcomes of oral anticoagulants and antiplatelet drugs in the secondary prevention of venous thromboembolism: systematic review and network meta-analysis. BMJ (Clinical research ed.) 347, f5133, doi:10.1136/bmj.f5133 (2013).
29. Castellucci, L. A. et al. Clinical and safety outcomes associated with treatment of acute venous thromboembolism: a systematic review and meta-analysis. Jama 312, 1122-1135, doi:10.1001/jama.2014.10538 (2014).
30. Cawston, H., Davie, A., Paget, M. A., Skljarevski, V. & Happich, M. Efficacy of duloxetine versus alternative oral therapies: an indirect comparison of randomised clinical trials in chronic low back pain. European spine journal : official publication of the European Spine Society, the European Spinal Deformity Society, and the European Section of the Cervical Spine Research Society 22, 1996-2009, doi:10.1007/s00586-013-2804-7 (2013).
31. Chambers, J. D., Winn, A., Zhong, Y., Olchanski, N. & Cangelosi, M. J. Potential role of network meta-analysis in value-based insurance design. The American journal of managed care 20, 641-648 (2014).
32. Chan, K. et al. A Bayesian meta-analysis of multiple treatment comparisons of systemic regimens for advanced pancreatic cancer. PloS one 9, e108749, doi:10.1371/journal.pone.0108749 (2014).
33. Chatterjee, S. et al. Benefits of beta blockers in patients with heart failure and reduced ejection fraction: network meta-analysis. BMJ (Clinical research ed.) 346, f55, doi:10.1136/bmj.f55 (2013).
34. Chatterjee, S., Sardar, P., Biondi-Zoccai, G. & Kumbhani, D. J. New oral anticoagulants and the risk of intracranial hemorrhage: traditional and Bayesian meta-analysis and mixed treatment comparison of randomized trials of new oral anticoagulants in atrial fibrillation. JAMA neurology 70, 1486-1490, doi:10.1001/jamaneurol.2013.4021 (2013).
35. Chen, L. et al. Phosphodiesterase 5 inhibitors for the treatment of erectile dysfunction: a trade-off network meta-analysis. European urology 68, 674-680, doi:10.1016/j.eururo.2015.03.031 (2015).
36. Chen, Y., Zhang, Y., Tang, Y., Huang, X. & Xie, Y. Long-term clinical efficacy and safety of adding cilostazol to dual antiplatelet therapy for patients undergoing PCI: a meta-analysis of randomized trials with adjusted indirect comparisons. Current medical research and opinion 30, 37-49, doi:10.1185/03007995.2013.850067 (2014).
37. Chilton, M. et al. The effect of antiobesity drugs on waist circumference: a mixed treatment comparison. Diabetes, obesity & metabolism 16, 237-247, doi:10.1111/dom.12198 (2014).
38. Cope, S. et al. Comparative efficacy of long-acting bronchodilators for COPD: a network meta-analysis. Respiratory research 14, 100, doi:10.1186/1465-9921-14-100 (2013).
39. Cope, S., Ouwens, M. J., Jansen, J. P. & Schmid, P. Progression-free survival with fulvestrant 500 mg and alternative endocrine therapies as second-line treatment for advanced breast cancer: a network meta-analysis with parametric survival models. Value in health : the journal of the International Society for Pharmacoeconomics and Outcomes Research 16, 403-417, doi:10.1016/j.jval.2012.10.019 (2013).
40. Corbett, M. S. et al. Acupuncture and other physical treatments for the relief of pain due to osteoarthritis of the knee: network meta-analysis. Osteoarthritis and cartilage 21, 1290-1298, doi:10.1016/j.joca.2013.05.007 (2013).
41. Craddy, P., Palin, H. J. & Johnson, K. I. Comparative effectiveness of dipeptidylpeptidase-4 inhibitors in type 2 diabetes: a systematic review and mixed treatment comparison. Diabetes therapy : research, treatment and education of diabetes and related disorders 5, 1-41, doi:10.1007/s13300-014-0061-3 (2014).
42. Cucherat, M., Stalmans, I. & Rouland, J. F. Relative efficacy and safety of preservative-free latanoprost (T2345) for the treatment of open-angle glaucoma and ocular hypertension: an adjusted Indirect comparison meta-analysis of randomized clinical trials. Journal of glaucoma 23, e69-75, doi:10.1097/IJG.0b013e3182a075e6 (2014).
43. Cui, J., Wu, B., Liu, C. & Li, Z. A systematic review and adjusted indirect comparison of oral anticoagulants. Orthopedics 37, 763-771, doi:10.3928/01477447-20141023-07 (2014).
44. Dai, N. et al. Different β-blockers and initiation time in patients undergoing noncardiac surgery: a meta-analysis. The American journal of the medical sciences 347, 235-244, doi:10.1097/MAJ.0b013e31828c607c (2014).
45. Dai, X., Wang, H., Jing, Z. & Fu, P. The effect of a dual combination of noninsulin antidiabetic drugs on lipids: a systematic review and network meta-analysis. Current medical research and opinion 30, 1777-1786, doi:10.1185/03007995.2014.921608 (2014).
46. Dal Molin, A. et al. Flushing the central venous catheter: is heparin necessary? The journal of vascular access 15, 241-248, doi:10.5301/jva.5000225 (2014).
47. Datto, C., Hellmund, R. & Siddiqui, M. K. Efficacy and tolerability of naproxen/esomeprazole magnesium tablets compared with non-specific NSAIDs and COX-2 inhibitors: a systematic review and network analyses. Open access rheumatology : research and reviews 5, 1-19, doi:10.2147/oarrr.s41420 (2013).
48. Desamericq, G. et al. Long-term neurocognitive effects of antipsychotics in schizophrenia: a network meta-analysis. European journal of clinical pharmacology 70, 127-134, doi:10.1007/s00228-013-1600-y (2014).
49. Dogliotti, A., Paolasso, E. & Giugliano, R. P. Current and new oral antithrombotics in non-valvular atrial fibrillation: a network meta-analysis of 79 808 patients. Heart (British Cardiac Society) 100, 396-405, doi:10.1136/heartjnl-2013-304347 (2014).
50. Dong, J., Gao, L., Lu, W., Xu, Z. & Zheng, J. Pharmacological interventions for acceleration of the onset time of rocuronium: a meta-analysis. PloS one 9, e114231, doi:10.1371/journal.pone.0114231 (2014).
51. Dong, W. et al. Treatments for shoulder impingement syndrome: a PRISMA systematic review and network meta-analysis. Medicine 94, e510, doi:10.1097/MD.0000000000000510 (2015).
52. Dong, Y. H. et al. Comparative safety of inhaled medications in patients with chronic obstructive pulmonary disease: systematic review and mixed treatment comparison meta-analysis of randomised controlled trials. Thorax 68, 48-56, doi:10.1136/thoraxjnl-2012-201926 (2013).
53. Dooley, C., Kaur, R. & Sobieraj, D. M. Comparison of the efficacy and safety of low molecular weight heparins for venous thromboembolism prophylaxis in medically ill patients. Current medical research and opinion 30, 367-380, doi:10.1185/03007995.2013.837818 (2014).
54. Dranitsaris, G. & Ellis, A. K. Sublingual or subcutaneous immunotherapy for seasonal allergic rhinitis: an indirect analysis of efficacy, safety and cost. Journal of evaluation in clinical practice 20, 225-238, doi:10.1111/jep.12112 (2014).
55. Dumville, J. C., McFarlane, E., Edwards, P., Lipp, A. & Holmes, A. Preoperative skin antiseptics for preventing surgical wound infections after clean surgery. The Cochrane database of systematic reviews, Cd003949, doi:10.1002/14651858.CD003949.pub3 (2013).
56. Edwards, S. J., Barton, S., Thurgar, E. & Trevor, N. Topotecan, pegylated liposomal doxorubicin hydrochloride, paclitaxel, trabectedin and gemcitabine for advanced recurrent or refractory ovarian cancer: a systematic review and economic evaluation. Health technology assessment (Winchester, England) 19, 1-480, doi:10.3310/hta19070 (2015).
57. Ellis, A. G. et al. Indirect comparison of bazedoxifene vs oral bisphosphonates for the prevention of vertebral fractures in postmenopausal osteoporotic women. Current medical research and opinion 30, 1617-1626, doi:10.1185/03007995.2014.908279 (2014).
58. Ellis, A. G. et al. Bazedoxifene versus oral bisphosphonates for the prevention of nonvertebral fractures in postmenopausal women with osteoporosis at higher risk of fracture: a network meta-analysis. Value in health : the journal of the International Society for Pharmacoeconomics and Outcomes Research 17, 424-432, doi:10.1016/j.jval.2014.01.008 (2014).
59. Faggion, C. M., Jr., Listl, S., Fruhauf, N., Chang, H. J. & Tu, Y. K. A systematic review and Bayesian network meta-analysis of randomized clinical trials on non-surgical treatments for peri-implantitis. Journal of clinical periodontology 41, 1015-1025, doi:10.1111/jcpe.12292 (2014).
60. Fang, Y. et al. Radioiodine therapy for patients with differentiated thyroid cancer after thyroidectomy: direct comparison and network meta-analyses. Journal of endocrinological investigation 36, 896-902, doi:10.3275/8998 (2013).
61. Filippini, G. et al. Immunomodulators and immunosuppressants for multiple sclerosis: a network meta-analysis. The Cochrane database of systematic reviews, Cd008933, doi:10.1002/14651858.CD008933.pub2 (2013).
62. Fournier, M., Germe, M., Theobald, K., Scholz, G. H. & Lehmacher, W. Indirect comparison of lixisenatide versus neutral protamine Hagedorn insulin as add-on to metformin and sulphonylurea in patients with type 2 diabetes mellitus. German medical science : GMS e-journal 12, Doc14, doi:10.3205/000199 (2014).
63. Furukawa, T. A. et al. Waiting list may be a nocebo condition in psychotherapy trials: a contribution from network meta-analysis. Acta psychiatrica Scandinavica 130, 181-192, doi:10.1111/acps.12275 (2014).
64. Gagne, J. J. et al. Effect of smoking on comparative efficacy of antiplatelet agents: systematic review, meta-analysis, and indirect comparison. BMJ (Clinical research ed.) 347, f5307, doi:10.1136/bmj.f5307 (2013).
65. Galvan-Banqueri, M., Marin Gil, R., Santos Ramos, B. & Bautista Paloma, F. J. Biological treatments for moderate-to-severe psoriasis: indirect comparison. Journal of clinical pharmacy and therapeutics 38, 121-130, doi:10.1111/jcpt.12044 (2013).
66. Galvan-Banqueri, M., Vega-Coca, M. D., Castillo-Munoz, M. A., Beltran Calvo, C. & Molina Lopez, T. Indirect comparison for Anti-TNF drugs in moderate to severe ulcerative colitis. Farmacia hospitalaria : organo oficial de expresion cientifica de la Sociedad Espanola de Farmacia Hospitalaria 39, 80-91, doi:10.7399/fh.2015.39.2.8218 (2015).
67. Gao, L., Xia, L., Zhao, F. L. & Li, S. C. Clinical efficacy and safety of the newer antiepileptic drugs as adjunctive treatment in adults with refractory partial-onset epilepsy: a meta-analysis of randomized placebo-controlled trials. Epilepsy research 103, 31-44, doi:10.1016/j.eplepsyres.2012.06.005 (2013).
68. Gerger, H. et al. Integrating fragmented evidence by network meta-analysis: relative effectiveness of psychological interventions for adults with post-traumatic stress disorder. Psychological medicine 44, 3151-3164, doi:10.1017/S0033291714000853 (2014).
69. Gonzalez-Vacarezza, N., Aleman, A., Gonzalez, G. & Perez, A. Rituximab and tocilizumab for the treatment of rheumatoid arthritis. International journal of technology assessment in health care 30, 282-288, doi:10.1017/S0266462314000221 (2014).
70. Goralczyk, A. D., Cameron, S. & Amanzada, A. Treatment of chronic HCV genotype 1 infection with telaprevir: a Bayesian mixed treatment comparison of fixed-length and response-guided treatment regimens in treatment-naïve and -experienced patients. BMC gastroenterology 13, 148, doi:10.1186/1471-230x-13-148 (2013).
71. Goring, S. et al. Dapagliflozin compared with other oral anti-diabetes treatments when added to metformin monotherapy: a systematic review and network meta-analysis. Diabetes, obesity & metabolism 16, 433-442, doi:10.1111/dom.12239 (2014).
72. Graudal, N., Hubeck-Graudal, T., Tarp, S., Christensen, R. & Jurgens, G. Effect of combination therapy on joint destruction in rheumatoid arthritis: a network meta-analysis of randomized controlled trials. PloS one 9, e106408, doi:10.1371/journal.pone.0106408 (2014).
73. Greco, T. et al. A Bayesian network meta-analysis on the effect of inodilatory agents on mortality. British journal of anaesthesia 114, 746-756, doi:10.1093/bja/aeu446 (2015).
74. Gresham, G. K., Wells, G. A., Gill, S., Cameron, C. & Jonker, D. J. Chemotherapy regimens for advanced pancreatic cancer: a systematic review and network meta-analysis. BMC cancer 14, 471, doi:10.1186/1471-2407-14-471 (2014).
75. Griebeler, M. L. et al. Pharmacologic interventions for painful diabetic neuropathy: An umbrella systematic review and comparative effectiveness network meta-analysis. Annals of internal medicine 161, 639-649, doi:10.7326/M14-0511 (2014).
76. Gupta, A. K. & Charrette, A. The efficacy and safety of 5alpha-reductase inhibitors in androgenetic alopecia: a network meta-analysis and benefit-risk assessment of finasteride and dutasteride. The Journal of dermatological treatment 25, 156-161, doi:10.3109/09546634.2013.813011 (2014).
77. Gupta, A. K., Daigle, D. & Lyons, D. C. Network Meta-analysis of Treatments for Chronic Plaque Psoriasis in Canada. Journal of cutaneous medicine and surgery 18, 371-378, doi:10.2310/7750.2014.13191 (2014).
78. Gupta, A. K. & Paquet, M. Network meta-analysis of the outcome 'participant complete clearance' in nonimmunosuppressed participants of eight interventions for actinic keratosis: a follow-up on a Cochrane review. The British journal of dermatology 169, 250-259, doi:10.1111/bjd.12343 (2013).
79. Hadjigeorgiou, G. M. et al. A network meta-analysis of randomized controlled trials for comparing the effectiveness and safety profile of treatments with marketing authorization for relapsing multiple sclerosis. Journal of clinical pharmacy and therapeutics 38, 433-439, doi:10.1111/jcpt.12090 (2013).
80. Haspinger, E. R. et al. Is there evidence for different effects among EGFR-TKIs? Systematic review and meta-analysis of EGFR tyrosine kinase inhibitors (TKIs) versus chemotherapy as first-line treatment for patients harboring EGFR mutations. Critical reviews in oncology/hematology 94, 213-227, doi:10.1016/j.critrevonc.2014.11.005 (2015).
81. Hazlewood, G. S. et al. Comparative effectiveness of immunosuppressants and biologics for inducing and maintaining remission in Crohn's disease: a network meta-analysis. Gastroenterology 148, 344-354 e345; quiz e314-345, doi:10.1053/j.gastro.2014.10.011 (2015).
82. Hoaglin, D. C., Filonenko, A., Glickman, M. E., Wasiak, R. & Gidwani, R. Use of mixed-treatment-comparison methods in estimating efficacy of treatments for heavy menstrual bleeding. European journal of medical research 18, 17, doi:10.1186/2047-783x-18-17 (2013).
83. Howell, N., Senanayake, E., Freemantle, N. & Pagano, D. Putting the record straight on aprotinin as safe and effective: results from a mixed treatment meta-analysis of trials of aprotinin. The Journal of thoracic and cardiovascular surgery 145, 234-240, doi:10.1016/j.jtcvs.2012.07.018 (2013).
84. Huai, Z. Y., Feng Xian, W., Chang Jiang, L. & Xi Chen, W. Submucosal injection solution for endoscopic resection in gastrointestinal tract: a traditional and network meta-analysis. Gastroenterology research and practice 2015, 702768, doi:10.1155/2015/702768 (2015).
85. Hutchinson, M. et al. Efficacy and safety of BG-12 (dimethyl fumarate) and other disease-modifying therapies for the treatment of relapsing-remitting multiple sclerosis: a systematic review and mixed treatment comparison. Current medical research and opinion 30, 613-627, doi:10.1185/03007995.2013.863755 (2014).
86. Huttner, F. J. et al. Meta-analysis of reconstruction techniques after low anterior resection for rectal cancer. The British journal of surgery 102, 735-745, doi:10.1002/bjs.9782 (2015).
87. Jansen, J. P., Buckley, F., Dejonckheere, F. & Ogale, S. Comparative efficacy of biologics as monotherapy and in combination with methotrexate on patient reported outcomes (PROs) in rheumatoid arthritis patients with an inadequate response to conventional DMARDs--a systematic review and network meta-analysis. Health and quality of life outcomes 12, 102, doi:10.1186/1477-7525-12-102 (2014).
88. Johnston, B. C. et al. Comparison of weight loss among named diet programs in overweight and obese adults: a meta-analysis. Jama 312, 923-933, doi:10.1001/jama.2014.10397 (2014).
89. Jonas, D. E. et al. in Psychological and Pharmacological Treatments for Adults With Posttraumatic Stress Disorder (PTSD) (Agency for Healthcare Research and Quality (US), 2013).
90. Kang, N. & Sobieraj, D. M. Indirect treatment comparison of new oral anticoagulants for the treatment of acute venous thromboembolism. Thrombosis research 133, 1145-1151, doi:10.1016/j.thromres.2014.03.035 (2014).
91. Karabis, A., Lindner, L., Mocarski, M., Huisman, E. & Greening, A. Comparative efficacy of aclidinium versus glycopyrronium and tiotropium, as maintenance treatment of moderate to severe COPD patients: a systematic review and network meta-analysis. International journal of chronic obstructive pulmonary disease 8, 405-423, doi:10.2147/COPD.S48967 (2013).
92. Katsanos, K. et al. Bayesian network meta-analysis of nitinol stents, covered stents, drug-eluting stents, and drug-coated balloons in the femoropopliteal artery. Journal of vascular surgery 59, 1123-1133 e1128, doi:10.1016/j.jvs.2014.01.041 (2014).
93. Kew, K. M., Dias, S. & Cates, C. J. Long-acting inhaled therapy (beta-agonists, anticholinergics and steroids) for COPD: a network meta-analysis. The Cochrane database of systematic reviews, Cd010844, doi:10.1002/14651858.CD010844.pub2 (2014).
94. Khan, N., Shah, D., Tongbram, V., Verdian, L. & Hawkins, N. The efficacy and tolerability of perampanel and other recently approved anti-epileptic drugs for the treatment of refractory partial onset seizure: a systematic review and Bayesian network meta-analysis. Current medical research and opinion 29, 1001-1013, doi:10.1185/03007995.2013.803461 (2013).
95. Kotb, A., Cameron, C., Hsieh, S. & Wells, G. Comparative effectiveness of different forms of telemedicine for individuals with heart failure (HF): a systematic review and network meta-analysis. PloS one 10, e0118681, doi:10.1371/journal.pone.0118681 (2015).
96. Kriston, L., von Wolff, A., Westphal, A., Holzel, L. P. & Harter, M. Efficacy and acceptability of acute treatments for persistent depressive disorder: a network meta-analysis. Depression and anxiety 31, 621-630, doi:10.1002/da.22236 (2014).
97. Kunitomi, T., Hashiguchi, M. & Mochizuki, M. Indirect comparison analysis of efficacy and safety between olanzapine and aripiprazole for schizophrenia. British journal of clinical pharmacology 77, 767-776, doi:10.1111/bcp.12221 (2014).
98. Kunitomi, T., Hashiguchi, M. & Mochizuki, M. Effect of common comparators in indirect comparison analysis of the effectiveness of different inhaled corticosteroids in the treatment of asthma. PloS one 10, e0120836, doi:10.1371/journal.pone.0120836 (2015).
99. Kwak, H. J. et al. Pharmacological prevention of rocuronium-induced injection pain or withdrawal movements: a meta-analysis. Journal of anesthesia 27, 742-749, doi:10.1007/s00540-013-1595-7 (2013).
100. Kwok, C. S., Pradhan, S., Yeong, J. K. & Loke, Y. K. Relative effects of two different enoxaparin regimens as comparators against newer oral anticoagulants: meta-analysis and adjusted indirect comparison. Chest 144, 593-600, doi:10.1378/chest.12-2634 (2013).
101. Landoni, G. et al. Anaesthetic drugs and survival: a Bayesian network meta-analysis of randomized trials in cardiac surgery. British journal of anaesthesia 111, 886-896, doi:10.1093/bja/aet231 (2013).
102. Laporte, S. et al. Indirect comparison meta-analysis of two enoxaparin regimens in patients undergoing major orthopaedic surgery. Impact on the interpretation of thromboprophylactic effects of new anticoagulant drugs. Thrombosis and haemostasis 112, 503-510, doi:10.1160/TH14-01-0064 (2014).
103. Le Cleach, L. et al. Oral antiviral therapy for prevention of genital herpes outbreaks in immunocompetent and nonpregnant patients. The Cochrane database of systematic reviews, Cd009036, doi:10.1002/14651858.CD009036.pub2 (2014).
104. Leaviss, J. et al. What is the clinical effectiveness and cost-effectiveness of cytisine compared with varenicline for smoking cessation? A systematic review and economic evaluation. Health technology assessment (Winchester, England) 18, 1-120, doi:10.3310/hta18330 (2014).
105. Lee, S. W., Chaiyakunapruk, N., Chong, H. Y. & Liong, M. L. Comparative effectiveness and safety of various treatment procedures for lower pole renal calculi: a systematic review and network meta-analysis. BJU international 116, 252-264, doi:10.1111/bju.12983 (2015).
106. Leibovici‐Weissman, Y. et al. Antimicrobial drugs for treating cholera. Cochrane Database of Systematic Reviews, doi:10.1002/14651858.CD008625.pub2 (2014).
107. Leucht, S. et al. Comparative efficacy and tolerability of 15 antipsychotic drugs in schizophrenia: a multiple-treatments meta-analysis. The Lancet 382, 951-962, doi:10.1016/s0140-6736(13)60733-3 (2013).
108. Levi Marpillat, N., Macquin-Mavier, I., Tropeano, A. I., Bachoud-Levi, A. C. & Maison, P. Antihypertensive classes, cognitive decline and incidence of dementia: a network meta-analysis. Journal of hypertension 31, 1073-1082, doi:10.1097/HJH.0b013e3283603f53 (2013).
109. Lewis, R. A. et al. Comparative clinical effectiveness of management strategies for sciatica: systematic review and network meta-analyses. The spine journal : official journal of the North American Spine Society 15, 1461-1477, doi:10.1016/j.spinee.2013.08.049 (2015).
110. Li, L. et al. The efficacy and safety of different kinds of laparoscopic cholecystectomy: a network meta analysis of 43 randomized controlled trials. PloS one 9, e90313, doi:10.1371/journal.pone.0090313 (2014).
111. Liang, W. et al. Network meta-analysis of erlotinib, gefitinib, afatinib and icotinib in patients with advanced non-small-cell lung cancer harboring EGFR mutations. PloS one 9, e85245, doi:10.1371/journal.pone.0085245 (2014).
112. Liao, W.-C. et al. Adjuvant treatments for resected pancreatic adenocarcinoma: a systematic review and network meta-analysis. The Lancet Oncology 14, 1095-1103, doi:10.1016/s1470-2045(13)70388-7 (2013).
113. Lin, L. et al. Comparative efficacy and tolerability of topical prostaglandin analogues for primary open-angle glaucoma and ocular hypertension. The Annals of pharmacotherapy 48, 1585-1593, doi:10.1177/1060028014548569 (2014).
114. Lin, P. Y., Chen, H. S., Wang, Y. H. & Tu, Y. K. Primary molar pulpotomy: a systematic review and network meta-analysis. Journal of dentistry 42, 1060-1077, doi:10.1016/j.jdent.2014.02.001 (2014).
115. Linde, K. et al. Efficacy and acceptability of pharmacological treatments for depressive disorders in primary care: systematic review and network meta-analysis. Annals of family medicine 13, 69-79, doi:10.1370/afm.1687 (2015).
116. Liu, J. et al. Comparative efficacy and acceptability of antidepressants in Parkinson's disease: a network meta-analysis. PloS one 8, e76651, doi:10.1371/journal.pone.0076651 (2013).
117. Liu, Y. et al. Aliskiren/amlodipine vs. aliskiren/hydrochlorothiazide in hypertension: indirect meta-analysis of trials comparing the two combinations vs. monotherapy. American journal of hypertension 27, 268-278, doi:10.1093/ajh/hpt210 (2014).
118. Llorca, P. M. et al. Relative efficacy and tolerability of vortioxetine versus selected antidepressants by indirect comparisons of similar clinical studies. Current medical research and opinion 30, 2589-2606, doi:10.1185/03007995.2014.969566 (2014).
119. Loke, Y. K., Pradhan, S., Yeong, J. K. & Kwok, C. S. Comparative coronary risks of apixaban, rivaroxaban and dabigatran: a meta-analysis and adjusted indirect comparison. British journal of clinical pharmacology 78, 707-717, doi:10.1111/bcp.12376 (2014).
120. Loveman, E. et al. The clinical effectiveness and cost-effectiveness of treatments for idiopathic pulmonary fibrosis: a systematic review and economic evaluation. Health technology assessment (Winchester, England) 19, i-xxiv, 1-336, doi:10.3310/hta19200 (2015).
121. Loveman, E. et al. The effectiveness and cost-effectiveness of treatments for idiopathic pulmonary fibrosis: systematic review, network meta-analysis and health economic evaluation. BMC pharmacology & toxicology 15, 63, doi:10.1186/2050-6511-15-63 (2014).
122. Loymans, R. J. et al. Comparative effectiveness of long term drug treatment strategies to prevent asthma exacerbations: network meta-analysis. BMJ (Clinical research ed.) 348, g3009, doi:10.1136/bmj.g3009 (2014).
123. Malloy, R. J., Kanaan, A. O., Silva, M. A. & Donovan, J. L. Evaluation of antiplatelet agents for secondary prevention of stroke using mixed treatment comparison meta-analysis. Clinical therapeutics 35, 1490-1500 e1497, doi:10.1016/j.clinthera.2013.09.004 (2013).
124. Maman, K. et al. Comparative efficacy and safety of medical treatments for the management of overactive bladder: a systematic literature review and mixed treatment comparison. European urology 65, 755-765, doi:10.1016/j.eururo.2013.11.010 (2014).
125. Mantha, S. & Ansell, J. Indirect comparison of dabigatran, rivaroxaban, apixaban and edoxaban for the treatment of acute venous thromboembolism. Journal of thrombosis and thrombolysis 39, 155-165, doi:10.1007/s11239-014-1102-5 (2015).
126. Mavranezouli, I., Meader, N., Cape, J. & Kendall, T. The cost effectiveness of pharmacological treatments for generalized anxiety disorder. PharmacoEconomics 31, 317-333, doi:10.1007/s40273-013-0031-z (2013).
127. Maxwell, L. J. et al. TNF-alpha inhibitors for ankylosing spondylitis. The Cochrane database of systematic reviews, Cd005468, doi:10.1002/14651858.CD005468.pub2 (2015).
128. Mealing, S. et al. The relative efficacy of imatinib, dasatinib and nilotinib for newly diagnosed chronic myeloid leukemia: a systematic review and network meta-analysis. Experimental hematology & oncology 2, 5, doi:10.1186/2162-3619-2-5 (2013).
129. Mealing, S. et al. The importance of baseline viral load when assessing relative efficacy in treatment-naïve HBeAg-positive chronic hepatitis B: a systematic review and network meta-analysis. Systematic reviews 3, 21, doi:10.1186/2046-4053-3-21 (2014).
130. Meissner, K. et al. Differential effectiveness of placebo treatments: a systematic review of migraine prophylaxis. JAMA internal medicine 173, 1941-1951, doi:10.1001/jamainternmed.2013.10391 (2013).
131. Messori, A., Fadda, V., Maratea, D. & Trippoli, S. First-line treatments for chronic lymphocytic leukaemia: interpreting efficacy data by network meta-analysis. Annals of hematology 94, 1003-1009, doi:10.1007/s00277-015-2310-6 (2015).
132. Messori, A. et al. Biological drugs for the treatment of moderate-to-severe psoriasis by subcutaneous route: determining statistical equivalence according to evidence-based methods. Clinical drug investigation 34, 593-598, doi:10.1007/s40261-014-0214-1 (2014).
133. Messori, A. et al. Biological drugs for the treatment of rheumatoid arthritis by the subcutaneous route: interpreting efficacy data to assess statistical equivalence. Therapeutic advances in musculoskeletal disease 6, 207-216, doi:10.1177/1759720x14554792 (2014).
134. Messori, A., Fadda, V., Maratea, D., Trippoli, S. & Marinai, C. Anti-reabsorptive agents in women with osteoporosis: determining statistical equivalence according to evidence-based methods. Journal of endocrinological investigation 37, 769-773, doi:10.1007/s40618-014-0124-3 (2014).
135. Migliore, A., Broccoli, S., Massafra, U., Cassol, M. & Frediani, B. Ranking antireabsorptive agents to prevent vertebral fractures in postmenopausal osteoporosis by mixed treatment comparison meta-analysis. European review for medical and pharmacological sciences 17, 658-667 (2013).
136. Mills, E. J. et al. Interventions to promote adherence to antiretroviral therapy in Africa: a network meta-analysis. The Lancet HIV 1, e104-e111, doi:https://doi.org/10.1016/S2352-3018(14)00003-4 (2014).
137. Mills, E. J., Thorlund, K., Eapen, S., Wu, P. & Prochaska, J. J. Cardiovascular events associated with smoking cessation pharmacotherapies: a network meta-analysis. Circulation 129, 28-41, doi:10.1161/CIRCULATIONAHA.113.003961 (2014).
138. Moja, L., Danese, S., Fiorino, G., Del Giovane, C. & Bonovas, S. Systematic review with network meta-analysis: comparative efficacy and safety of budesonide and mesalazine (mesalamine) for Crohn's disease. Alimentary pharmacology & therapeutics 41, 1055-1065, doi:10.1111/apt.13190 (2015).
139. Musini, V. M., Nazer, M., Bassett, K. & Wright, J. M. Blood pressure-lowering efficacy of monotherapy with thiazide diuretics for primary hypertension. The Cochrane database of systematic reviews, Cd003824, doi:10.1002/14651858.CD003824.pub2 (2014).
140. Myers, J. et al. The efficacy of duloxetine, non-steroidal anti-inflammatory drugs, and opioids in osteoarthritis: a systematic literature review and meta-analysis. BMC musculoskeletal disorders 15, 76, doi:10.1186/1471-2474-15-76 (2014).
141. Naci, H., Brugts, J. & Ades, T. Comparative tolerability and harms of individual statins: a study-level network meta-analysis of 246 955 participants from 135 randomized, controlled trials. Circulation. Cardiovascular quality and outcomes 6, 390-399, doi:10.1161/CIRCOUTCOMES.111.000071 (2013).
142. Naci, H., Brugts, J. J., Fleurence, R. & Ades, A. E. Comparative effects of statins on major cerebrovascular events: a multiple-treatments meta-analysis of placebo-controlled and active-comparator trials. QJM : monthly journal of the Association of Physicians 106, 299-306, doi:10.1093/qjmed/hct041 (2013).
143. Naci, H., Brugts, J. J., Fleurence, R. & Ades, A. E. Dose-comparative effects of different statins on serum lipid levels: a network meta-analysis of 256,827 individuals in 181 randomized controlled trials. European journal of preventive cardiology 20, 658-670, doi:10.1177/2047487313483600 (2013).
144. Naci, H. et al. Comparative benefits of statins in the primary and secondary prevention of major coronary events and all-cause mortality: a network meta-analysis of placebo-controlled and active-comparator trials. European journal of preventive cardiology 20, 641-657, doi:10.1177/2047487313480435 (2013).
145. Naci, H., Dias, S. & Ades, A. E. Industry sponsorship bias in research findings: a network meta-analysis of LDL cholesterol reduction in randomised trials of statins. BMJ (Clinical research ed.) 349, g5741, doi:10.1136/bmj.g5741 (2014).
146. Naci, H. & Ioannidis, J. P. Comparative effectiveness of exercise and drug interventions on mortality outcomes: metaepidemiological study. BMJ (Clinical research ed.) 347, f5577, doi:10.1136/bmj.f5577 (2013).
147. Nagayama, A. et al. Comparative effectiveness of neoadjuvant therapy for HER2-positive breast cancer: a network meta-analysis. Journal of the National Cancer Institute 106, doi:10.1093/jnci/dju203 (2014).
148. Naudet, F. et al. Which placebo to cure depression? A thought-provoking network meta-analysis. BMC medicine 11, 230, doi:10.1186/1741-7015-11-230 (2013).
149. Navarese, E. P. et al. Meta-analysis of impact of different types and doses of statins on new-onset diabetes mellitus. The American journal of cardiology 111, 1123-1130, doi:10.1016/j.amjcard.2012.12.037 (2013).
150. Navarese, E. P. et al. Safety and efficacy outcomes of first and second generation durable polymer drug eluting stents and biodegradable polymer biolimus eluting stents in clinical practice: comprehensive network meta-analysis. BMJ (Clinical research ed.) 347, f6530, doi:10.1136/bmj.f6530 (2013).
151. Nelson, H., Cartier, S., Allen-Ramey, F., Lawton, S. & Calderon, M. A. Network meta-analysis shows commercialized subcutaneous and sublingual grass products have comparable efficacy. The journal of allergy and clinical immunology. In practice 3, 256-266 e253, doi:10.1016/j.jaip.2014.09.018 (2015).
152. Oba, Y. & Lone, N. A. Comparative efficacy of inhaled corticosteroid and long-acting beta agonist combinations in preventing COPD exacerbations: a Bayesian network meta-analysis. International journal of chronic obstructive pulmonary disease 9, 469-479, doi:10.2147/COPD.S48492 (2014).
153. Oba, Y. & Lone, N. A. Mortality benefit of vasopressor and inotropic agents in septic shock: a Bayesian network meta-analysis of randomized controlled trials. Journal of critical care 29, 706-710, doi:10.1016/j.jcrc.2014.04.011 (2014).
154. Oba, Y. & Lone, N. A. Comparative efficacy of long-acting muscarinic antagonists in preventing COPD exacerbations: a network meta-analysis and meta-regression. Therapeutic advances in respiratory disease 9, 3-15, doi:10.1177/1753465814565624 (2015).
155. Oh, G. H., Yu, J. C., Choi, K. S., Joo, E. J. & Jeong, S. H. Simultaneous Comparison of Efficacy and Tolerability of Second-Generation Antipsychotics in Schizophrenia: Mixed-Treatment Comparison Analysis Based on Head-to-Head Trial Data. Psychiatry investigation 12, 46-54, doi:10.4306/pi.2015.12.1.46 (2015).
156. Orme, M. et al. A systematic review and mixed-treatment comparison of dapagliflozin with existing anti-diabetes treatments for those with type 2 diabetes mellitus inadequately controlled by sulfonylurea monotherapy. Diabetology & metabolic syndrome 6, 73, doi:10.1186/1758-5996-6-73 (2014).
157. Palmer, S. C. et al. Erythropoiesis-stimulating agents for anaemia in adults with chronic kidney disease: a network meta-analysis. The Cochrane database of systematic reviews 2014, Cd010590, doi:10.1002/14651858.CD010590.pub2 (2014).
158. Palmerini, T. et al. Clinical outcomes with bioabsorbable polymer- versus durable polymer-based drug-eluting and bare-metal stents: evidence from a comprehensive network meta-analysis. Journal of the American College of Cardiology 63, 299-307, doi:10.1016/j.jacc.2013.09.061 (2014).
159. Palmerini, T. et al. Clinical outcomes with drug-eluting and bare-metal stents in patients with ST-segment elevation myocardial infarction: evidence from a comprehensive network meta-analysis. Journal of the American College of Cardiology 62, 496-504, doi:10.1016/j.jacc.2013.05.022 (2013).
160. Palmieri, C., Fullarton, J. R. & Brown, J. Comparative efficacy of bisphosphonates in metastatic breast and prostate cancer and multiple myeloma: a mixed-treatment meta-analysis. Clinical cancer research : an official journal of the American Association for Cancer Research 19, 6863-6872, doi:10.1158/1078-0432.CCR-13-2275 (2013).
161. Pandor, A. et al. Remote monitoring after recent hospital discharge in patients with heart failure: a systematic review and network meta-analysis. Heart (British Cardiac Society) 99, 1717-1726, doi:10.1136/heartjnl-2013-303811 (2013).
162. Patel, D. A. et al. 48-week efficacy and safety of dolutegravir relative to commonly used third agents in treatment-naive HIV-1-infected patients: a systematic review and network meta-analysis. PloS one 9, e105653, doi:10.1371/journal.pone.0105653 (2014).
163. Pechlivanoglou, P., Le, H. H., Daenen, S., Snowden, J. A. & Postma, M. J. Mixed treatment comparison of prophylaxis against invasive fungal infections in neutropenic patients receiving therapy for haematological malignancies: a systematic review. The Journal of antimicrobial chemotherapy 69, 1-11, doi:10.1093/jac/dkt329 (2014).
164. Peruzzi, M. et al. A network meta-analysis on randomized trials focusing on the preventive effect of statins on contrast-induced nephropathy. BioMed research international 2014, 213239, doi:10.1155/2014/213239 (2014).
165. Phan, K. et al. Comparing energy sources for surgical ablation of atrial fibrillation: a Bayesian network meta-analysis of randomized, controlled trials. European journal of cardio-thoracic surgery : official journal of the European Association for Cardio-thoracic Surgery 48, 201-211, doi:10.1093/ejcts/ezu408 (2015).
166. Piccolo, R. et al. Meta-analysis of randomized trials comparing the effectiveness of different strategies for the treatment of drug-eluting stent restenosis. The American journal of cardiology 114, 1339-1346, doi:10.1016/j.amjcard.2014.07.069 (2014).
167. Price, R., MacLennan, G., Glen, J. & Su, D. C. Selective digestive or oropharyngeal decontamination and topical oropharyngeal chlorhexidine for prevention of death in general intensive care: systematic review and network meta-analysis. BMJ (Clinical research ed.) 348, g2197, doi:10.1136/bmj.g2197 (2014).
168. Rashiq, S. et al. Efficacy of supplemental peripheral nerve blockade for hip fracture surgery: multiple treatment comparison. Canadian journal of anaesthesia = Journal canadien d'anesthesie 60, 230-243, doi:10.1007/s12630-012-9880-8 (2013).
169. Regnier, S., Malcolm, W., Allen, F., Wright, J. & Bezlyak, V. Efficacy of anti-VEGF and laser photocoagulation in the treatment of visual impairment due to diabetic macular edema: a systematic review and network meta-analysis. PloS one 9, e102309, doi:10.1371/journal.pone.0102309 (2014).
170. Reinecke, H. et al. Analgesic efficacy of opioids in chronic pain: recent meta-analyses. British journal of pharmacology 172, 324-333, doi:10.1111/bph.12634 (2015).
171. Rochwerg, B. et al. Fluid resuscitation in sepsis: a systematic review and network meta-analysis. Annals of internal medicine 161, 347-355, doi:10.7326/M14-0178 (2014).
172. Rollins, B. M., Silva, M. A., Donovan, J. L. & Kanaan, A. O. Evaluation of oral anticoagulants for the extended treatment of venous thromboembolism using a mixed-treatment comparison, meta-analytic approach. Clinical therapeutics 36, 1454-1464 e1453, doi:10.1016/j.clinthera.2014.06.033 (2014).
173. Roskell, N. S., Anzueto, A., Hamilton, A., Disse, B. & Becker, K. Once-daily long-acting beta-agonists for chronic obstructive pulmonary disease: an indirect comparison of olodaterol and indacaterol. International journal of chronic obstructive pulmonary disease 9, 813-824, doi:10.2147/COPD.S59673 (2014).
174. Roskell, N. S., Setyawan, J., Zimovetz, E. A. & Hodgkins, P. Systematic evidence synthesis of treatments for ADHD in children and adolescents: indirect treatment comparisons of lisdexamfetamine with methylphenidate and atomoxetine. Current medical research and opinion 30, 1673-1685, doi:10.1185/03007995.2014.904772 (2014).
175. Rotta, I. et al. Efficacy of topical antifungals in the treatment of dermatophytosis: a mixed-treatment comparison meta-analysis involving 14 treatments. JAMA dermatology 149, 341-349, doi:10.1001/jamadermatol.2013.1721 (2013).
176. Rudroju, N. et al. Comparative efficacy and safety of six antidepressants and anticonvulsants in painful diabetic neuropathy: a network meta-analysis. Pain physician 16, E705-714 (2013).
177. Samarasekera, E. J., Sawyer, L., Wonderling, D., Tucker, R. & Smith, C. H. Topical therapies for the treatment of plaque psoriasis: systematic review and network meta-analyses. The British journal of dermatology 168, 954-967, doi:10.1111/bjd.12276 (2013).
178. Schmid, M. K. et al. Efficacy and adverse events of aflibercept, ranibizumab and bevacizumab in age-related macular degeneration: a trade-off analysis. The British journal of ophthalmology 99, 141-146, doi:10.1136/bjophthalmol-2014-305149 (2015).
179. Schmitt, J. et al. Efficacy and safety of systemic treatments for moderate-to-severe psoriasis: meta-analysis of randomized controlled trials. The British journal of dermatology 170, 274-303, doi:10.1111/bjd.12663 (2014).
180. Schoenberg, M. B. et al. Laparoscopic Heller myotomy versus endoscopic balloon dilatation for the treatment of achalasia: a network meta-analysis. Annals of surgery 258, 943-952, doi:10.1097/SLA.0000000000000212 (2013).
181. Scott, D. A., Boye, K. S., Timlin, L., Clark, J. F. & Best, J. H. A network meta-analysis to compare glycaemic control in patients with type 2 diabetes treated with exenatide once weekly or liraglutide once daily in comparison with insulin glargine, exenatide twice daily or placebo. Diabetes, obesity & metabolism 15, 213-223, doi:10.1111/dom.12007 (2013).
182. Shamliyan, T. A. et al. Preventive pharmacologic treatments for episodic migraine in adults. Journal of general internal medicine 28, 1225-1237, doi:10.1007/s11606-013-2433-1 (2013).
183. Shamliyan, T. A., Kane, R. L. & Taylor, F. R. in Migraine in Adults: Preventive Pharmacologic Treatments (Agency for Healthcare Research and Quality (US), 2013).
184. Shams, T. et al. SSRIs for hot flashes: a systematic review and meta-analysis of randomized trials. Journal of general internal medicine 29, 204-213, doi:10.1007/s11606-013-2535-9 (2014).
185. Shi, K. Q. et al. Secondary prophylaxis of variceal bleeding for cirrhotic patients: a multiple-treatments meta-analysis. European journal of clinical investigation 43, 844-854, doi:10.1111/eci.12115 (2013).
186. Shu, T. et al. Indirect comparison of anti-TNF-α agents for active ankylosing spondylitis: mixed treatment comparison of randomized controlled trials. Clinical and experimental rheumatology 31, 717-722 (2013).
187. Signorovitch, J. E. et al. Comparative efficacy of biological treatments for moderate-to-severe psoriasis: a network meta-analysis adjusting for cross-trial differences in reference arm response. The British journal of dermatology 172, 504-512, doi:10.1111/bjd.13437 (2015).
188. Singh, J. A. et al. Adverse effects of biologics: a network meta-analysis and Cochrane overview. The Cochrane database of systematic reviews, CD008794, doi:10.1002/14651858.CD008794.pub2 (2011).
189. Singh, S. et al. Comparative efficacy of biologic therapy in biologic-naïve patients with Crohn disease: a systematic review and network meta-analysis. Mayo Clinic proceedings 89, 1621-1635, doi:10.1016/j.mayocp.2014.08.019 (2014).
190. Singh, S. et al. Comparative efficacy of pharmacologic interventions in preventing relapse of Crohn's disease after surgery: a systematic review and network meta-analysis. Gastroenterology 148, 64-76 e62; quiz e14, doi:10.1053/j.gastro.2014.09.031 (2015).
191. Skoetz, N. et al. Effect of initial treatment strategy on survival of patients with advanced-stage Hodgkin's lymphoma: a systematic review and network meta-analysis. The Lancet Oncology 14, 943-952, doi:10.1016/s1470-2045(13)70341-3 (2013).
192. Snedecor, S. J. et al. Systematic review and meta-analysis of pharmacological therapies for pain associated with postherpetic neuralgia and less common neuropathic conditions. International journal of clinical practice 68, 900-918, doi:10.1111/ijcp.12411 (2014).
193. Snedecor, S. J. et al. Systematic review and meta-analysis of pharmacological therapies for painful diabetic peripheral neuropathy. Pain practice : the official journal of World Institute of Pain 14, 167-184, doi:10.1111/papr.12054 (2014).
194. Sobieraj, D. M. et al. Comparative efficacy and safety of anticoagulants and aspirin for extended treatment of venous thromboembolism: A network meta-analysis. Thrombosis research 135, 888-896, doi:10.1016/j.thromres.2015.02.032 (2015).
195. Stagg, H. R. et al. Treatment of latent tuberculosis infection: a network meta-analysis. Annals of internal medicine 161, 419-428, doi:10.7326/M14-1019 (2014).
196. Stevens, J. W. et al. Preventing the progression to type 2 diabetes mellitus in adults at high risk: a systematic review and network meta-analysis of lifestyle, pharmacological and surgical interventions. Diabetes research and clinical practice 107, 320-331, doi:10.1016/j.diabres.2015.01.027 (2015).
197. Stidham, R. W. et al. Systematic review with network meta-analysis: the efficacy of anti-TNF agents for the treatment of Crohn's disease. Alimentary pharmacology & therapeutics 39, 1349-1362, doi:10.1111/apt.12749 (2014).
198. Stidham, R. W. et al. Systematic review with network meta-analysis: the efficacy of anti-tumour necrosis factor-alpha agents for the treatment of ulcerative colitis. Alimentary pharmacology & therapeutics 39, 660-671, doi:10.1111/apt.12644 (2014).
199. Stynes, G. et al. Once-daily fluticasone furoate/vilanterol 100/25 mcg versus twice daily combination therapies in COPD - mixed treatment comparisons of clinical efficacy. Respiratory research 16, 25, doi:10.1186/s12931-015-0184-8 (2015).
200. Sun, F. et al. Effects of glucagon-like peptide-1 receptor agonists on weight loss in patients with type 2 diabetes: a systematic review and network meta-analysis. Journal of diabetes research 2015, 157201, doi:10.1155/2015/157201 (2015).
201. Sun, F. et al. Gastrointestinal adverse events of glucagon-like peptide-1 receptor agonists in patients with type 2 diabetes: a systematic review and network meta-analysis. Diabetes technology & therapeutics 17, 35-42, doi:10.1089/dia.2014.0188 (2015).
202. Sun, F. et al. Effect of GLP-1 receptor agonists on waist circumference among type 2 diabetes patients: a systematic review and network meta-analysis. Endocrine 48, 794-803, doi:10.1007/s12020-014-0373-0 (2015).
203. Sun, F. et al. Effect of glucagon-like peptide-1 receptor agonists on lipid profiles among type 2 diabetes: a systematic review and network meta-analysis. Clinical therapeutics 37, 225-241 e228, doi:10.1016/j.clinthera.2014.11.008 (2015).
204. Sun, Y., van Valkenhoef, G. & Morel, T. A mixed treatment comparison of gabapentin enacarbil, pramipexole, ropinirole and rotigotine in moderate-to-severe restless legs syndrome. Current medical research and opinion 30, 2267-2278, doi:10.1185/03007995.2014.946124 (2014).
205. Tadrous, M. et al. Comparative gastrointestinal safety of bisphosphonates in primary osteoporosis: a network meta-analysis. Osteoporosis international : a journal established as result of cooperation between the European Foundation for Osteoporosis and the National Osteoporosis Foundation of the USA 25, 1225-1235, doi:10.1007/s00198-013-2576-2 (2014).
206. Thakur, D., Dickerson, S., Kumar Bhutani, M. & Junor, R. Impact of prolonged-release oxycodone/naloxone on outcomes affecting patients' daily functioning in comparison with extended-release tapentadol: a systematic review. Clinical therapeutics 37, 212-224, doi:10.1016/j.clinthera.2014.12.001 (2015).
207. Thorlund, K. et al. Comparative efficacy of triptans for the abortive treatment of migraine: a multiple treatment comparison meta-analysis. Cephalalgia : an international journal of headache 34, 258-267, doi:10.1177/0333102413508661 (2014).
208. Thorlund, K., Wu, P., Druyts, E., Eapen, S. & Mills, E. J. Nonergot dopamine-receptor agonists for treating Parkinson's disease - a network meta-analysis. Neuropsychiatric disease and treatment 10, 767-776, doi:10.2147/NDT.S60061 (2014).
209. van Walsem, A. et al. Relative benefit-risk comparing diclofenac to other traditional non-steroidal anti-inflammatory drugs and cyclooxygenase-2 inhibitors in patients with osteoarthritis or rheumatoid arthritis: a network meta-analysis. Arthritis research & therapy 17, 66, doi:10.1186/s13075-015-0554-0 (2015).
210. Vegter, S. & Tolley, K. A network meta-analysis of the relative efficacy of treatments for actinic keratosis of the face or scalp in Europe. PloS one 9, e96829, doi:10.1371/journal.pone.0096829 (2014).
211. Wang, C. et al. Mechanical ventilation modes for respiratory distress syndrome in infants: a systematic review and network meta-analysis. Critical care (London, England) 19, 108, doi:10.1186/s13054-015-0843-7 (2015).
212. Wang, H. et al. The effectiveness and safety of avanafil for erectile dysfunction: a systematic review and meta-analysis. Current medical research and opinion 30, 1565-1571, doi:10.1185/03007995.2014.909391 (2014).
213. Wang, J. C., Tian, J. H., Ge, L., Gan, Y. H. & Yang, K. H. Which is the best Chinese herb injection based on the FOLFOX regimen for gastric cancer? A network meta- analysis of randomized controlled trials. Asian Pacific journal of cancer prevention : APJCP 15, 4795-4800, doi:10.7314/apjcp.2014.15.12.4795 (2014).
214. Wang, L., Baser, O., Kutikova, L., Page, J. H. & Barron, R. The impact of primary prophylaxis with granulocyte colony-stimulating factors on febrile neutropenia during chemotherapy: a systematic review and meta-analysis of randomized controlled trials. Supportive care in cancer : official journal of the Multinational Association of Supportive Care in Cancer 23, 3131-3140, doi:10.1007/s00520-015-2686-9 (2015).
215. Wang, X. et al. Comparative effectiveness of oral drug therapies for lower urinary tract symptoms due to benign prostatic hyperplasia: a systematic review and network meta-analysis. PloS one 9, e107593, doi:10.1371/journal.pone.0107593 (2014).
216. Wang, Z. et al. Systematic literature review and network meta-analysis comparing bone-targeted agents for the prevention of skeletal-related events in cancer patients with bone metastasis. The oncologist 20, 440-449, doi:10.1634/theoncologist.2014-0328 (2015).
217. Wertli, M. M., Kessels, A. G., Perez, R. S., Bachmann, L. M. & Brunner, F. Rational pain management in complex regional pain syndrome 1 (CRPS 1)--a network meta-analysis. Pain medicine (Malden, Mass.) 15, 1575-1589, doi:10.1111/pme.12466 (2014).
218. Wiens, A. et al. Comparative efficacy of oral nucleoside or nucleotide analog monotherapy used in chronic hepatitis B: a mixed-treatment comparison meta-analysis. Pharmacotherapy 33, 144-151, doi:10.1002/phar.1188 (2013).
219. Wilhelmus, K. R. Antiviral treatment and other therapeutic interventions for herpes simplex virus epithelial keratitis. Cochrane Database of Systematic Reviews, doi:10.1002/14651858.CD002898.pub5 (2015).
220. Windecker, S. et al. Revascularisation versus medical treatment in patients with stable coronary artery disease: network meta-analysis. BMJ (Clinical research ed.) 348, g3859, doi:10.1136/bmj.g3859 (2014).
221. Wu, H. Y. et al. Comparative effectiveness of renin-angiotensin system blockers and other antihypertensive drugs in patients with diabetes: systematic review and bayesian network meta-analysis. BMJ (Clinical research ed.) 347, f6008, doi:10.1136/bmj.f6008 (2013).
222. Wu, M. S., Tan, S. C. & Xiong, T. Indirect comparison of randomised controlled trials: comparative efficacy of dexlansoprazole vs. esomeprazole in the treatment of gastro-oesophageal reflux disease. Alimentary pharmacology & therapeutics 38, 190-201, doi:10.1111/apt.12349 (2013).
223. Xiao, J., Wu, W. X., Ye, Y. Y., Lin, W. J. & Wang, L. A Network Meta-analysis of Randomized Controlled Trials Focusing on Different Allergic Rhinitis Medications. American journal of therapeutics 23, e1568-e1578, doi:10.1097/mjt.0000000000000242 (2016).
224. Xiong, T. et al. Comparative efficacy and safety of treatments for localised prostate cancer: an application of network meta-analysis. BMJ open 4, e004285, doi:10.1136/bmjopen-2013-004285 (2014).
225. Yang, B., Shi, J., Chen, X., Ma, B. & Sun, H. Efficacy and safety of therapies for acute ischemic stroke in China: a network meta-analysis of 13289 patients from 145 randomized controlled trials. PloS one 9, e88440, doi:10.1371/journal.pone.0088440 (2014).
226. Yang, Z., Ye, X., Wu, Q., Wu, K. & Fan, D. A network meta-analysis on the efficacy of 5-aminosalicylates, immunomodulators and biologics for the prevention of postoperative recurrence in Crohn's disease. International journal of surgery (London, England) 12, 516-522, doi:10.1016/j.ijsu.2014.02.010 (2014).
227. Yildiz, A., Nikodem, M., Vieta, E., Correll, C. U. & Baldessarini, R. J. A network meta-analysis on comparative efficacy and all-cause discontinuation of antimanic treatments in acute bipolar mania. Psychological medicine 45, 299-317, doi:10.1017/S0033291714001305 (2015).
228. Yuan, J. et al. Comparative effectiveness and safety of oral phosphodiesterase type 5 inhibitors for erectile dysfunction: a systematic review and network meta-analysis. European urology 63, 902-912, doi:10.1016/j.eururo.2013.01.012 (2013).
229. Zaccara, G., Giovannelli, F., Maratea, D., Fadda, V. & Verrotti, A. Neurological adverse events of new generation sodium blocker antiepileptic drugs. Meta-analysis of randomized, double-blinded studies with eslicarbazepine acetate, lacosamide and oxcarbazepine. Seizure 22, 528-536, doi:10.1016/j.seizure.2013.03.016 (2013).
230. Zeng, C. et al. Electrical stimulation for pain relief in knee osteoarthritis: systematic review and network meta-analysis. Osteoarthritis and cartilage 23, 189-202, doi:10.1016/j.joca.2014.11.014 (2015).
231. Zeppetella, G., Davies, A., Eijgelshoven, I. & Jansen, J. P. A network meta-analysis of the efficacy of opioid analgesics for the management of breakthrough cancer pain episodes. Journal of pain and symptom management 47, 772-785 e775, doi:10.1016/j.jpainsymman.2013.05.020 (2014).
232. Zhang, Y. W. et al. Chemotherapy for patients with gastric cancer after complete resection: a network meta-analysis. World journal of gastroenterology 20, 584-592, doi:10.3748/wjg.v20.i2.584 (2014).
233. Zhu, G. Q. et al. Network meta-analysis of randomized controlled trials: efficacy and safety of UDCA-based therapies in primary biliary cirrhosis. Medicine 94, e609, doi:10.1097/md.0000000000000609 (2015).
234. Zhu, G. Q. et al. Systematic review with network meta-analysis: the comparative effectiveness and safety of interventions in patients with overt hepatic encephalopathy. Alimentary pharmacology & therapeutics 41, 624-635, doi:10.1111/apt.13122 (2015).
235. Zhu, Z. et al. Efficacy and toxicity of external-beam radiation therapy for localised prostate cancer: a network meta-analysis. British journal of cancer 110, 2396-2404, doi:10.1038/bjc.2014.197 (2014).
236. Ziakas, P. D., Zervou, F. N., Zacharioudakis, I. M. & Mylonakis, E. Graft-versus-host disease prophylaxis after transplantation: a network meta-analysis. PloS one 9, e114735, doi:10.1371/journal.pone.0114735 (2014).
237. Zintzaras, E. et al. Assessment of the relative effectiveness and tolerability of treatments of type 2 diabetes mellitus: a network meta-analysis. Clinical therapeutics 36, 1443-1453 e1449, doi:10.1016/j.clinthera.2014.06.035 (2014).
238. Reginster J-, Bianic F, Campbell R, Martin M, Williams SA, Fitzpatrick LA. Abaloparatide for risk reduction of nonvertebral and vertebral fractures in postmenopausal women with osteoporosis: a network meta-analysis Osteoporos Int. 2019 Jul;30(7):1465-1473. doi: 10.1007/s00198-019-04947-2. Epub 2019 Apr 6.
239. Miwa H, Igarashi A, Teng L, Uda A, Deguchi H, Tango T. Systematic review with network meta-analysis: indirect comparison of the efficacy of vonoprazan and proton-pump inhibitors for maintenance treatment of gastroesophageal reflux disease J Gastroenterol. 2019 Aug;54(8):718-729. doi: 10.1007/s00535-019-01572-y. Epub 2019 Mar 27.
240. Li JK, Qiu S, Jin K, Zheng XN, Tu X, Bi SW, Liao XY, Bao YG, Yang L, Wei Q. Efficacy and safety of phosphodiesterase type 5 inhibitors for the treatment of distal ureteral calculi of 5 to 10 mm in size: A systematic review and network meta-analysis Kaohsiung J Med Sci. 2019 May;35(5):257-264. doi: 10.1002/kjm2.12048. Epub 2019 Mar 21.
241. Strand V, Elaine Husni M, Betts KA, Song Y, Singh R, Griffith J, Beppu M, Zhao J, Ganguli A. Network meta-analysis and cost per responder of targeted Immunomodulators in the treatment of active psoriatic arthritis BMC Rheumatol. 2018 Feb 12;2:3. doi: 10.1186/s41927-018-0011-1. eCollection 2018.
242. Miyazaki H, Igarashi A, Takeuchi T, Teng L, Uda A, Deguchi H, Higuchi K, Tango T. Vonoprazan versus proton-pump inhibitors for healing gastroesophageal reflux disease: A systematic review J Gastroenterol Hepatol. 2019 Aug;34(8):1316-1328. doi: 10.1111/jgh.14664. Epub 2019 Apr 11.
243. Holleman MS, van Tinteren H, Groen HJ, Al MJ, Uyl-de Groot CA. First-line tyrosine kinase inhibitors in EGFR mutation-positive non-small-cell lung cancer: a network meta-analysis Onco Targets Ther. 2019 Feb 20;12:1413-1421. doi: 10.2147/OTT.S189438. eCollection 2019.
244. Khan SU, Khan MU, Rahman H, Khan MS, Riaz H, Novak M, Opoku-Asare I, Kaluski E. A Bayesian network meta-analysis of preventive strategies for contrast-induced nephropathy after cardiac catheterization Cardiovasc Revasc Med. 2019 Jan;20(1):29-37. doi: 10.1016/j.carrev.2018.06.005. Epub 2018 Jun 12.
245. Wang R, van Welie N, van Rijswijk J, Johnson NP, Norman RJ, Dreyer K, Mijatovic V, Mol BW. Effectiveness on fertility outcome of tubal flushing with different contrast media: systematic review and network meta-analysis Ultrasound Obstet Gynecol. 2019 Aug;54(2):172-181. doi: 10.1002/uog.20238. Epub 2019 Jun 26.
246. Wu IXY, Wong CHL, Ho RST, Cheung WKW, Ford AC, Wu JCY, Mak ADP, Cramer H, Chung VCH. Acupuncture and related therapies for treating irritable bowel syndrome: overview of systematic reviews and network meta-analysis Therap Adv Gastroenterol. 2019 Jan 20;12:1756284818820438. doi: 10.1177/1756284818820438. eCollection 2019.
247. Scapini KB, Bohlke M, Moraes OA, Rodrigues CG, Inácio JF, Sbruzzi G, Leguisamo CP, Sanches IC, Tourinho Filho H, Irigoyen MC. Combined training is the most effective training modality to improve aerobic capacity and blood pressure control in people requiring haemodialysis for end-stage renal disease: systematic review and network meta-analysis J Physiother. 2019 Jan;65(1):4-15. doi: 10.1016/j.jphys.2018.11.008. Epub 2018 Dec 21.
248. Gregori D, Giacovelli G, Minto C, Barbetta B, Gualtieri F, Azzolina D, Vaghi P, Rovati LC. Association of Pharmacological Treatments With Long-term Pain Control in Patients With Knee Osteoarthritis: A Systematic Review and Meta-analysis JAMA. 2018 Dec 25;320(24):2564-2579. doi: 10.1001/jama.2018.19319.
249. Jin K, Deng L, Qiu S, Tu X, Li J, Bao Y, Yang L, Wei Q. Comparative efficacy and safety of phosphodiesterase-5 inhibitors with selective serotonin reuptake inhibitors in men with premature ejaculation: A systematic review and Bayesian network meta-analysis Medicine (Baltimore). 2018 Dec;97(49):e13342. doi: 10.1097/MD.0000000000013342.
250. Jarde A, Lutsiv O, Beyene J, McDonald SD. Vaginal progesterone, oral progesterone, 17-OHPC, cerclage, and pessary for preventing preterm birth in at-risk singleton pregnancies: an updated systematic review and network meta-analysis BJOG. 2019 Apr;126(5):556-567. doi: 10.1111/1471-0528.15566. Epub 2018 Dec 29.
251. Ashaye AO, Khankhel Z, Xu Y, Fahrbach K, Mokgokong R, Orme ME, Lang K, Cappelleri JC, Mamolo C. A comparative evaluation of gemtuzumab ozogamicin + daunorubicin-cytarabine and other treatments for newly diagnosed acute myeloid leukemia Future Oncol. 2019 Feb;15(6):663-681. doi: 10.2217/fon-2018-0512. Epub 2018 Nov 19.
252. Fei Y, Tsoi MF, Cheung BMY. Determining the Optimal Systolic Blood Pressure for Hypertensive Patients: A Network Meta-analysis Can J Cardiol. 2018 Dec;34(12):1581-1589. doi: 10.1016/j.cjca.2018.08.013. Epub 2018 Aug 10.
253. Datta NR, Stutz E, Gomez S, Bodis S. Efficacy and Safety Evaluation of the Various Therapeutic Options in Locally Advanced Cervix Cancer: A Systematic Review and Network Meta-Analysis of Randomized Clinical Trials Int J Radiat Oncol Biol Phys. 2019 Feb 1;103(2):411-437. doi: 10.1016/j.ijrobp.2018.09.037. Epub 2018 Nov 1.
254. Azharuddin M, Adil M, Ghosh P, Sharma M. Sodium-glucose cotransporter 2 inhibitors and fracture risk in patients with type 2 diabetes mellitus: A systematic literature review and Bayesian network meta-analysis of randomized controlled trials Diabetes Res Clin Pract. 2018 Dec;146:180-190. doi: 10.1016/j.diabres.2018.10.019. Epub 2018 Oct 31.
255. Paschos P, Katsoula A, Salanti G, Giouleme O, Athanasiadou E, Tsapas A. Systematic review with network meta-analysis: the impact of medical interventions for moderate-to-severe ulcerative colitis on health-related quality of life Aliment Pharmacol Ther. 2018 Dec;48(11-12):1174-1185. doi: 10.1111/apt.15005. Epub 2018 Oct 30.
256. Yokoe T, Hayashida T, Nagayama A, Nakashoji A, Maeda H, Seki T, Takahashi M, Takano T, Abe T, Kitagawa Y. Effectiveness of Antiemetic Regimens for Highly Emetogenic Chemotherapy-Induced Nausea and Vomiting: A Systematic Review and Network Meta-Analysis Oncologist. 2019 Jun;24(6):e347-e357. doi: 10.1634/theoncologist.2018-0140. Epub 2018 Oct 17.
257. Kishan AU, Wang X, Seiferheld W, Collette L, Sandler KA, Sandler HM, Bolla M, Maingon P, De Reijke T, Hanks GE, Nickols NG, Rettig M, Drakaki A, Reiter RE, Spratt DE, Kupelian PA, Steinberg ML, King CR. Association of Gleason Grade With Androgen Deprivation Therapy Duration and Survival Outcomes: A Systematic Review and Patient-Level Meta-analysis JAMA Oncol. 2019 Jan 1;5(1):91-96. doi: 10.1001/jamaoncol.2018.3732.
258. McCool R, Fleetwood K, Glanville J, Arber M, Goodall H, Naidoo S. Systematic Review and Network Meta-Analysis of Treatments for Chemotherapy-Naive Patients with Asymptomatic/Mildly Symptomatic Metastatic Castration-Resistant Prostate Cancer Value Health. 2018 Oct;21(10):1259-1268. doi: 10.1016/j.jval.2018.03.012. Epub 2018 May 4.
259. Iantorno M, Lipinski MJ, Garcia-Garcia HM, Forrestal BJ, Rogers T, Gajanana D, Buchanan KD, Torguson R, Weintraub WS, Waksman R. Meta-Analysis of the Impact of Strut Thickness on Outcomes in Patients With Drug-Eluting Stents in a Coronary Artery Am J Cardiol. 2018 Nov 15;122(10):1652-1660. doi: 10.1016/j.amjcard.2018.07.040. Epub 2018 Sep 8.
260. Lehert P, Falissard B. Multiple treatment comparison in narcolepsy: a network meta-analysis Sleep. 2018 Dec 1;41(12):zsy185. doi: 10.1093/sleep/zsy185.
261. McInnes IB, Nash P, Ritchlin C, Choy EH, Kanters S, Thom H, Gandhi K, Pricop L, Jugl SM. Secukinumab for psoriatic arthritis: comparative effectiveness versus licensed biologics/apremilast: a network meta-analysis J Comp Eff Res. 2018 Nov;7(11):1107-1123. doi: 10.2217/cer-2018-0075. Epub 2018 Sep 19.
262. Bighelli I, Salanti G, Huhn M, Schneider-Thoma J, Krause M, Reitmeir C, Wallis S, Schwermann F, Pitschel-Walz G, Barbui C, Furukawa TA, Leucht S. Psychological interventions to reduce positive symptoms in schizophrenia: systematic review and network meta-analysis World Psychiatry. 2018 Oct;17(3):316-329. doi: 10.1002/wps.20577.
263. Yeo YH, Hsu CC, Lee CC, Ho HJ, Lin JT, Wu MS, Liou JM, Wu CY; Taiwan Gastrointestinal Disease and Helicobacter Consortium. Systematic review and network meta-analysis: Comparative effectiveness of therapies for second-line Helicobacter pylori eradication J Gastroenterol Hepatol. 2019 Jan;34(1):59-67. doi: 10.1111/jgh.14462. Epub 2018 Oct 11.
264. Murray E, Ellis A, Butylkova Y, Skup M, Kalabic J, Garg V. Systematic review and network meta-analysis: effect of biologics on radiographic progression in rheumatoid arthritis J Comp Eff Res. 2018 Oct;7(10):959-974. doi: 10.2217/cer-2017-0106. Epub 2018 Aug 21.
265. Sharma M, Singh S, Desai V, Shah VH, Kamath PS, Murad MH, Simonetto DA. Comparison of Therapies for Primary Prevention of Esophageal Variceal Bleeding: A Systematic Review and Network Meta-analysis Hepatology. 2019 Apr;69(4):1657-1675. doi: 10.1002/hep.30220. Epub 2019 Feb 20.
266. Iannaccone M, Saint-Hilary G, Menardi D, Vadalà P, Bernardi A, Bianco M, Montefusco A, Omedè P, D'Amico S, Piazza F, Scacciatella P, D'Amico M, Moretti C, Biondi-Zoccai G, Gasparini M, Gaita F, D'Ascenzo F. Network meta-analysis of studies comparing closure devices for femoral access after percutaneous coronary intervention J Cardiovasc Med (Hagerstown). 2018 Oct;19(10):586-596. doi: 10.2459/JCM.0000000000000697.
267. Wang Y, Zhu J, Qin Z, Wang Y, Chen C, Wang Y, Zhou X, Zhang Q, Meng X, Song N. Optimal biopsy strategy for prostate cancer detection by performing a Bayesian network meta-analysis of randomized controlled trials J Cancer. 2018 Jun 5;9(13):2237-2248. doi: 10.7150/jca.24690. eCollection 2018.
268. Leonart LP, Ferreira VL, Tonin FS, Fernandez-Llimos F, Pontarolo R. Medical Treatments for Acromegaly: A Systematic Review and Network Meta-Analysis Value Health. 2018 Jul;21(7):874-880. doi: 10.1016/j.jval.2017.12.014. Epub 2018 Feb 8.
269. Madenidou AV, Paschos P, Karagiannis T, Katsoula A, Athanasiadou E, Kitsios K, Bekiari E, Matthews DR, Tsapas A. Comparative Benefits and Harms of Basal Insulin Analogues for Type 2 Diabetes: A Systematic Review and Network Meta-analysis Ann Intern Med. 2018 Aug 7;169(3):165-174. doi: 10.7326/M18-0443. Epub 2018 Jul 10.
270. Zhu L, Ma Y, Ye S, Shu Z. Acupuncture for Diarrhoea-Predominant Irritable Bowel Syndrome: A Network Meta-Analysis Evid Based Complement Alternat Med. 2018 May 27;2018:2890465. doi: 10.1155/2018/2890465. eCollection 2018.
271. Spertus J, Horvitz-Lennon M, Abing H, Normand SL. Risk of weight gain for specific antipsychotic drugs: a meta-analysis NPJ Schizophr. 2018 Jun 27;4(1):12. doi: 10.1038/s41537-018-0053-9.
272. Davies C, Radua J, Cipriani A, Stahl D, Provenzani U, McGuire P, Fusar-Poli P. Efficacy and Acceptability of Interventions for Attenuated Positive Psychotic Symptoms in Individuals at Clinical High Risk of Psychosis: A Network Meta-Analysis Front Psychiatry. 2018 Jun 12;9:187. doi: 10.3389/fpsyt.2018.00187. eCollection 2018.
273. Cai XJ, Wang L, Hu CM. Efficacy of different drugs in the treatment of minimal hepatic encephalopathy: A network meta-analysis involving 826 patients based on 10 randomized controlled trials J Cell Biochem. 2018 Nov;119(10):8336-8345. doi: 10.1002/jcb.26886. Epub 2018 Jun 22.
274. Hao Z, Wang X, Zhang X. Comparing surgical interventions for intertrochanteric hip fracture by blood loss and operation time: a network meta-analysis J Orthop Surg Res. 2018 Jun 22;13(1):157. doi: 10.1186/s13018-018-0852-8.
275. Phan K, Lloyd D, Wilson-Smith A, Leung V, Andric M. Intraocular bleeding in patients managed with novel oral anticoagulation and traditional anticoagulation: a network meta-analysis and systematic review Br J Ophthalmol. 2018 Jun 20:bjophthalmol-2018-312198. doi: 10.1136/bjophthalmol-2018-312198. Online ahead of print.
276. Sridharan K, Sivaramakrishnan G, Sequeira RP, Al-Khaja KA. Pharmacological interventions for premature ejaculation: a mixed-treatment comparison network meta-analysis of randomized clinical trials Int J Impot Res. 2018 Oct;30(5):215-223. doi: 10.1038/s41443-018-0030-x. Epub 2018 Jun 20.
277. Singh S, Fumery M, Sandborn WJ, Murad MH. Systematic review and network meta-analysis: first- and second-line biologic therapies for moderate-severe Crohn's disease Aliment Pharmacol Ther. 2018 Aug;48(4):394-409. doi: 10.1111/apt.14852. Epub 2018 Jun 19.
278. Song P, Huang C, Wang Y. The efficacy and safety comparison of docetaxel, cabazitaxel, estramustine, and mitoxantrone for castration-resistant prostate cancer: A network meta-analysis Int J Surg. 2018 Aug;56:133-140. doi: 10.1016/j.ijsu.2018.06.010. Epub 2018 Jun 12.
279. Norman G, Westby MJ, Rithalia AD, Stubbs N, Soares MO, Dumville JC. Dressings and topical agents for treating venous leg ulcers Cochrane Database Syst Rev. 2018 Jun 15;6(6):CD012583. doi: 10.1002/14651858.CD012583.pub2.
280. Wang H, Tong H, Liu H, Wang Y, Wang R, Gao H, Yu P, Lv Y, Chen S, Wang G, Liu M, Li Y, Yu K, Wang C. Effectiveness of antimicrobial-coated central venous catheters for preventing catheter-related blood-stream infections with the implementation of bundles: a systematic review and network meta-analysis Ann Intensive Care. 2018 Jun 15;8(1):71. doi: 10.1186/s13613-018-0416-4.
281. Doosti-Irani A, Holakouie-Naieni K, Rahimi-Foroushani A, Mansournia MA, Haddad P. A network meta-analysis of the treatments for esophageal squamous cell carcinoma in terms of survival Crit Rev Oncol Hematol. 2018 Jul;127:80-90. doi: 10.1016/j.critrevonc.2018.05.007. Epub 2018 May 17.
282. Zhang Z, Cheng L, Wang R, Cen Y, Li Z. Effects and safety of triamcinolone acetonide-controlled common therapy in keloid treatment: a Bayesian network meta-analysis Ther Clin Risk Manag. 2018 May 29;14:973-980. doi: 10.2147/TCRM.S162315. eCollection 2018.
283. Tan PS, Aguiar P Jr, Haaland B, Lopes G. Addition of abiraterone, docetaxel, bisphosphonate, celecoxib or combinations to androgen-deprivation therapy (ADT) for metastatic hormone-sensitive prostate cancer (mHSPC): a network meta-analysis Prostate Cancer Prostatic Dis. 2018 Nov;21(4):516-523. doi: 10.1038/s41391-018-0055-8. Epub 2018 Jun 6.
284. Tsivgoulis G, Katsanos AH, Mavridis D, Frogoudaki A, Vrettou AR, Ikonomidis I, Parissis J, Deftereos S, Karapanayiotides T, Palaiodimou L, Filippatou A, Perren F, Hadjigeorgiou G, Alexandrov AW, Mitsias PD, Alexandrov AV. Percutaneous patent foramen ovale closure for secondary stroke prevention: Network meta-analysis Neurology. 2018 Jul 3;91(1):e8-e18. doi: 10.1212/WNL.0000000000005739. Epub 2018 Jun 6.
285. Matcham F, Galloway J, Hotopf M, Roberts E, Scott IC, Steer S, Norton S. The Impact of Targeted Rheumatoid Arthritis Pharmacologic Treatment on Mental Health: A Systematic Review and Network Meta-Analysis Arthritis Rheumatol. 2018 Sep;70(9):1377-1391. doi: 10.1002/art.40565. Epub 2018 Jul 31.
286. Debiasi M, Polanczyk CA, Ziegelmann P, Barrios C, Cao H, Dignam JJ, Goss P, Bychkovsky B, Finkelstein DM, Guindalini RS, Filho P, Albuquerque C, Reinert T, de Azambuja E, Olopade O. Efficacy of Anti-HER2 Agents in Combination With Adjuvant or Neoadjuvant Chemotherapy for Early and Locally Advanced HER2-Positive Breast Cancer Patients: A Network Meta-Analysis Front Oncol. 2018 May 22;8:156. doi: 10.3389/fonc.2018.00156. eCollection 2018.
287. Zhuang XD, He X, Yang DY, Guo Y, He JG, Xiao HP, Liao XX. Comparative cardiovascular outcomes in the era of novel anti-diabetic agents: a comprehensive network meta-analysis of 166,371 participants from 170 randomized controlled trials Cardiovasc Diabetol. 2018 Jun 5;17(1):79. doi: 10.1186/s12933-018-0722-z.
288. Lee CH, Lin C, Ho CL, Lin JC. Primary Fungal Prophylaxis in Hematological Malignancy: a Network Meta-Analysis of Randomized Controlled Trials Antimicrob Agents Chemother. 2018 Jul 27;62(8):e00355-18. doi: 10.1128/AAC.00355-18. Print 2018 Aug.
289. Dong P, Tang X, Cheng R, Wang J. Comparison of the Efficacy of Different Analgesia Treatments for Total Knee Arthroplasty: A Network Meta-Analysis Clin J Pain. 2018 Nov;34(11):1047-1060. doi: 10.1097/AJP.0000000000000631.
290. Hale P, Hahn AW, Rathi N, Pal SK, Haaland B, Agarwal N. Treatment of metastatic renal cell carcinoma in older patients: A network meta-analysis J Geriatr Oncol. 2019 Jan;10(1):149-154. doi: 10.1016/j.jgo.2018.05.010. Epub 2018 Jun 1.
291. Liew TM, Lee CS. Reappraising the Efficacy and Acceptability of Multicomponent Interventions for Caregiver Depression in Dementia: The Utility of Network Meta-Analysis Gerontologist. 2019 Jul 16;59(4):e380-e392. doi: 10.1093/geront/gny061.
292. Zhang W, Sun H, Atiquzzaman M, Sou J, Anis AH, Cooper C. Influenza vaccination for HIV-positive people: Systematic review and network meta-analysis Vaccine. 2018 Jun 27;36(28):4077-4086. doi: 10.1016/j.vaccine.2018.05.077. Epub 2018 May 30.
293. Davies C, Cipriani A, Ioannidis JPA, Radua J, Stahl D, Provenzani U, McGuire P, Fusar-Poli P. Lack of evidence to favor specific preventive interventions in psychosis: a network meta-analysis World Psychiatry. 2018 Jun;17(2):196-209. doi: 10.1002/wps.20526.
294. Yoon BH, Lee YK, Kim KC, Ha YC, Koo KH. No differences in the efficacy among various core decompression modalities and non-operative treatment: a network meta-analysis Int Orthop. 2018 Dec;42(12):2737-2743. doi: 10.1007/s00264-018-3977-9. Epub 2018 May 31.
295. Binde CD, Tvete IF, Gåsemyr J, Natvig B, Klemp M. A multiple treatment comparison meta-analysis of monoamine oxidase type B inhibitors for Parkinson's disease Br J Clin Pharmacol. 2018 Sep;84(9):1917-1927. doi: 10.1111/bcp.13651. Epub 2018 Jun 25.
296. Ricci C, Pagano N, Taffurelli G, Pacilio CA, Migliori M, Bazzoli F, Casadei R, Minni F. Comparison of Efficacy and Safety of 4 Combinations of Laparoscopic and Intraoperative Techniques for Management of Gallstone Disease With Biliary Duct Calculi: A Systematic Review and Network Meta-analysis JAMA Surg. 2018 Jul 18;153(7):e181167. doi: 10.1001/jamasurg.2018.1167. Epub 2018 Jul 18.
297. Bessissow T, Dulai PS, Restellini S, Landry T, Bisschops R, Murad MH, Singh S. Comparison of Endoscopic Dysplasia Detection Techniques in Patients With Ulcerative Colitis: A Systematic Review and Network Meta-analysis Inflamm Bowel Dis. 2018 Nov 29;24(12):2518-2526. doi: 10.1093/ibd/izy188.
298. Komajda M, Böhm M, Borer JS, Ford I, Tavazzi L, Pannaux M, Swedberg K. Incremental benefit of drug therapies for chronic heart failure with reduced ejection fraction: a network meta-analysis Eur J Heart Fail. 2018 Sep;20(9):1315-1322. doi: 10.1002/ejhf.1234. Epub 2018 Jun 19.
299. Deng L, Qiu S, Yang Y, Wang L, Li Y, Lin J, Wei Q, Yang L, Wang D, Liu M. Efficacy and tolerability of pharmacotherapy for post-stroke depression: a network meta-analysis Oncotarget. 2018 Jan 3;9(34):23718-23728. doi: 10.18632/oncotarget.23891. eCollection 2018 May 4.
300. Fillingham YA, Ramkumar DB, Jevsevar DS, Yates AJ, Shores P, Mullen K, Bini SA, Clarke HD, Schemitsch E, Johnson RL, Memtsoudis SG, Sayeed SA, Sah AP, Della Valle CJ. The Efficacy of Tranexamic Acid in Total Knee Arthroplasty: A Network Meta-Analysis J Arthroplasty. 2018 Oct;33(10):3090-3098.e1. doi: 10.1016/j.arth.2018.04.043. Epub 2018 May 5.
301. Krause M, Zhu Y, Huhn M, Schneider-Thoma J, Bighelli I, Chaimani A, Leucht S. Efficacy, acceptability, and tolerability of antipsychotics in children and adolescents with schizophrenia: A network meta-analysis Eur Neuropsychopharmacol. 2018 Jun;28(6):659-674. doi: 10.1016/j.euroneuro.2018.03.008. Epub 2018 May 24.
302. Gupta AK, Mays RR, Dotzert MS, Versteeg SG, Shear NH, Piguet V. Efficacy of non-surgical treatments for androgenetic alopecia: a systematic review and network meta-analysis J Eur Acad Dermatol Venereol. 2018 Dec;32(12):2112-2125. doi: 10.1111/jdv.15081. Epub 2018 Jul 11.
303. Wang Y, Zhang H, Shen W, He P, Zhou Z. Effectiveness and tolerability of targeted drugs for the treatment of metastatic castration-resistant prostate cancer: a network meta-analysis of randomized controlled trials J Cancer Res Clin Oncol. 2018 Sep;144(9):1751-1768. doi: 10.1007/s00432-018-2664-y. Epub 2018 May 24.
304. Wei Y, Liao H, Ye J. Therapeutic effects of various therapeutic strategies on non-exudative age-related macular degeneration: A PRISMA-compliant network meta-analysis of randomized controlled trials Medicine (Baltimore). 2018 May;97(21):e10422. doi: 10.1097/MD.0000000000010422.
305. Cao B, Rosenblat JD, Brietzke E, Park C, Lee Y, Musial N, Pan Z, Mansur RB, McIntyre RS. Comparative efficacy and acceptability of antidiabetic agents for Alzheimer's disease and mild cognitive impairment: A systematic review and network meta-analysis Diabetes Obes Metab. 2018 Oct;20(10):2467-2471. doi: 10.1111/dom.13373. Epub 2018 Jun 19.
306. Zhang T, Feng F, Zhao W, Tian J, Yao Y, Zhou C, Dong S, Wang C, Zang C, Lv Q, Sun C. Effect of first-line endocrine therapy in patients with hormone-sensitive advanced breast cancer: a network meta-analysis Onco Targets Ther. 2018 May 8;11:2647-2656. doi: 10.2147/OTT.S165681. eCollection 2018.
307. Wang W, Zhang Z, Gu C, Liu Q, Liang Z, He W, Chen J, Lai J. The optimal choice for pancreatic anastomosis after pancreaticoduodenectomy: A network meta-analysis of randomized control trials Int J Surg. 2018 Sep;57:111-116. doi: 10.1016/j.ijsu.2018.04.005. Epub 2018 May 17.
308. Gagelmann N, Ljungman P, Styczynski J, Kröger N. Comparative Efficacy and Safety of Different Antiviral Agents for Cytomegalovirus Prophylaxis in Allogeneic Hematopoietic Cell Transplantation: A Systematic Review and Meta-Analysis Biol Blood Marrow Transplant. 2018 Oct;24(10):2101-2109. doi: 10.1016/j.bbmt.2018.05.017. Epub 2018 May 16.
309. Tsikopoulos K, Mavridis D, Georgiannos D, Vasiliadis HS. Does Multimodal Rehabilitation for Ankle Instability Improve Patients' Self-assessed Functional Outcomes? A Network Meta-analysis Clin Orthop Relat Res. 2018 Jun;476(6):1295-1310. doi: 10.1097/01.blo.0000534691.24149.a2.
310. Liang JH, Xu Y, Lin L, Jia RX, Zhang HB, Hang L. Comparison of multiple interventions for older adults with Alzheimer disease or mild cognitive impairment: A PRISMA-compliant network meta-analysis Medicine (Baltimore). 2018 May;97(20):e10744. doi: 10.1097/MD.0000000000010744.
311. Lee A, Kim TH, Lee HH, Kim YS, Enkhbold T, Lee B, Park YJ, Song K. Therapeutic Approaches to Atrophic Vaginitis in Postmenopausal Women: A Systematic Review with a Network Meta-analysis of Randomized Controlled Trials J Menopausal Med. 2018 Apr;24(1):1-10. doi: 10.6118/jmm.2018.24.1.1. Epub 2018 Apr 30.
312. Zeeck A, Herpertz-Dahlmann B, Friederich HC, Brockmeyer T, Resmark G, Hagenah U, Ehrlich S, Cuntz U, Zipfel S, Hartmann A. Psychotherapeutic Treatment for Anorexia Nervosa: A Systematic Review and Network Meta-Analysis Front Psychiatry. 2018 May 1;9:158. doi: 10.3389/fpsyt.2018.00158. eCollection 2018.
313. Li JL, Fan GY, Liu YJ, Zeng ZH, Huang JJ, Yang ZM, Meng XY. Long-Term Efficacy of Maintenance Therapy for Multiple Myeloma: A Quantitative Synthesis of 22 Randomized Controlled Trials Front Pharmacol. 2018 Apr 30;9:430. doi: 10.3389/fphar.2018.00430. eCollection 2018.
314. Campos MSA, Ayres LR, Morelo MRS, Carizio FAM, Pereira LRL. Comparative efficacy of antiepileptic drugs for patients with generalized epileptic seizures: systematic review and network meta-analyses Int J Clin Pharm. 2018 Jun;40(3):589-598. doi: 10.1007/s11096-018-0641-9. Epub 2018 May 9.
315. Qin B, Chen H, Gao W, Zhao LB, Zhao MJ, Qin HX, Chen W, Chen L, Yang MX. Efficacy, acceptability, and tolerability of antidepressant treatments for patients with post-stroke depression: a network meta-analysis Braz J Med Biol Res. 2018;51(7):e7218. doi: 10.1590/1414-431x20187218. Epub 2018 May 7.
316. Cui JY, Zhou RR, Han S, Wang TS, Wang LQ, Xie XH. Statin therapy on glycemic control in type 2 diabetic patients: A network meta-analysis J Clin Pharm Ther. 2018 Aug;43(4):556-570. doi: 10.1111/jcpt.12690. Epub 2018 May 7.
317. Siskind DJ, Lee M, Ravindran A, Zhang Q, Ma E, Motamarri B, Kisely S. Augmentation strategies for clozapine refractory schizophrenia: A systematic review and meta-analysis Aust N Z J Psychiatry. 2018 Aug;52(8):751-767. doi: 10.1177/0004867418772351. Epub 2018 May 6.
318. Hwang SD, Lee JH, Lee SW, Park KM, Kim JK, Kim MJ, Song JH. Efficacy and Safety of Induction Therapy in Kidney Transplantation: A Network Meta-Analysis Transplant Proc. 2018 May;50(4):987-992. doi: 10.1016/j.transproceed.2018.01.022.
319. Luthra P, Burr NE, Brenner DM, Ford AC. Efficacy of pharmacological therapies for the treatment of opioid-induced constipation: systematic review and network meta-analysis Gut. 2019 Mar;68(3):434-444. doi: 10.1136/gutjnl-2018-316001. Epub 2018 May 5.
320. Slade E, Keeney E, Mavranezouli I, Dias S, Fou L, Stockton S, Saxon L, Waller G, Turner H, Serpell L, Fairburn CG, Kendall T. Treatments for bulimia nervosa: a network meta-analysis Psychol Med. 2018 Dec;48(16):2629-2636. doi: 10.1017/S0033291718001071. Epub 2018 May 6.
321. Cai Z, Yin Y, Yin Y, Shen C, Wang J, Yin X, Chen Z, Zhou Y, Zhang B. Comparative effectiveness of adjuvant treatments for resected gastric cancer: a network meta-analysis Gastric Cancer. 2018 Nov;21(6):1031-1040. doi: 10.1007/s10120-018-0831-0. Epub 2018 May 4.
322. Sridharan K, Sivaramakrishnan G. Efficacy and safety of iron chelators in thalassemia and sickle cell disease: a multiple treatment comparison network meta-analysis and trial sequential analysis Expert Rev Clin Pharmacol. 2018 Jun;11(6):641-650. doi: 10.1080/17512433.2018.1473760. Epub 2018 May 18.
323. Wolfe Z, Khan SU, Nasir F, Raghu Subramanian C, Lash B. A systematic review and Bayesian network meta-analysis of risk of intracranial hemorrhage with direct oral anticoagulants J Thromb Haemost. 2018 Jul;16(7):1296-1306. doi: 10.1111/jth.14131. Epub 2018 May 30.
324. Tsoi KK, Chan JY, Chan FC, Hirai HW, Kwok TC, Wong SY. Monotherapy Is Good Enough for Patients with Mild-to-Moderate Alzheimer's Disease: A Network Meta-analysis of 76 Randomized Controlled Trials Clin Pharmacol Ther. 2019 Jan;105(1):121-130. doi: 10.1002/cpt.1104. Epub 2018 Jun 19.
325. Witkowski M, Wilkinson L, Webb N, Weids A, Glah D, Vrazic H. A Systematic Literature Review and Network Meta-Analysis Comparing Once-Weekly Semaglutide with Other GLP-1 Receptor Agonists in Patients with Type 2 Diabetes Previously Receiving Basal Insulin Diabetes Ther. 2018 Jun;9(3):1233-1251. doi: 10.1007/s13300-018-0428-y. Epub 2018 Apr 30.
326. Lv S, Yu J, Xu X. A comparison of effectiveness among frequent treatments of recurrent spontaneous abortion: A Bayesian network meta-analysis Am J Reprod Immunol. 2018 Jul;80(1):e12856. doi: 10.1111/aji.12856. Epub 2018 Apr 30.
327. Iocca O, Farcomeni A, Di Rocco A, Di Maio P, Golusinski P, Pardiñas López S, Savo A, Pellini R, Spriano G. Locally advanced squamous cell carcinoma of the head and neck: A systematic review and Bayesian network meta-analysis of the currently available treatment options Oral Oncol. 2018 May;80:40-51. doi: 10.1016/j.oraloncology.2018.03.001. Epub 2018 Mar 27.
328. Zhang Y, Lin X, Lu F. Current treatment of ocular toxoplasmosis in immunocompetent patients: a network meta-analysis Acta Trop. 2018 Sep;185:52-62. doi: 10.1016/j.actatropica.2018.04.026. Epub 2018 Apr 25.
329. Kelleher C, Hakimi Z, Zur R, Siddiqui E, Maman K, Aballéa S, Nazir J, Chapple C. Efficacy and Tolerability of Mirabegron Compared with Antimuscarinic Monotherapy or Combination Therapies for Overactive Bladder: A Systematic Review and Network Meta-analysis Eur Urol. 2018 Sep;74(3):324-333. doi: 10.1016/j.eururo.2018.03.020. Epub 2018 Apr 23.
330. Bukhsh A, Khan TM, Lee SWH, Lee LH, Chan KG, Goh BH. Efficacy of Pharmacist Based Diabetes Educational Interventions on Clinical Outcomes of Adults With Type 2 Diabetes Mellitus: A Network Meta-Analysis Front Pharmacol. 2018 Apr 10;9:339. doi: 10.3389/fphar.2018.00339. eCollection 2018.
331. Jevsevar DS, Shores PB, Mullen K, Schulte DM, Brown GA, Cummins DS. Mixed Treatment Comparisons for Nonsurgical Treatment of Knee Osteoarthritis: A Network Meta-analysis J Am Acad Orthop Surg. 2018 May 1;26(9):325-336. doi: 10.5435/JAAOS-D-17-00318.
332. Rao F, Yang J, Gong C, Huang R, Wang Q, Shen J. Systematic review of preservation solutions for allografts for liver transplantation based on a network meta-analysis Int J Surg. 2018 Jun;54(Pt A):1-6. doi: 10.1016/j.ijsu.2018.04.024. Epub 2018 Apr 21.
333. Kim WH, Hur M, Park SK, Jung DE, Kang P, Yoo S, Bahk JH. Pharmacological interventions for protecting renal function after cardiac surgery: a Bayesian network meta-analysis of comparative effectiveness Anaesthesia. 2018 Aug;73(8):1019-1031. doi: 10.1111/anae.14227. Epub 2018 Apr 16.
334. Bruderer-Hofstetter M, Rausch-Osthoff AK, Meichtry A, Münzer T, Niedermann K. Effective multicomponent interventions in comparison to active control and no interventions on physical capacity, cognitive function and instrumental activities of daily living in elderly people with and without mild impaired cognition - A systematic review and network meta-analysis Ageing Res Rev. 2018 Aug;45:1-14. doi: 10.1016/j.arr.2018.04.002. Epub 2018 Apr 18.
335. Zheng SL, Roddick AJ, Aghar-Jaffar R, Shun-Shin MJ, Francis D, Oliver N, Meeran K. Association Between Use of Sodium-Glucose Cotransporter 2 Inhibitors, Glucagon-like Peptide 1 Agonists, and Dipeptidyl Peptidase 4 Inhibitors With All-Cause Mortality in Patients With Type 2 Diabetes: A Systematic Review and Meta-analysis JAMA. 2018 Apr 17;319(15):1580-1591. doi: 10.1001/jama.2018.3024.
336. Witkowski M, Wilkinson L, Webb N, Weids A, Glah D, Vrazic H. A Systematic Literature Review and Network Meta-Analysis Comparing Once-Weekly Semaglutide with Other GLP-1 Receptor Agonists in Patients with Type 2 Diabetes Previously Receiving 1-2 Oral Anti-Diabetic Drugs Diabetes Ther. 2018 Jun;9(3):1149-1167. doi: 10.1007/s13300-018-0424-2. Epub 2018 Apr 19.
337. Bazerbachi F, Haffar S, Sawas T, Vargas EJ, Kaur RJ, Wang Z, Prokop LJ, Murad MH, Abu Dayyeh BK. Fluid-Filled Versus Gas-Filled Intragastric Balloons as Obesity Interventions: a Network Meta-analysis of Randomized Trials Obes Surg. 2018 Sep;28(9):2617-2625. doi: 10.1007/s11695-018-3227-7.
338. Dorjee P, Long ZW. A mixed treatment comparison of toxicity of gemcitabine combined with different targeted drugs in the treatment of advanced or metastatic pancreatic cancer Cancer Biol Ther. 2018 Jun 3;19(6):497-506. doi: 10.1080/15384047.2018.1433503. Epub 2018 Apr 16.
339. Wallis CJD, Klaassen Z, Bhindi B, Ye XY, Chandrasekar T, Farrell AM, Goldberg H, Boorjian SA, Leibovich B, Kulkarni GS, Shah PS, Bjarnason GA, Heng DYC, Satkunasivam R, Finelli A. First-line Systemic Therapy for Metastatic Renal Cell Carcinoma: A Systematic Review and Network Meta-analysis Eur Urol. 2018 Sep;74(3):309-321. doi: 10.1016/j.eururo.2018.03.036. Epub 2018 Apr 13.
340. Belletti A, Benedetto U, Putzu A, Martino EA, Biondi-Zoccai G, Angelini GD, Zangrillo A, Landoni G. Vasopressors During Cardiopulmonary Resuscitation. A Network Meta-Analysis of Randomized Trials Crit Care Med. 2018 May;46(5):e443-e451. doi: 10.1097/CCM.0000000000003049.
341. Zhang X, Zhang Z, Wen J, Lu J, Sun Y, Sang D. The effectiveness of therapeutic strategies for patients with radiculopathy: A network meta-analysis Mol Pain. 2018 Jan-Dec;14:1744806918768972. doi: 10.1177/1744806918768972.
342. Ciliberto D, Staropoli N, Caglioti F, Chiellino S, Ierardi A, Ingargiola R, Botta C, Arbitrio M, Correale P, Tassone P, Tagliaferri P. The best strategy for RAS wild-type metastatic colorectal cancer patients in first-line treatment: A classic and Bayesian meta-analysis Crit Rev Oncol Hematol. 2018 May;125:69-77. doi: 10.1016/j.critrevonc.2018.03.003. Epub 2018 Mar 9.
343. Tian J, Luo Y, Wu H, Long H, Zhao M, Lu Q. Risk of adverse events from different drugs for SLE: a systematic review and network meta-analysis Lupus Sci Med. 2018 Mar 9;5(1):e000253. doi: 10.1136/lupus-2017-000253. eCollection 2018.
344. Kongpakwattana K, Sawangjit R, Tawankanjanachot I, Bell JS, Hilmer SN, Chaiyakunapruk N. Pharmacological treatments for alleviating agitation in dementia: a systematic review and network meta-analysis Br J Clin Pharmacol. 2018 Jul;84(7):1445-1456. doi: 10.1111/bcp.13604. Epub 2018 May 14.
345. Conde-Agudelo A, Romero R, Da Fonseca E, O'Brien JM, Cetingoz E, Creasy GW, Hassan SS, Erez O, Pacora P, Nicolaides KH. Vaginal progesterone is as effective as cervical cerclage to prevent preterm birth in women with a singleton gestation, previous spontaneous preterm birth, and a short cervix: updated indirect comparison meta-analysis Am J Obstet Gynecol. 2018 Jul;219(1):10-25. doi: 10.1016/j.ajog.2018.03.028. Epub 2018 Apr 7.
346. Dong Z, Gong J, Liao R, Xu S. Effectiveness of multiple therapeutic strategies in neovascular glaucoma patients: A PRISMA-compliant network meta-analysis Medicine (Baltimore). 2018 Apr;97(14):e9897. doi: 10.1097/MD.0000000000009897.
347. Armstrong AW, Betts KA, Signorovitch JE, Sundaram M, Li J, Ganguli AX, Wu EQ. Number needed to treat and costs per responder among biologic treatments for moderate-to-severe psoriasis: a network meta-analysis Curr Med Res Opin. 2018 Jul;34(7):1325-1333. doi: 10.1080/03007995.2018.1457516. Epub 2018 Apr 23.
348. Cai W, Gu Y, Cui H, Cao Y, Wang X, Yao Y, Wang M. The Efficacy and Safety of Mainstream Medications for Patients With cDMARD-Naïve Rheumatoid Arthritis: A Network Meta-Analysis Front Pharmacol. 2018 Mar 21;9:138. doi: 10.3389/fphar.2018.00138. eCollection 2018.
349. Liu S, Wu JR, Zhang D, Wang KH, Zhang B, Zhang XM, Tan D, Duan XJ, Cui YY, Liu XK. Comparative efficacy of Chinese herbal injections for treating acute cerebral infarction: a network meta-analysis of randomized controlled trials BMC Complement Altern Med. 2018 Apr 3;18(1):120. doi: 10.1186/s12906-018-2178-9.
350. Vuong NL, Elshafay A, Thao LP, Abdalla AR, Mohyeldin IA, Elsabaa K, Omran ES, Yu F, Hirayama K, Huy NT. Efficacy of treatments in primary spontaneous pneumothorax: A systematic review and network meta-analysis of randomized clinical trials Respir Med. 2018 Apr;137:152-166. doi: 10.1016/j.rmed.2018.03.009. Epub 2018 Mar 6.
351. Zhang JJ, Liu X. Aspirin plus dipyridamole has the highest surface under the cumulative ranking curves (SUCRA) values in terms of mortality, intracranial hemorrhage, and adverse event rate among 7 drug therapies in the treatment of cerebral infarction Medicine (Baltimore). 2018 Mar;97(13):e0123. doi: 10.1097/MD.0000000000010123.
352. Mitra S, Florez ID, Tamayo ME, Mbuagbaw L, Vanniyasingam T, Veroniki AA, Zea AM, Zhang Y, Sadeghirad B, Thabane L. Association of Placebo, Indomethacin, Ibuprofen, and Acetaminophen With Closure of Hemodynamically Significant Patent Ductus Arteriosus in Preterm Infants: A Systematic Review and Meta-analysis JAMA. 2018 Mar 27;319(12):1221-1238. doi: 10.1001/jama.2018.1896.
353. Fu X, Wang Y, Wang C, Wu H, Li J, Li M, Ma Q, Yang W. A mixed treatment comparison on efficacy and safety of treatments for spasticity caused by multiple sclerosis: a systematic review and network meta-analysis Clin Rehabil. 2018 Jun;32(6):713-721. doi: 10.1177/0269215517745348. Epub 2018 Mar 27.
354. Cai J, Huang X, Zheng Z, Lin Q, Peng M, Shen D. Comparative efficacy of individual renin-angiotensin system inhibitors on major renal outcomes in diabetic kidney disease: a network meta-analysis Nephrol Dial Transplant. 2018 Nov 1;33(11):1968-1976. doi: 10.1093/ndt/gfy001.
355. Yang Y, Qiu S, Tang X, Li XR, Deng LH, Wei Q, Fu P. Efficacy and Safety of Different Bisphosphonates for Bone Loss Prevention in Kidney Transplant Recipients: A Network Meta-Analysis of Randomized Controlled Trials Chin Med J (Engl). 2018 Apr 5;131(7):818-828. doi: 10.4103/0366-6999.228252.
356. Khan SU, Talluri S, Riaz H, Rahman H, Nasir F, Bin Riaz I, Sattur S, Ahmed H, Kaluski E, Krasuski R. A Bayesian network meta-analysis of PCSK9 inhibitors, statins and ezetimibe with or without statins for cardiovascular outcomes Eur J Prev Cardiol. 2018 May;25(8):844-853. doi: 10.1177/2047487318766612. Epub 2018 Mar 23.
357. Zhang D, Wu J, Wang K, Duan X, Liu S, Zhang B. Which are the best Chinese herbal injections combined with XELOX regimen for gastric cancer?: A PRISMA-compliant network meta-analysis Medicine (Baltimore). 2018 Mar;97(12):e0127. doi: 10.1097/MD.0000000000010127.
358. Gomez-Ospina JC, Zapata-Copete JA, Bejarano M, García-Perdomo HA. Antibiotic Prophylaxis in Elective Laparoscopic Cholecystectomy: a Systematic Review and Network Meta-Analysis J Gastrointest Surg. 2018 Jul;22(7):1193-1203. doi: 10.1007/s11605-018-3739-4. Epub 2018 Mar 19.
359. Zhao Y, Huang H, Chen C, Liu H, Liu H, Su F, Bi J, Lam TB, Li J, Lin T, Huang J. Efficacy and safety of different interventions in castration resistant prostate cancer progressing after docetaxel-based chemotherapy: Bayesian network analysis of randomized controlled trials J Cancer. 2018 Jan 11;9(4):690-701. doi: 10.7150/jca.22365. eCollection 2018.
360. De Mello RA, Escriu C, Castelo-Branco P, Cabral PL, Mountzios G, Lopes GL, Madureira P. Comparative outcome assessment of epidermal growth factor receptor tyrosine kinase inhibitors for the treatment of advanced non-small-cell lung cancer: a network meta-analysis Oncotarget. 2017 Dec 23;9(14):11805-11815. doi: 10.18632/oncotarget.23668. eCollection 2018 Feb 20.
361. Cheng P, Tan L, Ning P, Li L, Gao Y, Wu Y, Schwebel DC, Chu H, Yin H, Hu G. Comparative Effectiveness of Published Interventions for Elderly Fall Prevention: A Systematic Review and Network Meta-Analysis Int J Environ Res Public Health. 2018 Mar 12;15(3):498. doi: 10.3390/ijerph15030498.
362. Zhou L, Huang J, Chen C. Most effective pain-control procedure for open liver surgery: a network meta-analysis ANZ J Surg. 2018 Dec;88(12):1236-1242. doi: 10.1111/ans.14456. Epub 2018 Mar 13.
363. Zaccardi F, Kunutsor SK, Seidu S, Davies MJ, Khunti K. Is the lower risk of venous thromboembolism with statins related to low-density-lipoprotein reduction? A network meta-analysis and meta-regression of randomised controlled trials Atherosclerosis. 2018 Apr;271:223-231. doi: 10.1016/j.atherosclerosis.2018.02.035. Epub 2018 Mar 2.
364. Gao YN, Wu YC, Lin SY, Chang JZ, Tu YK. Short-term efficacy of minimally invasive treatments for adult obstructive sleep apnea: A systematic review and network meta-analysis of randomized controlled trials J Formos Med Assoc. 2019 Apr;118(4):750-765. doi: 10.1016/j.jfma.2018.02.008. Epub 2018 Mar 6.
365. Barrionuevo P, Nabhan M, Altayar O, Wang Z, Erwin PJ, Asi N, Martin KA, Murad MH. Treatment Options for Hirsutism: A Systematic Review and Network Meta-Analysis J Clin Endocrinol Metab. 2018 Apr 1;103(4):1258-1264. doi: 10.1210/jc.2017-02052.
366. Kotsakis GA, Lian Q, Ioannou AL, Michalowicz BS, John MT, Chu H. A network meta-analysis of interproximal oral hygiene methods in the reduction of clinical indices of inflammation J Periodontol. 2018 May;89(5):558-570. doi: 10.1002/JPER.17-0368.
367. Cai J, Zhao C, Du Y, Zhang Y, Zhao M, Zhao Q. Comparative efficacy and tolerability of probiotics for antibiotic-associated diarrhea: Systematic review with network meta-analysis United European Gastroenterol J. 2018 Mar;6(2):169-180. doi: 10.1177/2050640617736987. Epub 2017 Oct 4.
368. Yoon JH, Min SH, Ahn CH, Cho YM, Hahn S. Comparison of non-insulin antidiabetic agents as an add-on drug to insulin therapy in type 2 diabetes: a network meta-analysis Sci Rep. 2018 Mar 6;8(1):4095. doi: 10.1038/s41598-018-22443-1.
369. Xie D, Liao Y, Yue J, Zhang C, Wang Y, Deng C, Chen L. Effects of five types of selenium supplementation for treatment of Kashin-Beck disease in children: a systematic review and network meta-analysis BMJ Open. 2018 Mar 6;8(3):e017883. doi: 10.1136/bmjopen-2017-017883.
370. Öberg S, Andresen K, Klausen TW, Rosenberg J. Chronic pain after mesh versus nonmesh repair of inguinal hernias: A systematic review and a network meta-analysis of randomized controlled trials Surgery. 2018 May;163(5):1151-1159. doi: 10.1016/j.surg.2017.12.017. Epub 2018 Mar 13.
371. Maiese EM, Ainsworth C, Le Moine JG, Ahdesmäki O, Bell J, Hawe E. Comparative Efficacy of Treatments for Previously Treated Multiple Myeloma: A Systematic Literature Review and Network Meta-analysis Clin Ther. 2018 Mar;40(3):480-494.e23. doi: 10.1016/j.clinthera.2018.01.014. Epub 2018 Feb 28.
372. Schmidt E, Lister J, Neumann M, Wiecek W, Fu S, Vataire AL, Sostar J, Huang S, Marteau F. Cabozantinib Versus Standard-of-Care Comparators in the Treatment of Advanced/Metastatic Renal Cell Carcinoma in Treatment-naïve Patients: a Systematic Review and Network Meta-Analysis Target Oncol. 2018 Apr;13(2):205-216. doi: 10.1007/s11523-018-0559-0.
373. Sridharan K, Sivaramakrishnan G. Pharmacological interventions for reducing pain related to immunization or intramuscular injection in children: A mixed treatment comparison network meta-analysis of randomized controlled clinical trials J Child Health Care. 2018 Sep;22(3):393-405. doi: 10.1177/1367493518760735. Epub 2018 Feb 27.
374. Pulikkotil SJ, Nagendrababu V, Veettil SK, Jinatongthai P, Setzer FC. Effect of oral premedication on the anaesthetic efficacy of inferior alveolar nerve block in patients with irreversible pulpitis - A systematic review and network meta-analysis of randomized controlled trials Int Endod J. 2018 Sep;51(9):989-1004. doi: 10.1111/iej.12912. Epub 2018 Mar 24.
375. Dawoud D, O'Mahony R, Wonderling D, Cobb J, Higgins B, Amiel SA. Basal Insulin Regimens for Adults with Type 1 Diabetes Mellitus: A Systematic Review and Network Meta-Analysis Value Health. 2018 Feb;21(2):176-184. doi: 10.1016/j.jval.2017.04.024. Epub 2017 Jun 20.
376. Cipriani A, Furukawa TA, Salanti G, Chaimani A, Atkinson LZ, Ogawa Y, Leucht S, Ruhe HG, Turner EH, Higgins JPT, Egger M, Takeshima N, Hayasaka Y, Imai H, Shinohara K, Tajika A, Ioannidis JPA, Geddes JR. Comparative efficacy and acceptability of 21 antidepressant drugs for the acute treatment of adults with major depressive disorder: a systematic review and network meta-analysis Lancet. 2018 Apr 7;391(10128):1357-1366. doi: 10.1016/S0140-6736(17)32802-7. Epub 2018 Feb 21.
377. Freeman SC, Scott NW, Powell R, Johnston M, Sutton AJ, Cooper NJ. Component network meta-analysis identifies the most effective components of psychological preparation for adults undergoing surgery under general anesthesia J Clin Epidemiol. 2018 Jun;98:105-116. doi: 10.1016/j.jclinepi.2018.02.012. Epub 2018 Feb 21.
378. Zeng L, Tian J, Song F, Li W, Jiang L, Gui G, Zhang Y, Ge L, Shi J, Sun X, Mu D, Zhang L. Corticosteroids for the prevention of bronchopulmonary dysplasia in preterm infants: a network meta-analysis Arch Dis Child Fetal Neonatal Ed. 2018 Nov;103(6):F506-F511. doi: 10.1136/archdischild-2017-313759. Epub 2018 Feb 23.
379. Sridharan K, Sivaramakrishnan G. Pharmacological interventions for treating sialorrhea associated with neurological disorders: A mixed treatment network meta-analysis of randomized controlled trials J Clin Neurosci. 2018 May;51:12-17. doi: 10.1016/j.jocn.2018.02.011. Epub 2018 Feb 21.
380. Lai CC, Tu YK, Wang TG, Huang YT, Chien KL. Effects of resistance training, endurance training and whole-body vibration on lean body mass, muscle strength and physical performance in older people: a systematic review and network meta-analysis Age Ageing. 2018 May 1;47(3):367-373. doi: 10.1093/ageing/afy009.
381. Siontis GC, Mavridis D, Greenwood JP, Coles B, Nikolakopoulou A, Jüni P, Salanti G, Windecker S. Outcomes of non-invasive diagnostic modalities for the detection of coronary artery disease: network meta-analysis of diagnostic randomised controlled trials BMJ. 2018 Feb 21;360:k504. doi: 10.1136/bmj.k504.
382. Kim S, Hwang J, Kim MJ, Lim JY, Lee WH, Choi JE. SYSTEMATIC REVIEW WITH NETWORK META-ANALYSIS OF RANDOMIZED CONTROLLED TRIALS OF ROTATOR CUFF TEAR TREATMENT Int J Technol Assess Health Care. 2018 Feb 22:1-9. doi: 10.1017/S0266462317004500. Online ahead of print.
383. Zhu X, Wu D, Sang L, Wang Y, Shen Y, Zhuang X, Chu M, Jiang L. Comparative effectiveness of glucosamine, chondroitin, acetaminophen or celecoxib for the treatment of knee and/or hip osteoarthritis: a network meta-analysis Clin Exp Rheumatol. 2018 Jul-Aug;36(4):595-602. Epub 2018 Jan 31.
384. Padilha SCOS, Virtuoso S, Tonin FS, Borba HHL, Pontarolo R. Efficacy and safety of drugs for attention deficit hyperactivity disorder in children and adolescents: a network meta-analysis Eur Child Adolesc Psychiatry. 2018 Oct;27(10):1335-1345. doi: 10.1007/s00787-018-1125-0. Epub 2018 Feb 19.
385. Dimopoulos MA, Kaufman JL, White D, Cook G, Rizzo M, Xu Y, Fahrbach K, Gaudig M, Slavcev M, Dearden L, Lam A. A Comparison of the Efficacy of Immunomodulatory-containing Regimens in Relapsed/Refractory Multiple Myeloma: A Network Meta-analysis Clin Lymphoma Myeloma Leuk. 2018 Mar;18(3):163-173.e6. doi: 10.1016/j.clml.2017.12.011. Epub 2018 Jan 5.
386. Zhao TT, Xu H, Xu HM, Wang ZN, Xu YY, Song YX, Yin SC, Liu XY, Miao ZF. The efficacy and safety of targeted therapy with or without chemotherapy in advanced gastric cancer treatment: a network meta-analysis of well-designed randomized controlled trials Gastric Cancer. 2018 May;21(3):361-371. doi: 10.1007/s10120-018-0813-2. Epub 2018 Feb 17.
387. Morabito A. Second-line treatment for advanced NSCLC without actionable mutations: is immunotherapy the 'panacea' for all patients? BMC Med. 2018 Feb 16;16(1):24. doi: 10.1186/s12916-018-1011-0.
388. Lv J, Zhou D, Wang Y, Zhao J, Chen Z, Zhang J, Di T, Hu J, Li B, Li P, Huang F. Quantitative evaluation to efficacy and safety of therapies for psoriasis: A network meta-analysis Mol Pain. 2018 Jan-Dec;14:1744806918762205. doi: 10.1177/1744806918762205. Epub 2018 Feb 15.
389. Chan KKW, Saluja R, Delos Santos K, Lien K, Shah K, Cramarossa G, Zhu X, Wong RKS. Neoadjuvant treatments for locally advanced, resectable esophageal cancer: A network meta-analysis Int J Cancer. 2018 Jul 15;143(2):430-437. doi: 10.1002/ijc.31312. Epub 2018 Mar 8.
390. Loos AM, Liu S, Segel C, Ollendorf DA, Pearson SD, Linder JA. Comparative effectiveness of targeted immunomodulators for the treatment of moderate-to-severe plaque psoriasis: A systematic review and network meta-analysis J Am Acad Dermatol. 2018 Jul;79(1):135-144.e7. doi: 10.1016/j.jaad.2018.02.027. Epub 2018 Feb 10.
391. Jung SY, Jang EJ, Nam SW, Kwon HH, Im SG, Kim D, Cho SK, Kim D, Sung YK. Comparative effectiveness of oral pharmacologic interventions for knee osteoarthritis: A network meta-analysis Mod Rheumatol. 2018 Nov;28(6):1021-1028. doi: 10.1080/14397595.2018.1439694. Epub 2018 Mar 1.
392. Zhang GX, Zhang YY, Zhang XX, Wang PQ, Liu J, Liu Q, Wang Z. Different network pharmacology mechanisms of Danshen-based Fangjis in the treatment of stable angina Acta Pharmacol Sin. 2018 Jun;39(6):952-960. doi: 10.1038/aps.2017.191. Epub 2018 Feb 8.
393. Bonsu KO, Arunmanakul P, Chaiyakunapruk N. Pharmacological treatments for heart failure with preserved ejection fraction-a systematic review and indirect comparison Heart Fail Rev. 2018 Mar;23(2):147-156. doi: 10.1007/s10741-018-9679-y.
394. Pasquali S, Hadjinicolaou AV, Chiarion Sileni V, Rossi CR, Mocellin S. Systemic treatments for metastatic cutaneous melanoma Cochrane Database Syst Rev. 2018 Feb 6;2(2):CD011123. doi: 10.1002/14651858.CD011123.pub2.
395. Ma WQ, Zhao Y, Wang Y, Han XQ, Zhu Y, Liu NF. Comparative efficacy of pharmacological interventions for contrast-induced nephropathy prevention after coronary angiography: a network meta-analysis from randomized trials Int Urol Nephrol. 2018 Jun;50(6):1085-1095. doi: 10.1007/s11255-018-1814-0. Epub 2018 Feb 5.
396. Bae SC, Lee YH. Comparison of the efficacy and tolerability of tocilizumab, sarilumab, and sirukumab in patients with active rheumatoid arthritis: a Bayesian network meta-analysis of randomized controlled trials Clin Rheumatol. 2018 Jun;37(6):1471-1479. doi: 10.1007/s10067-018-4006-5. Epub 2018 Feb 5.
397. Mina GS, Watti H, Soliman D, Shewale A, Atkins J, Reddy P, Dominic P. Long term outcomes of new generation drug eluting stents versus coronary artery bypass grafting for multivessel and/or left main coronary artery disease. A Bayesian network meta-analysis of randomized controlled trials Cardiovasc Revasc Med. 2018 Sep;19(6):671-678. doi: 10.1016/j.carrev.2018.01.003. Epub 2018 Jan 5.
398. Gutiérrez-Castrellón P, Indrio F, Bolio-Galvis A, Jiménez-Gutiérrez C, Jimenez-Escobar I, López-Velázquez G. Efficacy of Lactobacillus reuteri DSM 17938 for infantile colic: Systematic review with network meta-analysis Medicine (Baltimore). 2017 Dec;96(51):e9375. doi: 10.1097/MD.0000000000009375.
399. Guo C, Sun X, Wang X, Guo Q, Chen D. Network Meta-Analysis Comparing the Efficacy of Therapeutic Treatments for Bronchiolitis in Children JPEN J Parenter Enteral Nutr. 2018 Jan;42(1):186-195. doi: 10.1002/jpen.1030. Epub 2017 Nov 3.
400. Wang KH, Wu JR, Zhang D, Duan XJ, Ni MW. Comparative efficacy of Chinese herbal injections for treating chronic heart failure: a network meta-analysis BMC Complement Altern Med. 2018 Jan 31;18(1):41. doi: 10.1186/s12906-018-2090-3.
401. Ma DN, Zhang XQ, Ying J, Chen ZJ, Li LX. Efficacy and safety of 9 nonoperative regimens for the treatment of spinal cord injury: A network meta-analysis Medicine (Baltimore). 2017 Nov;96(47):e8679. doi: 10.1097/MD.0000000000008679.
402. Zhang W, Zhang J, Yang B, Wu K, Lin H, Wang Y, Zhou L, Wang H, Zeng C, Chen X, Wang Z, Zhu J, Songming C. Effectiveness of oral hydration in preventing contrast-induced acute kidney injury in patients undergoing coronary angiography or intervention: a pairwise and network meta-analysis Coron Artery Dis. 2018 Jun;29(4):286-293. doi: 10.1097/MCA.0000000000000607.
403. Shah ED, Kim HM, Schoenfeld P. Efficacy and Tolerability of Guanylate Cyclase-C Agonists for Irritable Bowel Syndrome with Constipation and Chronic Idiopathic Constipation: A Systematic Review and Meta-Analysis Am J Gastroenterol. 2018 Mar;113(3):329-338. doi: 10.1038/ajg.2017.495. Epub 2018 Jan 30.
404. Tang H, Li G, Zhao Y, Wang F, Gower EW, Shi L, Wang T. Comparisons of diabetic retinopathy events associated with glucose-lowering drugs in patients with type 2 diabetes mellitus: A network meta-analysis Diabetes Obes Metab. 2018 May;20(5):1262-1279. doi: 10.1111/dom.13232. Epub 2018 Feb 23.
405. Pompoli A, Furukawa TA, Efthimiou O, Imai H, Tajika A, Salanti G. Dismantling cognitive-behaviour therapy for panic disorder: a systematic review and component network meta-analysis Psychol Med. 2018 Sep;48(12):1945-1953. doi: 10.1017/S0033291717003919. Epub 2018 Jan 25.
406. Song D, He A, Xu R, Xiu X, Wei Y. Efficacy of Pain Relief in Different Postherpetic Neuralgia Therapies: A Network Meta-Analysis Pain Physician. 2018 Jan;21(1):19-32.
407. Li BD, Cui JJ, Song J, Qi C, Ma PF, Wang YR, Bai J. Comparison of the Efficacy of Different Drugs on Non-Motor Symptoms of Parkinson's Disease: a Network Meta-Analysis Cell Physiol Biochem. 2018;45(1):119-130. doi: 10.1159/000486252. Epub 2018 Jan 15.
408. Benedetto U, Taggart DP, Sousa-Uva M, Biondi-Zoccai G, Di Franco A, Ohmes LB, Rahouma M, Kamel M, Caputo M, Girardi LN, Angelini GD, Gaudino M. New-generation stents compared with coronary bypass surgery for unprotected left main disease: A word of caution J Thorac Cardiovasc Surg. 2018 May;155(5):2013-2019.e16. doi: 10.1016/j.jtcvs.2017.11.066. Epub 2018 Jan 12.
409. Wang R, Dasgupta A, Ward MM. Comparative Efficacy of Tumor Necrosis Factor-α Inhibitors in Ankylosing Spondylitis: A Systematic Review and Bayesian Network Metaanalysis J Rheumatol. 2018 Apr;45(4):481-490. doi: 10.3899/jrheum.170224. Epub 2018 Jan 15.
410. Sridharan K, Sivaramakrishnan G. Efficacy and safety of alpha blockers in medical expulsive therapy for ureteral stones: a mixed treatment network meta-analysis and trial sequential analysis of randomized controlled clinical trials Expert Rev Clin Pharmacol. 2018 Mar;11(3):291-307. doi: 10.1080/17512433.2018.1424537. Epub 2018 Jan 19.
411. Zhang Z, Wu Y, Sun G, Zhang J, Li J, Qiu C, Zheng X, Wang B, Yang L, Wang X. Bayesian network meta-analysis: Efficacy of air insufflation, CO(2) insufflation, water exchange, and water immersion in colonoscopy Dig Endosc. 2018 May;30(3):321-331. doi: 10.1111/den.13012. Epub 2018 Feb 5.
412. Koifman E, Lipinski MJ, Buchanan K, Yu Kang W, Escarcega RO, Waksman R, Bernardo NL. Comparison of treatment strategies for femoro-popliteal disease: A network meta-analysis Catheter Cardiovasc Interv. 2018 Jun;91(7):1320-1328. doi: 10.1002/ccd.27484. Epub 2018 Jan 14.
413. Song P, Wen Y, Huang C, Wang W, Yuan N, Lu Y, Wang Q, Zhang T, Wen J. The efficacy and safety comparison of surgical treatments for stress urinary incontinence: A network meta-analysis Neurourol Urodyn. 2018 Apr;37(4):1199-1211. doi: 10.1002/nau.23468. Epub 2018 Jan 13.
414. Zhang Z, Zhang Y, Chen G, Hong S, Yang Y, Fang W, Luo F, Chen X, Ma Y, Zhao Y, Zhan J, Xue C, Hou X, Zhou T, Ma S, Gao F, Huang Y, Chen L, Zhou N, Zhao H, Zhang L. Olanzapine-Based Triple Regimens Versus Neurokinin-1 Receptor Antagonist-Based Triple Regimens in Preventing Chemotherapy-Induced Nausea and Vomiting Associated with Highly Emetogenic Chemotherapy: A Network Meta-Analysis Oncologist. 2018 May;23(5):603-616. doi: 10.1634/theoncologist.2017-0378. Epub 2018 Jan 12.
415. Sawyer L, Fotheringham I, Wright E, Yasmeen N, Gibbons C, Holmen Møller A. The comparative efficacy of brodalumab in patients with moderate-to-severe psoriasis: a systematic literature review and network meta-analysis J Dermatolog Treat. 2018 Sep;29(6):557-568. doi: 10.1080/09546634.2018.1427205. Epub 2018 Jan 29.
416. Niu X, Zhang J, Bai M, Peng Y, Sun S, Zhang Z. Effect of intracoronary agents on the no-reflow phenomenon during primary percutaneous coronary intervention in patients with ST-elevation myocardial infarction: a network meta-analysis BMC Cardiovasc Disord. 2018 Jan 10;18(1):3. doi: 10.1186/s12872-017-0722-z.
417. Wu S, Cipriani A, Yang Z, Yang J, Cai T, Xu Y, Quan X, Zhang Y, Chai S, Sun F, Zhan S. The cardiovascular effect of incretin-based therapies among type 2 diabetes: a systematic review and network meta-analysis Expert Opin Drug Saf. 2018 Mar;17(3):243-249. doi: 10.1080/14740338.2018.1424826. Epub 2018 Jan 10.
418. Richter JE, Kumar A, Lipka S, Miladinovic B, Velanovich V. Efficacy of Laparoscopic Nissen Fundoplication vs Transoral Incisionless Fundoplication or Proton Pump Inhibitors in Patients With Gastroesophageal Reflux Disease: A Systematic Review and Network Meta-analysis Gastroenterology. 2018 Apr;154(5):1298-1308.e7. doi: 10.1053/j.gastro.2017.12.021. Epub 2018 Jan 3.
419. Khera R, Pandey A, Chandar AK, Murad MH, Prokop LJ, Neeland IJ, Berry JD, Camilleri M, Singh S. Effects of Weight-Loss Medications on Cardiometabolic Risk Profiles: A Systematic Review and Network Meta-analysis Gastroenterology. 2018 Apr;154(5):1309-1319.e7. doi: 10.1053/j.gastro.2017.12.024. Epub 2018 Jan 3.
420. Dhakal B, Szabo A, Chhabra S, Hamadani M, D'Souza A, Usmani SZ, Sieracki R, Gyawali B, Jackson JL, Asimakopoulos F, Hari PN. Autologous Transplantation for Newly Diagnosed Multiple Myeloma in the Era of Novel Agent Induction: A Systematic Review and Meta-analysis JAMA Oncol. 2018 Mar 1;4(3):343-350. doi: 10.1001/jamaoncol.2017.4600.
421. Botta C, Ciliberto D, Rossi M, Staropoli N, Cucè M, Galeano T, Tagliaferri P, Tassone P. Network meta-analysis of randomized trials in multiple myeloma: efficacy and safety in relapsed/refractory patients Blood Adv. 2017 Feb 27;1(7):455-466. doi: 10.1182/bloodadvances.2016003905. eCollection 2017 Feb 28.
422. Mo JJ, Li JY, Yang Z, Liu Z, Feng JS. Efficacy and safety of anti-amyloid-β immunotherapy for Alzheimer's disease: a systematic review and network meta-analysis Ann Clin Transl Neurol. 2017 Oct 30;4(12):931-942. doi: 10.1002/acn3.469. eCollection 2017 Dec.
423. Yu X, Zhang D, Chen X, Yang J, Shi L, Pang Q. Effectiveness of various hip preservation treatments for non-traumatic osteonecrosis of the femoral head: A network meta-analysis of randomized controlled trials J Orthop Sci. 2018 Mar;23(2):356-364. doi: 10.1016/j.jos.2017.12.004. Epub 2017 Dec 29.
424. Zhang Y, Hui F, Yang Y, Chu H, Qin X, Zhao M, Zhao Q. Can Kushen injection combined with TACE improve therapeutic efficacy and safety in patients with advanced HCC? a systematic review and network meta-analysis Oncotarget. 2017 Sep 15;8(63):107258-107272. doi: 10.18632/oncotarget.20921. eCollection 2017 Dec 5.
425. Tan PS, Aguiar P Jr, Haaland B, Lopes G. Comparative effectiveness of immune-checkpoint inhibitors for previously treated advanced non-small cell lung cancer - A systematic review and network meta-analysis of 3024 participants Lung Cancer. 2018 Jan;115:84-88. doi: 10.1016/j.lungcan.2017.11.017. Epub 2017 Nov 22.
426. Kawalec P, Holko P, Moćko P, Pilc A. Comparative effectiveness of abatacept, apremilast, secukinumab and ustekinumab treatment of psoriatic arthritis: a systematic review and network meta-analysis Rheumatol Int. 2018 Feb;38(2):189-201. doi: 10.1007/s00296-017-3919-7. Epub 2017 Dec 28.
427. Kang SH, Gogas BD, Jeon KH, Park JS, Lee W, Yoon CH, Suh JW, Hwang SS, Youn TJ, Chae IH, Kim HS. Long-term safety of bioresorbable scaffolds: insights from a network meta-analysis including 91 trials EuroIntervention. 2018 Mar 20;13(16):1904-1913. doi: 10.4244/EIJ-D-17-00646.
428. Fei Y, Tsoi MF, Kumana CR, Cheung TT, Cheung BMY. Network meta-analysis of cardiovascular outcomes in randomized controlled trials of new antidiabetic drugs Int J Cardiol. 2018 Mar 1;254:291-296. doi: 10.1016/j.ijcard.2017.12.039. Epub 2017 Dec 20.
429. Sbidian E, Chaimani A, Garcia-Doval I, Do G, Hua C, Mazaud C, Droitcourt C, Hughes C, Ingram JR, Naldi L, Chosidow O, Le Cleach L. Systemic pharmacological treatments for chronic plaque psoriasis: a network meta-analysis Cochrane Database Syst Rev. 2017 Dec 22;12(12):CD011535. doi: 10.1002/14651858.CD011535.pub2.
430. Rosati A, Ilvento L, Lucenteforte E, Pugi A, Crescioli G, McGreevy KS, Virgili G, Mugelli A, De Masi S, Guerrini R. Comparative efficacy of antiepileptic drugs in children and adolescents: A network meta-analysis Epilepsia. 2018 Feb;59(2):297-314. doi: 10.1111/epi.13981. Epub 2017 Dec 22.
431. Bedenne L, Le Malicot K, Drouillard A. Is it still time for meta-analyses in operable esophageal cancers, or rather for a change of paradigm? J Thorac Dis. 2017 Oct;9(10):3594-3597. doi: 10.21037/jtd.2017.09.35.
432. Rogliani P, Matera MG, Ora J, Cazzola M, Calzetta L. The impact of dual bronchodilation on cardiovascular serious adverse events and mortality in COPD: a quantitative synthesis Int J Chron Obstruct Pulmon Dis. 2017 Dec 5;12:3469-3485. doi: 10.2147/COPD.S146338. eCollection 2017.
433. Cipriani A, Williams T, Nikolakopoulou A, Salanti G, Chaimani A, Ipser J, Cowen PJ, Geddes JR, Stein DJ. Comparative efficacy and acceptability of pharmacological treatments for post-traumatic stress disorder in adults: a network meta-analysis Psychol Med. 2018 Sep;48(12):1975-1984. doi: 10.1017/S003329171700349X. Epub 2017 Dec 19.
434. Mhaskar R, Kumar A, Miladinovic B, Djulbegovic B. Bisphosphonates in multiple myeloma: an updated network meta-analysis Cochrane Database Syst Rev. 2017 Dec 18;12(12):CD003188. doi: 10.1002/14651858.CD003188.pub4.
435. Saber H, Rajah GB, Kherallah RY, Jadhav AP, Narayanan S. Comparison of the efficacy and safety of thrombectomy devices in acute stroke : a network meta-analysis of randomized trials J Neurointerv Surg. 2018 Aug;10(8):729-734. doi: 10.1136/neurintsurg-2017-013544. Epub 2017 Dec 15.
436. Wu DM, Wang YJ, Fan SH, Zhuang J, Zhang ZF, Shan Q, Han XR, Wen X, Li MQ, Hu B, Sun CH, Bao YX, Xiao HJ, Yang L, Lu J, Zheng YL. Network meta-analysis of the efficacy of first-line chemotherapy regimens in patients with advanced colorectal cancer Oncotarget. 2017 Oct 31;8(59):100668-100677. doi: 10.18632/oncotarget.22177. eCollection 2017 Nov 21.
437. Xie H, Yu H, Tian S, Yang X, Wang X, Yang Z, Wang H, Guo Z. What is the best combination treatment with transarterial chemoembolization of unresectable hepatocellular carcinoma? a systematic review and network meta-analysis Oncotarget. 2017 Aug 10;8(59):100508-100523. doi: 10.18632/oncotarget.20119. eCollection 2017 Nov 21.
438. Han SY, Hong ZY, Xie YH, Zhao Y, Xu X. Therapeutic effect of Chinese herbal medicines for post stroke recovery: A traditional and network meta-analysis Medicine (Baltimore). 2017 Dec;96(49):e8830. doi: 10.1097/MD.0000000000008830.
439. Guo WQ, Li L, Su Q, Dai WR, Ye ZL. Effect of Dipeptidyl Peptidase-4 Inhibitors on Heart Failure: A Network Meta-Analysis Value Health. 2017 Dec;20(10):1427-1430. doi: 10.1016/j.jval.2017.04.010. Epub 2017 May 29.
440. Wang KL, Chiu CC, Giugliano RP, Tan DS, Lin CY, Lai EY, Goto S, Chiang CE. Drug Class, Renal Elimination, and Outcomes of Direct Oral Anticoagulants in Asian Patients: A Meta-Analysis J Stroke Cerebrovasc Dis. 2018 Apr;27(4):857-864. doi: 10.1016/j.jstrokecerebrovasdis.2017.10.027. Epub 2017 Nov 26.
441. Zhang HL, Tan M, Qiu AM, Tao Z, Wang CH. Antibiotics for treatment of acute exacerbation of chronic obstructive pulmonary disease: a network meta-analysis BMC Pulm Med. 2017 Dec 12;17(1):196. doi: 10.1186/s12890-017-0541-0.
442. Wang Q, He Y, Shen J. The best surgical strategy for anal fistula based on a network meta-analysis Oncotarget. 2017 Oct 12;8(58):99075-99084. doi: 10.18632/oncotarget.21836. eCollection 2017 Nov 17.
443. Liu GF, Wang ZQ, Liu L, Zhang BT, Miao YY, Yu SN. A network meta-analysis on the short-term efficacy and adverse events of different anti-osteoporosis drugs for the treatment of postmenopausal osteoporosis J Cell Biochem. 2018 Jun;119(6):4469-4481. doi: 10.1002/jcb.26550. Epub 2018 Feb 28.
444. Peng H, Chen BB, Chen L, Chen YP, Liu X, Tang LL, Mao YP, Li WF, Zhang Y, Lin AH, Sun Y, Ma J. A network meta-analysis in comparing prophylactic treatments of radiotherapy-induced oral mucositis for patients with head and neck cancers receiving radiotherapy Oral Oncol. 2017 Dec;75:89-94. doi: 10.1016/j.oraloncology.2017.11.001. Epub 2017 Nov 8.
445. Wen J, Peng P, Chen P, Zeng L, Pan Q, Wei W, He J. Probiotics in 14-day triple therapy for Asian pediatric patients with Helicobacter pylori infection: a network meta-analysis Oncotarget. 2017 Oct 7;8(56):96409-96418. doi: 10.18632/oncotarget.21633. eCollection 2017 Nov 10.
446. Wei C, Wang S, Ye Z, Chen Z. Efficacy of targeted therapy for advanced renal cell carcinoma: a systematic review and meta-analysis of randomized controlled trials Int Braz J Urol. 2018 Mar-Apr;44(2):219-237. doi: 10.1590/S1677-5538.IBJU.2017.0315.
447. Xu Y, Fahrbach K, Dorman E, Baculea S, Côté S, Sanden SV, Diels J. Front-line treatment of patients with chronic lymphocytic leukemia: a systematic review and network meta-analysis J Comp Eff Res. 2018 May;7(5):421-441. doi: 10.2217/cer-2017-0086. Epub 2017 Dec 6.
448. Cai P, Hao J, Wang D, Xu J. Comparative efficacy of different chemotherapies for non-Hodgkin lymphoma: a network-meta analysis Oncotarget. 2017 Aug 24;8(53):91238-91247. doi: 10.18632/oncotarget.20437. eCollection 2017 Oct 31.
449. Yang J, Huang C, Wu S, Xu Y, Cai T, Chai S, Yang Z, Sun F, Zhan S. The effects of dipeptidyl peptidase-4 inhibitors on bone fracture among patients with type 2 diabetes mellitus: A network meta-analysis of randomized controlled trials PLoS One. 2017 Dec 5;12(12):e0187537. doi: 10.1371/journal.pone.0187537. eCollection 2017.
450. Zaccardi F, Dhalwani NN, Dales J, Mani H, Khunti K, Davies MJ, Webb DR. Comparison of glucose-lowering agents after dual therapy failure in type 2 diabetes: A systematic review and network meta-analysis of randomized controlled trials Diabetes Obes Metab. 2018 Apr;20(4):985-997. doi: 10.1111/dom.13185. Epub 2018 Jan 8.
451. Bonovas S, Lytras T, Nikolopoulos G, Peyrin-Biroulet L, Danese S. Systematic review with network meta-analysis: comparative assessment of tofacitinib and biological therapies for moderate-to-severe ulcerative colitis Aliment Pharmacol Ther. 2018 Feb;47(4):454-465. doi: 10.1111/apt.14449. Epub 2017 Dec 4.
452. Singh S, Fumery M, Sandborn WJ, Murad MH. Systematic review with network meta-analysis: first- and second-line pharmacotherapy for moderate-severe ulcerative colitis Aliment Pharmacol Ther. 2018 Jan;47(2):162-175. doi: 10.1111/apt.14422. Epub 2017 Dec 4.
453. Alhazzani W, Alshamsi F, Belley-Cote E, Heels-Ansdell D, Brignardello-Petersen R, Alquraini M, Perner A, Møller MH, Krag M, Almenawer S, Rochwerg B, Dionne J, Jaeschke R, Alshahrani M, Deane A, Perri D, Thebane L, Al-Omari A, Finfer S, Cook D, Guyatt G. Efficacy and safety of stress ulcer prophylaxis in critically ill patients: a network meta-analysis of randomized trials Intensive Care Med. 2018 Jan;44(1):1-11. doi: 10.1007/s00134-017-5005-8. Epub 2017 Dec 4.
454. Li-Na Z, Deng C, Hai-Jiao W, Da X, Ge T, Ling L. Indirect comparison of third-generation antiepileptic drugs as adjunctive treatment for uncontrolled focal epilepsy Epilepsy Res. 2018 Jan;139:60-72. doi: 10.1016/j.eplepsyres.2017.11.008. Epub 2017 Nov 26.
455. You R, Cao YS, Huang PY, Chen L, Yang Q, Liu YP, Zou X, Zhang YN, Jiang R, Zhang MX, Duan CY, Lin AH, Hong MH, Chen MY. The Changing Therapeutic Role of Chemo-radiotherapy for Loco-regionally Advanced Nasopharyngeal Carcinoma from Two/Three-Dimensional Radiotherapy to Intensity-Modulated Radiotherapy: A Network Meta-Analysis Theranostics. 2017 Oct 17;7(19):4825-4835. doi: 10.7150/thno.21815. eCollection 2017.
456. Ordóñez-Mena JM, McCarthy ND, Fanshawe TR. Comparative efficacy of drugs for treating giardiasis: a systematic update of the literature and network meta-analysis of randomized clinical trials J Antimicrob Chemother. 2018 Mar 1;73(3):596-606. doi: 10.1093/jac/dkx430.
457. Deng L, Sun X, Qiu S, Xiong Y, Li Y, Wang L, Wei Q, Wang D, Liu M. Interventions for management of post-stroke depression: A Bayesian network meta-analysis of 23 randomized controlled trials Sci Rep. 2017 Nov 28;7(1):16466. doi: 10.1038/s41598-017-16663-0.
458. El Rassy E, Aoun F, Sleilaty G, Kattan J, Banyurwabuke B, Zanaty M, Bakouny Z, Albisinni S, Peltier A, Roumeguere T. Network meta-analysis of second-line treatment in metastatic renal cell carcinoma: efficacy and safety Future Oncol. 2017 Dec;13(29):2709-2717. doi: 10.2217/fon-2017-0268. Epub 2017 Nov 29.
459. Chen ZX, Zhuang X, Zhu XH, Hao YL, Gu GF, Cai MZ, Qin G. Comparative Effectiveness of Prophylactic Strategies for Perinatal Transmission of Hepatitis B Virus: A Network Meta-analysis of Randomized Controlled Trials Open Forum Infect Dis. 2017 Oct 17;4(4):ofx225. doi: 10.1093/ofid/ofx225. eCollection 2017 Fall.
460. Luan R, Mu Z, Yue F, He S. Efficacy and Tolerability of Different Interventions in Children and Adolescents with Attention Deficit Hyperactivity Disorder Front Psychiatry. 2017 Nov 13;8:229. doi: 10.3389/fpsyt.2017.00229. eCollection 2017.
461. Ma Y, Wu X, Yu J, Zhu J, Pen X, Meng X. Can polysaccharide K improve therapeutic efficacy and safety in gastrointestinal cancer? a systematic review and network meta-analysis Oncotarget. 2017 Jul 6;8(51):89108-89118. doi: 10.18632/oncotarget.19059. eCollection 2017 Oct 24.
462. Zeng W, Liu Z, Dai H, Yan M, Luo H, Ke M, Cai X. Anti-fibrotic, anti-VEGF or radiotherapy treatments as adjuvants for pterygium excision: a systematic review and network meta-analysis BMC Ophthalmol. 2017 Nov 25;17(1):211. doi: 10.1186/s12886-017-0601-5.
463. Zhao S, Gao F, Zhang Y, Zhang Z, Zhang L. Bevacizumab in combination with different platinum-based doublets in the first-line treatment for advanced nonsquamous non-small-cell lung cancer: A network meta-analysis Int J Cancer. 2018 Apr 15;142(8):1676-1688. doi: 10.1002/ijc.31175. Epub 2017 Dec 4.
464. Sridharan K, Sivaramakrishnan G. Pharmacological interventions for preventing acute mountain sickness: a network meta-analysis and trial sequential analysis of randomized clinical trials Ann Med. 2018 Mar;50(2):147-155. doi: 10.1080/07853890.2017.1407034. Epub 2017 Nov 23.
465. Zhang Y, Liu Y, Su Y, You Y, Ma Y, Yang G, Song Y, Liu X, Wang M, Zhang L, Kou C. The metabolic side effects of 12 antipsychotic drugs used for the treatment of schizophrenia on glucose: a network meta-analysis BMC Psychiatry. 2017 Nov 21;17(1):373. doi: 10.1186/s12888-017-1539-0.
466. Shi L, Zhang X, Li J, Bai X. Favorable Effects of Endoscopic Ligation Combined with Drugs on Rebleeding and Mortality in Cirrhotic Patients: A Network Meta-Analysis Dig Dis. 2018;36(2):136-149. doi: 10.1159/000484082. Epub 2017 Nov 21.
467. Lopez-Olivo MA, Kakpovbia-Eshareturi V, des Bordes JK, Barbo A, Christensen R, Suarez-Almazor ME. Treating Early Undifferentiated Arthritis: A Systematic Review and Meta-Analysis of Direct and Indirect Trial Evidence Arthritis Care Res (Hoboken). 2018 Sep;70(9):1355-1365. doi: 10.1002/acr.23474. Epub 2018 Jul 5.
468. Li F, Zhang SH, Pang LM. Meta-analysis of efficacy and adverse events of erlotinib-based targeted therapies for advanced/metastatic non-small cell lung cancer Oncotarget. 2017 Jul 31;8(49):86816-86827. doi: 10.18632/oncotarget.19735. eCollection 2017 Oct 17.
469. Chou CY, Chang YT, Yang JL, Wang JY, Lee TE, Wang RY, Hung CC. Effect of Long-term Incretin-Based Therapies on Ischemic Heart Diseases in Patients with Type 2 Diabetes Mellitus: A Network Meta-analysis Sci Rep. 2017 Nov 17;7(1):15795. doi: 10.1038/s41598-017-16101-1.
470. Siddiqui MK, Khurana IS, Budhia S, Hettle R, Harty G, Wong SL. Systematic literature review and network meta-analysis of cladribine tablets versus alternative disease-modifying treatments for relapsing-remitting multiple sclerosis Curr Med Res Opin. 2018 Aug;34(8):1361-1371. doi: 10.1080/03007995.2017.1407303. Epub 2017 Nov 28.
471. Sridharan K, Sivaramakrishnan G. Growth factors for diabetic foot ulcers: mixed treatment comparison analysis of randomized clinical trials Br J Clin Pharmacol. 2018 Mar;84(3):434-444. doi: 10.1111/bcp.13470. Epub 2018 Jan 5.
472. Fonseca EC, Rocha EM, Arruda GV. Comparison among adjuvant treatments for primary pterygium: a network meta-analysis Br J Ophthalmol. 2018 Jun;102(6):748-756. doi: 10.1136/bjophthalmol-2017-310288. Epub 2017 Nov 16.
473. Reboussin DM, Allen NB, Griswold ME, Guallar E, Hong Y, Lackland DT, Miller EPR 3rd, Polonsky T, Thompson-Paul AM, Vupputuri S. Systematic Review for the 2017 ACC/AHA/AAPA/ABC/ACPM/AGS/APhA/ASH/ASPC/NMA/PCNA Guideline for the Prevention, Detection, Evaluation, and Management of High Blood Pressure in Adults: A Report of the American College of Cardiology/American Heart Association Task Force on Clinical Practice Guidelines J Am Coll Cardiol. 2018 May 15;71(19):2176-2198. doi: 10.1016/j.jacc.2017.11.004. Epub 2017 Nov 13.
474. Li F, Huang W, Zhang X. Efficacy and safety of different regimens for primary open-angle glaucoma or ocular hypertension: a systematic review and network meta-analysis Acta Ophthalmol. 2018 May;96(3):e277-e284. doi: 10.1111/aos.13568. Epub 2017 Nov 16.
475. Nazir J, Kelleher C, Aballéa S, Maman K, Hakimi Z, Mankowski C, Odeyemi I. Comparative efficacy and tolerability of solifenacin 5 mg/day versus other oral antimuscarinic agents in overactive bladder: A systematic literature review and network meta-analysis Neurourol Urodyn. 2018 Mar;37(3):986-996. doi: 10.1002/nau.23413. Epub 2017 Nov 15.
476. Tian Q, Du P, Li S, Bai Z, Yang Y, Zeng J. Effect of antitumor treatments on triple-negative breast cancer patients: A PRISMA-compliant network meta-analysis of randomized controlled trials Medicine (Baltimore). 2017 Nov;96(45):e8389. doi: 10.1097/MD.0000000000008389.
477. Guest JF, Esteban J, Manganelli AG, Novelli A, Rizzardini G, Serra M. Comparative efficacy and safety of antibiotics used to treat acute bacterial skin and skin structure infections: Results of a network meta-analysis PLoS One. 2017 Nov 14;12(11):e0187792. doi: 10.1371/journal.pone.0187792. eCollection 2017.
478. Jimenez D, Martin-Saborido C, Muriel A, Zamora J, Morillo R, Barrios D, Klok FA, Huisman MV, Tapson V, Yusen RD. Efficacy and safety outcomes of recanalisation procedures in patients with acute symptomatic pulmonary embolism: systematic review and network meta-analysis Thorax. 2018 May;73(5):464-471. doi: 10.1136/thoraxjnl-2017-210040. Epub 2017 Nov 13.
479. Lin H, Wang M, Yu Y, Qin Z, Zhong X, Ma J, Zhao F, Zhang X. Efficacy and tolerability of pharmacological interventions for pulmonary arterial hypertension: A network meta-analysis Pulm Pharmacol Ther. 2018 Jun;50:1-10. doi: 10.1016/j.pupt.2017.11.002. Epub 2017 Nov 8.
480. Click Z, Varughese C, Geswein L. Comparison of antipseudomonal beta-lactams for febrile neutropenia empiric therapy Clin Microbiol Infect. 2018 Mar;24(3):324. doi: 10.1016/j.cmi.2017.10.027. Epub 2017 Nov 9.
481. Nakashoji A, Hayashida T, Yokoe T, Maeda H, Toyota T, Kikuchi M, Watanuki R, Nagayama A, Seki T, Takahashi M, Abe T, Kitagawa Y. The updated network meta-analysis of neoadjuvant therapy for HER2-positive breast cancer Cancer Treat Rev. 2018 Jan;62:9-17. doi: 10.1016/j.ctrv.2017.10.009. Epub 2017 Oct 31.
482. Xu JB, Jiang B, Chen Y, Qi FZ, Zhang JH, Yuan H. Optimal adjuvant chemotherapy for resected pancreatic adenocarcinoma: a systematic review and network meta-analysis Oncotarget. 2017 Jul 7;8(46):81419-81429. doi: 10.18632/oncotarget.19082. eCollection 2017 Oct 6.
483. Kim HS, Jeong CW, Kwak C, Kim HH, Ku JH. Adjuvant chemotherapy for muscle-invasive bladder cancer: a systematic review and network meta-analysis of randomized clinical trials Oncotarget. 2017 Sep 18;8(46):81204-81214. doi: 10.18632/oncotarget.20979. eCollection 2017 Oct 6.
484. López-López JA, Humphriss RL, Beswick AD, Thom HHZ, Hunt LP, Burston A, Fawsitt CG, Hollingworth W, Higgins JPT, Welton NJ, Blom AW, Marques EMR. Choice of implant combinations in total hip replacement: systematic review and network meta-analysis BMJ. 2017 Nov 2;359:j4651. doi: 10.1136/bmj.j4651.
485. Iketani R, Kawasaki Y, Yamada H. Comparative Utility of Atypical Antipsychotics for the Treatment of Psychosis in Parkinson's Disease: A Systematic Review and Bayesian Network Meta-analysis Biol Pharm Bull. 2017;40(11):1976-1982. doi: 10.1248/bpb.b17-00602.
486. Yang TX, Liao BH, Chen YT, Li H, He Q, Liu QY, Wang KJ. A network meta-analysis on the beneficial effect of medical expulsive therapy after extracorporeal shock wave lithotripsy Sci Rep. 2017 Oct 31;7(1):14429. doi: 10.1038/s41598-017-14862-3.
487. Jiang X, Shen W, Xu X, Shen X, Li Y, He Q. Immunosuppressive therapy for steroid-resistant nephrotic syndrome: a Bayesian network meta-analysis of randomized controlled studies Clin Exp Nephrol. 2018 Jun;22(3):562-569. doi: 10.1007/s10157-017-1484-8. Epub 2017 Oct 27.
488. Gajulapalli RD, Dias S, Pattanshetty DJ, Athappan G. Optimal duration of dual antiplatelet therapy after drug eluting stent implantation: a network meta-analysis Anatol J Cardiol. 2017 Oct;18(4):251-260. doi: 10.14744/AnatolJCardiol.2017.7672.
489. He HL, Yao WX. A network meta-analysis of short-term efficacy of different single-drug targeted therapies in the treatment of renal cell carcinoma Biosci Rep. 2017 Dec 7;37(6):BSR20170827. doi: 10.1042/BSR20170827. Print 2017 Dec 22.
490. Keshavarz K, Lotfi F, Sanati E, Salesi M, Hashemi-Meshkini A, Jafari M, Mojahedian MM, Najafi B, Nikfar S. Linagliptin versus sitagliptin in patients with type 2 diabetes mellitus: a network meta-analysis of randomized clinical trials Daru. 2017 Oct 25;25(1):23. doi: 10.1186/s40199-017-0189-6.
491. Li S, Yang H, Guo P, Ao X, Wan J, Li Q, Tan L. Efficacy and safety of immunosuppressive medications for steroid-resistant nephrotic syndrome in children: a systematic review and network meta-analysis Oncotarget. 2017 Aug 21;8(42):73050-73062. doi: 10.18632/oncotarget.20377. eCollection 2017 Sep 22.
492. Wang SL, Dong WB, Dong XL, Zhu WM, Wang FF, Han F, Yan X. Comparison of twelve single-drug regimens for the treatment of type 2 diabetes mellitus Oncotarget. 2017 Aug 16;8(42):72700-72713. doi: 10.18632/oncotarget.20282. eCollection 2017 Sep 22.
493. Li Z, Li Y, Liu Y, Xu W, Wang Q. Comparative risk of new-onset diabetes mellitus for antihypertensive drugs: A network meta-analysis J Clin Hypertens (Greenwich). 2017 Dec;19(12):1348-1356. doi: 10.1111/jch.13108. Epub 2017 Oct 25.
494. Guo X, Tan Z. Effectiveness of interventions for pain relief in hysterosalpingographyAnetwork meta-analysis and systematic review Pak J Med Sci. 2017 Jul-Aug;33(4):1029-1035. doi: 10.12669/pjms.334.13065.
495. Zhang J, Chen J, Yin Y, Zhang L, Zhang H. Therapeutic effects of different drugs on obstructive sleep apnea/hypopnea syndrome in children World J Pediatr. 2017 Dec;13(6):537-543. doi: 10.1007/s12519-017-0062-1. Epub 2017 Oct 20.
496. Bonovas S, Nikolopoulos GK, Lytras T, Fiorino G, Peyrin-Biroulet L, Danese S. Comparative safety of systemic and low-bioavailability steroids in inflammatory bowel disease: Systematic review and network meta-analysis Br J Clin Pharmacol. 2018 Feb;84(2):239-251. doi: 10.1111/bcp.13456. Epub 2017 Dec 1.
497. Cheng J, Cai M, Shuai X, Gao J, Wang G, Tao K. Comparative efficacy and tolerability of antiemetic prophylaxis for adult highly emetogenic chemotherapy: A network meta-analysis of 143 randomized controlled trials Int J Cancer. 2018 Mar 1;142(5):1067-1076. doi: 10.1002/ijc.31125. Epub 2017 Oct 31.
498. Baune BT, Brignone M, Larsen KG. A Network Meta-Analysis Comparing Effects of Various Antidepressant Classes on the Digit Symbol Substitution Test (DSST) as a Measure of Cognitive Dysfunction in Patients with Major Depressive Disorder Int J Neuropsychopharmacol. 2018 Feb 1;21(2):97-107. doi: 10.1093/ijnp/pyx070.
499. Al Yami MS, Silva MA, Donovan JL, Kanaan AO. Venous thromboembolism prophylaxis in medically ill patients: a mixed treatment comparison meta-analysis J Thromb Thrombolysis. 2018 Jan;45(1):36-47. doi: 10.1007/s11239-017-1562-5.
500. McLeod SL, Brignardello-Petersen R, Worster A, You J, Iansavichene A, Guyatt G, Cheskes S. Comparative effectiveness of antiarrhythmics for out-of-hospital cardiac arrest: A systematic review and network meta-analysis Resuscitation. 2017 Dec;121:90-97. doi: 10.1016/j.resuscitation.2017.10.012. Epub 2017 Oct 14.
501. Horita N, Nagashima A, Nakashima K, Shibata Y, Ito K, Goto A, Yamanaka T, Kaneko T. The best platinum regimens for chemo-naive incurable non-small cell lung cancer: network meta-analysis Sci Rep. 2017 Oct 13;7(1):13185. doi: 10.1038/s41598-017-13724-2.
502. Zhang BF, Wang PF, Huang H, Cong YX, Wang H, Zhuang Y. Interventions for treating displaced intracapsular femoral neck fractures in the elderly: a Bayesian network meta-analysis of randomized controlled trials Sci Rep. 2017 Oct 12;7(1):13103. doi: 10.1038/s41598-017-13377-1.
503. D'Ascenzo F, Iannaccone M, Saint-Hilary G, Bertaina M, Schulz-Schüpke S, Wahn Lee C, Chieffo A, Helft G, Gili S, Barbero U, Biondi Zoccai G, Moretti C, Ugo F, D'Amico M, Garbo R, Stone G, Rettegno S, Omedè P, Conrotto F, Templin C, Colombo A, Park SJ, Kastrati A, Hildick-Smith D, Gasparini M, Gaita F. Impact of design of coronary stents and length of dual antiplatelet therapies on ischaemic and bleeding events: a network meta-analysis of 64 randomized controlled trials and 102 735 patients Eur Heart J. 2017 Nov 7;38(42):3160-3172. doi: 10.1093/eurheartj/ehx437.
504. Wang Y, Xie J, Xing Y, Chen L, Li Y, Meng T, Dong W, Wang X, Dong Y. Choosing Optimal Antifungal Agents To Prevent Fungal Infections in Nonneutropenic Critically Ill Patients: Trial Sequential Analysis, Network Meta-analysis, and Pharmacoeconomic Analysis Antimicrob Agents Chemother. 2017 Nov 22;61(12):e00620-17. doi: 10.1128/AAC.00620-17. Print 2017 Dec.
505. Gao JH, Chu XC, Wang LL, Ning B, Zhao CX. Effects of different anticoagulant drugs on the prevention of complications in patients after arthroplasty: A network meta-analysis Medicine (Baltimore). 2017 Oct;96(40):e8059. doi: 10.1097/MD.0000000000008059.
506. Lee SWH, Chan CKY, Chua SS, Chaiyakunapruk N. Comparative effectiveness of telemedicine strategies on type 2 diabetes management: A systematic review and network meta-analysis Sci Rep. 2017 Oct 4;7(1):12680. doi: 10.1038/s41598-017-12987-z.
507. Zhang D, Zheng J, Ni M, Wu J, Wang K, Duan X, Zhang X, Zhang B. Comparative efficacy and safety of Chinese herbal injections combined with the FOLFOX regimen for treating gastric cancer in China: a network meta-analysis Oncotarget. 2017 Aug 18;8(40):68873-68889. doi: 10.18632/oncotarget.20320. eCollection 2017 Sep 15.
508. Zhou M, Yu P, Hernick Davin DB, Li Y, Wang Y, Fu L, Zhang J. Is FOLFOXIRI alone or combined with targeted therapy administered as first-line treatment a reasonable choice for most patients with mCRC? Systematic review and network meta-analysis Oncotarget. 2017 May 9;8(37):62339-62348. doi: 10.18632/oncotarget.17725. eCollection 2017 Sep 22.
509. Corbett M, Chehadah F, Biswas M, Moe-Byrne T, Palmer S, Soares M, Walton M, Harden M, Ho P, Woolacott N, Bojke L. Certolizumab pegol and secukinumab for treating active psoriatic arthritis following inadequate response to disease-modifying antirheumatic drugs: a systematic review and economic evaluation Health Technol Assess. 2017 Oct;21(56):1-326. doi: 10.3310/hta21560.
510. Toth PP, Worthy G, Gandra SR, Sattar N, Bray S, Cheng LI, Bridges I, Worth GM, Dent R, Forbes CA, Deshpande S, Ross J, Kleijnen J, Stroes ESG. Systematic Review and Network Meta-Analysis on the Efficacy of Evolocumab and Other Therapies for the Management of Lipid Levels in Hyperlipidemia J Am Heart Assoc. 2017 Oct 2;6(10):e005367. doi: 10.1161/JAHA.116.005367.
511. Arai Y, Jo T, Matsui H, Kondo T, Takaori-Kondo A. Comparison of up-front treatments for newly diagnosed immune thrombocytopenia -a systematic review and network meta-analysis Haematologica. 2018 Jan;103(1):163-171. doi: 10.3324/haematol.2017.174615. Epub 2017 Sep 29.
512. Doosti-Irani A, Mansournia MA, Rahimi-Foroushani A, Haddad P, Holakouie-Naieni K. Complications of stent placement in patients with esophageal cancer: A systematic review and network meta-analysis PLoS One. 2017 Oct 2;12(10):e0184784. doi: 10.1371/journal.pone.0184784. eCollection 2017.
513. Bae SC, Lee YH. Comparative efficacy and tolerability of monotherapy with leflunomide or tacrolimus for the treatment of rheumatoid arthritis: a Bayesian network meta-analysis of randomized controlled trials Clin Rheumatol. 2018 Feb;37(2):323-330. doi: 10.1007/s10067-017-3857-5. Epub 2017 Oct 1.
514. Wang C, Song DJ, Xu ZL, Xie SP, Hu JH. A network meta-analysis of the short-term efficacy of five chemotherapy regimens based on cisplatin and fluorouracil for esophagogastric junctional adenocarcinoma Exp Mol Med. 2017 Sep 29;49(9):e383. doi: 10.1038/emm.2017.170.
515. Putananon C, Tuchinda H, Arirachakaran A, Wongsak S, Narinsorasak T, Kongtharvonskul J. Comparison of direct anterior, lateral, posterior and posterior-2 approaches in total hip arthroplasty: network meta-analysis Eur J Orthop Surg Traumatol. 2018 Feb;28(2):255-267. doi: 10.1007/s00590-017-2046-1. Epub 2017 Sep 27.
516. Li MJ, Li Q, Sun M, Liu LQ. Comparative effectiveness and acceptability of the FDA-licensed proton pump inhibitors for erosive esophagitis: A PRISMA-compliant network meta-analysis Medicine (Baltimore). 2017 Sep;96(39):e8120. doi: 10.1097/MD.0000000000008120.
517. Moser W, Schindler C, Keiser J. Efficacy of recommended drugs against soil transmitted helminths: systematic review and network meta-analysis BMJ. 2017 Sep 25;358:j4307. doi: 10.1136/bmj.j4307.
518. Shah R, Askari R, Haji SA, Rashid A. Mortality and operator experience with vascular access for percutaneous coronary intervention in patients with acute coronary syndromes: A pairwise and network meta-analysis of randomized controlled trials Int J Cardiol. 2017 Dec 1;248:114-119. doi: 10.1016/j.ijcard.2017.05.026.
519. Pang B, Zhao LH, Li XL, Song J, Li QW, Liao X, Feng S, Zhao XY, Zheng YJ, Gou XW, Ni Q, Tong XL. Different intervention strategies for preventing type 2 diabetes mellitus in China: A systematic review and network meta-analysis of randomized controlled trials Diabetes Obes Metab. 2018 Mar;20(3):718-722. doi: 10.1111/dom.13121. Epub 2017 Nov 8.
520. Huang R, Zhao PF, Xu JH, Liu DD, Luo FD, Dai YH. Effects of placebo-controlled insulin-sensitizing drugs on hormonal parameters in polycystic ovary syndrome patients: A network meta-analysis J Cell Biochem. 2018 Mar;119(3):2501-2511. doi: 10.1002/jcb.26410. Epub 2017 Oct 30.
521. Palpacuer C, Duprez R, Huneau A, Locher C, Boussageon R, Laviolle B, Naudet F. Pharmacologically controlled drinking in the treatment of alcohol dependence or alcohol use disorders: a systematic review with direct and network meta-analyses on nalmefene, naltrexone, acamprosate, baclofen and topiramate Addiction. 2018 Feb;113(2):220-237. doi: 10.1111/add.13974. Epub 2017 Sep 20.
522. Hu Q, Zhang F, Teng W, Hao F, Zhang J, Yin M, Wang N. Efficacy and safety of antiepileptic drugs for refractory partial-onset epilepsy: a network meta-analysis J Neurol. 2018 Jan;265(1):1-11. doi: 10.1007/s00415-017-8621-x. Epub 2017 Sep 22.
523. Yang L, Guo G, Sun L, Li C, Zhang H. Efficacy and safety of traditional chemotherapies for patients with ovarian neoplasm: a network meta-analysis Oncotarget. 2017 Mar 30;8(35):59867-59877. doi: 10.18632/oncotarget.16729. eCollection 2017 Aug 29.
524. Wu J, Chen WK, Zhang W, Zhang JS, Liu JH, Jiang YM, Fang KW. Network meta-analysis of the efficacy and adverse effects of several treatments for advanced/metastatic prostate cancer Oncotarget. 2017 Aug 2;8(35):59709-59719. doi: 10.18632/oncotarget.19810. eCollection 2017 Aug 29.
525. Kang M, Jeong CW, Kwak C, Ku JH, Kim HH. Comparing the clinical efficacy of abiraterone acetate, enzalutamide, and orteronel in patients with metastatic castration-resistant prostate cancer by performing a network meta-analysis of eight randomized controlled trials Oncotarget. 2017 May 10;8(35):59690-59697. doi: 10.18632/oncotarget.17741. eCollection 2017 Aug 29.
526. Feng F, Xu Q, Yan F, Xie Y, Deng Z, Hu C, Zhu X, Cai L. Comparison of 7 Surgical Interventions for Lumbar Disc Herniation: A Network Meta-analysis Pain Physician. 2017 Sep;20(6):E863-E871.
527. Li X, Wang R, Xing X, Shi X, Tian J, Zhang J, Ge L, Zhang J, Li L, Yang K. Acupuncture for Myofascial Pain Syndrome: A Network Meta-Analysis of 33 Randomized Controlled Trials Pain Physician. 2017 Sep;20(6):E883-E902.
528. Katsanos K, Kitrou P, Spiliopoulos S, Maroulis I, Petsas T, Karnabatidis D. Comparative effectiveness of different transarterial embolization therapies alone or in combination with local ablative or adjuvant systemic treatments for unresectable hepatocellular carcinoma: A network meta-analysis of randomized controlled trials PLoS One. 2017 Sep 21;12(9):e0184597. doi: 10.1371/journal.pone.0184597. eCollection 2017.
529. Liang HL, Ma SJ, Xiao YN, Tan HZ. Comparative efficacy and safety of oral antidiabetic drugs and insulin in treating gestational diabetes mellitus: An updated PRISMA-compliant network meta-analysis Medicine (Baltimore). 2017 Sep;96(38):e7939. doi: 10.1097/MD.0000000000007939.
530. Zhang SH, Liu GF, Li XF, Liu L, Yu SN. Efficacy of different chemotherapy regimens in treatment of advanced or metastatic pancreatic cancer: A network meta-analysis J Cell Physiol. 2018 Apr;233(4):3352-3374. doi: 10.1002/jcp.26183. Epub 2017 Nov 1.
531. Sridharan K, Sivaramakrishnan G. Vasoactive Agents for Hepatorenal Syndrome: A Mixed Treatment Comparison Network Meta-Analysis and Trial Sequential Analysis of Randomized Clinical Trials J Gen Intern Med. 2018 Jan;33(1):97-102. doi: 10.1007/s11606-017-4178-8. Epub 2017 Sep 18.
532. Poon Y, Pechlivanoglou P, Alibhai SMH, Naimark D, Hoch JS, Papadimitropoulos E, Hogan ME, Krahn M. Systematic review and network meta-analysis on the relative efficacy of osteoporotic medications: men with prostate cancer on continuous androgen-deprivation therapy to reduce risk of fragility fractures BJU Int. 2018 Jan;121(1):17-28. doi: 10.1111/bju.14015. Epub 2017 Oct 15.
533. Zhong P, Wu D, Ye X, Wu Y, Li T, Tong S, Liu X. Secondary prevention of major cerebrovascular events with seven different statins: a multi-treatment meta-analysis Drug Des Devel Ther. 2017 Aug 30;11:2517-2526. doi: 10.2147/DDDT.S135785. eCollection 2017.
534. Sridharan K, Sivaramakrishnan G. Drugs for Treating Opioid-Induced Constipation: A Mixed Treatment Comparison Network Meta-analysis of Randomized Controlled Clinical Trials J Pain Symptom Manage. 2018 Feb;55(2):468-479.e1. doi: 10.1016/j.jpainsymman.2017.08.022. Epub 2017 Sep 15.
535. Wu MY, Chen TT, Chen YC, Tarng DC, Wu YC, Lin HH, Tu YK. Effects and safety of oral tolvaptan in patients with congestive heart failure: A systematic review and network meta-analysis PLoS One. 2017 Sep 12;12(9):e0184380. doi: 10.1371/journal.pone.0184380. eCollection 2017.
536. Ren S, Wang Y, Xian L, Toyama T, Jardine M, Li G, Perkovic V, Hong D. Comparative effectiveness and tolerance of immunosuppressive treatments for idiopathic membranous nephropathy: A network meta-analysis PLoS One. 2017 Sep 12;12(9):e0184398. doi: 10.1371/journal.pone.0184398. eCollection 2017.
537. Yan Y, Xu D. The Effect of Adjuvant Treatment to Prevent and Treat Intrauterine Adhesions: A Network Meta-Analysis of Randomized Controlled Trials J Minim Invasive Gynecol. 2018 May-Jun;25(4):589-599. doi: 10.1016/j.jmig.2017.09.006. Epub 2017 Sep 8.
538. Iftikhar IH, Alghothani L, Trotti LM. Gabapentin enacarbil, pregabalin and rotigotine are equally effective in restless legs syndrome: a comparative meta-analysis Eur J Neurol. 2017 Dec;24(12):1446-1456. doi: 10.1111/ene.13449. Epub 2017 Oct 5.
539. Agarwal R, Bartsch SM, Kelly BJ, Prewitt M, Liu Y, Chen Y, Umscheid CA. Newer glycopeptide antibiotics for treatment of complicated skin and soft tissue infections: systematic review, network meta-analysis and cost analysis Clin Microbiol Infect. 2018 Apr;24(4):361-368. doi: 10.1016/j.cmi.2017.08.028. Epub 2017 Sep 4.
540. Li BD, Bi ZY, Liu JF, Si WJ, Shi QQ, Xue LP, Bai J. Adverse effects produced by different drugs used in the treatment of Parkinson's disease: A mixed treatment comparison CNS Neurosci Ther. 2017 Oct;23(10):827-842. doi: 10.1111/cns.12727. Epub 2017 Sep 4.
541. Antoniou SA, Mavridis D, Hajibandeh S, Hajibandeh S, Antoniou GA, Gorter R, Tenhagen M, Koutras C, Pointner R, Chalkiadakis GE, Granderath FA, Fragiadakis GF, Philalithis AE, Bonjer HJ. Optimal stump management in laparoscopic appendectomy: A network meta-analysis by the Minimally Invasive Surgery Synthesis of Interventions and Outcomes Network Surgery. 2017 Nov;162(5):994-1005. doi: 10.1016/j.surg.2017.07.013. Epub 2017 Aug 30.
542. Taipale K, Winfree KB, Boye M, Basson M, Sleilaty G, Eaton J, Evans R, Chouaid C. A cost-effectiveness analysis of first-line induction and maintenance treatment sequences in patients with advanced nonsquamous non-small-cell lung cancer in France Clinicoecon Outcomes Res. 2017 Aug 16;9:505-518. doi: 10.2147/CEOR.S128371. eCollection 2017.
543. Zhu LN, Chen D, Xu D, Tan G, Wang HJ, Liu L. Newer antiepileptic drugs compared to levetiracetam as adjunctive treatments for uncontrolled focal epilepsy: An indirect comparison Seizure. 2017 Oct;51:121-132. doi: 10.1016/j.seizure.2017.07.017. Epub 2017 Aug 16.
544. Guyot P, Pandhi S, Nixon RM, Iqbal A, Chaves RL, Andrew Moore R. Efficacy and safety of diclofenac in osteoarthritis: Results of a network meta-analysis of unpublished legacy studies Scand J Pain. 2017 Jul;16:74-88. doi: 10.1016/j.sjpain.2017.03.006. Epub 2017 Apr 22.
545. Wang J, Xu H, Liu P, Li M. Network meta-analysis of success rate and safety in antibiotic treatments of bronchitis Int J Chron Obstruct Pulmon Dis. 2017 Aug 9;12:2391-2405. doi: 10.2147/COPD.S139521. eCollection 2017.
546. Jinatongthai P, Kongwatcharapong J, Foo CY, Phrommintikul A, Nathisuwan S, Thakkinstian A, Reid CM, Chaiyakunapruk N. Comparative efficacy and safety of reperfusion therapy with fibrinolytic agents in patients with ST-segment elevation myocardial infarction: a systematic review and network meta-analysis Lancet. 2017 Aug 19;390(10096):747-759. doi: 10.1016/S0140-6736(17)31441-1.
547. Pike E, Hamidi V, Saeterdal I, Odgaard-Jensen J, Klemp M. Multiple treatment comparison of seven new drugs for patients with advanced malignant melanoma: a systematic review and health economic decision model in a Norwegian setting BMJ Open. 2017 Aug 21;7(8):e014880. doi: 10.1136/bmjopen-2016-014880.
548. Wu S, Chai S, Yang J, Cai T, Xu Y, Yang Z, Zhang Y, Ji L, Sun F, Zhan S. Gastrointestinal Adverse Events of Dipeptidyl Peptidase 4 Inhibitors in Type 2 Diabetes: A Systematic Review and Network Meta-analysis Clin Ther. 2017 Sep;39(9):1780-1789.e33. doi: 10.1016/j.clinthera.2017.07.036. Epub 2017 Aug 18.
549. Chen X, Huang T, Cao X, Xu G. Comparative Efficacy of Drugs for Preventing Acute Kidney Injury after Cardiac Surgery: A Network Meta-Analysis Am J Cardiovasc Drugs. 2018 Feb;18(1):49-58. doi: 10.1007/s40256-017-0245-0.
550. Natto ZS, Yaghmoor W, Bannuru RR, Nevins M. Identification and Efficacy Ranking of Allograft and Xenograft for Extraction and Ridge Preservation Procedures Int J Periodontics Restorative Dent. 2017 Sep/Oct;37(5):e253-e260. doi: 10.11607/prd.3323.
551. Zhang J, Huang Y, Wang C, He Y, Zheng S, Wu K. Efficacy and safety of endocrine monotherapy as first-line treatment for hormone-sensitive advanced breast cancer: A network meta-analysis Medicine (Baltimore). 2017 Aug;96(33):e7846. doi: 10.1097/MD.0000000000007846.
552. Tonin FS, Steimbach LM, Borba HH, Sanches AC, Wiens A, Pontarolo R, Fernandez-Llimos F. Efficacy and safety of amphotericin B formulations: a network meta-analysis and a multicriteria decision analysis J Pharm Pharmacol. 2017 Dec;69(12):1672-1683. doi: 10.1111/jphp.12802. Epub 2017 Aug 17.
553. Jung YS, Park CH, Nam E, Eun CS, Park DI, Han DS. Comparative efficacy of cold polypectomy techniques for diminutive colorectal polyps: a systematic review and network meta-analysis Surg Endosc. 2018 Mar;32(3):1149-1159. doi: 10.1007/s00464-017-5786-4. Epub 2017 Aug 15.
554. Becker-Schiebe M, Christiansen H. [The best multimodal treatment for locally advanced nasopharyngeal carcinoma : An update of the MAC-NPC data] Strahlenther Onkol. 2017 Oct;193(10):856-858. doi: 10.1007/s00066-017-1189-7.
555. Tsoi B, Akioyamen LE, Bonner A, Frankfurter C, Levine M, Pullenayegum E, Goeree R, O'Reilly D. Comparative Efficacy of Angiotensin II Antagonists in Essential Hypertension: Systematic Review and Network Meta-Analysis of Randomised Controlled Trials Heart Lung Circ. 2018 Jun;27(6):666-682. doi: 10.1016/j.hlc.2017.06.721. Epub 2017 Jul 14.
556. Wang Z, Zheng J, Zhao Y, Xiang Y, Chen X, Jin Y. Effectiveness and Tolerability of Anticoagulants for Thromboprophylaxis after Major Joint Surgery: a Network Meta-Analysis Cell Physiol Biochem. 2017;42(5):1999-2020. doi: 10.1159/000479840. Epub 2017 Aug 9.
557. Tomizawa Y, Melek J, Komaki Y, Kavitt RT, Sakuraba A. Efficacy of Pharmacologic Therapy for Eosinophilic Esophagitis: A Systematic Review and Network Meta-Analysis J Clin Gastroenterol. 2018 Aug;52(7):596-606. doi: 10.1097/MCG.0000000000000878.
558. Hur M, Park SK, Koo CH, Jung ED, Kang P, Kim WH, Kim JT, Jung CW, Bahk JH. Comparative efficacy and safety of anticoagulants for prevention of venous thromboembolism after hip and knee arthroplasty Acta Orthop. 2017 Dec;88(6):634-641. doi: 10.1080/17453674.2017.1361131. Epub 2017 Aug 8.
559. Yang X, Bai Q, Li Y, Liu H, Guo H, Zhang X. Comparative Efficacy and Safety of Phosphate Binders in Hyperphosphatemia Patients With Chronic Kidney Disease JPEN J Parenter Enteral Nutr. 2018 May;42(4):766-777. doi: 10.1177/0148607117715440. Epub 2017 Dec 19.
560. Sun Y, Liang Y, Jiao Y, Lin J, Qu H, Xu J, Zhao C. Comparative efficacy and acceptability of antidepressant treatment in poststroke depression: a multiple-treatments meta-analysis BMJ Open. 2017 Aug 3;7(8):e016499. doi: 10.1136/bmjopen-2017-016499.
561. Whegang Youdom S, Tahar R, Basco LK. Comparison of anti-malarial drugs efficacy in the treatment of uncomplicated malaria in African children and adults using network meta-analysis Malar J. 2017 Aug 3;16(1):311. doi: 10.1186/s12936-017-1963-0.
562. Wang J, Luo L, Wang D, Guo B, Li J, Yang Z, Tang D. Combination adjuvant chemotherapy with targeted drugs for treatment of colorectal cancer: A network meta-analysis J Cell Biochem. 2018 Feb;119(2):1521-1537. doi: 10.1002/jcb.26312. Epub 2017 Nov 24.
563. Ha V, Bonner AJ, Jadoo JK, Beyene J, Anand SS, de Souza RJ. The effects of various diets on glycemic outcomes during pregnancy: A systematic review and network meta-analysis PLoS One. 2017 Aug 3;12(8):e0182095. doi: 10.1371/journal.pone.0182095. eCollection 2017.
564. Wang G, Sui L, Gai P, Li G, Qi X, Jiang X. The efficacy and safety of vertebral fracture prevention therapies in post-menopausal osteoporosis treatment: Which therapies work best? a network meta-analysis Bone Joint Res. 2017 Jul;6(7):452-463. doi: 10.1302/2046-3758.67.BJR-2016-0292.R1.
565. Zenner D, Beer N, Harris RJ, Lipman MC, Stagg HR, van der Werf MJ. Treatment of Latent Tuberculosis Infection: An Updated Network Meta-analysis Ann Intern Med. 2017 Aug 15;167(4):248-255. doi: 10.7326/M17-0609. Epub 2017 Aug 1.
566. Zhang Y, Sun N, Jiang X, Xi Y. Comparative efficacy of β-blockers on mortality and cardiovascular outcomes in patients with hypertension: a systematic review and network meta-analysis J Am Soc Hypertens. 2017 Jul;11(7):394-401. doi: 10.1016/j.jash.2017.05.001. Epub 2017 May 18.
567. Al Sawah S, Foster SA, Burge R, Amato D, Schacht A, Zhu B, Hartz S, Leonardi C. Cost per additional responder for ixekizumab and other FDA-approved biologics in moderate-to-severe plaque psoriasis J Med Econ. 2017 Dec;20(12):1224-1230. doi: 10.1080/13696998.2017.1362413. Epub 2017 Aug 22.
568. Yu Z, Guo X, Jiang Y, Teng L, Luo J, Wang P, Liang Y, Zhang H. Adjuvant endocrine monotherapy for postmenopausal early breast cancer patients with hormone-receptor positive: a systemic review and network meta-analysis Breast Cancer. 2018 Jan;25(1):8-16. doi: 10.1007/s12282-017-0794-8. Epub 2017 Jul 28.
569. Cazzola M, Rogliani P, Calzetta L, Hanania NA, Matera MG. Impact of Mucolytic Agents on COPD Exacerbations: A Pair-wise and Network Meta-analysis COPD. 2017 Oct;14(5):552-563. doi: 10.1080/15412555.2017.1347918. Epub 2017 Jul 28.
570. Wilson FR, Varu A, Mitra D, Cameron C, Iyer S. Systematic review and network meta-analysis comparing palbociclib with chemotherapy agents for the treatment of postmenopausal women with HR-positive and HER2-negative advanced/metastatic breast cancer Breast Cancer Res Treat. 2017 Nov;166(1):167-177. doi: 10.1007/s10549-017-4404-4. Epub 2017 Jul 27.
571. Guo JR, Jin XJ, Shen HC, Wang H, Zhou X, Liu XQ, Zhu NN. A Comparison of the Efficacy and Tolerability of the Treatments for Sciatica: A Network Meta-Analysis Ann Pharmacother. 2017 Dec;51(12):1041-1052. doi: 10.1177/1060028017722008. Epub 2017 Jul 26.
572. Mihara T, Asakura A, Owada G, Yokoi A, Ka K, Goto T. A network meta-analysis of the clinical properties of various types of supraglottic airway device in children Anaesthesia. 2017 Oct;72(10):1251-1264. doi: 10.1111/anae.13970. Epub 2017 Jul 24.
573. Zhu Y, Krause M, Huhn M, Rothe P, Schneider-Thoma J, Chaimani A, Li C, Davis JM, Leucht S. Antipsychotic drugs for the acute treatment of patients with a first episode of schizophrenia: a systematic review with pairwise and network meta-analyses Lancet Psychiatry. 2017 Sep;4(9):694-705. doi: 10.1016/S2215-0366(17)30270-5. Epub 2017 Jul 20.
574. Tang H, Dai Q, Shi W, Zhai S, Song Y, Han J. SGLT2 inhibitors and risk of cancer in type 2 diabetes: a systematic review and meta-analysis of randomised controlled trials Diabetologia. 2017 Oct;60(10):1862-1872. doi: 10.1007/s00125-017-4370-8. Epub 2017 Jul 19.
575. Sridharan K, Sivaramakrishnan G. Interventions for Refractory Trigeminal Neuralgia: A Bayesian Mixed Treatment Comparison Network Meta-Analysis of Randomized Controlled Clinical Trials Clin Drug Investig. 2017 Sep;37(9):819-831. doi: 10.1007/s40261-017-0553-9.
576. Zheng JN, Han YJ, Zou TT, Zhou YJ, Sun DQ, Zhong JH, Braddock M, Zheng MH. Comparative efficacy of vasoconstrictor therapies for type 1 hepatorenal syndrome: a network meta-analysis Expert Rev Gastroenterol Hepatol. 2017 Nov;11(11):1009-1018. doi: 10.1080/17474124.2017.1356223. Epub 2017 Jul 27.
577. He YM, Yu C, Li WB, Li ZP, Xu N. Evaluation of short-term effectiveness of eight targeted agents combined with chemotherapy for treating esophageal-gastric junction adenocarcinoma: A network meta-analysis J Cell Biochem. 2018 Jan;119(1):1183-1192. doi: 10.1002/jcb.26288. Epub 2017 Oct 5.
578. Sukhato K, Lotrakul M, Dellow A, Ittasakul P, Thakkinstian A, Anothaisintawee T. Efficacy of home-based non-pharmacological interventions for treating depression: a systematic review and network meta-analysis of randomised controlled trials BMJ Open. 2017 Jul 12;7(7):e014499. doi: 10.1136/bmjopen-2016-014499.
579. Liu T, Li W, Zhou H, Wang Z. Verifying the Relative Efficacy between Continuous Positive Airway Pressure Therapy and Its Alternatives for Obstructive Sleep Apnea: A Network Meta-analysis Front Neurol. 2017 Jun 28;8:289. doi: 10.3389/fneur.2017.00289. eCollection 2017.
580. da Costa BR, Reichenbach S, Keller N, Nartey L, Wandel S, Jüni P, Trelle S. Effectiveness of non-steroidal anti-inflammatory drugs for the treatment of pain in knee and hip osteoarthritis: a network meta-analysis Lancet. 2017 Jul 8;390(10090):e21-e33. doi: 10.1016/S0140-6736(17)31744-0.
581. Jerzak KJ, Delos Santos K, Saluja R, Lien K, Lee J, Chan KKW. A network meta-analysis of the sequencing and types of systemic therapies with definitive radiotherapy in locally advanced squamous cell carcinoma of the head and neck (LASCCHN)☆ Oral Oncol. 2017 Aug;71:1-10. doi: 10.1016/j.oraloncology.2017.05.011. Epub 2017 Jun 3.
582. Huxley N, Crathorne L, Varley-Campbell J, Tikhonova I, Snowsill T, Briscoe S, Peters J, Bond M, Napier M, Hoyle M. The clinical effectiveness and cost-effectiveness of cetuximab (review of technology appraisal no. 176) and panitumumab (partial review of technology appraisal no. 240) for previously untreated metastatic colorectal cancer: a systematic review and economic evaluation Health Technol Assess. 2017 Jun;21(38):1-294. doi: 10.3310/hta21380.
583. Wang XF, Huang WF, Nie J, Zhou Y, Tan DW, Jiang JH. Toxicity of chemotherapy regimens in advanced and metastatic pancreatic cancer therapy: A network meta-analysis J Cell Biochem. 2018 Jul;119(7):5082-5103. doi: 10.1002/jcb.26266. Epub 2018 Apr 6.
584. Xu WW, Hu SJ, Wu T. Risk analysis of new oral anticoagulants for gastrointestinal bleeding and intracranial hemorrhage in atrial fibrillation patients: a systematic review and network meta-analysis J Zhejiang Univ Sci B. 2017 Jul;18(7):567-576. doi: 10.1631/jzus.B1600143.
585. Feng JR, Wang F, Qiu X, McFarland LV, Chen PF, Zhou R, Liu J, Zhao Q, Li J. Efficacy and safety of probiotic-supplemented triple therapy for eradication of Helicobacter pylori in children: a systematic review and network meta-analysis Eur J Clin Pharmacol. 2017 Oct;73(10):1199-1208. doi: 10.1007/s00228-017-2291-6. Epub 2017 Jul 5.
586. Tan PS, Bilger M, de Lima Lopes G, Acharyya S, Haaland B. Meta-analysis of first-line therapies with maintenance regimens for advanced non-small-cell lung cancer (NSCLC) in molecularly and clinically selected populations Cancer Med. 2017 Aug;6(8):1847-1860. doi: 10.1002/cam4.1101. Epub 2017 Jul 3.
587. Liu Y, Ma X, Zheng J, Jia J, Yan T. Effects of angiotensin-converting enzyme inhibitors and angiotensin receptor blockers on cardiovascular events and residual renal function in dialysis patients: a meta-analysis of randomised controlled trials BMC Nephrol. 2017 Jun 30;18(1):206. doi: 10.1186/s12882-017-0605-7.
588. Nevitt SJ, Sudell M, Weston J, Tudur Smith C, Marson AG. Antiepileptic drug monotherapy for epilepsy: a network meta-analysis of individual participant data Cochrane Database Syst Rev. 2017 Jun 29;6(6):CD011412. doi: 10.1002/14651858.CD011412.pub2.
589. Lee JY, Jeh SU, Kim MD, Kang DH, Kwon JK, Ham WS, Choi YD, Cho KS. Intraoperative and postoperative feasibility and safety of total tubeless, tubeless, small-bore tube, and standard percutaneous nephrolithotomy: a systematic review and network meta-analysis of 16 randomized controlled trials BMC Urol. 2017 Jun 27;17(1):48. doi: 10.1186/s12894-017-0239-x.
590. Yang YJ, Bang CS, Baik GH, Park TY, Shin SP, Suk KT, Kim DJ. Prokinetics for the treatment of functional dyspepsia: Bayesian network meta-analysis BMC Gastroenterol. 2017 Jun 26;17(1):83. doi: 10.1186/s12876-017-0639-0.
591. Fu X, Wu H, Li J, Wang C, Li M, Ma Q, Yang W. Efficacy of Drug Interventions for Chemotherapy-Induced Chronic Peripheral Neurotoxicity: A Network Meta-analysis Front Neurol. 2017 Jun 8;8:223. doi: 10.3389/fneur.2017.00223. eCollection 2017.
592. Fonseca MCM, Castro R, Machado M, Conte T, Girao MJBC. Uterine Artery Embolization and Surgical Methods for the Treatment of Symptomatic Uterine Leiomyomas: A Systemic Review and Meta-analysis Followed by Indirect Treatment Comparison Clin Ther. 2017 Jul;39(7):1438-1455.e2. doi: 10.1016/j.clinthera.2017.05.346. Epub 2017 Jun 20.
593. Westby MJ, Dumville JC, Soares MO, Stubbs N, Norman G. Dressings and topical agents for treating pressure ulcers Cochrane Database Syst Rev. 2017 Jun 22;6(6):CD011947. doi: 10.1002/14651858.CD011947.pub2.
594. Virgili G, Parravano M, Evans JR, Gordon I, Lucenteforte E. Anti-vascular endothelial growth factor for diabetic macular oedema: a network meta-analysis Cochrane Database Syst Rev. 2017 Jun 22;6(6):CD007419. doi: 10.1002/14651858.CD007419.pub5.
595. Ayers D, Kanters S, Goldgrub R, Hughes M, Kato R, Kragh N. Network meta-analysis of liraglutide versus dipeptidyl peptidase-4 inhibitors for the treatment of type 2 diabetes in Japanese patients Curr Med Res Opin. 2017 Sep;33(9):1653-1661. doi: 10.1080/03007995.2017.1345730. Epub 2017 Jul 19.
596. Feng JS, Li JY, Chen XY, Yang Z, Li SH. Strategies for Preventing Endoscopic Recurrence of Crohn's Disease 1 Year after Surgery: A Network Meta-Analysis Gastroenterol Res Pract. 2017;2017:7896160. doi: 10.1155/2017/7896160. Epub 2017 May 28.
597. Yu Y, Fang L, Zhang R, He J, Xiong Y, Guo X, Du Q, Huang Y, Sun Y. Comparative effectiveness of 9 ovulation-induction therapies in patients with clomiphene citrate-resistant polycystic ovary syndrome: a network meta-analysis Sci Rep. 2017 Jun 19;7(1):3812. doi: 10.1038/s41598-017-03803-9.
598. Xie T, Huang CY, Kang X, Luo JS, Qin XM, Han F. A Network Meta-Analysis of Short and Long-Term Efficacy of Targeted Therapy With Single or Double-Drug Regimens in the Treatment of Stage III/IV Malignant Melanoma Based on 16 Randomized Controlled Trials J Cell Biochem. 2018 Jan;119(1):640-649. doi: 10.1002/jcb.26225. Epub 2017 Jul 31.
599. Kennedy NA, Goodhand JR, Rampton DS. Editorial: which iron preparation for patients with IBD? Aliment Pharmacol Ther. 2017 Jul;46(2):194-195. doi: 10.1111/apt.14105.
600. Leonart LP, Tonin FS, Ferreira VL, Penteado STS, Wiens A, Motta FA, Pontarolo R. A network meta-analysis of primary prophylaxis for invasive fungal infection in haematological patients J Clin Pharm Ther. 2017 Oct;42(5):530-538. doi: 10.1111/jcpt.12579. Epub 2017 Jun 15.
601. Ballesio A, Aquino MRJV, Feige B, Johann AF, Kyle SD, Spiegelhalder K, Lombardo C, Rücker G, Riemann D, Baglioni C. The effectiveness of behavioural and cognitive behavioural therapies for insomnia on depressive and fatigue symptoms: A systematic review and network meta-analysis Sleep Med Rev. 2018 Feb;37:114-129. doi: 10.1016/j.smrv.2017.01.006. Epub 2017 Feb 7.
602. Liu GF, Li GJ, Zhao H. Efficacy and Toxicity of Different Chemotherapy Regimens in the Treatment of Advanced or Metastatic Pancreatic Cancer: A Network Meta-Analysis J Cell Biochem. 2018 Jan;119(1):511-523. doi: 10.1002/jcb.26210. Epub 2017 Jul 17.
603. Sun Q, Chang S, Lu S, Zhang Y, Chang Y. The Efficacy and Safety of 3 Types of Interventions for Stroke Prevention in Patients With Cardiovascular and Cerebrovascular Diseases: A Network Meta-analysis Clin Ther. 2017 Jul;39(7):1291-1312.e8. doi: 10.1016/j.clinthera.2017.04.008. Epub 2017 Jun 10.
604. Lee YH, Song GG. Comparative efficacy and safety of intravenous or subcutaneous belimumab in combination with standard therapy in patients with active systemic lupus erythematosus: a Bayesian network meta-analysis of randomized controlled trials Lupus. 2018 Jan;27(1):112-119. doi: 10.1177/0961203317713143. Epub 2017 Jun 7.
605. Ungaro RC, Colombel JF. Editorial: biologics in inflammatory bowel disease-time for direct comparisons Aliment Pharmacol Ther. 2017 Jul;46(1):68-69. doi: 10.1111/apt.14090.
606. Wang LG, Wang H, Liu Q, Hua WC, Li CM. A network meta-analysis for efficacy and safety of seven regimens in the treatment of type II diabetes Biomed Pharmacother. 2017 Aug;92:707-719. doi: 10.1016/j.biopha.2017.05.002. Epub 2017 Jun 3.
607. Veettil SK, Teerawattanapong N, Ching SM, Lim KG, Saokaew S, Phisalprapa P, Chaiyakunapruk N. Effects of chemopreventive agents on the incidence of recurrent colorectal adenomas: a systematic review with network meta-analysis of randomized controlled trials Onco Targets Ther. 2017 May 23;10:2689-2700. doi: 10.2147/OTT.S127335. eCollection 2017.
608. Zhuo C, Jiang R, Li G, Shao M, Chen C, Chen G, Tian H, Li J, Xue R, Jiang D. Efficacy and Tolerability of Second and Third Generation Anti-epileptic Drugs in Refractory Epilepsy: A Network Meta-Analysis Sci Rep. 2017 May 31;7(1):2535. doi: 10.1038/s41598-017-02525-2.
609. Bundy JD, Li C, Stuchlik P, Bu X, Kelly TN, Mills KT, He H, Chen J, Whelton PK, He J. Systolic Blood Pressure Reduction and Risk of Cardiovascular Disease and Mortality: A Systematic Review and Network Meta-analysis JAMA Cardiol. 2017 Jul 1;2(7):775-781. doi: 10.1001/jamacardio.2017.1421.
610. Zhuo C, Xue R, Luo L, Ji F, Tian H, Qu H, Lin X, Jiang R, Tao R. Efficacy of antidepressive medication for depression in Parkinson disease: a network meta-analysis Medicine (Baltimore). 2017 Jun;96(22):e6698. doi: 10.1097/MD.0000000000006698.
611. Kishi T, Ikuta T, Matsunaga S, Matsuda Y, Oya K, Iwata N. Comparative efficacy and safety of antipsychotics in the treatment of schizophrenia: a network meta-analysis in a Japanese population Neuropsychiatr Dis Treat. 2017 May 11;13:1281-1302. doi: 10.2147/NDT.S134340. eCollection 2017.
612. Wang F, Feng J, Chen P, Liu X, Ma M, Zhou R, Chang Y, Liu J, Li J, Zhao Q. Probiotics in Helicobacter pylori eradication therapy: Systematic review and network meta-analysis Clin Res Hepatol Gastroenterol. 2017 Sep;41(4):466-475. doi: 10.1016/j.clinre.2017.04.004. Epub 2017 May 25.
613. Ungprasert P, Erwin PJ, Koster MJ. Indirect comparisons of the efficacy of biological agents in patients with active ankylosing spondylitis: a systematic review and meta-analysis Clin Rheumatol. 2017 Jul;36(7):1569-1577. doi: 10.1007/s10067-017-3693-7. Epub 2017 May 28.
614. Lee G, Oh SW, Hwang SS, Yoon JW, Kang S, Joh HK, Kwon H, Kim J, Park D. Comparative effectiveness of oral antidiabetic drugs in preventing cardiovascular mortality and morbidity: A network meta-analysis PLoS One. 2017 May 25;12(5):e0177646. doi: 10.1371/journal.pone.0177646. eCollection 2017.
615. Zhang D, Wu J, Liu S, Zhang X, Zhang B. Network meta-analysis of Chinese herbal injections combined with the chemotherapy for the treatment of pancreatic cancer Medicine (Baltimore). 2017 May;96(21):e7005. doi: 10.1097/MD.0000000000007005.
616. Yu W, Sui W, Mu L, Yi W, Li H, Wei L, Yin W. Preventing necrotizing enterocolitis by food additives in neonates: A network meta-analysis revealing the efficacy and safety Medicine (Baltimore). 2017 May;96(21):e6652. doi: 10.1097/MD.0000000000006652.
617. Yang H, Wu X, Wang M. The Effect of Three Different Meditation Exercises on Hypertension: A Network Meta-Analysis Evid Based Complement Alternat Med. 2017;2017:9784271. doi: 10.1155/2017/9784271. Epub 2017 Apr 26.
618. Meng Z, Yu J, Acuff M, Luo C, Wang S, Yu L, Huang R. Tolerability of Opioid Analgesia for Chronic Pain: A Network Meta-Analysis Sci Rep. 2017 May 17;7(1):1995. doi: 10.1038/s41598-017-02209-x.
619. Li M, Song X, Zhu J, Fu A, Li J, Chen T. The interventional effect of new drugs combined with the Stupp protocol on glioblastoma: A network meta-analysis Clin Neurol Neurosurg. 2017 Aug;159:6-12. doi: 10.1016/j.clineuro.2017.05.015. Epub 2017 May 11.
620. Li K, Wang HT, He YK, Guo T. New idea for treatment strategies for Barcelona Clinic Liver Cancer stages based on a network meta-analysis Medicine (Baltimore). 2017 May;96(20):e6950. doi: 10.1097/MD.0000000000006950.
621. Gao XF, Zhang JJ, Jiang XM, Ge Z, Wang ZM, Li B, Mao WX, Chen SL. Targeted drugs for pulmonary arterial hypertension: a network meta-analysis of 32 randomized clinical trials Patient Prefer Adherence. 2017 May 8;11:871-885. doi: 10.2147/PPA.S133288. eCollection 2017.
622. Batson S, Mitchell SA, Windisch R, Damonte E, Munk VC, Reguart N. Tyrosine kinase inhibitor combination therapy in first-line treatment of non-small-cell lung cancer: systematic review and network meta-analysis Onco Targets Ther. 2017 May 5;10:2473-2482. doi: 10.2147/OTT.S134382. eCollection 2017.
623. Hilfiker R, Meichtry A, Eicher M, Nilsson Balfe L, Knols RH, Verra ML, Taeymans J. Exercise and other non-pharmaceutical interventions for cancer-related fatigue in patients during or after cancer treatment: a systematic review incorporating an indirect-comparisons meta-analysis Br J Sports Med. 2018 May;52(10):651-658. doi: 10.1136/bjsports-2016-096422. Epub 2017 May 13.
624. Wu HL, Marwah S, Wang P, Wang QM, Chen XW. Misoprostol for medical treatment of missed abortion: a systematic review and network meta-analysis Sci Rep. 2017 May 10;7(1):1664. doi: 10.1038/s41598-017-01892-0.
625. Giacoppo D, Gargiulo G, Buccheri S, Aruta P, Byrne RA, Cassese S, Dangas G, Kastrati A, Mehran R, Tamburino C, Capodanno D. Preventive Strategies for Contrast-Induced Acute Kidney Injury in Patients Undergoing Percutaneous Coronary Procedures: Evidence From a Hierarchical Bayesian Network Meta-Analysis of 124 Trials and 28 240 Patients Circ Cardiovasc Interv. 2017 May;10(5):e004383. doi: 10.1161/CIRCINTERVENTIONS.116.004383.
626. Singh JA, Hossain A, Mudano AS, Tanjong Ghogomu E, Suarez-Almazor ME, Buchbinder R, Maxwell LJ, Tugwell P, Wells GA. Biologics or tofacitinib for people with rheumatoid arthritis naive to methotrexate: a systematic review and network meta-analysis Cochrane Database Syst Rev. 2017 May 8;5(5):CD012657. doi: 10.1002/14651858.CD012657.
627. Montagnani F, Fornaro L, Frumento P, Vivaldi C, Falcone A, Fioretto L. Multimodality treatment of locally advanced squamous cell carcinoma of the oesophagus: A comprehensive review and network meta-analysis Crit Rev Oncol Hematol. 2017 Jun;114:24-32. doi: 10.1016/j.critrevonc.2017.03.024. Epub 2017 Mar 23.
628. Ren Z, Sun J, Sun X, Hou H, Li K, Ge Q. Efficacy and safety of different molecular targeted agents based on chemotherapy for gastric cancer patients treatment: a network meta-analysis Oncotarget. 2017 Jul 18;8(29):48253-48262. doi: 10.18632/oncotarget.17192.
629. Chong HY, Lai NM, Apisarnthanarak A, Chaiyakunapruk N. Comparative Efficacy of Antimicrobial Central Venous Catheters in Reducing Catheter-Related Bloodstream Infections in Adults: Abridged Cochrane Systematic Review and Network Meta-Analysis Clin Infect Dis. 2017 May 15;64(suppl_2):S131-S140. doi: 10.1093/cid/cix019.
630. Pyo JS, Kang G. Immunotherapy in advanced melanoma: a network meta-analysis Immunotherapy. 2017 May;9(6):471-479. doi: 10.2217/imt-2016-0143.
631. Yang XL, Duo-Ji MM, Long ZW. Efficacy and Safety of Single- or Double-Drug Antidiabetic Regimens in the Treatment of Type 2 Diabetes Mellitus: A Network Meta-Analysis J Cell Biochem. 2017 Dec;118(12):4536-4547. doi: 10.1002/jcb.26115. Epub 2017 Jun 13.
632. Huang Z, Liu H, Wang Y, Zhang C, Xu T. Determining optimal maintenance schedules for adjuvant intravesical bacillus Calmette-Guerin immunotherapy in non-muscle-invasive bladder cancer: a systematic review and network meta-analysis Curr Med Res Opin. 2017 Aug;33(8):1379-1387. doi: 10.1080/03007995.2017.1326889. Epub 2017 Jun 6.
633. da Silveira Nogueira Lima JP, Georgieva M, Haaland B, de Lima Lopes G. A systematic review and network meta-analysis of immunotherapy and targeted therapy for advanced melanoma Cancer Med. 2017 Jun;6(6):1143-1153. doi: 10.1002/cam4.1001. Epub 2017 May 1.
634. Zhang Y, Zhang Z, Huang X, Kang S, Chen G, Wu M, Miao S, Huang Y, Zhao H, Zhang L. Therapeutic Efficacy Comparison of 5 Major EGFR-TKIs in Advanced EGFR-positive Non-Small-cell Lung Cancer: A Network Meta-analysis Based on Head-to-Head Trials Clin Lung Cancer. 2017 Sep;18(5):e333-e340. doi: 10.1016/j.cllc.2016.09.006. Epub 2016 Oct 27.
635. Xie S, Zhang H, Wang X, Ge Q, Hu J. The relative efficacy and safety of targeted agents used in combination with chemotherapy in treating patients with untreated advanced gastric cancer: a network meta-analysis Oncotarget. 2017 Apr 18;8(16):26959-26968. doi: 10.18632/oncotarget.15923.
636. Jabbar-Lopez ZK, Yiu ZZN, Ward V, Exton LS, Mohd Mustapa MF, Samarasekera E, Burden AD, Murphy R, Owen CM, Parslew R, Venning V, Warren RB, Smith CH. Quantitative Evaluation of Biologic Therapy Options for Psoriasis: A Systematic Review and Network Meta-Analysis J Invest Dermatol. 2017 Aug;137(8):1646-1654. doi: 10.1016/j.jid.2017.04.009. Epub 2017 Apr 27.
637. Coward S, Kuenzig ME, Hazlewood G, Clement F, McBrien K, Holmes R, Panaccione R, Ghosh S, Seow CH, Rezaie A, Kaplan GG. Comparative Effectiveness of Mesalamine, Sulfasalazine, Corticosteroids, and Budesonide for the Induction of Remission in Crohn's Disease: A Bayesian Network Meta-analysis: Republished Inflamm Bowel Dis. 2017 May 1;23(5):E26-E37. doi: 10.1097/MIB.0000000000001158.
638. Zhao X, Zhou C, Ma J, Zhu Y, Sun M, Wang P, Zhang Y, Ma H, Zhang H. Efficacy and safety of rectal 5-aminosalicylic acid versus corticosteroids in active distal ulcerative colitis: a systematic review and network meta-analysis Sci Rep. 2017 Apr 25;7:46693. doi: 10.1038/srep46693.
639. Orme ME, Nguyen H, Lu JY, Thomas SA. Comparative effectiveness of glycemic control in patients with type 2 diabetes treated with GLP-1 receptor agonists: a network meta-analysis of placebo-controlled and active-comparator trials Diabetes Metab Syndr Obes. 2017 Mar 29;10:111-122. doi: 10.2147/DMSO.S116810. eCollection 2017.
640. Arirachakaran A, Amphansap T, Thanindratarn P, Piyapittayanun P, Srisawat P, Kongtharvonskul J. Comparative outcome of PFNA, Gamma nails, PCCP, Medoff plate, LISS and dynamic hip screws for fixation in elderly trochanteric fractures: a systematic review and network meta-analysis of randomized controlled trials Eur J Orthop Surg Traumatol. 2017 Oct;27(7):937-952. doi: 10.1007/s00590-017-1964-2. Epub 2017 Apr 22.
641. Bonovas S, Peyrin-Biroulet L, Danese S. Is Mesalamine Effective for the Induction of Remission in Crohn's Disease? Inflamm Bowel Dis. 2017 May;23(5):E22-E23. doi: 10.1097/MIB.0000000000001124.
642. Jung YS, Park CH, Park JH, Nam E, Lee HL. Efficacy of Helicobacter pylori eradication therapies in Korea: A systematic review and network meta-analysis Helicobacter. 2017 Aug;22(4). doi: 10.1111/hel.12389. Epub 2017 Apr 19.
643. Shi K, Shen Z, Zhu G, Meng F, Gu M, Ji F. Systematic review with network meta-analysis: dual therapy for high-risk bleeding peptic ulcers BMC Gastroenterol. 2017 Apr 19;17(1):55. doi: 10.1186/s12876-017-0610-0.
644. Li S, Liu H, Diao C, Wang X, Gao M, Li Z, Song L, Gao X, Han J, Wang F, Li W, Han X. Prognosis of surgery combined with different adjuvant therapies in esophageal cancer treatment: a network meta-analysis Oncotarget. 2017 May 30;8(22):36339-36353. doi: 10.18632/oncotarget.16193.
645. Song GM, Liu XL, Bian W, Wu J, Deng YH, Zhang H, Tian X. Systematic review with network meta-analysis: comparative efficacy of different enteral immunonutrition formulas in patients underwent gastrectomy Oncotarget. 2017 Apr 4;8(14):23376-23388. doi: 10.18632/oncotarget.15580.
646. He J, Wu P, Tang Y, Liu S, Xie C, Luo S, Zeng J, Xu J, Zhao S. Chemoradiotherapy enhanced the efficacy of radiotherapy in nasopharyngeal carcinoma patients: a network meta-analysis Oncotarget. 2017 Jun 13;8(24):39782-39794. doi: 10.18632/oncotarget.16349.
647. Wa D, Zhu P, Long Z. Comparative efficacy and safety of antiplatelet agents in cerebral ischemic disease: A network meta-analysis J Cell Biochem. 2019 Jun;120(6):8919-8934. doi: 10.1002/jcb.26065. Epub 2019 Mar 10.
648. Wheat CL, Ko CW, Clark-Snustad K, Grembowski D, Thornton TA, Devine B. Inflammatory Bowel Disease (IBD) pharmacotherapy and the risk of serious infection: a systematic review and network meta-analysis BMC Gastroenterol. 2017 Apr 14;17(1):52. doi: 10.1186/s12876-017-0602-0.
649. Facciorusso A, Chandar AK, Murad MH, Prokop LJ, Muscatiello N, Kamath PS, Singh S. Comparative efficacy of pharmacological strategies for management of type 1 hepatorenal syndrome: a systematic review and network meta-analysis Lancet Gastroenterol Hepatol. 2017 Feb;2(2):94-102. doi: 10.1016/S2468-1253(16)30157-1. Epub 2016 Dec 2.
650. Wu YC, Tsai WC, Tu YK, Yu TY. Comparative Effectiveness of Nonoperative Treatments for Chronic Calcific Tendinitis of the Shoulder: A Systematic Review and Network Meta-Analysis of Randomized Controlled Trials Arch Phys Med Rehabil. 2017 Aug;98(8):1678-1692.e6. doi: 10.1016/j.apmr.2017.02.030. Epub 2017 Apr 8.
651. Pease C, Hutton B, Yazdi F, Wolfe D, Hamel C, Quach P, Skidmore B, Moher D, Alvarez GG. Efficacy and completion rates of rifapentine and isoniazid (3HP) compared to other treatment regimens for latent tuberculosis infection: a systematic review with network meta-analyses BMC Infect Dis. 2017 Apr 11;17(1):265. doi: 10.1186/s12879-017-2377-x.
652. Ebinger SM, Hardt J, Warschkow R, Schmied BM, Herold A, Post S, Marti L. Operative and medical treatment of chronic anal fissures-a review and network meta-analysis of randomized controlled trials J Gastroenterol. 2017 Jun;52(6):663-676. doi: 10.1007/s00535-017-1335-0. Epub 2017 Apr 10.
653. Shah R, Rashid A, Hwang I, Fan TM, Khouzam RN, Reed GL. Meta-Analysis of the Relative Efficacy and Safety of Oral P2Y12 Inhibitors in Patients With Acute Coronary Syndrome Am J Cardiol. 2017 Jun 1;119(11):1723-1728. doi: 10.1016/j.amjcard.2017.03.011. Epub 2017 Mar 16.
654. Zaccara G, Giovannelli F, Giorgi FS, Franco V, Gasparini S, Benedetto U. Tolerability of new antiepileptic drugs: a network meta-analysis Eur J Clin Pharmacol. 2017 Jul;73(7):811-817. doi: 10.1007/s00228-017-2245-z. Epub 2017 Apr 4.
655. Bergrath E, Gerber RA, Gruben D, Lukic T, Makin C, Wallenstein G. Tofacitinib versus Biologic Treatments in Moderate-to-Severe Rheumatoid Arthritis Patients Who Have Had an Inadequate Response to Nonbiologic DMARDs: Systematic Literature Review and Network Meta-Analysis Int J Rheumatol. 2017;2017:8417249. doi: 10.1155/2017/8417249. Epub 2017 Mar 9.
656. Zhuo C, Zhu X, Jiang R, Ji F, Su Z, Xue R, Zhou Y. Comparison for Efficacy and Tolerability among Ten Drugs for Treatment of Parkinson's Disease: A Network Meta-Analysis Sci Rep. 2017 Apr 4;8:45865. doi: 10.1038/srep45865.
657. Chierrito de Oliveira D, Guerrero de Sousa P, Borges Dos Reis C, Tonin FS, Maria Steimbach L, Virtuoso S, Fernandez-Llimos F, Pontarolo R, Cristina Conegero Sanches A. Safety of Treatments for ADHD in Adults: Pairwise and Network Meta-Analyses J Atten Disord. 2019 Jan;23(2):111-120. doi: 10.1177/1087054717696773. Epub 2017 Apr 3.
658. van Nooten F, Treur M, Pantiri K, Stoker M, Charokopou M. Capsaicin 8% Patch Versus Oral Neuropathic Pain Medications for the Treatment of Painful Diabetic Peripheral Neuropathy: A Systematic Literature Review and Network Meta-analysis Clin Ther. 2017 Apr;39(4):787-803.e18. doi: 10.1016/j.clinthera.2017.02.010. Epub 2017 Mar 30.
659. Rodríguez-Perálvarez M, Guerrero-Misas M, Thorburn D, Davidson BR, Tsochatzis E, Gurusamy KS. Maintenance immunosuppression for adults undergoing liver transplantation: a network meta-analysis Cochrane Database Syst Rev. 2017 Mar 31;3(3):CD011639. doi: 10.1002/14651858.CD011639.pub2.
660. Wang X, Zheng H, Shou T, Tang C, Miao K, Wang P. Effectiveness of multi-drug regimen chemotherapy treatment in osteosarcoma patients: a network meta-analysis of randomized controlled trials J Orthop Surg Res. 2017 Mar 29;12(1):52. doi: 10.1186/s13018-017-0544-9.
661. Gibbison B, López-López JA, Higgins JP, Miller T, Angelini GD, Lightman SL, Annane D. Corticosteroids in septic shock: a systematic review and network meta-analysis Crit Care. 2017 Mar 28;21(1):78. doi: 10.1186/s13054-017-1659-4.
662. Wen D, McAlinden C, Flitcroft I, Tu R, Wang Q, Alió J, Marshall J, Huang Y, Song B, Hu L, Zhao Y, Zhu S, Gao R, Bao F, Yu A, Yu Y, Lian H, Huang J. Postoperative Efficacy, Predictability, Safety, and Visual Quality of Laser Corneal Refractive Surgery: A Network Meta-analysis Am J Ophthalmol. 2017 Jun;178:65-78. doi: 10.1016/j.ajo.2017.03.013. Epub 2017 Mar 20.
663. Wang T, Xu C, Pan K, Xiong H. Acupuncture and moxibustion for chronic fatigue syndrome in traditional Chinese medicine: a systematic review and meta-analysis BMC Complement Altern Med. 2017 Mar 23;17(1):163. doi: 10.1186/s12906-017-1647-x.
664. Zhu Y, Zhang T, Li H, Yang Y, Chen Q, Kong L, Tai B. Discovering the Relative Efficacy of Inhaled Medications for Chronic Obstructive Pulmonary Disease: Multiple Treatment Comparisons Cell Physiol Biochem. 2017;41(4):1532-1546. doi: 10.1159/000470818. Epub 2017 Mar 27.
665. Popat S, Mellemgaard A, Reck M, Hastedt C, Griebsch I. Nintedanib plus docetaxel as second-line therapy in patients with non-small-cell lung cancer of adenocarcinoma histology: a network meta-analysis vs new therapeutic options Future Oncol. 2017 Jun;13(13):1159-1171. doi: 10.2217/fon-2016-0493. Epub 2017 Feb 27.
666. Schubert A, Buchholt AT, El Khoury AC, Kamal A, Taieb V. Evaluating the costs of glycemic response with canagliflozin versus dapagliflozin and empagliflozin as add-on to metformin in patients with type 2 diabetes mellitus in the United Arab Emirates Curr Med Res Opin. 2017 Jun;33(6):1155-1163. doi: 10.1080/03007995.2017.1310091. Epub 2017 Apr 28.
667. Zhang Z, Chen K, Ni H, Zhang X, Fan H. Sedation of mechanically ventilated adults in intensive care unit: a network meta-analysis Sci Rep. 2017 Mar 21;7:44979. doi: 10.1038/srep44979.
668. Fanaroff AC, Hasselblad V, Roe MT, Bhatt DL, James SK, Steg PG, Gibson CM, Ohman EM. Antithrombotic agents for secondary prevention after acute coronary syndromes: A systematic review and network meta-analysis Int J Cardiol. 2017 Aug 15;241:87-96. doi: 10.1016/j.ijcard.2017.03.046. Epub 2017 Mar 14.
669. Wu S, Gao YJ, Ge ZZ. Optimal use of polyethylene glycol for preparation of small bowel video capsule endoscopy: a network meta-analysis Curr Med Res Opin. 2017 Jun;33(6):1149-1154. doi: 10.1080/03007995.2017.1308922. Epub 2017 Apr 13.
670. Zhao X, Zhou C, Chen H, Ma J, Zhu Y, Wang P, Zhang Y, Ma H, Zhang H. Efficacy and safety of medical therapy for low bone mineral density in patients with Crohn disease: A systematic review with network meta-analysis Medicine (Baltimore). 2017 Mar;96(11):e6378. doi: 10.1097/MD.0000000000006378.
671. Ilfeld BM, McCartney CJL. Searching for the Optimal Pain Management Technique after Knee Arthroplasty: Analgesia Is Just the Tip of the Iceberg Anesthesiology. 2017 May;126(5):768-770. doi: 10.1097/ALN.0000000000001608.
672. Fleetwood K, McCool R, Glanville J, Edwards SC, Gsteiger S, Daigl M, Fisher M. Systematic Review and Network Meta-analysis of Idiopathic Pulmonary Fibrosis Treatments J Manag Care Spec Pharm. 2017 Mar;23(3-b Suppl):S5-S16. doi: 10.18553/jmcp.2017.23.3-b.s5.
673. Boehm BE, Cornell JE, Wang H, Mukherjee N, Oppenheimer JS, Svatek RS. Efficacy of bacillus Calmette-Guérin Strains for Treatment of Nonmuscle Invasive Bladder Cancer: A Systematic Review and Network Meta-Analysis J Urol. 2017 Sep;198(3):503-510. doi: 10.1016/j.juro.2017.01.086. Epub 2017 Mar 10.
674. Leonart LP, Tonin FS, Ferreira VL, Tavares da Silva Penteado S, de Araújo Motta F, Pontarolo R. Fluconazole Doses Used for Prophylaxis of Invasive Fungal Infection in Neonatal Intensive Care Units: A Network Meta-Analysis J Pediatr. 2017 Jun;185:129-135.e6. doi: 10.1016/j.jpeds.2017.02.039. Epub 2017 Mar 10.
675. Singh JA, Hossain A, Tanjong Ghogomu E, Mudano AS, Maxwell LJ, Buchbinder R, Lopez-Olivo MA, Suarez-Almazor ME, Tugwell P, Wells GA. Biologics or tofacitinib for people with rheumatoid arthritis unsuccessfully treated with biologics: a systematic review and network meta-analysis Cochrane Database Syst Rev. 2017 Mar 10;3(3):CD012591. doi: 10.1002/14651858.CD012591.
676. Wu C, Zhou X, Miao C, Zhang J, Tang Q, Chang X, Ni H. Assessing the Feasibility of Replacing Standard-Dose Bacillus Calmette-Guérin Immunotherapy with Other Intravesical Instillation Therapies in Bladder Cancer Patients: A Network Meta-Analysis Cell Physiol Biochem. 2017;41(4):1298-1312. doi: 10.1159/000464432. Epub 2017 Mar 8.
677. Sarri G, Pedder H, Dias S, Guo Y, Lumsden MA. Vasomotor symptoms resulting from natural menopause: a systematic review and network meta-analysis of treatment effects from the National Institute for Health and Care Excellence guideline on menopause BJOG. 2017 Sep;124(10):1514-1523. doi: 10.1111/1471-0528.14619. Epub 2017 May 11.
678. Jarde A, Lutsiv O, Park CK, Beyene J, Dodd JM, Barrett J, Shah PS, Cook JL, Saito S, Biringer AB, Sabatino L, Giglia L, Han Z, Staub K, Mundle W, Chamberlain J, McDonald SD. Effectiveness of progesterone, cerclage and pessary for preventing preterm birth in singleton pregnancies: a systematic review and network meta-analysis BJOG. 2017 Jul;124(8):1176-1189. doi: 10.1111/1471-0528.14624. Epub 2017 Apr 17.
679. Kay S, Strickson A, Puelles J, Selby R, Benson E, Tolley K. Comparative Effectiveness of Adding Alogliptin to Metformin Plus Sulfonylurea with Other DPP-4 Inhibitors in Type 2 Diabetes: A Systematic Review and Network Meta-Analysis Diabetes Ther. 2017 Apr;8(2):251-273. doi: 10.1007/s13300-017-0245-8. Epub 2017 Mar 8.
680. Makai P, IntHout J, Deinum J, Jenniskens K, Wilt GJV. A Network Meta-Analysis of Clinical Management Strategies for Treatment-Resistant Hypertension: Making Optimal Use of the Evidence J Gen Intern Med. 2017 Aug;32(8):921-930. doi: 10.1007/s11606-017-4000-7. Epub 2017 Mar 8.
681. Gunst J, Van den Berghe G. Blood glucose control in the ICU: how tight? Ann Transl Med. 2017 Feb;5(4):76. doi: 10.21037/atm.2017.01.45.
682. Xing F, Chen J, Zhao B, Jiang J, Tang A, Chen Y. Real role of β-blockers in regression of left ventricular mass in hypertension patients: Bayesian network meta-analysis Medicine (Baltimore). 2017 Mar;96(10):e6290. doi: 10.1097/MD.0000000000006290.
683. Woods B, Manca A, Weatherly H, Saramago P, Sideris E, Giannopoulou C, Rice S, Corbett M, Vickers A, Bowes M, MacPherson H, Sculpher M. Cost-effectiveness of adjunct non-pharmacological interventions for osteoarthritis of the knee PLoS One. 2017 Mar 7;12(3):e0172749. doi: 10.1371/journal.pone.0172749. eCollection 2017.
684. Ostacher M, Ng-Mak D, Patel P, Ntais D, Schlueter M, Loebel A. Lurasidone compared to other atypical antipsychotic monotherapies for bipolar depression: A systematic review and network meta-analysis World J Biol Psychiatry. 2018 Dec;19(8):586-601. doi: 10.1080/15622975.2017.1285050. Epub 2017 Mar 7.
685. Chen Y, Chen L, Zhong D. Comparing the adverse effects of platinum in combination with etoposide or irinotecan in previously untreated small-cell lung cancer patients with extensive disease: A network meta-analyses Thorac Cancer. 2017 May;8(3):170-180. doi: 10.1111/1759-7714.12420. Epub 2017 Mar 6.
686. Joseph A, Ayyagari R, Xie M, Cai S, Xie J, Huss M, Sikirica V. Comparative efficacy and safety of attention-deficit/hyperactivity disorder pharmacotherapies, including guanfacine extended release: a mixed treatment comparison Eur Child Adolesc Psychiatry. 2017 Aug;26(8):875-897. doi: 10.1007/s00787-017-0962-6. Epub 2017 Mar 3.
687. Sekercioglu N, Angeliki Veroniki A, Thabane L, Busse JW, Akhtar-Danesh N, Iorio A, Cruz Lopes L, Guyatt GH. Effects of different phosphate lowering strategies in patients with CKD on laboratory outcomes: A systematic review and NMA PLoS One. 2017 Mar 1;12(3):e0171028. doi: 10.1371/journal.pone.0171028. eCollection 2017.
688. van Beurden-Tan CHY, Franken MG, Blommestein HM, Uyl-de Groot CA, Sonneveld P. Systematic Literature Review and Network Meta-Analysis of Treatment Outcomes in Relapsed and/or Refractory Multiple Myeloma J Clin Oncol. 2017 Apr 20;35(12):1312-1319. doi: 10.1200/JCO.2016.71.1663. Epub 2017 Feb 27.
689. Tang H, Li D, Zhang J, Li Y, Wang T, Zhai S, Song Y. Sodium-glucose co-transporter-2 inhibitors and risk of adverse renal outcomes among patients with type 2 diabetes: A network and cumulative meta-analysis of randomized controlled trials Diabetes Obes Metab. 2017 Aug;19(8):1106-1115. doi: 10.1111/dom.12917. Epub 2017 Mar 31.
690. Palmer SC, Tunnicliffe DJ, Singh-Grewal D, Mavridis D, Tonelli M, Johnson DW, Craig JC, Tong A, Strippoli GFM. Induction and Maintenance Immunosuppression Treatment of Proliferative Lupus Nephritis: A Network Meta-analysis of Randomized Trials Am J Kidney Dis. 2017 Sep;70(3):324-336. doi: 10.1053/j.ajkd.2016.12.008. Epub 2017 Feb 21.
691. Duo-Ji MM, Long ZW. Comparative efficacy and acceptability of endothelin receptor antagonists for pulmonary arterial hypertension: A network meta-analysis Int J Cardiol. 2017 May 1;234:90-98. doi: 10.1016/j.ijcard.2016.12.092. Epub 2017 Jan 5.
692. Elgendy IY, Mahmoud AN, Kumbhani DJ, Bhatt DL, Bavry AA. Complete or Culprit-Only Revascularization for Patients With Multivessel Coronary Artery Disease Undergoing Percutaneous Coronary Intervention: A Pairwise and Network Meta-Analysis of Randomized Trials JACC Cardiovasc Interv. 2017 Feb 27;10(4):315-324. doi: 10.1016/j.jcin.2016.11.047.
693. Yang Y, Xu H. Comparing six antihypertensive medication classes for preventing new-onset diabetes mellitus among hypertensive patients: a network meta-analysis J Cell Mol Med. 2017 Sep;21(9):1742-1750. doi: 10.1111/jcmm.13096. Epub 2017 Feb 23.
694. Wang J, Xu H, Wang D, Li M. Comparison of Pathogen Eradication Rate and Safety of Anti-Bacterial Agents for Bronchitis: A Network Meta-Analysis J Cell Biochem. 2017 Oct;118(10):3171-3183. doi: 10.1002/jcb.25951. Epub 2017 Jun 16.
695. Obloza A, Kirby J, Yates D, Toozs-Hobson P. Indirect treatment comparison (ITC) of medical therapies for an overactive bladder Neurourol Urodyn. 2017 Sep;36(7):1824-1831. doi: 10.1002/nau.23189. Epub 2017 Feb 21.
696. He A, Song D, Zhang L, Li C. Unveiling the relative efficacy, safety and tolerability of prophylactic medications for migraine: pairwise and network-meta analysis J Headache Pain. 2017 Dec;18(1):26. doi: 10.1186/s10194-017-0720-7. Epub 2017 Feb 20.
697. Pagsberg AK, Tarp S, Glintborg D, Stenstrøm AD, Fink-Jensen A, Correll CU, Christensen R. Acute Antipsychotic Treatment of Children and Adolescents With Schizophrenia-Spectrum Disorders: A Systematic Review and Network Meta-Analysis J Am Acad Child Adolesc Psychiatry. 2017 Mar;56(3):191-202. doi: 10.1016/j.jaac.2016.12.013. Epub 2016 Dec 29.
698. Chen K, Wang Q, Pleasants RA, Ge L, Liu W, Peng K, Zhai S. Empiric treatment against invasive fungal diseases in febrile neutropenic patients: a systematic review and network meta-analysis BMC Infect Dis. 2017 Feb 20;17(1):159. doi: 10.1186/s12879-017-2263-6.
699. Iftikhar IH, Bittencourt L, Youngstedt SD, Ayas N, Cistulli P, Schwab R, Durkin MW, Magalang UJ. Comparative efficacy of CPAP, MADs, exercise-training, and dietary weight loss for sleep apnea: a network meta-analysis Sleep Med. 2017 Feb;30:7-14. doi: 10.1016/j.sleep.2016.06.001. Epub 2016 Jun 28.
700. Hanif H, Belley-Cote EP, Alotaibi A, Dvirnik N, Neupane B, Beyene J, Eikelboom JW, Holmes D, Whitlock RP. Left atrial appendage occlusion for stroke prevention in patients with atrial fibrillation: a systematic review and network meta-analysis of randomized controlled trials J Cardiovasc Surg (Torino). 2018 Feb;59(1):128-139. doi: 10.23736/S0021-9509.17.09824-X. Epub 2017 Feb 17.
701. Zhao T, Feng X, Liu J, Gao J, Zhou C. Evaluate the Efficacy and Safety of Anti-Epileptic Medications for Partial Seizures of Epilepsy: A Network Meta-Analysis J Cell Biochem. 2017 Sep;118(9):2850-2864. doi: 10.1002/jcb.25936. Epub 2017 Apr 27.
702. Chatterton ML, Stockings E, Berk M, Barendregt JJ, Carter R, Mihalopoulos C. Psychosocial therapies for the adjunctive treatment of bipolar disorder in adults: network meta-analysis Br J Psychiatry. 2017 May;210(5):333-341. doi: 10.1192/bjp.bp.116.195321. Epub 2017 Feb 16.
703. Sandhu SS, Piepho HP, Khehra HS. Comparing the effectiveness profile of pharmacological interventions used for orthodontic pain relief: an arm-based multilevel network meta-analysis of longitudinal data Eur J Orthod. 2017 Nov 30;39(6):601-614. doi: 10.1093/ejo/cjw088.
704. Stevenson MD, Wailoo AJ, Tosh JC, Hernandez-Alava M, Gibson LA, Stevens JW, Archer RJ, Simpson EL, Hock ES, Young A, Scott DL. The Cost-effectiveness of Sequences of Biological Disease-modifying Antirheumatic Drug Treatment in England for Patients with Rheumatoid Arthritis Who Can Tolerate Methotrexate J Rheumatol. 2017 Jul;44(7):973-980. doi: 10.3899/jrheum.160941. Epub 2017 Feb 15.
705. Pasquali S, Chiarion-Sileni V, Rossi CR, Mocellin S. Immune checkpoint inhibitors and targeted therapies for metastatic melanoma: A network meta-analysis Cancer Treat Rev. 2017 Mar;54:34-42. doi: 10.1016/j.ctrv.2017.01.006. Epub 2017 Feb 2.
706. Chen C, Zhang X, Ma X. Durability of cervical disc arthroplasties and its influence factors: A systematic review and a network meta-analysis Medicine (Baltimore). 2017 Feb;96(6):e5947. doi: 10.1097/MD.0000000000005947.
707. Donohue JF, Betts KA, Du EX, Altman P, Goyal P, Keininger DL, Gruenberger JB, Signorovitch JE. Comparative efficacy of long-acting β2-agonists as monotherapy for chronic obstructive pulmonary disease: a network meta-analysis Int J Chron Obstruct Pulmon Dis. 2017 Jan 19;12:367-381. doi: 10.2147/COPD.S119908. eCollection 2017.
708. Navarese EP, Gurbel PA, Andreotti F, Kołodziejczak MM, Palmer SC, Dias S, Buffon A, Kubica J, Kowalewski M, Jadczyk T, Laskiewicz M, Jędrzejek M, Brockmeyer M, Airoldi F, Ruospo M, De Servi S, Wojakowski W, O' Connor C, Strippoli GF. Prevention of contrast-induced acute kidney injury in patients undergoing cardiovascular procedures-a systematic review and network meta-analysis PLoS One. 2017 Feb 2;12(2):e0168726. doi: 10.1371/journal.pone.0168726. eCollection 2017.
709. Wang JH, Sun T. Comparison of effects of seven treatment methods for distal radius fracture on minimizing complex regional pain syndrome Arch Med Sci. 2017 Feb 1;13(1):163-173. doi: 10.5114/aoms.2016.59794. Epub 2016 May 11.
710. Wang R, Kim BV, van Wely M, Johnson NP, Costello MF, Zhang H, Ng EH, Legro RS, Bhattacharya S, Norman RJ, Mol BW. Treatment strategies for women with WHO group II anovulation: systematic review and network meta-analysis BMJ. 2017 Jan 31;356:j138. doi: 10.1136/bmj.j138.
711. Hu ZH, Shi AM, Hu DM, Bao JJ. Efficacy of proton pump inhibitors for patients with duodenal ulcers: A pairwise and network meta-analysis of randomized controlled trials Saudi J Gastroenterol. 2017 Jan-Feb;23(1):11-19. doi: 10.4103/1319-3767.199117.
712. Duan P, Liu Y, Li J. The comparative efficacy and safety of topical non-steroidal anti-inflammatory drugs for the treatment of anterior chamber inflammation after cataract surgery: a systematic review and network meta-analysis Graefes Arch Clin Exp Ophthalmol. 2017 Apr;255(4):639-649. doi: 10.1007/s00417-017-3599-8. Epub 2017 Jan 27.
713. Boonchan T, Wilasrusmee C, McEvoy M, Attia J, Thakkinstian A. Network meta-analysis of antibiotic prophylaxis for prevention of surgical-site infection after groin hernia surgery Br J Surg. 2017 Jan;104(2):e106-e117. doi: 10.1002/bjs.10441.
714. Bangalore S, Toklu B, Gianos E, Schwartzbard A, Weintraub H, Ogedegbe G, Messerli FH. Optimal Systolic Blood Pressure Target After SPRINT: Insights from a Network Meta-Analysis of Randomized Trials Am J Med. 2017 Jun;130(6):707-719.e8. doi: 10.1016/j.amjmed.2017.01.004. Epub 2017 Jan 19.
715. Bai Z, Wang G, Cai S, Ding X, Liu W, Huang D, Shen W, Zhang J, Chen K, Yang Y, Zhang L, Zhao X, Ouyang Q, Zhao J, Lu H, Hao W. Efficacy, acceptability and tolerability of 8 atypical antipsychotics in Chinese patients with acute schizophrenia: A network meta-analysis Schizophr Res. 2017 Jul;185:73-79. doi: 10.1016/j.schres.2017.01.002. Epub 2017 Jan 17.
716. Xing T, Xu H, Cao L, Ye M. HBeAg Seroconversion in HBeAg-Positive Chronic Hepatitis B Patients Receiving Long-Term Nucleos(t)ide Analog Treatment: A Systematic Review and Network Meta-Analysis PLoS One. 2017 Jan 20;12(1):e0169444. doi: 10.1371/journal.pone.0169444. eCollection 2017.
717. Johnston R, Uthman O, Cummins E, Clar C, Royle P, Colquitt J, Tan BK, Clegg A, Shantikumar S, Court R, O'Hare JP, McGrane D, Holt T, Waugh N. Canagliflozin, dapagliflozin and empagliflozin monotherapy for treating type 2 diabetes: systematic review and economic evaluation Health Technol Assess. 2017 Jan;21(2):1-218. doi: 10.3310/hta21020.
718. Kapoor A, Ellis A, Shaffer N, Gurwitz J, Chandramohan A, Saulino J, Ishak A, Okubanjo T, Michota F, Hylek E, Trikalinos TA. Comparative effectiveness of venous thromboembolism prophylaxis options for the patient undergoing total hip and knee replacement: a network meta-analysis J Thromb Haemost. 2017 Feb;15(2):284-294. doi: 10.1111/jth.13566. Epub 2017 Jan 19.
719. Zhang C, Kwong JS, Yuan RX, Chen H, Xu C, Wang YP, Yang GL, Yan JZ, Peng L, Zeng XT, Weng H, Luo J, Niu YM. Effectiveness and Tolerability of Different Recommended Doses of PPIs and H(2)RAs in GERD: Network Meta-Analysis and GRADE system Sci Rep. 2017 Jan 19;7:41021. doi: 10.1038/srep41021.
720. Duo-Ji MM, Ci-Ren BS, Long ZW, Zhang XH, Luo DL. Short-term efficacy of different chemotherapy regimens in the treatment of advanced gastric cancer: a network meta-analysis Oncotarget. 2017 Jun 6;8(23):37896-37911. doi: 10.18632/oncotarget.14664.
721. Lee YH, Bae SC. Comparative efficacy and safety of baricitinib 2 mg and 4 mg in patients with active rheumatoid arthritis : A Bayesian network meta-analysis of randomized controlled trials Z Rheumatol. 2018 May;77(4):335-342. doi: 10.1007/s00393-016-0254-4.
722. Suissa K, Larivière J, Eisenberg MJ, Eberg M, Gore GC, Grad R, Joseph L, Reynier PM, Filion KB. Efficacy and Safety of Smoking Cessation Interventions in Patients With Cardiovascular Disease: A Network Meta-Analysis of Randomized Controlled Trials Circ Cardiovasc Qual Outcomes. 2017 Jan;10(1):e002458. doi: 10.1161/CIRCOUTCOMES.115.002458.
723. Westman PC, Lipinski MJ, Torguson R, Waksman R. A comparison of cangrelor, prasugrel, ticagrelor, and clopidogrel in patients undergoing percutaneous coronary intervention: A network meta-analysis Cardiovasc Revasc Med. 2017 Mar;18(2):79-85. doi: 10.1016/j.carrev.2016.10.005. Epub 2016 Oct 21.
724. Burnett H, Earley A, Voors AA, Senni M, McMurray JJ, Deschaseaux C, Cope S. Thirty Years of Evidence on the Efficacy of Drug Treatments for Chronic Heart Failure With Reduced Ejection Fraction: A Network Meta-Analysis Circ Heart Fail. 2017 Jan;10(1):e003529. doi: 10.1161/CIRCHEARTFAILURE.116.003529.
725. Qu CP, Sun GX, Yang SQ, Tian J, Si JG, Wang YF. Toxicities of different first-line chemotherapy regimens in the treatment of advanced ovarian cancer: A network meta-analysis Medicine (Baltimore). 2017 Jan;96(2):e5797. doi: 10.1097/MD.0000000000005797.
726. Park SK, Lee MY, Jang EJ, Kim HL, Ha DM, Lee EK. A comparison of discontinuation rates of tofacitinib and biologic disease-modifying anti-rheumatic drugs in rheumatoid arthritis: a systematic review and Bayesian network meta-analysis Clin Exp Rheumatol. 2017 Jul-Aug;35(4):689-699. Epub 2017 Jan 5.
727. Yang M, He M, Zhao M, Zou B, Liu J, Luo LM, Li QL, He JH, Lei PG. Proton pump inhibitors for preventing non-steroidal anti-inflammatory drug induced gastrointestinal toxicity: a systematic review Curr Med Res Opin. 2017 Jun;33(6):973-980. doi: 10.1080/03007995.2017.1281110. Epub 2017 Jan 25.
728. McCool R, Gould IM, Eales J, Barata T, Arber M, Fleetwood K, Glanville J, Kauf TL. Systematic review and network meta-analysis of tedizolid for the treatment of acute bacterial skin and skin structure infections caused by MRSA BMC Infect Dis. 2017 Jan 7;17(1):39. doi: 10.1186/s12879-016-2100-3.
729. Naing C, Reid SA, Aung K. Comparing antibiotic treatment for leptospirosis using network meta-analysis: a tutorial BMC Infect Dis. 2017 Jan 5;17(1):29. doi: 10.1186/s12879-016-2145-3.
730. Liu X, Chen J, He YA, Meng X, Li K, He CK, Liu S. Comparing efficacy and survivals of initial treatments for elderly patients with newly diagnosed multiple myeloma: a network meta-analysis of randomized controlled trials Onco Targets Ther. 2016 Dec 22;10:121-128. doi: 10.2147/OTT.S123680. eCollection 2017.
731. Amaral MA, Guedes GHBF, Epifanio M, Wagner MB, Jones MH, Mattiello R. Network meta-analysis of probiotics to prevent respiratory infections in children and adolescents Pediatr Pulmonol. 2017 Jun;52(6):833-843. doi: 10.1002/ppul.23643. Epub 2017 Jan 3.
732. Huang R, Feng Y, Wang Y, Qin X, Melgiri ND, Sun Y, Li X. Comparative Efficacy and Safety of Antihypertensive Agents for Adult Diabetic Patients with Microalbuminuric Kidney Disease: A Network Meta-Analysis PLoS One. 2017 Jan 3;12(1):e0168582. doi: 10.1371/journal.pone.0168582. eCollection 2017.
733. Rinciog C, Watkins M, Chang S, Maher TM, LeReun C, Esser D, Diamantopoulos A. A Cost-Effectiveness Analysis of Nintedanib in Idiopathic Pulmonary Fibrosis in the UK Pharmacoeconomics. 2017 Apr;35(4):479-491. doi: 10.1007/s40273-016-0480-2.
734. Martinez V, Beloeil H, Marret E, Fletcher D, Ravaud P, Trinquart L. Non-opioid analgesics in adults after major surgery: systematic review with network meta-analysis of randomized trials Br J Anaesth. 2017 Jan;118(1):22-31. doi: 10.1093/bja/aew391.
735. Papadimitropoulou K, Vossen C, Karabis A, Donatti C, Kubitz N. Comparative efficacy and tolerability of pharmacological and somatic interventions in adult patients with treatment-resistant depression: a systematic review and network meta-analysis Curr Med Res Opin. 2017 Apr;33(4):701-711. doi: 10.1080/03007995.2016.1277201. Epub 2017 Feb 6.
736. Zhang J, Wang B, Li R, Ge L, Chen KH, Tian J. Does antimicrobial lock solution reduce catheter-related infections in hemodialysis patients with central venous catheters? A Bayesian network meta-analysis Int Urol Nephrol. 2017 Apr;49(4):701-716. doi: 10.1007/s11255-016-1490-x. Epub 2016 Dec 29.
737. Brunoni AR, Chaimani A, Moffa AH, Razza LB, Gattaz WF, Daskalakis ZJ, Carvalho AF. Repetitive Transcranial Magnetic Stimulation for the Acute Treatment of Major Depressive Episodes: A Systematic Review With Network Meta-analysis JAMA Psychiatry. 2017 Feb 1;74(2):143-152. doi: 10.1001/jamapsychiatry.2016.3644.
738. Abdel-Qadir H, Ong G, Fazelzad R, Amir E, Lee DS, Thavendiranathan P, Tomlinson G. Interventions for preventing cardiomyopathy due to anthracyclines: a Bayesian network meta-analysis Ann Oncol. 2017 Mar 1;28(3):628-633. doi: 10.1093/annonc/mdw671.
739. Huang Y, Wang H, Luo G, Zhang Y, Wang L, Li K. A systematic review and network meta-analysis of neoadjuvant therapy combined with surgery for patients with resectable esophageal squamous cell carcinoma Int J Surg. 2017 Feb;38:41-47. doi: 10.1016/j.ijsu.2016.12.035. Epub 2016 Dec 24.
740. Tarp S, Eric Furst D, Boers M, Luta G, Bliddal H, Tarp U, Heller Asmussen K, Brock B, Dossing A, Schjødt Jørgensen T, Thirstrup S, Christensen R. Risk of serious adverse effects of biological and targeted drugs in patients with rheumatoid arthritis: a systematic review meta-analysis Rheumatology (Oxford). 2017 Mar 1;56(3):417-425. doi: 10.1093/rheumatology/kew442.
741. Ding PN, Lord SJ, Gebski V, Links M, Bray V, Gralla RJ, Yang JC, Lee CK. Risk of Treatment-Related Toxicities from EGFR Tyrosine Kinase Inhibitors: A Meta-analysis of Clinical Trials of Gefitinib, Erlotinib, and Afatinib in Advanced EGFR-Mutated Non-Small Cell Lung Cancer J Thorac Oncol. 2017 Apr;12(4):633-643. doi: 10.1016/j.jtho.2016.11.2236. Epub 2016 Dec 19.
742. Amer MA, Smith MD, Herbison GP, Plank LD, McCall JL. Network meta-analysis of the effect of preoperative carbohydrate loading on recovery after elective surgery Br J Surg. 2017 Feb;104(3):187-197. doi: 10.1002/bjs.10408. Epub 2016 Dec 21.
743. Lorenzi M, Ploug UJ, Langer J, Skovgaard R, Zoratti M, Jansen J. Liraglutide Versus SGLT-2 Inhibitors in People with Type 2 Diabetes: A Network Meta-Analysis Diabetes Ther. 2017 Feb;8(1):85-99. doi: 10.1007/s13300-016-0217-4. Epub 2016 Dec 19.
744. Guo L, Li S, Wang P, Zhong X, Hong Y. Comparative Efficacy of Clinical Events Prevention of Five Anticoagulants in Patients With Atrial Fibrillation (A Network Meta-Analysis) Am J Cardiol. 2017 Feb 15;119(4):585-593. doi: 10.1016/j.amjcard.2016.11.006. Epub 2016 Nov 16.
745. Schwendicke F, Göstemeyer G. Cost-effectiveness of root caries preventive treatments J Dent. 2017 Jan;56:58-64. doi: 10.1016/j.jdent.2016.10.016. Epub 2016 Oct 29.
746. Htike ZZ, Zaccardi F, Papamargaritis D, Webb DR, Khunti K, Davies MJ. Efficacy and safety of glucagon-like peptide-1 receptor agonists in type 2 diabetes: A systematic review and mixed-treatment comparison analysis Diabetes Obes Metab. 2017 Apr;19(4):524-536. doi: 10.1111/dom.12849. Epub 2017 Feb 17.
747. Rajendra A, Spivakovsky S. Antibiotics in aggressive periodontitis, is there a clinical benefit? Evid Based Dent. 2016 Dec;17(4):100. doi: 10.1038/sj.ebd.6401197.
748. Svedsater H, Stynes G, Wex J, Frith L, Leather D, Castelnuovo E, Detry M, Berry S. Once-daily fluticasone furoate/vilanterol versus twice daily combination therapies in asthma-mixed treatment comparisons of clinical efficacy Asthma Res Pract. 2016 Feb 8;2:4. doi: 10.1186/s40733-015-0016-0. eCollection 2016.
749. Komaki Y, Komaki F, Micic D, Yamada A, Suzuki Y, Sakuraba A. Pharmacologic therapies for severe steroid refractory hospitalized ulcerative colitis: A network meta-analysis J Gastroenterol Hepatol. 2017 Jun;32(6):1143-1151. doi: 10.1111/jgh.13674.
750. Xu H, Han W, Wang J, Li M. Network meta-analysis of migraine disorder treatment by NSAIDs and triptans J Headache Pain. 2016 Dec;17(1):113. doi: 10.1186/s10194-016-0703-0. Epub 2016 Dec 12.
751. Niu M, Hong D, Ma TC, Chen XW, Han JH, Sun J, Xu K. Short-term and long-term efficacy of 7 targeted therapies for the treatment of advanced hepatocellular carcinoma: a network meta-analysis: Efficacy of 7 targeted therapies for AHCC Medicine (Baltimore). 2016 Dec;95(49):e5591. doi: 10.1097/MD.0000000000005591.
752. Dulai PS, Singh S, Marquez E, Khera R, Prokop LJ, Limburg PJ, Gupta S, Murad MH. Chemoprevention of colorectal cancer in individuals with previous colorectal neoplasia: systematic review and network meta-analysis BMJ. 2016 Dec 5;355:i6188. doi: 10.1136/bmj.i6188.
753. Yang XC, Deng ZH, Wen T, Luo W, Xiao WF, Zhao RB, Li YS. Network Meta-Analysis of Pharmacological Agents for Osteoporosis Treatment and Fracture Prevention Cell Physiol Biochem. 2016;40(3-4):781-795. doi: 10.1159/000453138. Epub 2016 Dec 5.
754. Kanters S, Park JJ, Chan K, Ford N, Forrest J, Thorlund K, Nachega JB, Mills EJ. Use of peers to improve adherence to antiretroviral therapy: a global network meta-analysis J Int AIDS Soc. 2016 Nov 30;19(1):21141. doi: 10.7448/IAS.19.1.21141. eCollection 2016.
755. Rodriguez-Gutierrez R, Montori VM. Existing evidence is insufficient to justify metformin or other agents as first-line therapy for type 2 diabetes Evid Based Med. 2016 Dec;21(6):224. doi: 10.1136/ebmed-2016-110562. Epub 2016 Oct 18.
756. Chen JJ, Zhao LB, Liu YY, Fan SH, Xie P. Comparative efficacy and acceptability of electroconvulsive therapy versus repetitive transcranial magnetic stimulation for major depression: A systematic review and multiple-treatments meta-analysis Behav Brain Res. 2017 Mar 1;320:30-36. doi: 10.1016/j.bbr.2016.11.028. Epub 2016 Nov 19.
757. Antonopoulos CN, Mylonas SN, Moulakakis KG, Sergentanis TN, Sfyroeras GS, Lazaris AM, Kakisis JD, Vasdekis SN. A network meta-analysis of randomized controlled trials comparing treatment modalities for de novo superficial femoral artery occlusive lesions J Vasc Surg. 2017 Jan;65(1):234-245.e11. doi: 10.1016/j.jvs.2016.08.095. Epub 2016 Nov 16.
758. Kanters S, Park JJ, Chan K, Socias ME, Ford N, Forrest JI, Thorlund K, Nachega JB, Mills EJ. Interventions to improve adherence to antiretroviral therapy: a systematic review and network meta-analysis Lancet HIV. 2017 Jan;4(1):e31-e40. doi: 10.1016/S2352-3018(16)30206-5. Epub 2016 Nov 16.
759. Li D, Wang T, Shen S, Fang Z, Dong Y, Tang H. Urinary tract and genital infections in patients with type 2 diabetes treated with sodium-glucose co-transporter 2 inhibitors: A meta-analysis of randomized controlled trials Diabetes Obes Metab. 2017 Mar;19(3):348-355. doi: 10.1111/dom.12825. Epub 2016 Dec 19.
760. Mao EJ, Hazlewood GS, Kaplan GG, Peyrin-Biroulet L, Ananthakrishnan AN. Systematic review with meta-analysis: comparative efficacy of immunosuppressants and biologics for reducing hospitalisation and surgery in Crohn's disease and ulcerative colitis Aliment Pharmacol Ther. 2017 Jan;45(1):3-13. doi: 10.1111/apt.13847. Epub 2016 Nov 10.
761. Cabon Y, Molinari N, Marin G, Vachier I, Gamez AS, Chanez P, Bourdin A. Comparison of anti-interleukin-5 therapies in patients with severe asthma: global and indirect meta-analyses of randomized placebo-controlled trials Clin Exp Allergy. 2017 Jan;47(1):129-138. doi: 10.1111/cea.12853. Epub 2016 Dec 8.
762. Emilsson L, Holme Ø, Bretthauer M, Cook NR, Buring JE, Løberg M, Adami HO, Sesso HD, Gaziano MJ, Kalager M. Systematic review with meta-analysis: the comparative effectiveness of aspirin vs. screening for colorectal cancer prevention Aliment Pharmacol Ther. 2017 Jan;45(2):193-204. doi: 10.1111/apt.13857. Epub 2016 Nov 17.
763. Singh JA, Hossain A, Tanjong Ghogomu E, Mudano AS, Tugwell P, Wells GA. Biologic or tofacitinib monotherapy for rheumatoid arthritis in people with traditional disease-modifying anti-rheumatic drug (DMARD) failure: a Cochrane Systematic Review and network meta-analysis (NMA) Cochrane Database Syst Rev. 2016 Nov 17;11(11):CD012437. doi: 10.1002/14651858.CD012437.
764. Komócsi A, Kehl D, d'Ascenso F, DiNicolantonio J, Vorobcsuk A. Pathfinding to an optimal strategy of revascularization in primary coronary intervention in patients with multivessel disease: a network meta-analysis of randomized trials Curr Med Res Opin. 2017 Mar;33(3):421-429. doi: 10.1080/03007995.2016.1260534. Epub 2016 Dec 16.
765. CiRen B, Wang X, Long Z. The evaluation of immunotherapy and chemotherapy treatment on melanoma: a network meta-analysis Oncotarget. 2016 Dec 6;7(49):81493-81511. doi: 10.18632/oncotarget.13277.
766. Lipinski MJ, Lee RC, Gaglia MA Jr, Torguson R, Garcia-Garcia HM, Pichard AD, Satler LF, Waksman R. Comparison of heparin, bivalirudin, and different glycoprotein IIb/IIIa inhibitor regimens for anticoagulation during percutaneous coronary intervention: A network meta-analysis Cardiovasc Revasc Med. 2016 Dec;17(8):535-545. doi: 10.1016/j.carrev.2016.09.011. Epub 2016 Sep 30.
767. Jiang XP, Rui XH, Guo CX, Huang YQ, Li Q, Xu Y. A network meta-analysis of eight chemotherapy regimens for treatment of advanced ovarian cancer Oncotarget. 2017 Mar 21;8(12):19125-19136. doi: 10.18632/oncotarget.13253.
768. Zhao J, Zhang H, Wei L, Xie S, Suo Z. Comparing the long-term efficacy of standard and combined minimally invasive procedures for unresectable HCC: a mixed treatment comparison Oncotarget. 2017 Feb 28;8(9):15101-15113. doi: 10.18632/oncotarget.13145.
769. Rihn JA, Radcliff K, Norvell DC, Eastlack R, Phillips FM, Berland D, Sherry N, Freedman M, Vaccaro AR. Comparative Effectiveness of Treatments for Chronic Low Back Pain: A Multiple Treatment Comparison Analysis Clin Spine Surg. 2017 Jun;30(5):204-225. doi: 10.1097/BSD.0000000000000410.
770. Wang W, Huang P, Gao W, Cao F, Yi M, Chen L, Guo X. Efficacy and Acceptability of Different Auxiliary Drugs in Pediatric Sevoflurane Anesthesia: A Network Meta-analysis of Mixed Treatment Comparisons Sci Rep. 2016 Nov 10;6:36553. doi: 10.1038/srep36553.
771. Gunst J, Doig GS. The optimal blood glucose target in critically ill patients: more questions than answers Intensive Care Med. 2017 Jan;43(1):110-112. doi: 10.1007/s00134-016-4610-2. Epub 2016 Nov 8.
772. Rousseau B, Kempf E, Desamericq G, Boissier E, Chaubet-Houdu M, Joly C, Saldana C, Boussion H, Neuzillet C, Macquin-Mavier I, Oudard S, Salomon L, de la Taille A, Tournigand C. First-line antiangiogenics for metastatic renal cell carcinoma: A systematic review and network meta-analysis Crit Rev Oncol Hematol. 2016 Nov;107:44-53. doi: 10.1016/j.critrevonc.2016.08.012. Epub 2016 Sep 1.
773. Wang X, Deng Q, Liu B, Yu X. Preventing Emergence Agitation Using Ancillary Drugs with Sevoflurane for Pediatric Anesthesia: A Network Meta-Analysis Mol Neurobiol. 2017 Nov;54(9):7312-7326. doi: 10.1007/s12035-016-0229-0. Epub 2016 Nov 4.
774. Ba-Sang DZ, Long ZW, Teng H, Zhao XP, Qiu J, Li MS. A network meta-analysis on the efficacy of sixteen targeted drugs in combination with chemotherapy for treatment of advanced/metastatic colorectal cancer Oncotarget. 2016 Dec 20;7(51):84468-84479. doi: 10.18632/oncotarget.12994.
775. Tsikopoulos K, Vasiliadis HS, Mavridis D. Injection therapies for plantar fasciopathy ('plantar fasciitis'): a systematic review and network meta-analysis of 22 randomised controlled trials Br J Sports Med. 2016 Nov;50(22):1367-1375. doi: 10.1136/bjsports-2015-095437. Epub 2016 May 3.
776. Moggia E, Rouse B, Simillis C, Li T, Vaughan J, Davidson BR, Gurusamy KS. Methods to decrease blood loss during liver resection: a network meta-analysis Cochrane Database Syst Rev. 2016 Oct 31;10(10):CD010683. doi: 10.1002/14651858.CD010683.pub3.
777. Wang L, Yu QY, Liu Y, Zhu ZL, Huang YW, Li K. Efficacy and Safety of Laser Surgery and Transurethral Resection of the Prostate for Treating Benign Prostate Hyperplasia: a Network Meta-analysis Asian Pac J Cancer Prev. 2016;17(9):4281-4288.
778. Zhang Y, Yang Y, Zhang Z, Fang W, Kang S, Luo Y, Sheng J, Zhan J, Hong S, Huang Y, Zhou N, Zhao H, Zhang L. Neurokinin-1 Receptor Antagonist-Based Triple Regimens in Preventing Chemotherapy-Induced Nausea and Vomiting: A Network Meta-Analysis J Natl Cancer Inst. 2016 Oct 30;109(2):djw217. doi: 10.1093/jnci/djw217. Print 2017 Feb.
779. Kanters S, Vitoria M, Doherty M, Socias ME, Ford N, Forrest JI, Popoff E, Bansback N, Nsanzimana S, Thorlund K, Mills EJ. Comparative efficacy and safety of first-line antiretroviral therapy for the treatment of HIV infection: a systematic review and network meta-analysis Lancet HIV. 2016 Nov;3(11):e510-e520. doi: 10.1016/S2352-3018(16)30091-1. Epub 2016 Sep 6.
780. Zhuo C, Li X, Zhuang H, Tian S, Cui H, Jiang R, Liu C, Tao R, Lin X. Evaluating the efficacy and safety of intravesical chemotherapies for non-muscle invasive bladder cancer: a network meta-analysis Oncotarget. 2016 Dec 13;7(50):82567-82579. doi: 10.18632/oncotarget.12856.
781. Devji T, Levine O, Neupane B, Beyene J, Xie F. Systemic Therapy for Previously Untreated Advanced BRAF-Mutated Melanoma: A Systematic Review and Network Meta-Analysis of Randomized Clinical Trials JAMA Oncol. 2017 Mar 1;3(3):366-373. doi: 10.1001/jamaoncol.2016.4877.
782. Wang Q, Huang H, Zeng X, Ma Y, Zhao X, Huang M. Single-agent maintenance therapy for advanced non-small cell lung cancer (NSCLC): a systematic review and Bayesian network meta-analysis of 26 randomized controlled trials PeerJ. 2016 Oct 20;4:e2550. doi: 10.7717/peerj.2550. eCollection 2016.
783. Campos MS, Ayres LR, Morelo MR, Marques FA, Pereira LR. Efficacy and Tolerability of Antiepileptic Drugs in Patients with Focal Epilepsy: Systematic Review and Network Meta-analyses Pharmacotherapy. 2016 Dec;36(12):1255-1271. doi: 10.1002/phar.1855.
784. Vickers AD, Ainsworth C, Mody R, Bergman A, Ling CS, Medjedovic J, Smyth M. Systematic Review with Network Meta-Analysis: Comparative Efficacy of Biologics in the Treatment of Moderately to Severely Active Ulcerative Colitis PLoS One. 2016 Oct 24;11(10):e0165435. doi: 10.1371/journal.pone.0165435. eCollection 2016.
785. Rodrigues IA, Lim KS. Re: Li et al.: Comparative effectiveness of first-line medications for primary open-angle glaucoma: a systematic review and network meta-analysis (Ophthalmology 2016;123:129-40) Ophthalmology. 2016 Nov;123(11):e65-e66. doi: 10.1016/j.ophtha.2016.03.042.
786. Palmer SC, Gardner S, Tonelli M, Mavridis D, Johnson DW, Craig JC, French R, Ruospo M, Strippoli GF. Phosphate-Binding Agents in Adults With CKD: A Network Meta-analysis of Randomized Trials Am J Kidney Dis. 2016 Nov;68(5):691-702. doi: 10.1053/j.ajkd.2016.05.015. Epub 2016 Jul 22.
787. Tarp S, Furst DE, Dossing A, Østergaard M, Lorenzen T, Hansen MS, Singh JA, Choy EH, Boers M, Suarez-Almazor ME, Kristensen LE, Bliddal H, Christensen R. Defining the optimal biological monotherapy in rheumatoid arthritis: A systematic review and meta-analysis of randomised trials Semin Arthritis Rheum. 2017 Jun;46(6):699-708. doi: 10.1016/j.semarthrit.2016.09.003. Epub 2016 Sep 14.
788. Yang Y, Pei J, Gao G, Yang Z, Guo S, Yue B, Qiu J. Pharmacological interventions for melanoma: Comparative analysis using bayesian meta-analysis Oncotarget. 2016 Dec 6;7(49):80855-80871. doi: 10.18632/oncotarget.12644.
789. Lu Y, Cheng Z, Zhao Y, Chang X, Chan C, Bai Y, Cheng N. Efficacy and safety of long-term treatment with statins for coronary heart disease: A Bayesian network meta-analysis Atherosclerosis. 2016 Nov;254:215-227. doi: 10.1016/j.atherosclerosis.2016.10.025. Epub 2016 Oct 14.
790. Currie AC, Malietzis G, Jenkins JT, Yamada T, Ashrafian H, Athanasiou T, Okabayashi K, Kennedy RH. Network meta-analysis of protocol-driven care and laparoscopic surgery for colorectal cancer Br J Surg. 2016 Dec;103(13):1783-1794. doi: 10.1002/bjs.10306. Epub 2016 Oct 20.
791. Qin Z, Wu J, Tian J, Zhou J, Liu Y, Liu Z. Network Meta-Analysis of the Efficacy of Acupuncture, Alpha-blockers and Antibiotics on Chronic Prostatitis/Chronic Pelvic Pain Syndrome Sci Rep. 2016 Oct 19;6:35737. doi: 10.1038/srep35737.
792. Borba HH, Wiens A, Steimbach LM, Perlin CM, Tonin FS, Pedroso ML, Fernandez-Llimos F, Pontarolo R. Network meta-analysis of first- and second-generation protease inhibitors for chronic hepatitis C genotype 1: efficacy based on RVR and SVR 24 Eur J Clin Pharmacol. 2017 Jan;73(1):1-14. doi: 10.1007/s00228-016-2146-6. Epub 2016 Oct 19.
793. Nee J, Feuerstein JD. Review: Prucalopride, velusetrag, bisacodyl, and sodium picosulfate improve chronic idiopathic constipation Ann Intern Med. 2016 Oct 18;165(8):JC41. doi: 10.7326/ACPJC-2016-165-8-041.
794. Betts KA, Griffith J, Song Y, Mittal M, Joshi A, Wu EQ, Ganguli A. Network Meta-Analysis and Cost Per Responder of Tumor Necrosis Factor-α and Interleukin Inhibitors in the Treatment of Active Ankylosing Spondylitis Rheumatol Ther. 2016 Dec;3(2):323-336. doi: 10.1007/s40744-016-0038-y. Epub 2016 Jul 25.
795. Zhou J, Wang T, Zhao X, Miller DR, Zhai S. Comparative Efficacy of Bisphosphonates to Prevent Fracture in Men with Osteoporosis: A Systematic Review with Network Meta-Analyses Rheumatol Ther. 2016 Jun;3(1):117-128. doi: 10.1007/s40744-016-0030-6. Epub 2016 Apr 22.
796. Li Y, Gao J, He S, Zhang Y, Wang Q. An Evaluation on the Efficacy and Safety of Treatments for Attention Deficit Hyperactivity Disorder in Children and Adolescents: a Comparison of Multiple Treatments Mol Neurobiol. 2017 Nov;54(9):6655-6669. doi: 10.1007/s12035-016-0179-6. Epub 2016 Oct 13.
797. Zhao BC, Huang TY, Deng QW, Liu WF, Liu J, Deng WT, Liu KX, Li C. Prophylaxis Against Atrial Fibrillation After General Thoracic Surgery: Trial Sequential Analysis and Network Meta-Analysis Chest. 2017 Jan;151(1):149-159. doi: 10.1016/j.chest.2016.08.1476. Epub 2016 Oct 8.
798. Alfonso-Cristancho R, Armstrong N, Arjunji R, Riemsma R, Worthy G, Ganguly R, Kleijnen J. Comparative effectiveness of biologics for the management of rheumatoid arthritis: systematic review and network meta-analysis Clin Rheumatol. 2017 Jan;36(1):25-34. doi: 10.1007/s10067-016-3435-2. Epub 2016 Oct 10.
799. Guo M, Cao Y, Yang J, Zhang J. A comparison of drug resistances of targeted drugs for advanced renal cell cancer approved by the Food and Drug Administration: A meta-analysis of randomized clinical trials J Cancer Res Ther. 2016 Oct;12(Supplement):109-115. doi: 10.4103/0973-1482.191617.
800. Huang J, Sun M, Wang Z, Zhang Q, Lou J, Cai Y, Chen W, Du X. Induction treatments for acute promyelocytic leukemia: a network meta-analysis Oncotarget. 2016 Nov 1;7(44):71974-71986. doi: 10.18632/oncotarget.12451.
801. Brigo F, Bragazzi NL, Nardone R, Trinka E. Efficacy and tolerability of brivaracetam compared to lacosamide, eslicarbazepine acetate, and perampanel as adjunctive treatments in uncontrolled focal epilepsy: Results of an indirect comparison meta-analysis of RCTs Seizure. 2016 Nov;42:29-37. doi: 10.1016/j.seizure.2016.08.007. Epub 2016 Sep 24.
802. Park J, Lee JM, Lee JS, Cho YJ. Pharmacological and Mechanical Thromboprophylaxis in Critically Ill Patients: a Network Meta-Analysis of 12 Trials J Korean Med Sci. 2016 Nov;31(11):1828-1837. doi: 10.3346/jkms.2016.31.11.1828.
803. Katsanos K, Kitrou P, Spiliopoulos S, Diamantopoulos A, Karnabatidis D. Comparative Effectiveness of Plain Balloon Angioplasty, Bare Metal Stents, Drug-Coated Balloons, and Drug-Eluting Stents for the Treatment of Infrapopliteal Artery Disease: Systematic Review and Bayesian Network Meta-analysis of Randomized Controlled Trials J Endovasc Ther. 2016 Dec;23(6):851-863. doi: 10.1177/1526602816671740. Epub 2016 Oct 5.
804. Su X, Xie X, Liu L, Lv J, Song F, Perkovic V, Zhang H. Comparative Effectiveness of 12 Treatment Strategies for Preventing Contrast-Induced Acute Kidney Injury: A Systematic Review and Bayesian Network Meta-analysis Am J Kidney Dis. 2017 Jan;69(1):69-77. doi: 10.1053/j.ajkd.2016.07.033. Epub 2016 Oct 1.
805. Tao R, Li X, Ran R, Xiao Z, Zhang H, Kong H, Song Q, Huang Y, Wang L, Huang J. A mixed analysis comparing nine minimally invasive surgeries for unresectable hepatocellular carcinoma patients Oncotarget. 2017 Jan 17;8(3):5460-5473. doi: 10.18632/oncotarget.12348.
806. Zhao YJ, Lin L, Teng M, Khoo AL, Soh LB, Furukawa TA, Baldessarini RJ, Lim BP, Sim K. Long-term antipsychotic treatment in schizophrenia: systematic review and network meta-analysis of randomised controlled trials BJPsych Open. 2016 Feb 5;2(1):59-66. doi: 10.1192/bjpo.bp.115.002576. eCollection 2016 Jan.
807. Elborn JS, Vataire AL, Fukushima A, Aballea S, Khemiri A, Moore C, Medic G, Hemels ME. Comparison of Inhaled Antibiotics for the Treatment of Chronic Pseudomonas aeruginosa Lung Infection in Patients With Cystic Fibrosis: Systematic Literature Review and Network Meta-analysis Clin Ther. 2016 Oct;38(10):2204-2226. doi: 10.1016/j.clinthera.2016.08.014. Epub 2016 Sep 29.
808. Moćko P, Kawalec P, Pilc A. Safety profile of biologic drugs in the therapy of Crohn disease: A systematic review and network meta-analysis Pharmacol Rep. 2016 Dec;68(6):1237-1243. doi: 10.1016/j.pharep.2016.07.013. Epub 2016 Aug 1.
809. Yeo YH, Shiu SI, Ho HJ, Zou B, Lin JT, Wu MS, Liou JM, Wu CY; Taiwan Gastrointestinal Disease and Helicobacter Consortium. First-line Helicobacter pylori eradication therapies in countries with high and low clarithromycin resistance: a systematic review and network meta-analysis Gut. 2018 Jan;67(1):20-27. doi: 10.1136/gutjnl-2016-311868. Epub 2016 Sep 26.
810. Liu W, Wu YH, Zhang L, Liu XY, Bin Xue, Bin Liu, Wang Y, Ji Y. Efficacy and safety of TNF-α inhibitors for active ankylosing spondylitis patients: Multiple treatment comparisons in a network meta-analysis Sci Rep. 2016 Sep 26;6:32768. doi: 10.1038/srep32768.
811. Tang H, Fang Z, Wang T, Cui W, Zhai S, Song Y. Meta-Analysis of Effects of Sodium-Glucose Cotransporter 2 Inhibitors on Cardiovascular Outcomes and All-Cause Mortality Among Patients With Type 2 Diabetes Mellitus Am J Cardiol. 2016 Dec 1;118(11):1774-1780. doi: 10.1016/j.amjcard.2016.08.061. Epub 2016 Aug 31.
812. Rowe BH, Kirkland SW, Vandermeer B, Campbell S, Newton A, Ducharme FM, Villa-Roel C. Prioritizing Systemic Corticosteroid Treatments to Mitigate Relapse in Adults With Acute Asthma: A Systematic Review and Network Meta-analysis Acad Emerg Med. 2017 Mar;24(3):371-381. doi: 10.1111/acem.13107.
813. Belletti A, Benedetto U, Biondi-Zoccai G, Leggieri C, Silvani P, Angelini GD, Zangrillo A, Landoni G. The effect of vasoactive drugs on mortality in patients with severe sepsis and septic shock. A network meta-analysis of randomized trials J Crit Care. 2017 Feb;37:91-98. doi: 10.1016/j.jcrc.2016.08.010. Epub 2016 Aug 13.
814. Fogarty E, Schmitz S, Tubridy N, Walsh C, Barry M. Comparative efficacy of disease-modifying therapies for patients with relapsing remitting multiple sclerosis: Systematic review and network meta-analysis Mult Scler Relat Disord. 2016 Sep;9:23-30. doi: 10.1016/j.msard.2016.06.001. Epub 2016 Jun 8.
815. Wang Y, Wang H, Jiang J, Zhao D, Liu Y. Comparative Efficacy and Acceptability of Anti-TNF-Alpha Therapy in Ankylosing Spondylitis: A Mixed-Treatments Comparison Cell Physiol Biochem. 2016;39(5):1679-1694. doi: 10.1159/000447869. Epub 2016 Sep 19.
816. Lee YH, Song GG. Comparative efficacy and safety of tacrolimus, mycophenolate mofetil, azathioprine, and cyclophosphamide as maintenance therapy for lupus nephritis : A Bayesian network meta-analysis of randomized controlled trials Z Rheumatol. 2017 Dec;76(10):904-912. doi: 10.1007/s00393-016-0186-z.
817. Singh JA, Hossain A, Kotb A, Wells G. Risk of serious infections with immunosuppressive drugs and glucocorticoids for lupus nephritis: a systematic review and network meta-analysis BMC Med. 2016 Sep 13;14(1):137. doi: 10.1186/s12916-016-0673-8.
818. Mazurek M, Lip GY. To occlude or not? Left atrial appendage occlusion for stroke prevention in atrial fibrillation Heart. 2017 Jan 15;103(2):93-95. doi: 10.1136/heartjnl-2016-310255. Epub 2016 Sep 12.
819. Windle SB, Filion KB, Mancini JG, Adye-White L, Joseph L, Gore GC, Habib B, Grad R, Pilote L, Eisenberg MJ. Combination Therapies for Smoking Cessation: A Hierarchical Bayesian Meta-Analysis Am J Prev Med. 2016 Dec;51(6):1060-1071. doi: 10.1016/j.amepre.2016.07.011. Epub 2016 Sep 9.
820. Jain S, Khera R, Girotra S, Badesch D, Wang Z, Murad MH, Blevins A, Schmidt GA, Singh S, Gerke AK. Comparative Effectiveness of Pharmacologic Interventions for Pulmonary Arterial Hypertension: A Systematic Review and Network Meta-Analysis Chest. 2017 Jan;151(1):90-105. doi: 10.1016/j.chest.2016.08.1461. Epub 2016 Sep 9.
821. Zhang W, Deng X, Liu C, Wang X. Intravesical treatment for interstitial cystitis/painful bladder syndrome: a network meta-analysis Int Urogynecol J. 2017 Apr;28(4):515-525. doi: 10.1007/s00192-016-3079-4. Epub 2016 Sep 10.
822. Smith PH, Weinberger AH, Zhang J, Emme E, Mazure CM, McKee SA. Sex Differences in Smoking Cessation Pharmacotherapy Comparative Efficacy: A Network Meta-analysis Nicotine Tob Res. 2017 Mar 1;19(3):273-281. doi: 10.1093/ntr/ntw144.
823. O'Connor AM, Yuan C, Cullen JN, Coetzee JF, da Silva N, Wang C. A mixed treatment meta-analysis of antibiotic treatment options for bovine respiratory disease - An update Prev Vet Med. 2016 Sep 15;132:130-139. doi: 10.1016/j.prevetmed.2016.07.003. Epub 2016 Aug 31.
824. Qiu S, Tang Z, Deng L, Liu L, Han P, Yang L, Wei Q. Comparisons of regular and on-demand regimen of PED5-Is in the treatment of ED after nerve-sparing radical prostatectomy for Prostate Cancer Sci Rep. 2016 Sep 9;6:32853. doi: 10.1038/srep32853.
825. Li S, Yang H, Guo Y, Wei F, Yang X, Li D, Li M, Xu W, Li W, Sun L, Gao Y, Wang Y. Comparative efficacy and safety of urate-lowering therapy for the treatment of hyperuricemia: a systematic review and network meta-analysis Sci Rep. 2016 Sep 8;6:33082. doi: 10.1038/srep33082.
826. Riboh JC, Cvetanovich GL, Cole BJ, Yanke AB. Comparative efficacy of cartilage repair procedures in the knee: a network meta-analysis Knee Surg Sports Traumatol Arthrosc. 2017 Dec;25(12):3786-3799. doi: 10.1007/s00167-016-4300-1. Epub 2016 Sep 7.
827. Moćko P, Kawalec P, Pilc A. Safety Profile of Biologic Drugs in the Treatment of Inflammatory Bowel Diseases: A Systematic Review and Network Meta-analysis of Randomized Controlled Trials Clin Drug Investig. 2017 Jan;37(1):25-37. doi: 10.1007/s40261-016-0459-y.
828. Alfirevic Z, Keeney E, Dowswell T, Welton NJ, Medley N, Dias S, Jones LV, Gyte G, Caldwell DM. Which method is best for the induction of labour? A systematic review, network meta-analysis and cost-effectiveness analysis Health Technol Assess. 2016 Aug;20(65):1-584. doi: 10.3310/hta20650.
829. Abdel-Rahman O. Neurokinin-1 inhibitors in the prevention of nausea and vomiting from highly emetogenic chemotherapy: a network meta-analysis Ther Adv Med Oncol. 2016 Sep;8(5):396-406. doi: 10.1177/1758834016654902. Epub 2016 Jun 29.
830. Chen L, Chen Y, Li B. The efficacy and safety of proton-pump inhibitors in treating patients with non-erosive reflux disease: a network meta-analysis Sci Rep. 2016 Sep 1;6:32126. doi: 10.1038/srep32126.
831. Jones-Hughes T, Snowsill T, Haasova M, Coelho H, Crathorne L, Cooper C, Mujica-Mota R, Peters J, Varley-Campbell J, Huxley N, Moore J, Allwood M, Lowe J, Hyde C, Hoyle M, Bond M, Anderson R. Immunosuppressive therapy for kidney transplantation in adults: a systematic review and economic model Health Technol Assess. 2016 Aug;20(62):1-594. doi: 10.3310/hta20620.
832. Ter Veer E, Haj Mohammad N, van Valkenhoef G, Ngai LL, Mali RMA, Anderegg MC, van Oijen MGH, van Laarhoven HWM. The Efficacy and Safety of First-line Chemotherapy in Advanced Esophagogastric Cancer: A Network Meta-analysis J Natl Cancer Inst. 2016 Aug 30;108(10). doi: 10.1093/jnci/djw166. Print 2016 Oct.
833. Hazlewood GS, Barnabe C, Tomlinson G, Marshall D, Devoe DJ, Bombardier C. Methotrexate monotherapy and methotrexate combination therapy with traditional and biologic disease modifying anti-rheumatic drugs for rheumatoid arthritis: A network meta-analysis Cochrane Database Syst Rev. 2016 Aug 29;2016(8):CD010227. doi: 10.1002/14651858.CD010227.pub2.
834. Tawfik A, Bielecki JM, Krahn M, Dorian P, Hoch JS, Boon H, Husereau D, Pechlivanoglou P. Systematic review and network meta-analysis of stroke prevention treatments in patients with atrial fibrillation Clin Pharmacol. 2016 Aug 11;8:93-107. doi: 10.2147/CPAA.S105165. eCollection 2016.
835. Batson S, Sutton A, Abrams K. Exploratory Network Meta Regression Analysis of Stroke Prevention in Atrial Fibrillation Fails to Identify Any Interactions with Treatment Effect PLoS One. 2016 Aug 25;11(8):e0161864. doi: 10.1371/journal.pone.0161864. eCollection 2016.
836. Xiao Y, Chen Z, Yang Y, Kou L. Network meta-analysis of balloon angioplasty, nondrug metal stent, drug-eluting balloon, and drug-eluting stent for treatment of infrapopliteal artery occlusive disease Diagn Interv Radiol. 2016 Sep-Oct;22(5):436-43. doi: 10.5152/dir.2016.15430.
837. Kleeff J, Stöß C, Mayerle J, Stecher L, Maak M, Simon P, Nitsche U, Friess H. Evidence-Based Surgical Treatments for Chronic Pancreatitis Dtsch Arztebl Int. 2016 Jul 25;113(29-30):489-96. doi: 10.3238/arztebl.2016.0489.
838. Escribano M, Figuero E, Martín C, Tobías A, Serrano J, Roldán S, Herrera D. Efficacy of adjunctive anti-plaque chemical agents: a systematic review and network meta-analyses of the Turesky modification of the Quigley and Hein plaque index J Clin Periodontol. 2016 Dec;43(12):1059-1073. doi: 10.1111/jcpe.12616. Epub 2016 Oct 10.
839. Thorlund K, Toor K, Wu P, Chan K, Druyts E, Ramos E, Bhambri R, Donnet A, Stark R, Goadsby PJ. Comparative tolerability of treatments for acute migraine: A network meta-analysis Cephalalgia. 2017 Sep;37(10):965-978. doi: 10.1177/0333102416660552. Epub 2016 Aug 12.
840. Sawangjit R, Chongmelaxme B, Phisalprapa P, Saokaew S, Thakkinstian A, Kowdley KV, Chaiyakunapruk N. Comparative efficacy of interventions on nonalcoholic fatty liver disease (NAFLD): A PRISMA-compliant systematic review and network meta-analysis Medicine (Baltimore). 2016 Aug;95(32):e4529. doi: 10.1097/MD.0000000000004529.
841. Rodriguez EA, Donath E, Waljee AK, Sussman DA. Value of Oral Proton Pump Inhibitors in Acute, Nonvariceal Upper Gastrointestinal Bleeding: A Network Meta-Analysis J Clin Gastroenterol. 2017 Sep;51(8):707-719. doi: 10.1097/MCG.0000000000000625.
842. Bannuru RR, Osani M, Vaysbrot EE, McAlindon TE. Comparative safety profile of hyaluronic acid products for knee osteoarthritis: a systematic review and network meta-analysis Osteoarthritis Cartilage. 2016 Dec;24(12):2022-2041. doi: 10.1016/j.joca.2016.07.010. Epub 2016 Aug 2.
843. Cullum N, Buckley H, Dumville J, Hall J, Lamb K, Madden M, Morley R, O’Meara S, Goncalves PS, Soares M, Stubbs N. Wounds research for patient benefit: a 5-year programme of research Southampton (UK): NIHR Journals Library; 2016 Aug.
844. Xin Y, Manson J, Govan L, Harbour R, Bennison J, Watson E, Wu O. Pharmacological regimens for eradication of Helicobacter pylori: an overview of systematic reviews and network meta-analysis BMC Gastroenterol. 2016 Jul 26;16(1):80. doi: 10.1186/s12876-016-0491-7.
845. Qi L, Ding L, Wang S, Zhong Y, Zhao D, Gao L, Wang W, Lv P, Xu Y, Wang S. A network meta-analysis: the overall and progression-free survival of glioma patients treated by different chemotherapeutic interventions combined with radiation therapy (RT) Oncotarget. 2016 Oct 18;7(42):69002-69013. doi: 10.18632/oncotarget.10763.
846. Cairo F, Pagliaro U, Buti J, Baccini M, Graziani F, Tonelli P, Pagavino G, Tonetti MS. Root coverage procedures improve patient aesthetics. A systematic review and Bayesian network meta-analysis J Clin Periodontol. 2016 Nov;43(11):965-975. doi: 10.1111/jcpe.12603. Epub 2016 Sep 16.
847. Zhao F, Wang S, Liu L, Wang Y. Comparative effectiveness of histamine-2 receptor antagonists as short-term therapy for gastro-esophageal reflux disease: a network meta-analysis Int J Clin Pharmacol Ther. 2016 Oct;54(10):761-70. doi: 10.5414/CP202564.
848. Ferreira VL, Assis Jarek NA, Tonin FS, Borba HH, Wiens A, Pontarolo R. Safety of interferon-free therapies for chronic hepatitis C: a network meta-analysis J Clin Pharm Ther. 2016 Oct;41(5):478-85. doi: 10.1111/jcpt.12426. Epub 2016 Jul 21.
849. Zhang L, Wang W, Gao Y, Lan J, Xie L. The Efficacy and Safety of Current Treatments in Diabetic Macular Edema: A Systematic Review and Network Meta-Analysis PLoS One. 2016 Jul 19;11(7):e0159553. doi: 10.1371/journal.pone.0159553. eCollection 2016.
850. Andersen SE, Christensen M. Hypoglycaemia when adding sulphonylurea to metformin: a systematic review and network meta-analysis Br J Clin Pharmacol. 2016 Nov;82(5):1291-1302. doi: 10.1111/bcp.13059. Epub 2016 Aug 3.
851. Datta NR, Rogers S, Klingbiel D, Gómez S, Puric E, Bodis S. Hyperthermia and radiotherapy with or without chemotherapy in locally advanced cervical cancer: a systematic review with conventional and network meta-analyses Int J Hyperthermia. 2016 Nov;32(7):809-21. doi: 10.1080/02656736.2016.1195924. Epub 2016 Aug 14.
852. Tang HL, Li DD, Zhang JJ, Hsu YH, Wang TS, Zhai SD, Song YQ. Lack of evidence for a harmful effect of sodium-glucose co-transporter 2 (SGLT2) inhibitors on fracture risk among type 2 diabetes patients: a network and cumulative meta-analysis of randomized controlled trials Diabetes Obes Metab. 2016 Dec;18(12):1199-1206. doi: 10.1111/dom.12742. Epub 2016 Aug 15.
853. Park CH, Jung YS, Nam E, Eun CS, Park DI, Han DS. Comparison of Efficacy of Prophylactic Endoscopic Therapies for Postpolypectomy Bleeding in the Colorectum: A Systematic Review and Network Meta-Analysis Am J Gastroenterol. 2016 Sep;111(9):1230-43. doi: 10.1038/ajg.2016.287. Epub 2016 Jul 12.
854. IsHak WW, Naghdechi L. Are sertraline, paroxetine and duloxetine the most effective antidepressants for use in depressed adults over 60 years? Evid Based Ment Health. 2016 Nov;19(4):e26. doi: 10.1136/eb-2016-102392. Epub 2016 Jul 7.
855. Igarashi A, Inoue S, Ishii T, Tsutani K, Watanabe H. Comparative Effectiveness of Oral Medications for Pulmonary Arterial Hypertension Int Heart J. 2016 Jul 27;57(4):466-72. doi: 10.1536/ihj.15-459. Epub 2016 Jul 7.
856. Patel S, Hee SW, Mistry D, Jordan J, Brown S, Dritsaki M, Ellard DR, Friede T, Lamb SE, Lord J, Madan J, Morris T, Stallard N, Tysall C, Willis A, Underwood M; the Repository Group. Identifying back pain subgroups: developing and applying approaches using individual patient data collected within clinical trials Southampton (UK): NIHR Journals Library; 2016 Jul.
857. Ge L, Wang YF, Tian JH, Mao L, Zhang J, Zhang JH, Shen XP, Yang KH. Network meta-analysis of Chinese herb injections combined with FOLFOX chemotherapy in the treatment of advanced colorectal cancer J Clin Pharm Ther. 2016 Aug;41(4):383-91. doi: 10.1111/jcpt.12410.
858. Wang W, Shi G, Ma B, Hao X, Dong X, Zhang B. Chemotherapy for Adults with Malignant Glioma: A Systematic Review and Network Meta-Analysis Turk Neurosurg. 2017;27(2):174-181. doi: 10.5137/1019-5149.JTN.15462-15.0.
859. García García MA, Rosero Arenas MA, Ruiz Granell R, Chorro Gascó FJ, Martínez Cornejo A. Usefulness of cardiac resynchronisation therapy devices and implantable cardioverter defibrillators in the treatment of heart failure due to severe systolic dysfunction: systematic review of clinical trials and network meta-analysis Heart Asia. 2016 Jan 27;8(1):8-15. doi: 10.1136/heartasia-2015-010634. eCollection 2016.
860. Becker U, Briggs AH, Moreno SG, Ray JA, Ngo P, Samanta K. Cost-Effectiveness Model for Chemoimmunotherapy Options in Patients with Previously Untreated Chronic Lymphocytic Leukemia Unsuitable for Full-Dose Fludarabine-Based Therapy Value Health. 2016 Jun;19(4):374-82. doi: 10.1016/j.jval.2015.12.018. Epub 2016 Mar 4.
861. Kang M, Jeong CW, Kwak C, Kim HH, Ku JH. Single, immediate postoperative instillation of chemotherapy in non-muscle invasive bladder cancer: a systematic review and network meta-analysis of randomized clinical trials using different drugs Oncotarget. 2016 Jul 19;7(29):45479-45488. doi: 10.18632/oncotarget.9991.
862. Wu F, Wu D, Ren Y, Duan C, Chen S, Xu A. Bayesian network meta-analysis comparing five contemporary treatment strategies for newly diagnosed acute promyelocytic leukaemia Oncotarget. 2016 Jul 26;7(30):47319-47331. doi: 10.18632/oncotarget.10118.
863. Lee CM, Woodward M, Colagiuri S. Triple therapy combinations for the treatment of type 2 diabetes - A network meta-analysis Diabetes Res Clin Pract. 2016 Jun;116:149-58. doi: 10.1016/j.diabres.2016.04.037. Epub 2016 Apr 27.
864. Shah R, Rogers KC, Ahmed AJ, King BJ, Rao SV. Effect of Post-Primary Percutaneous Coronary Intervention Bivalirudin Infusion on Acute Stent Thrombosis: Meta-Analysis of Randomized Controlled Trials JACC Cardiovasc Interv. 2016 Jul 11;9(13):1313-20. doi: 10.1016/j.jcin.2016.03.031. Epub 2016 Jun 15.
865. Siontis GC, Piccolo R, Praz F, Valgimigli M, Räber L, Mavridis D, Jüni P, Windecker S. Percutaneous Coronary Interventions for the Treatment of Stenoses in Small Coronary Arteries: A Network Meta-Analysis JACC Cardiovasc Interv. 2016 Jul 11;9(13):1324-34. doi: 10.1016/j.jcin.2016.03.025. Epub 2016 Jun 15.
866. Skapinakis P, Caldwell DM, Hollingworth W, Bryden P, Fineberg NA, Salkovskis P, Welton NJ, Baxter H, Kessler D, Churchill R, Lewis G. Pharmacological and psychotherapeutic interventions for management of obsessive-compulsive disorder in adults: a systematic review and network meta-analysis Lancet Psychiatry. 2016 Aug;3(8):730-739. doi: 10.1016/S2215-0366(16)30069-4. Epub 2016 Jun 16.
867. Moćko P, Kawalec P, Pilc A. Safety Profile of Biologic Drugs in the Therapy of Ulcerative Colitis: A Systematic Review and Network Meta-Analysis Pharmacotherapy. 2016 Aug;36(8):870-9. doi: 10.1002/phar.1785. Epub 2016 Jul 15.
868. Li Z, Chen P, Wang J, Mao Q, Xiang H, Wang X, Wang X, Zhang X. The impact of surgical treatments for lower urinary tract symptoms/benign prostatic hyperplasia on male erectile function: A systematic review and network meta-analysis Medicine (Baltimore). 2016 Jun;95(24):e3862. doi: 10.1097/MD.0000000000003862.
869. He F, Man LB, Li GZ, Liu N. Efficacy of α-blocker in improving ureteral stent-related symptoms: a meta-analysis of both direct and indirect comparison Drug Des Devel Ther. 2016 May 26;10:1783-93. doi: 10.2147/DDDT.S103195. eCollection 2016.
870. Wise J. Weight loss drugs are compared for effectiveness BMJ. 2016 Jun 14;353:i3330. doi: 10.1136/bmj.i3330.
871. Skapinakis P, Caldwell D, Hollingworth W, Bryden P, Fineberg N, Salkovskis P, Welton N, Baxter H, Kessler D, Churchill R, Lewis G. A systematic review of the clinical effectiveness and cost-effectiveness of pharmacological and psychological interventions for the management of obsessive-compulsive disorder in children/adolescents and adults Health Technol Assess. 2016 Jun;20(43):1-392. doi: 10.3310/hta20430.
872. Stagg HR, Harris RJ, Hatherell HA, Obach D, Zhao H, Tsuchiya N, Kranzer K, Nikolayevskyy V, Kim J, Lipman MC, Abubakar I. What are the most efficacious treatment regimens for isoniazid-resistant tuberculosis? A systematic review and network meta-analysis Thorax. 2016 Oct;71(10):940-9. doi: 10.1136/thoraxjnl-2015-208262. Epub 2016 Jun 13.
873. Gómez-García F, Epstein D, Isla-Tejera B, Lorente A, Vélez García-Nieto A, Ruano J. Short-term efficacy and safety of new biological agents targeting the interleukin-23-T helper 17 pathway for moderate-to-severe plaque psoriasis: a systematic review and network meta-analysis Br J Dermatol. 2017 Mar;176(3):594-603. doi: 10.1111/bjd.14814. Epub 2016 Oct 13.
874. Jureidini J. Antidepressants fail, but no cause for therapeutic gloom Lancet. 2016 Aug 27;388(10047):844-5. doi: 10.1016/S0140-6736(16)30585-2. Epub 2016 Jun 8.
875. Thakker D, Nair S, Pagada A, Jamdade V, Malik A. Statin use and the risk of developing diabetes: a network meta-analysis Pharmacoepidemiol Drug Saf. 2016 Oct;25(10):1131-1149. doi: 10.1002/pds.4020. Epub 2016 Jun 9.
876. Sekercioglu N, Thabane L, Díaz Martínez JP, Nesrallah G, Longo CJ, Busse JW, Akhtar-Danesh N, Agarwal A, Al-Khalifah R, Iorio A, Guyatt GH. Comparative Effectiveness of Phosphate Binders in Patients with Chronic Kidney Disease: A Systematic Review and Network Meta-Analysis PLoS One. 2016 Jun 8;11(6):e0156891. doi: 10.1371/journal.pone.0156891. eCollection 2016.
877. Zhou J, Ma X, Wang T, Zhai S. Comparative efficacy of bisphosphonates in short-term fracture prevention for primary osteoporosis: a systematic review with network meta-analyses Osteoporos Int. 2016 Nov;27(11):3289-3300. doi: 10.1007/s00198-016-3654-z. Epub 2016 Jun 7.
878. Kang SH, Chae IH, Park JJ, Lee HS, Kang DY, Hwang SS, Youn TJ, Kim HS. Stent Thrombosis With Drug-Eluting Stents and Bioresorbable Scaffolds: Evidence From a Network Meta-Analysis of 147 Trials JACC Cardiovasc Interv. 2016 Jun 27;9(12):1203-12. doi: 10.1016/j.jcin.2016.03.038. Epub 2016 Jun 1.
879. Sanderson J, Martyn-St James M, Stevens J, Goka E, Wong R, Campbell F, Selby P, Gittoes N, Davis S. Clinical effectiveness of bisphosphonates for the prevention of fragility fractures: A systematic review and network meta-analysis Bone. 2016 Aug;89:52-58. doi: 10.1016/j.bone.2016.05.013. Epub 2016 Jun 1.
880. Choi M, Hyun MK, Choi S, Tchoe HJ, Lee SY, Son KM, Kim MJ, Jung YO, Kim HA. Comparative efficacy of biological agents in methotrexate-refractory rheumatoid arthritis patients: a Bayesian mixed treatment comparison Korean J Intern Med. 2017 May;32(3):536-547. doi: 10.3904/kjim.2015.135. Epub 2016 Jun 2.
881. Archer R, Tappenden P, Ren S, Martyn-St James M, Harvey R, Basarir H, Stevens J, Carroll C, Cantrell A, Lobo A, Hoque S. Infliximab, adalimumab and golimumab for treating moderately to severely active ulcerative colitis after the failure of conventional therapy (including a review of TA140 and TA262): clinical effectiveness systematic review and economic model Health Technol Assess. 2016 May;20(39):1-326. doi: 10.3310/hta20390.
882. Remonti LR, Dias S, Leitão CB, Kramer CK, Klassman LP, Welton NJ, Ades AE, Gross JL. Classes of antihypertensive agents and mortality in hypertensive patients with type 2 diabetes-Network meta-analysis of randomized trials J Diabetes Complications. 2016 Aug;30(6):1192-200. doi: 10.1016/j.jdiacomp.2016.04.020. Epub 2016 Apr 29.
883. Tereshchenko LG, Henrikson CA, Cigarroa J, Steinberg JS. Comparative Effectiveness of Interventions for Stroke Prevention in Atrial Fibrillation: A Network Meta-Analysis J Am Heart Assoc. 2016 May 20;5(5):e003206. doi: 10.1161/JAHA.116.003206.
884. Bonovas S, Fiorino G, Allocca M, Lytras T, Nikolopoulos GK, Peyrin-Biroulet L, Danese S. Biologic Therapies and Risk of Infection and Malignancy in Patients With Inflammatory Bowel Disease: A Systematic Review and Network Meta-analysis Clin Gastroenterol Hepatol. 2016 Oct;14(10):1385-1397.e10. doi: 10.1016/j.cgh.2016.04.039. Epub 2016 May 14.
885. Meister R, von Wolff A, Mohr H, Härter M, Nestoriuc Y, Hölzel L, Kriston L. Comparative Safety of Pharmacologic Treatments for Persistent Depressive Disorder: A Systematic Review and Network Meta-Analysis PLoS One. 2016 May 17;11(5):e0153380. doi: 10.1371/journal.pone.0153380. eCollection 2016.
886. Morrell CJ, Sutcliffe P, Booth A, Stevens J, Scope A, Stevenson M, Harvey R, Bessey A, Cantrell A, Dennis CL, Ren S, Ragonesi M, Barkham M, Churchill D, Henshaw C, Newstead J, Slade P, Spiby H, Stewart-Brown S. A systematic review, evidence synthesis and meta-analysis of quantitative and qualitative studies evaluating the clinical effectiveness, the cost-effectiveness, safety and acceptability of interventions to prevent postnatal depression Health Technol Assess. 2016 May;20(37):1-414. doi: 10.3310/hta20370.
887. Singh JA, Hossain A, Tanjong Ghogomu E, Kotb A, Christensen R, Mudano AS, Maxwell LJ, Shah NP, Tugwell P, Wells GA. Biologics or tofacitinib for rheumatoid arthritis in incomplete responders to methotrexate or other traditional disease-modifying anti-rheumatic drugs: a systematic review and network meta-analysis Cochrane Database Syst Rev. 2016 May 13;2016(5):CD012183. doi: 10.1002/14651858.CD012183.
888. Gupta AK, Daigle D, Foley KA. Network Meta-Analysis of Onychomycosis Treatments Skin Appendage Disord. 2015 Sep;1(2):74-81. doi: 10.1159/000433473. Epub 2015 Jun 26.
889. Cahill K, Lindson-Hawley N, Thomas KH, Fanshawe TR, Lancaster T. Nicotine receptor partial agonists for smoking cessation Cochrane Database Syst Rev. 2016 May 9;2016(5):CD006103. doi: 10.1002/14651858.CD006103.pub7.
890. Li Z, Zhang Y, Quan X, Yang Z, Zeng X, Ji L, Sun F, Zhan S. Efficacy and Acceptability of Glycemic Control of Glucagon-Like Peptide-1 Receptor Agonists among Type 2 Diabetes: A Systematic Review and Network Meta-Analysis PLoS One. 2016 May 9;11(5):e0154206. doi: 10.1371/journal.pone.0154206. eCollection 2016.
891. Fan Y, Cao D, Wei Q, Tang Z, Tan P, Yang L, Liu L, Liu Z, Li X, Xue W. The characteristics of circular disposable devices and in situ devices for optimizing male circumcision: a network meta-analysis Sci Rep. 2016 May 9;6:25514. doi: 10.1038/srep25514.
892. Clive AO, Jones HE, Bhatnagar R, Preston NJ, Maskell N. Interventions for the management of malignant pleural effusions: a network meta-analysis Cochrane Database Syst Rev. 2016 May 8;2016(5):CD010529. doi: 10.1002/14651858.CD010529.pub2.
893. Min SH, Yoon JH, Hahn S, Cho YM. Comparison between SGLT2 inhibitors and DPP4 inhibitors added to insulin therapy in type 2 diabetes: a systematic review with indirect comparison meta-analysis Diabetes Metab Res Rev. 2017 Jan;33(1). doi: 10.1002/dmrr.2818. Epub 2016 Jun 8.
894. Dundar Y, Greenhalgh J, Richardson M, Dwan K. Pharmacological treatment of acute agitation associated with psychotic and bipolar disorder: a systematic review and meta-analysis Hum Psychopharmacol. 2016 Jul;31(4):268-85. doi: 10.1002/hup.2535. Epub 2016 May 5.
895. Stevenson M, Archer R, Tosh J, Simpson E, Everson-Hock E, Stevens J, Hernandez-Alava M, Paisley S, Dickinson K, Scott D, Young A, Wailoo A. Adalimumab, etanercept, infliximab, certolizumab pegol, golimumab, tocilizumab and abatacept for the treatment of rheumatoid arthritis not previously treated with disease-modifying antirheumatic drugs and after the failure of conventional disease-modifying antirheumatic drugs only: systematic review and economic evaluation Health Technol Assess. 2016 Apr;20(35):1-610. doi: 10.3310/hta20350.
896. Abdel-Rahman O, Fouad M. A network meta-analysis of the risk of immune-related renal toxicity in cancer patients treated with immune checkpoint inhibitors Immunotherapy. 2016 May;8(5):665-74. doi: 10.2217/imt-2015-0020.
897. Nagendran M, Maruthappu M, Gordon AC, Gurusamy KS. Comparative safety and efficacy of vasopressors for mortality in septic shock: A network meta-analysis J Intensive Care Soc. 2016 May;17(2):136-145. doi: 10.1177/1751143715620203. Epub 2015 Dec 17.
898. Weisel K, Doyen C, Dimopoulos M, Yee A, Lahuerta JJ, Martin A, Travers K, Druyts E, Toor K, Abildgaard N, Lu J, Van Droogenbroeck J, Geraldes C, Petrini M, Voillat L, Voog E, Facon T. A systematic literature review and network meta-analysis of treatments for patients with untreated multiple myeloma not eligible for stem cell transplantation Leuk Lymphoma. 2017 Jan;58(1):153-161. doi: 10.1080/10428194.2016.1177772. Epub 2016 Apr 28.
899. Yuan JQ, Tsoi KK, Yang M, Wang JY, Threapleton DE, Yang ZY, Zou B, Mao C, Tang JL, Chan FK. Systematic review with network meta-analysis: comparative effectiveness and safety of strategies for preventing NSAID-associated gastrointestinal toxicity Aliment Pharmacol Ther. 2016 Jun;43(12):1262-75. doi: 10.1111/apt.13642. Epub 2016 Apr 28.
900. Miligkos M, Papamichael K, Vande Casteele N, Mantzaris GJ, Gils A, Levesque BG, Zintzaras E. Efficacy and Safety Profile of Anti-tumor Necrosis Factor-α Versus Anti-integrin Agents for the Treatment of Crohn's Disease: A Network Meta-analysis of Indirect Comparisons Clin Ther. 2016 Jun;38(6):1342-1358.e6. doi: 10.1016/j.clinthera.2016.03.018. Epub 2016 Apr 16.
901. Rouse B, Cipriani A, Shi Q, Coleman AL, Dickersin K, Li T. Network Meta-analysis for Clinical Practice Guidelines: A Case Study on First-Line Medical Therapies for Primary Open-Angle Glaucoma Ann Intern Med. 2016 May 17;164(10):674-82. doi: 10.7326/M15-2367. Epub 2016 Apr 19.
902. Khouri C, Jouve T, Blaise S, Carpentier P, Cracowski JL, Roustit M. Peripheral vasoconstriction induced by β-adrenoceptor blockers: a systematic review and a network meta-analysis Br J Clin Pharmacol. 2016 Aug;82(2):549-60. doi: 10.1111/bcp.12980. Epub 2016 May 31.
903. Gurusamy KS, Belgaumkar AP, Haswell A, Pereira SP, Davidson BR. Interventions for necrotising pancreatitis Cochrane Database Syst Rev. 2016 Apr 16;4:CD011383. doi: 10.1002/14651858.CD011383.pub2.
904. Ji T, Feng C, Sun L, Ye X, Bai Y, Chen Q, Qin Y, Zhu J, Zhao X. Are beta-blockers effective for preventing post-coronary artery bypass grafting atrial fibrillation? Direct and network meta-analyses Ir J Med Sci. 2016 May;185(2):503-11. doi: 10.1007/s11845-016-1447-1. Epub 2016 Mar 29.
905. Wang WT, You LK, Chiang CE, Sung SH, Chuang SY, Cheng HM, Chen CH. Comparative Effectiveness of Blood Pressure-lowering Drugs in Patients who have Already Suffered From Stroke: Traditional and Bayesian Network Meta-analysis of Randomized Trials Medicine (Baltimore). 2016 Apr;95(15):e3302. doi: 10.1097/MD.0000000000003302.
906. Lan T, Chang L, Rahmathullah MN, Wu L, Yuan YF. Comparative Efficacy of Interventional Therapies for Early-stage Hepatocellular Carcinoma: A PRISMA-compliant Systematic Review and Network Meta-analysis Medicine (Baltimore). 2016 Apr;95(15):e3185. doi: 10.1097/MD.0000000000003185.
907. Beck-Schimmer B, Shafer SL. How "Volatile" Is the Protection Provided by Inhalational Anesthetics? Anesthesiology. 2016 Jun;124(6):1213-4. doi: 10.1097/ALN.0000000000001121.
908. Zaccardi F, Webb DR, Htike ZZ, Youssef D, Khunti K, Davies MJ. Efficacy and safety of sodium-glucose co-transporter-2 inhibitors in type 2 diabetes mellitus: systematic review and network meta-analysis Diabetes Obes Metab. 2016 Aug;18(8):783-94. doi: 10.1111/dom.12670. Epub 2016 May 13.
909. Thurgar E, Barton S, Karner C, Edwards SJ. Clinical effectiveness and cost-effectiveness of interventions for the treatment of anogenital warts: systematic review and economic evaluation Health Technol Assess. 2016 Mar;20(24):v-vi, 1-486. doi: 10.3310/hta20240.
910. Zhao ZY, Wang HY, Wen B, Yang ZB, Feng K, Fan JC. A Comparison of Midazolam, Lorazepam, and Diazepam for the Treatment of Status Epilepticus in Children: A Network Meta-analysis J Child Neurol. 2016 Aug;31(9):1093-107. doi: 10.1177/0883073816638757. Epub 2016 Mar 28.
911. Bonaparte JP, Ellis D, Quinn JG, Rabski J, Hutton B. A Comparative Assessment of Three Formulations of Botulinum Toxin Type A for Facial Rhytides: A Systematic Review with Meta-Analyses Plast Reconstr Surg. 2016 Apr;137(4):1125-40. doi: 10.1097/PRS.0000000000002004.
912. Shi Q, Li W, Li H, Le Q, Liu S, Zong S, Zheng L, Hou F. Prevention of cisplatin-based chemotherapy-induced delayed nausea and vomiting using triple antiemetic regimens: a mixed treatment comparison Oncotarget. 2016 Apr 26;7(17):24402-14. doi: 10.18632/oncotarget.8255.
913. Papageorgiou SN, Papageorgiou PN, Deschner J, Götz W. Comparative effectiveness of natural and synthetic bone grafts in oral and maxillofacial surgery prior to insertion of dental implants: Systematic review and network meta-analysis of parallel and cluster randomized controlled trials J Dent. 2016 May;48:1-8. doi: 10.1016/j.jdent.2016.03.010. Epub 2016 Mar 21.
914. Zhang X, Shen P, He Q, Yin X, Chen Z, Gui H, Shu K, Tang Q, Yang Y, Pan X, Wang J, Chen N, Zeng H. Different lasers in the treatment of benign prostatic hyperplasia: a network meta-analysis Sci Rep. 2016 Mar 24;6:23503. doi: 10.1038/srep23503.
915. Alfirevic Z, Keeney E, Dowswell T, Welton NJ, Medley N, Dias S, Jones LV, Caldwell DM. Methods to induce labour: a systematic review, network meta-analysis and cost-effectiveness analysis BJOG. 2016 Aug;123(9):1462-70. doi: 10.1111/1471-0528.13981. Epub 2016 Mar 22.
916. Lee YH, Song GG. Comparative efficacy and tolerability of duloxetine, pregabalin, and milnacipran for the treatment of fibromyalgia: a Bayesian network meta-analysis of randomized controlled trials Rheumatol Int. 2016 May;36(5):663-72. doi: 10.1007/s00296-016-3468-5. Epub 2016 Mar 21.
917. Niu PP, Guo ZN, Jin H, Xing YQ, Yang Y. Antiplatelet regimens in the long-term secondary prevention of transient ischaemic attack and ischaemic stroke: an updated network meta-analysis BMJ Open. 2016 Mar 17;6(3):e009013. doi: 10.1136/bmjopen-2015-009013.
918. Chen C, Zhang X, Xiao L, Zhang X, Ma X. Comparative Effectiveness of Biologic Therapy Regimens for Ankylosing Spondylitis: A Systematic Review and a Network Meta-Analysis Medicine (Baltimore). 2016 Mar;95(11):e3060. doi: 10.1097/MD.0000000000003060.
919. Zong SL, Zhao G, Su LX, Liang WD, Li LG, Cheng G, Wang AJ, Cao XQ, Zheng QT, Li LD, Kan SL. Treatments for the Fifth Metacarpal Neck Fractures: A Network Meta-analysis of Randomized Controlled Trials Medicine (Baltimore). 2016 Mar;95(11):e3059. doi: 10.1097/MD.0000000000003059.
920. Ge L, Mao L, Tian JH, Shi FY, Lou Li-li, Qiu X, Li JL, Yang KH. [Network meta-analysis on selecting Chinese medical injections in radiotherapy for esophageal cancer] Zhongguo Zhong Yao Za Zhi. 2015 Sep;40(18):3674-81.
921. Santos RD. Review: PCSK9 inhibitors reduce mortality but increase neurocognitive events in hypercholesterolemia Ann Intern Med. 2016 Mar 15;164(6):JC31. doi: 10.7326/ACPJC-2016-164-6-031.
922. Wang C, Wang X, Chi C, Guo L, Guo L, Zhao N, Wang W, Pi X, Sun B, Lian A, Shi J, Li E. Lung ventilation strategies for acute respiratory distress syndrome: a systematic review and network meta-analysis Sci Rep. 2016 Mar 9;6:22855. doi: 10.1038/srep22855.
923. Catalá-López F, Macías Saint-Gerons D, González-Bermejo D, Rosano GM, Davis BR, Ridao M, Zaragoza A, Montero-Corominas D, Tobías A, de la Fuente-Honrubia C, Tabarés-Seisdedos R, Hutton B. Cardiovascular and Renal Outcomes of Renin-Angiotensin System Blockade in Adult Patients with Diabetes Mellitus: A Systematic Review with Network Meta-Analyses PLoS Med. 2016 Mar 8;13(3):e1001971. doi: 10.1371/journal.pmed.1001971. eCollection 2016 Mar.
924. Zhu GQ, Zou ZL, Zheng JN, Chen DZ, Zou TT, Shi KQ, Zheng MH. Systematic Review and Network Meta-Analysis of Randomized Controlled Trials: Comparative Effectiveness and Safety of Direct-Acting Antiviral Agents for Treatment-Naive Hepatitis C Genotype 1 Medicine (Baltimore). 2016 Mar;95(9):e3004. doi: 10.1097/MD.0000000000003004.
925. Shao JB, Ni CF, Duan PF, Jin YH. Preventive Effects of Different Drugs on Asymptomatic Lower Extremities Deep Venous Thrombosis After Artificial Joint Replacement: A Mixed Treatment Comparison Am J Ther. 2019 Jan/Feb;26(1):e45-e53. doi: 10.1097/MJT.0000000000000438.
926. Panikker S, Lord J, Jarman JW, Armstrong S, Jones DG, Haldar S, Butcher C, Khan H, Mantziari L, Nicol E, Hussain W, Clague JR, Foran JP, Markides V, Wong T. Outcomes and costs of left atrial appendage closure from randomized controlled trial and real-world experience relative to oral anticoagulation Eur Heart J. 2016 Dec 7;37(46):3470-3482. doi: 10.1093/eurheartj/ehw048. Epub 2016 Mar 1.
927. Zhang Y, Sheng J, Yang Y, Fang W, Kang S, He Y, Hong S, Zhan J, Zhao Y, Xue C, Ma Y, Zhou T, Ma S, Gao F, Qin T, Hu Z, Tian Y, Hou X, Huang Y, Zhou N, Zhao H, Zhang L. Optimized selection of three major EGFR-TKIs in advanced EGFR-positive non-small cell lung cancer: a network meta-analysis Oncotarget. 2016 Apr 12;7(15):20093-108. doi: 10.18632/oncotarget.7713.
928. Tsiami A, Chaimani A, Mavridis D, Siskou M, Assimakopoulos E, Sotiriadis A. Surgical treatment for hydrosalpinx prior to in-vitro fertilization embryo transfer: a network meta-analysis Ultrasound Obstet Gynecol. 2016 Oct;48(4):434-445. doi: 10.1002/uog.15900. Epub 2016 Sep 13.
929. Lanitis T, Leipold R, Hamilton M, Rublee D, Quon P, Browne C, Cohen AT. Cost-effectiveness of Apixaban Versus Other Oral Anticoagulants for the Initial Treatment of Venous Thromboembolism and Prevention of Recurrence Clin Ther. 2016 Mar;38(3):478-93.e1-16. doi: 10.1016/j.clinthera.2016.01.020. Epub 2016 Feb 26.
930. Liang X, Fan R, Sun J, Shaikh J, Taneja A, Gupta S, Hamed K. Effect of Telbivudine Versus Other Nucleos(t)ide Analogs on HBeAg Seroconversion and Other Outcomes in Patients with Chronic Hepatitis B: A Network Meta-Analysis Adv Ther. 2016 Apr;33(4):519-31. doi: 10.1007/s12325-016-0305-x. Epub 2016 Feb 26.
931. Gurusamy KS, Vaughan J, Fraser IS, Best LM, Richards T. Medical Therapies for Uterine Fibroids - A Systematic Review and Network Meta-Analysis of Randomised Controlled Trials PLoS One. 2016 Feb 26;11(2):e0149631. doi: 10.1371/journal.pone.0149631. eCollection 2016.
932. Schwendicke F, Göstemeyer G, Blunck U, Paris S, Hsu LY, Tu YK. Directly Placed Restorative Materials: Review and Network Meta-analysis J Dent Res. 2016 Jun;95(6):613-22. doi: 10.1177/0022034516631285. Epub 2016 Feb 24.
933. Shyangdan DS, Uthman OA, Waugh N. SGLT-2 receptor inhibitors for treating patients with type 2 diabetes mellitus: a systematic review and network meta-analysis BMJ Open. 2016 Feb 24;6(2):e009417. doi: 10.1136/bmjopen-2015-009417.
934. Freemantle N, Ginsberg DA, McCool R, Fleetwood K, Arber M, Khalaf K, Loveman C, Ni Q, Glanville J. Comparative assessment of onabotulinumtoxinA and mirabegron for overactive bladder: an indirect treatment comparison BMJ Open. 2016 Feb 23;6(2):e009122. doi: 10.1136/bmjopen-2015-009122.
935. Chang X, Zhang F, Liu T, Yang R, Ji C, Zhao X, Xu L, Liu G, Guo H. Comparative efficacy and safety of first-line treatments in patients with metastatic renal cell cancer: a network meta-analysis based on phase 3 RCTs Oncotarget. 2016 Mar 29;7(13):15801-10. doi: 10.18632/oncotarget.7511.
936. Migliore A, Bizzi E, Petrella L, Bruzzese V, Cassol M, Integlia D. The Challenge of Treating Early-Stage Rheumatoid Arthritis: The Contribution of Mixed Treatment Comparison to Choosing Appropriate Biologic Agents BioDrugs. 2016 Apr;30(2):105-15. doi: 10.1007/s40259-016-0164-7.
937. Sheng M, Zhao Y, Wang F, Li S, Wang X, Shou T, Luo Y, Tang W. Targeted drugs for unselected patients with advanced non-small-cell lung cancer: a network meta-analysis J Thorac Dis. 2016 Jan;8(1):98-115. doi: 10.3978/j.issn.2072-1439.2016.01.28.
938. Kayaniyil S, Lozano-Ortega G, Bennett HA, Johnsson K, Shaunik A, Grandy S, Kartman B. A Network Meta-analysis Comparing Exenatide Once Weekly with Other GLP-1 Receptor Agonists for the Treatment of Type 2 Diabetes Mellitus Diabetes Ther. 2016 Mar;7(1):27-43. doi: 10.1007/s13300-016-0155-1. Epub 2016 Feb 17.
939. Yin S, Zhang D, Du H, Du H, Yin Z, Qiu Y. Is there any difference in survivorship of total hip arthroplasty with different bearing surfaces? A systematic review and network meta-analysis Int J Clin Exp Med. 2015 Nov 15;8(11):21871-85. eCollection 2015.
940. Freemantle N, Chou E, Frois C, Zhuo D, Lehmacher W, Vlajnic A, Wang H, Chung HW, Zhang Q, Wu E, Gerrits C. Safety and efficacy of insulin glargine 300 u/mL compared with other basal insulin therapies in patients with type 2 diabetes mellitus: a network meta-analysis BMJ Open. 2016 Feb 15;6(2):e009421. doi: 10.1136/bmjopen-2015-009421.
941. Sun W, Zhang H, Guo J, Zhang X, Zhang L, Li C, Zhang L. Comparison of the Efficacy and Safety of Different ACE Inhibitors in Patients With Chronic Heart Failure: A PRISMA-Compliant Network Meta-Analysis Medicine (Baltimore). 2016 Feb;95(6):e2554. doi: 10.1097/MD.0000000000002554.
942. Giustino G, Mehran R. Drug-eluting stents and drug-eluting balloons are the best strategies to treat coronary in-stent restenosis Evid Based Med. 2016 Jun;21(3):90. doi: 10.1136/ebmed-2015-110372. Epub 2016 Feb 10.
943. Lehmacher W, Klasser M, Duering A. Letter to the editor: no increased febrile neutropenia risk to cancer patients receiving lipegfilgrastim--correction of a systematic review and meta-analysis of randomized controlled trials with G-CSF Support Care Cancer. 2016 May;24(5):1935-1937. doi: 10.1007/s00520-016-3116-3. Epub 2016 Feb 10.
944. Wang W, Zhang L, Liu W, Zhu Q, Lan Q, Zhao J. Antiplatelet Agents for the Secondary Prevention of Ischemic Stroke or Transient Ischemic Attack: A Network Meta-Analysis J Stroke Cerebrovasc Dis. 2016 May;25(5):1081-1089. doi: 10.1016/j.jstrokecerebrovasdis.2016.01.026. Epub 2016 Feb 5.
945. Lotrionte M, Biasucci LM, Peruzzi M, Frati G, Giordano A, Biondi-Zoccai G. Which Aspirin Dose and Preparation Is Best for the Long-Term Prevention of Cardiovascular Disease and Cancer? Evidence From a Systematic Review and Network Meta-Analysis Prog Cardiovasc Dis. 2016 Mar-Apr;58(5):495-504. doi: 10.1016/j.pcad.2016.02.001. Epub 2016 Feb 4.
946. Zhao BC, Jiang HY, Ma WY, Jin DD, Li HM, Lu H, Nakajima H, Huang TY, Sun KY, Chen SL, Chen KB. Albendazole and Corticosteroids for the Treatment of Solitary Cysticercus Granuloma: A Network Meta-analysis PLoS Negl Trop Dis. 2016 Feb 5;10(2):e0004418. doi: 10.1371/journal.pntd.0004418. eCollection 2016 Feb.
947. Corbett M, Soares M, Jhuti G, Rice S, Spackman E, Sideris E, Moe-Byrne T, Fox D, Marzo-Ortega H, Kay L, Woolacott N, Palmer S. Tumour necrosis factor-α inhibitors for ankylosing spondylitis and non-radiographic axial spondyloarthritis: a systematic review and economic evaluation Health Technol Assess. 2016 Feb;20(9):1-334, v-vi. doi: 10.3310/hta20090.
948. Huang HP, Lin WH, Chen SG, Chen LZ, Chen MY, Che CH. Comparative Efficacy and Safety of Nine Anti-Platelet Therapies for Patients with Ischemic Stroke or Transient Ischemic Attack: a Mixed Treatment Comparisons Mol Neurobiol. 2017 Mar;54(2):1456-1466. doi: 10.1007/s12035-016-9739-z. Epub 2016 Feb 5.
949. Smith SR, Deshpande BR, Collins JE, Katz JN, Losina E. Comparative pain reduction of oral non-steroidal anti-inflammatory drugs and opioids for knee osteoarthritis: systematic analytic review Osteoarthritis Cartilage. 2016 Jun;24(6):962-72. doi: 10.1016/j.joca.2016.01.135. Epub 2016 Feb 1.
950. Rochwerg B, Neupane B, Zhang Y, Garcia CC, Raghu G, Richeldi L, Brozek J, Beyene J, Schünemann H. Treatment of idiopathic pulmonary fibrosis: a network meta-analysis BMC Med. 2016 Feb 3;14:18. doi: 10.1186/s12916-016-0558-x.
951. Kane JM, Correll CU. The Role of Clozapine in Treatment-Resistant Schizophrenia JAMA Psychiatry. 2016 Mar;73(3):187-8. doi: 10.1001/jamapsychiatry.2015.2966.
952. Steurer J. [COPD: LAMA / LABA fixed combination more effective than separately ] Praxis (Bern 1994). 2016 Feb 3;105(3):167-8. doi: 10.1024/1661-8157/a002260.
953. Raccah BH, Perlman A, Danenberg HD, Pollak A, Muszkat M, Matok I. Major Bleeding and Hemorrhagic Stroke With Direct Oral Anticoagulants in Patients With Renal Failure: Systematic Review and Meta-Analysis of Randomized Trials Chest. 2016 Jun;149(6):1516-24. doi: 10.1016/j.chest.2015.12.029. Epub 2016 Jan 18.
954. Canestaro WJ, Forrester SH, Raghu G, Ho L, Devine BE. Drug Treatment of Idiopathic Pulmonary Fibrosis: Systematic Review and Network Meta-Analysis Chest. 2016 Mar;149(3):756-66. doi: 10.1016/j.chest.2015.11.013. Epub 2016 Jan 13.
955. Lipka S, Kumar A, Miladinovic B, Richter JE. Systematic review with network meta-analysis: comparative effectiveness of topical steroids vs. PPIs for the treatment of the spectrum of eosinophilic oesophagitis Aliment Pharmacol Ther. 2016 Mar;43(6):663-73. doi: 10.1111/apt.13537. Epub 2016 Feb 1.
956. Huang J, Wen D, Wang Q, McAlinden C, Flitcroft I, Chen H, Saw SM, Chen H, Bao F, Zhao Y, Hu L, Li X, Gao R, Lu W, Du Y, Jinag Z, Yu A, Lian H, Jiang Q, Yu Y, Qu J. Efficacy Comparison of 16 Interventions for Myopia Control in Children: A Network Meta-analysis Ophthalmology. 2016 Apr;123(4):697-708. doi: 10.1016/j.ophtha.2015.11.010. Epub 2016 Jan 27.
957. Zhang H, Li X, Huang J, Li H, Su Z, Wang J. Comparative Efficacy and Safety of Prostacyclin Analogs for Pulmonary Arterial Hypertension: A Network Meta-Analysis Medicine (Baltimore). 2016 Jan;95(4):e2575. doi: 10.1097/MD.0000000000002575.
958. Liu B, Duan CY, Luo CF, Ou CW, Wu ZY, Zhang JW, Ni XB, Chen PY, Chen MS. Impact of Timing following Acute Myocardial Infarction on Efficacy and Safety of Bone Marrow Stem Cells Therapy: A Network Meta-Analysis Stem Cells Int. 2016;2016:1031794. doi: 10.1155/2016/1031794. Epub 2015 Dec 13.
959. Amiche MA, Albaum JM, Tadrous M, Pechlivanoglou P, Lévesque LE, Adachi JD, Cadarette SM. Efficacy of osteoporosis pharmacotherapies in preventing fracture among oral glucocorticoid users: a network meta-analysis Osteoporos Int. 2016 Jun;27(6):1989-98. doi: 10.1007/s00198-015-3476-4. Epub 2016 Jan 18.
960. Bonsu KO, Reidpath DD, Kadirvelu A. Lipophilic Statin Versus Rosuvastatin (Hydrophilic) Treatment for Heart Failure: a Meta-Analysis and Adjusted Indirect Comparison of Randomised Trials Cardiovasc Drugs Ther. 2016 Apr;30(2):177-88. doi: 10.1007/s10557-015-6636-z.
961. Inácio JF, da Rosa Mdos S, Shah J, Rosário J, Vissoci JR, Manica AL, Rodrigues CG. Monophasic and biphasic shock for transthoracic conversion of atrial fibrillation: Systematic review and network meta-analysis Resuscitation. 2016 Mar;100:66-75. doi: 10.1016/j.resuscitation.2015.12.009. Epub 2016 Jan 8.
962. Song GG, Seo YH, Kim JH, Choi SJ, Ji JD, Lee YH. Relative efficacy and tolerability of etoricoxib, celecoxib, and naproxen in the treatment of osteoarthritis : A Bayesian network meta-analysis of randomized controlled trials based on patient withdrawal Z Rheumatol. 2016 Jun;75(5):508-16. doi: 10.1007/s00393-015-0023-9.
963. Quon P, Le HH, Raymond V, Mtibaa M, Moshyk A. Clinical and economic benefits of extended treatment with apixaban for the treatment and prevention of recurrent venous thromboembolism in Canada J Med Econ. 2016 Jun;19(6):557-67. doi: 10.3111/13696998.2016.1141780. Epub 2016 Feb 3.
964. Blanchard P, Ribassin-Majed L, Lee A, Pignon JP. Comment on "Chemoradiotherapy regimens for locoregionally advanced nasopharyngeal carcinoma: a Bayesian network meta-analysis", published in Eur J Cancer 51 (2015), 1570-1579 Eur J Cancer. 2016 Mar;56:183-185. doi: 10.1016/j.ejca.2015.10.067. Epub 2016 Jan 2.
965. Guo JL, Zhang DD, Zhao Y, Zhang D, Zhang XM, Zhou CQ, Yao SZ. Pharmacologic Interventions in Preventing Ovarian Hyperstimulation Syndrome: A Systematic Review and Network Meta-Analysis Sci Rep. 2016 Jan 11;6:19093. doi: 10.1038/srep19093.
966. Schlueter M, Gonzalez-Rojas N, Baldwin M, Groenke L, Voss F, Reason T. Comparative efficacy of fixed-dose combinations of long-acting muscarinic antagonists and long-acting β2-agonists: a systematic review and network meta-analysis Ther Adv Respir Dis. 2016 Apr;10(2):89-104. doi: 10.1177/1753465815624612. Epub 2016 Jan 8.
967. Mosseri J, Trinquart L, Nizard R, Ravaud P. Meta-Analysis of a Complex Network of Non-Pharmacological Interventions: The Example of Femoral Neck Fracture PLoS One. 2016 Jan 6;11(1):e0146336. doi: 10.1371/journal.pone.0146336. eCollection 2016.
968. Wu X, Chung VC, Lu P, Poon SK, Hui EP, Lau AY, Balneaves LG, Wong SY, Wu JC. Chinese Herbal Medicine for Improving Quality of Life Among Nonsmall Cell Lung Cancer Patients: Overview of Systematic Reviews and Network Meta-Analysis Medicine (Baltimore). 2016 Jan;95(1):e2410. doi: 10.1097/MD.0000000000002410.
969. Amdahl J, Chen L, Delea TE. Network Meta-analysis of Progression-Free Survival and Overall Survival in First-Line Treatment of BRAF Mutation-Positive Metastatic Melanoma Oncol Ther. 2016;4(2):239-256. doi: 10.1007/s40487-016-0030-2. Epub 2016 Sep 27.
970. Suwanthawornkul T, Anothaisintawee T, Sobhonslidsuk A, Thakkinstian A, Teerawattananon Y. Efficacy of Second Generation Direct-Acting Antiviral Agents for Treatment Naïve Hepatitis C Genotype 1: A Systematic Review and Network Meta-Analysis PLoS One. 2015 Dec 31;10(12):e0145953. doi: 10.1371/journal.pone.0145953. eCollection 2015.
971. Xie W, Zheng F, Song X, Zhong B, Yan L. Renin-angiotensin-aldosterone system blockers for heart failure with reduced ejection fraction or left ventricular dysfunction: Network meta-analysis Int J Cardiol. 2016 Feb 15;205:65-71. doi: 10.1016/j.ijcard.2015.12.010. Epub 2015 Dec 15.
972. Cohen AT, Hamilton M, Mitchell SA, Phatak H, Liu X, Bird A, Tushabe D, Batson S. Comparison of the Novel Oral Anticoagulants Apixaban, Dabigatran, Edoxaban, and Rivaroxaban in the Initial and Long-Term Treatment and Prevention of Venous Thromboembolism: Systematic Review and Network Meta-Analysis PLoS One. 2015 Dec 30;10(12):e0144856. doi: 10.1371/journal.pone.0144856. eCollection 2015.
973. Lozano-Ortega G, Goring S, Bennett HA, Bergenheim K, Sternhufvud C, Mukherjee J. Network meta-analysis of treatments for type 2 diabetes mellitus following failure with metformin plus sulfonylurea Curr Med Res Opin. 2016 May;32(5):807-16. doi: 10.1185/03007995.2015.1135110. Epub 2016 Feb 29.
974. Rognoni C, Venturini S, Meregaglia M, Marmifero M, Tarricone R. Efficacy and Safety of Ferric Carboxymaltose and Other Formulations in Iron-Deficient Patients: A Systematic Review and Network Meta-analysis of Randomised Controlled Trials Clin Drug Investig. 2016 Mar;36(3):177-94. doi: 10.1007/s40261-015-0361-z.
975. Kobayashi H, Ohnishi T, Nakagawa R, Yoshizawa K. The comparative efficacy and safety of cholinesterase inhibitors in patients with mild-to-moderate Alzheimer's disease: a Bayesian network meta-analysis Int J Geriatr Psychiatry. 2016 Aug;31(8):892-904. doi: 10.1002/gps.4405. Epub 2015 Dec 17.
976. Bardakos NV. CORR (®) Insights: Is Local Infiltration Analgesia Superior to Peripheral Nerve Blockade for Pain Management after THA: A Network Meta-analysis Clin Orthop Relat Res. 2016 Feb;474(2):517-9. doi: 10.1007/s11999-015-4664-4. Epub 2015 Dec 16.
977. Tornyos A, Kehl D, D'Ascenzo F, Komócsi A. Risk of Myocardial Infarction in Patients with Long-Term Non-Vitamin K Antagonist Oral Anticoagulant Treatment Prog Cardiovasc Dis. 2016 Mar-Apr;58(5):483-94. doi: 10.1016/j.pcad.2015.12.001. Epub 2015 Dec 7.
978. Li D, Wang T, Shen S, Cheng S, Yu J, Zhang Y, Zhang C, Tang H. Effects of Fluroquinolones in Newly Diagnosed, Sputum-Positive Tuberculosis Therapy: A Systematic Review and Network Meta-Analysis PLoS One. 2015 Dec 15;10(12):e0145066. doi: 10.1371/journal.pone.0145066. eCollection 2015.
979. Downes MJ, Bettington EK, Gunton JE, Turkstra E. Triple therapy in type 2 diabetes; a systematic review and network meta-analysis PeerJ. 2015 Dec 7;3:e1461. doi: 10.7717/peerj.1461. eCollection 2015.
980. McCarthy M. Comparing once weekly GLP-1 receptor agonists for type 2 diabetes finds no clear winner BMJ. 2015 Dec 9;351:h6702. doi: 10.1136/bmj.h6702.
981. Xue C, Zhou C, Dai B, Yu S, Xu C, Mao Z, Ye C, Chen D, Zhao X, Wu J, Chen W, Mei C. Antihypertensive treatments in adult autosomal dominant polycystic kidney disease: network meta-analysis of the randomized controlled trials Oncotarget. 2015 Dec 15;6(40):42515-29. doi: 10.18632/oncotarget.6452.
982. Tarp S, Amarilyo G, Foeldvari I, Christensen R, Woo JM, Cohen N, Pope TD, Furst DE. Efficacy and safety of biological agents for systemic juvenile idiopathic arthritis: a systematic review and meta-analysis of randomized trials Rheumatology (Oxford). 2016 Apr;55(4):669-79. doi: 10.1093/rheumatology/kev382. Epub 2015 Nov 30.
983. Wade R, Sideris E, Paton F, Rice S, Palmer S, Fox D, Woolacott N, Spackman E. Graduated compression stockings for the prevention of deep-vein thrombosis in postoperative surgical patients: a systematic review and economic model with a value of information analysis Health Technol Assess. 2015 Nov;19(98):1-220. doi: 10.3310/hta19980.
984. Barbato L, Kalemaj Z, Buti J, Baccini M, La Marca M, Duvina M, Tonelli P. Effect of Surgical Intervention for Removal of Mandibular Third Molar on Periodontal Healing of Adjacent Mandibular Second Molar: A Systematic Review and Bayesian Network Meta-Analysis J Periodontol. 2016 Mar;87(3):291-302. doi: 10.1902/jop.2015.150363. Epub 2015 Nov 26.
985. Simillis C, Robertson FP, Afxentiou T, Davidson BR, Gurusamy KS. A network meta-analysis comparing perioperative outcomes of interventions aiming to decrease ischemia reperfusion injury during elective liver resection Surgery. 2016 Apr;159(4):1157-69. doi: 10.1016/j.surg.2015.10.011. Epub 2015 Nov 19.
986. Ismaila AS, Huisman EL, Punekar YS, Karabis A. Comparative efficacy of long-acting muscarinic antagonist monotherapies in COPD: a systematic review and network meta-analysis Int J Chron Obstruct Pulmon Dis. 2015 Nov 16;10:2495-517. doi: 10.2147/COPD.S92412. eCollection 2015.
987. Xie X, Liu Y, Perkovic V, Li X, Ninomiya T, Hou W, Zhao N, Liu L, Lv J, Zhang H, Wang H. Renin-Angiotensin System Inhibitors and Kidney and Cardiovascular Outcomes in Patients With CKD: A Bayesian Network Meta-analysis of Randomized Clinical Trials Am J Kidney Dis. 2016 May;67(5):728-41. doi: 10.1053/j.ajkd.2015.10.011. Epub 2015 Nov 18.
988. Chen PC, Chuang CH, Tu YK, Bai CH, Chen CF, Liaw M. A Bayesian network meta-analysis: Comparing the clinical effectiveness of local corticosteroid injections using different treatment strategies for carpal tunnel syndrome BMC Musculoskelet Disord. 2015 Nov 19;16:363. doi: 10.1186/s12891-015-0815-8.
989. Mocellin S, Pilati P, Briarava M, Nitti D. Breast Cancer Chemoprevention: A Network Meta-Analysis of Randomized Controlled Trials J Natl Cancer Inst. 2015 Nov 18;108(2):djv318. doi: 10.1093/jnci/djv318. Print 2016 Feb.
990. Zhang T, Ye X, Zhu T, Xiao X, Liu Y, Wei X, Liu Y, Wu C, Guan R, Li X, Guo X, Hu H, He J. Antithrombotic Treatment for Recurrent Miscarriage: Bayesian Network Meta-Analysis and Systematic Review Medicine (Baltimore). 2015 Nov;94(45):e1732. doi: 10.1097/MD.0000000000001732.
991. Scott DA, Woods B, Thompson JC, Clark JF, Hawkins N, Chambers M, Celli BR, Calverley P. Mortality and drug therapy in patients with chronic obstructive pulmonary disease: a network meta-analysis BMC Pulm Med. 2015 Nov 11;15:145. doi: 10.1186/s12890-015-0138-4.
992. Qiu WJ, Li YF, Ji YH, Xu W, Zhu XD, Tang XZ, Zhao HL, Wang GB, Jia YQ, Zhu SC, Zhang FF, Liu HM. The comparative risk of developing postoperative complications in patients with distal radius fractures following different treatment modalities Sci Rep. 2015 Nov 9;5:15318. doi: 10.1038/srep15318.
993. Sethi A, Malhotra G, Singh S, Singh PP, Khosla S. Efficacy of various percutaneous interventions for in-stent restenosis: comprehensive network meta-analysis of randomized controlled trials Circ Cardiovasc Interv. 2015 Nov;8(11):e002778. doi: 10.1161/CIRCINTERVENTIONS.115.002778.
994. Glue P, Herbison P. Comparative efficacy and acceptability of combined antipsychotics and mood stabilizers versus individual drug classes for acute mania: Network meta-analysis Aust N Z J Psychiatry. 2015 Dec;49(12):1215-20. doi: 10.1177/0004867415614109. Epub 2015 Nov 6.
995. Zhao YJ, Khoo AL, Tan G, Teng M, Tee C, Tan BH, Ong B, Lim BP, Chai LY. Network Meta-analysis and Pharmacoeconomic Evaluation of Fluconazole, Itraconazole, Posaconazole, and Voriconazole in Invasive Fungal Infection Prophylaxis Antimicrob Agents Chemother. 2015 Nov 2;60(1):376-86. doi: 10.1128/AAC.01985-15. Print 2016 Jan.
996. Linde K, Rücker G, Schneider A, Kriston L. Questionable assumptions hampered interpretation of a network meta-analysis of primary care depression treatments J Clin Epidemiol. 2016 Mar;71:86-96. doi: 10.1016/j.jclinepi.2015.10.010. Epub 2015 Oct 30.
997. Pike E, Torkilseng EB, Saeterdal I, Jimenez E, Odgaard-Jensen J, Harboe I, Klemp M. A Health Technology Assessment of the New Drugs for Inoperable or Metastatic Malignant Melanoma Patients Oslo, Norway: Knowledge Centre for the Health Services at The Norwegian Institute of Public Health (NIPH); 2015 Nov. Report from Norwegian Knowledge Centre for the Health Services (NOKC) No. 22-2015.
998. Arya R, Kothari H, Zhang Z, Han B, Horn PS, Glauser TA. Efficacy of nonvenous medications for acute convulsive seizures: A network meta-analysis Neurology. 2015 Nov 24;85(21):1859-68. doi: 10.1212/WNL.0000000000002142. Epub 2015 Oct 28.
999. Tricco AC, Strifler L, Veroniki AA, Yazdi F, Khan PA, Scott A, Ng C, Antony J, Mrklas K, D'Souza J, Cardoso R, Straus SE. Comparative safety and effectiveness of long-acting inhaled agents for treating chronic obstructive pulmonary disease: a systematic review and network meta-analysis BMJ Open. 2015 Oct 26;5(10):e009183. doi: 10.1136/bmjopen-2015-009183.
1000. Feng W, Wu K, Liu Z, Kong G, Deng Z, Chen S, Wu Y, Chen M, Liu S, Wang H. Oral direct factor Xa inhibitor versus enoxaparin for thromboprophylaxis after hip or knee arthroplasty: Systemic review, traditional meta-analysis, dose-response meta-analysis and network meta-analysis Thromb Res. 2015 Dec;136(6):1133-44. doi: 10.1016/j.thromres.2015.10.009. Epub 2015 Oct 20.
1001. Zhao X, Liu L, Li K, Li W, Zhao L, Zou H. Comparative study on individual aromatase inhibitors on cardiovascular safety profile: a network meta-analysis Onco Targets Ther. 2015 Sep 29;8:2721-30. doi: 10.2147/OTT.S88179. eCollection 2015.
1002. Steenhuis LA, Nauta MH, Bocking CL, Pijnenborg GH. Treating Depressive Symptoms in Psychosis: A Network Meta-Analysis on the Effects of Non-Verbal Therapies PLoS One. 2015 Oct 20;10(10):e0140637. doi: 10.1371/journal.pone.0140637. eCollection 2015.
1003. Naci H, Ioannidis JP. Comparative effectiveness of exercise and drug interventions on mortality outcomes: metaepidemiological study Br J Sports Med. 2015 Nov;49(21):1414-22. doi: 10.1136/bjsports-2015-f5577rep.
1004. Firwana B, Sonbol MB, Diab M, Raza S, Hasan R, Yousef I, Zarzour A, Garipalli A, Doll D, Murad MH, Al-Kali A. Tyrosine kinase inhibitors as a first-line treatment in patients with newly diagnosed chronic myeloid leukemia in chronic phase: A mixed-treatment comparison Int J Cancer. 2016 Mar 15;138(6):1545-53. doi: 10.1002/ijc.29889. Epub 2015 Oct 28.
1005. Singh S, Singh M, Grewal N, Khosla S. Comparative Efficacy and Safety of Prasugrel, Ticagrelor, and Standard-Dose and High-Dose Clopidogrel in Patients Undergoing Percutaneous Coronary Intervention: A Network Meta-analysis Am J Ther. 2016 Jan-Feb;23(1):e52-62. doi: 10.1097/MJT.0000000000000350.
1006. Yang M, Wang HT, Zhao M, Meng WB, Ou JQ, He JH, Zou B, Lei PG. Network Meta-Analysis Comparing Relatively Selective COX-2 Inhibitors Versus Coxibs for the Prevention of NSAID-Induced Gastrointestinal Injury Medicine (Baltimore). 2015 Oct;94(40):e1592. doi: 10.1097/MD.0000000000001592.
1007. Hutton B, Clemons M, Mazzarello S, Kuchuk I, Skidmore B, Ng T. Identifying an optimal antiemetic regimen for patients receiving anthracycline and cyclophosphamide-based chemotherapy for breast cancer--an inspection of the evidence base informing clinical decision-making Cancer Treat Rev. 2015 Dec;41(10):951-9. doi: 10.1016/j.ctrv.2015.09.007. Epub 2015 Sep 30.
1008. Petrou P, Dias S. A mixed treatment comparison for short- and long-term outcomes of bare-metal and drug-eluting coronary stents Int J Cardiol. 2016 Jan 1;202:448-62. doi: 10.1016/j.ijcard.2015.08.134. Epub 2015 Aug 22.
1009. Simillis C, Thoukididou SN, Slesser AA, Rasheed S, Tan E, Tekkis PP. Systematic review and network meta-analysis comparing clinical outcomes and effectiveness of surgical treatments for haemorrhoids Br J Surg. 2015 Dec;102(13):1603-18. doi: 10.1002/bjs.9913. Epub 2015 Sep 30.
1010. Pillay J, Armstrong MJ, Butalia S, Donovan LE, Sigal RJ, Vandermeer B, Chordiya P, Dhakal S, Hartling L, Nuspl M, Featherstone R, Dryden DM. Behavioral Programs for Type 2 Diabetes Mellitus: A Systematic Review and Network Meta-analysis Ann Intern Med. 2015 Dec 1;163(11):848-60. doi: 10.7326/M15-1400. Epub 2015 Sep 29.
1011. Bucher J, Koyfman A. What Is the Most Effective Treatment of Herpes Simplex Keratitis? Ann Emerg Med. 2016 Jul;68(1):26-7. doi: 10.1016/j.annemergmed.2015.08.017. Epub 2015 Sep 26.
1012. Fujii T, Le Du F, Xiao L, Kogawa T, Barcenas CH, Alvarez RH, Valero V, Shen Y, Ueno NT. Effectiveness of an Adjuvant Chemotherapy Regimen for Early-Stage Breast Cancer: A Systematic Review and Network Meta-analysis JAMA Oncol. 2015 Dec;1(9):1311-8. doi: 10.1001/jamaoncol.2015.3062.
1013. Huisman EL, Cockle SM, Ismaila AS, Karabis A, Punekar YS. Comparative efficacy of combination bronchodilator therapies in COPD: a network meta-analysis Int J Chron Obstruct Pulmon Dis. 2015 Sep 9;10:1863-81. doi: 10.2147/COPD.S87082. eCollection 2015.
1014. Dong W, Goost H, Lin XB, Burger C, Paul C, Wang ZL, Kong FL, Welle K, Jiang ZC, Kabir K. Injection therapies for lateral epicondylalgia: a systematic review and Bayesian network meta-analysis Br J Sports Med. 2016 Aug;50(15):900-8. doi: 10.1136/bjsports-2014-094387. Epub 2015 Sep 21.
1015. Zhao HL, Wang GB, Jia YQ, Zhu SC, Zhang FF, Liu HM. Comparison of Risk of Carpal Tunnel Syndrome in Patients with Distal Radius Fractures After 7 Treatments Med Sci Monit. 2015 Sep 22;21:2837-44. doi: 10.12659/MSM.894075.
1016. Tramacere I, Del Giovane C, Salanti G, D'Amico R, Filippini G. Immunomodulators and immunosuppressants for relapsing-remitting multiple sclerosis: a network meta-analysis Cochrane Database Syst Rev. 2015 Sep 18;(9):CD011381. doi: 10.1002/14651858.CD011381.pub2.
1017. Zhu GQ, Shi KQ, Huang GQ, Wang LR, Lin YQ, Braddock M, Chen YP, Zhou MT, Zheng MH. A network meta-analysis of the efficacy and side effects of UDCA-based therapies for primary sclerosing cholangitis Oncotarget. 2015 Sep 29;6(29):26757-69. doi: 10.18632/oncotarget.5610.
1018. Migliore A, Bizzi E, Egan CG, Bernardi M, Petrella L. Efficacy of biological agents administered as monotherapy in rheumatoid arthritis: a Bayesian mixed-treatment comparison analysis Ther Clin Risk Manag. 2015 Sep 1;11:1325-35. doi: 10.2147/TCRM.S89678. eCollection 2015.
1019. Tan PS, Lopes G, Acharyya S, Bilger M, Haaland B. Bayesian network meta-comparison of maintenance treatments for stage IIIb/IV non-small-cell lung cancer (NSCLC) patients with good performance status not progressing after first-line induction chemotherapy: results by performance status, EGFR mutation, histology and response to previous induction Eur J Cancer. 2015 Nov;51(16):2330-44. doi: 10.1016/j.ejca.2015.07.007. Epub 2015 Sep 10.
1020. Lehert P, Villaseca P, Hogervorst E, Maki PM, Henderson VW. Individually modifiable risk factors to ameliorate cognitive aging: a systematic review and meta-analysis Climacteric. 2015 Oct;18(5):678-89. doi: 10.3109/13697137.2015.1078106. Epub 2015 Sep 11.
1021. Sun F, Wu S, Guo S, Yu K, Yang Z, Li L, Zhang Y, Quan X, Ji L, Zhan S. Impact of GLP-1 receptor agonists on blood pressure, heart rate and hypertension among patients with type 2 diabetes: A systematic review and network meta-analysis Diabetes Res Clin Pract. 2015 Oct;110(1):26-37. doi: 10.1016/j.diabres.2015.07.015. Epub 2015 Aug 13.
1022. Ullman AJ, Cooke ML, Mitchell M, Lin F, New K, Long DA, Mihala G, Rickard CM. Dressings and securement devices for central venous catheters (CVC) Cochrane Database Syst Rev. 2015 Sep 10;2015(9):CD010367. doi: 10.1002/14651858.CD010367.pub2.
1023. Dong H, Pi F, Ding Z, Chen W, Pang S, Dong W, Zhang Q. Efficacy of Supplementation with B Vitamins for Stroke Prevention: A Network Meta-Analysis of Randomized Controlled Trials PLoS One. 2015 Sep 10;10(9):e0137533. doi: 10.1371/journal.pone.0137533. eCollection 2015.
1024. Jiang J, Zou J, Ma H, Jiao Y, Yang H, Zhang X, Miao Y. Network Meta-analysis of Randomized Trials on the Safety of Vascular Closure Devices for Femoral Arterial Puncture Site Haemostasis Sci Rep. 2015 Sep 8;5:13761. doi: 10.1038/srep13761.
1025. Yamaguchi N, Fujii T, Aoi S, Kozuch PS, Hortobagyi GN, Blum RH. Comparison of cardiac events associated with liposomal doxorubicin, epirubicin and doxorubicin in breast cancer: a Bayesian network meta-analysis Eur J Cancer. 2015 Nov;51(16):2314-20. doi: 10.1016/j.ejca.2015.07.031. Epub 2015 Sep 3.
1026. Chevalier B. In-stent restenosis treatment: quest for the best device Lancet. 2015 Aug 15;386(9994):627-8. doi: 10.1016/S0140-6736(15)60939-4.
1027. Police RL, Trask PC, Wang J, Olivares R, Khan S, Abbe A, Colosia A, Njue A, Sherril B, Ruiz-Soto R, Kaye JA, Hamadani M. Randomized controlled trials in relapsed/refractory follicular lymphoma: a systematic review and meta-analysis J Oncol Pharm Pract. 2016 Oct;22(5):666-78. doi: 10.1177/1078155215603230. Epub 2015 Aug 28.
1028. Nassar AP Jr, Zampieri FG, Ranzani OT, Park M. Protocolized sedation effect on post-ICU posttraumatic stress disorder prevalence: A systematic review and network meta-analysis J Crit Care. 2015 Dec;30(6):1278-82. doi: 10.1016/j.jcrc.2015.07.023. Epub 2015 Jul 29.
1029. Xie W, Zheng F, Zhong B, Song X. Long-Term Antiplatelet Mono- and Dual Therapies After Ischemic Stroke or Transient Ischemic Attack: Network Meta-Analysis J Am Heart Assoc. 2015 Aug 24;4(8):e002259. doi: 10.1161/JAHA.115.002259.
1030. Kramer DB, Hatfield LA, Normand SL. Comparative effectiveness of cardiac implantable electrical devices Heart. 2015 Nov;101(22):1773-5. doi: 10.1136/heartjnl-2015-308295. Epub 2015 Aug 24.
1031. Zhang L, Cao HY, Zhao S, Yuan LJ, Han D, Jiang H, Wu S, Wu HM. Effect of exogenous pulmonary surfactants on mortality rate in neonatal respiratory distress syndrome: A network meta-analysis of randomized controlled trials Pulm Pharmacol Ther. 2015 Oct;34:46-54. doi: 10.1016/j.pupt.2015.08.005. Epub 2015 Aug 18.
1032. Brigo F, Nardone R, Tezzon F, Trinka E. A Common Reference-Based Indirect Comparison Meta-Analysis of Buccal versus Intranasal Midazolam for Early Status Epilepticus CNS Drugs. 2015 Sep;29(9):741-57. doi: 10.1007/s40263-015-0271-x.
1033. Khoo AL, Zhou HJ, Teng M, Lin L, Zhao YJ, Soh LB, Mok YM, Lim BP, Gwee KP. Network Meta-Analysis and Cost-Effectiveness Analysis of New Generation Antidepressants CNS Drugs. 2015 Aug;29(8):695-712. doi: 10.1007/s40263-015-0267-6.
1034. Li BZ, Threapleton DE, Wang JY, Xu JM, Yuan JQ, Zhang C, Li P, Ye QL, Guo B, Mao C, Ye DQ. Comparative effectiveness and tolerance of treatments for Helicobacter pylori: systematic review and network meta-analysis BMJ. 2015 Aug 19;351:h4052. doi: 10.1136/bmj.h4052.
1035. Linde K, Rücker G, Sigterman K, Jamil S, Meissner K, Schneider A, Kriston L. Comparative effectiveness of psychological treatments for depressive disorders in primary care: network meta-analysis BMC Fam Pract. 2015 Aug 19;16:103. doi: 10.1186/s12875-015-0314-x.
1036. Chen YP, Guo R, Liu N, Liu X, Mao YP, Tang LL, Zhou GQ, Lin AH, Sun Y, Ma J. Efficacy of the Additional Neoadjuvant Chemotherapy to Concurrent Chemoradiotherapy for Patients with Locoregionally Advanced Nasopharyngeal Carcinoma: a Bayesian Network Meta-analysis of Randomized Controlled Trials J Cancer. 2015 Jul 17;6(9):883-92. doi: 10.7150/jca.11814. eCollection 2015.
1037. Katsanos K, Spiliopoulos S, Saha P, Diamantopoulos A, Karunanithy N, Krokidis M, Modarai B, Karnabatidis D. Comparative Efficacy and Safety of Different Antiplatelet Agents for Prevention of Major Cardiovascular Events and Leg Amputations in Patients with Peripheral Arterial Disease: A Systematic Review and Network Meta-Analysis PLoS One. 2015 Aug 14;10(8):e0135692. doi: 10.1371/journal.pone.0135692. eCollection 2015.
1038. Wolff RF, Ryder S, Bossi A, Briganti A, Crook J, Henry A, Karnes J, Potters L, de Reijke T, Stone N, Burckhardt M, Duffy S, Worthy G, Kleijnen J. A systematic review of randomised controlled trials of radiotherapy for localised prostate cancer Eur J Cancer. 2015 Nov;51(16):2345-67. doi: 10.1016/j.ejca.2015.07.019. Epub 2015 Aug 5.
1039. Zhong X, Zhang T, Liu Y, Wei X, Zhang X, Qin Y, Jin Z, Chen Q, Ma X, Wang R, He J. Effects of three injectable antidiabetic agents on glycaemic control, weight change and drop-out in type 2 diabetes suboptimally controlled with metformin and/or a sulfonylurea: A network meta-analysis Diabetes Res Clin Pract. 2015 Sep;109(3):451-60. doi: 10.1016/j.diabres.2015.05.048. Epub 2015 Jun 5.
1040. Badiani B, Messori A. Targeted Treatments for Pulmonary Arterial Hypertension: Interpreting Outcomes by Network Meta-analysis Heart Lung Circ. 2016 Jan;25(1):46-52. doi: 10.1016/j.hlc.2015.05.020. Epub 2015 Jul 6.
1041. Lee JY, Cho KS, Kang DH, Jung HD, Kwon JK, Oh CK, Ham WS, Choi YD. A network meta-analysis of therapeutic outcomes after new image technology-assisted transurethral resection for non-muscle invasive bladder cancer: 5-aminolaevulinic acid fluorescence vs hexylaminolevulinate fluorescence vs narrow band imaging BMC Cancer. 2015 Aug 1;15:566. doi: 10.1186/s12885-015-1571-8.
1042. Zangrillo A, Musu M, Greco T, Di Prima AL, Matteazzi A, Testa V, Nardelli P, Febres D, Monaco F, Calabrò MG, Ma J, Finco G, Landoni G. Additive Effect on Survival of Anaesthetic Cardiac Protection and Remote Ischemic Preconditioning in Cardiac Surgery: A Bayesian Network Meta-Analysis of Randomized Trials PLoS One. 2015 Jul 31;10(7):e0134264. doi: 10.1371/journal.pone.0134264. eCollection 2015.
1043. Liang C, Yang F, Lin W, Fan Y. Efficacies of surgical treatments based on Harris hip score in elderly patients with femoral neck fracture Int J Clin Exp Med. 2015 May 15;8(5):6784-93. eCollection 2015.
1044. Zhang W, Zou K, Doherty M. Placebos for Knee Osteoarthritis: Reaffirmation of "Needle Is Better Than Pill" Ann Intern Med. 2015 Sep 1;163(5):392-3. doi: 10.7326/M15-1580.
1045. Zhou F, Mao Z, Zeng X, Kang H, Liu H, Pan L, Hou PC. Vasopressors in septic shock: a systematic review and network meta-analysis Ther Clin Risk Manag. 2015 Jul 14;11:1047-59. doi: 10.2147/TCRM.S80060. eCollection 2015.
1046. Song GM, Tian X, Zhang L, Ou YX, Yi LJ, Shuai T, Zhou JG, Zeng Z, Yang HL. Immunonutrition Support for Patients Undergoing Surgery for Gastrointestinal Malignancy: Preoperative, Postoperative, or Perioperative? A Bayesian Network Meta-Analysis of Randomized Controlled Trials Medicine (Baltimore). 2015 Jul;94(29):e1225. doi: 10.1097/MD.0000000000001225.
1047. Larkin J, Paine A, Foley G, Mitchell S, Chen C. First-line treatment in the management of advanced renal cell carcinoma: systematic review and network meta-analysis Expert Opin Pharmacother. 2015;16(13):1915-27. doi: 10.1517/14656566.2015.1058359.
1048. Qi P, Chen M, Zhang LX, Song RX, He ZH, Wang ZP. A Meta-Analysis and Indirect Comparison of Endothelin A Receptor Antagonist for Castration-Resistant Prostate Cancer PLoS One. 2015 Jul 20;10(7):e0133803. doi: 10.1371/journal.pone.0133803. eCollection 2015.
1049. Singh S, Khera R, Allen AM, Murad MH, Loomba R. Comparative effectiveness of pharmacological interventions for nonalcoholic steatohepatitis: A systematic review and network meta-analysis Hepatology. 2015 Nov;62(5):1417-32. doi: 10.1002/hep.27999. Epub 2015 Oct 1.
1050. Fang XZ, Gao J, Ge YL, Zhou LJ, Zhang Y. Network Meta-Analysis on the Efficacy of Dexmedetomidine, Midazolam, Ketamine, Propofol, and Fentanyl for the Prevention of Sevoflurane-Related Emergence Agitation in Children Am J Ther. 2016 Jul-Aug;23(4):e1032-42. doi: 10.1097/MJT.0000000000000321.
1051. Yuan JQ, Mao C, Wong SY, Yang ZY, Fu XH, Dai XY, Tang JL. Comparative Effectiveness and Safety of Monodrug Therapies for Lower Urinary Tract Symptoms Associated With Benign Prostatic Hyperplasia: A Network Meta-analysis Medicine (Baltimore). 2015 Jul;94(27):e974. doi: 10.1097/MD.0000000000000974.
1052. Potts JE, Gray LJ, Brady EM, Khunti K, Davies MJ, Bodicoat DH. The Effect of Glucagon-Like Peptide 1 Receptor Agonists on Weight Loss in Type 2 Diabetes: A Systematic Review and Mixed Treatment Comparison Meta-Analysis PLoS One. 2015 Jun 29;10(6):e0126769. doi: 10.1371/journal.pone.0126769. eCollection 2015.
1053. Zhu GQ, Huang S, Huang GQ, Wang LR, Lin YQ, Wu YM, Shi KQ, Wang JT, Zhou ZR, Braddock M, Chen YP, Zhou MT, Zheng MH. Optimal drug regimens for primary biliary cirrhosis: a systematic review and network meta-analysis Oncotarget. 2015 Sep 15;6(27):24533-49. doi: 10.18632/oncotarget.4528.
1054. Mearns ES, Saulsberry WJ, White CM, Kohn CG, Lemieux S, Sihabout A, Salamucha I, Coleman CI. Efficacy and safety of antihyperglycaemic drug regimens added to metformin and sulphonylurea therapy in Type 2 diabetes: a network meta-analysis Diabet Med. 2015 Dec;32(12):1530-40. doi: 10.1111/dme.12837. Epub 2015 Jul 17.
1055. Singh S, Murad MH, Chandar AK, Bongiorno CM, Singal AK, Atkinson SR, Thursz MR, Loomba R, Shah VH. Comparative Effectiveness of Pharmacological Interventions for Severe Alcoholic Hepatitis: A Systematic Review and Network Meta-analysis Gastroenterology. 2015 Oct;149(4):958-70.e12. doi: 10.1053/j.gastro.2015.06.006. Epub 2015 Jun 16.
1056. Tricco AC, Soobiah C, Blondal E, Veroniki AA, Khan PA, Vafaei A, Ivory J, Strifler L, Ashoor H, MacDonald H, Reynen E, Robson R, Ho J, Ng C, Antony J, Mrklas K, Hutton B, Hemmelgarn BR, Moher D, Straus SE. Comparative efficacy of serotonin (5-HT3) receptor antagonists in patients undergoing surgery: a systematic review and network meta-analysis BMC Med. 2015 Jun 18;13:136. doi: 10.1186/s12916-015-0371-y.
1057. Tian SY, Feldman BM, Beyene J, Brown PE, Uleryk EM, Silverman ED. Immunosuppressive Therapies for the Maintenance Treatment of Proliferative Lupus Nephritis: A Systematic Review and Network Metaanalysis J Rheumatol. 2015 Aug;42(8):1392-400. doi: 10.3899/jrheum.141650. Epub 2015 Jun 15.
1058. Palmerini T, Benedetto U, Biondi-Zoccai G, Della Riva D, Bacchi-Reggiani L, Smits PC, Vlachojannis GJ, Jensen LO, Christiansen EH, Berencsi K, Valgimigli M, Orlandi C, Petrou M, Rapezzi C, Stone GW. Long-Term Safety of Drug-Eluting and Bare-Metal Stents: Evidence From a Comprehensive Network Meta-Analysis J Am Coll Cardiol. 2015 Jun 16;65(23):2496-507. doi: 10.1016/j.jacc.2015.04.017.
1059. Blann AD, Skjøth F, Rasmussen LH, Larsen TB, Lip GY. Edoxaban versus placebo, aspirin, or aspirin plus clopidogrel for stroke prevention in atrial fibrillation. An indirect comparison analysis Thromb Haemost. 2015 Aug;114(2):403-9. doi: 10.1160/TH15-05-0383. Epub 2015 Jun 11.
1060. Zhao Y, Han S, Shang J, Zhao X, Pu R, Shi L. Effectiveness of drug treatment strategies to prevent asthma exacerbations and increase symptom-free days in asthmatic children: a network meta-analysis J Asthma. 2015 Oct;52(8):846-57. doi: 10.3109/02770903.2015.1014101. Epub 2015 Jun 10.
1061. Zhu GQ, Shi KQ, Yu HJ, He SY, Braddock M, Zhou MT, Chen YP, Zheng MH. Optimal adjuvant therapy for resected hepatocellular carcinoma: a systematic review with network meta-analysis Oncotarget. 2015 Jul 20;6(20):18151-61. doi: 10.18632/oncotarget.4098.
1062. Regnier SA, Larsen M, Bezlyak V, Allen F. Comparative efficacy and safety of approved treatments for macular oedema secondary to branch retinal vein occlusion: a network meta-analysis BMJ Open. 2015 Jun 5;5(6):e007527. doi: 10.1136/bmjopen-2014-007527.
1063. Generali D, Venturini S, Rognoni C, Ciani O, Pusztai L, Loi S, Jerusalem G, Bottini A, Tarricone R. A network meta-analysis of everolimus plus exemestane versus chemotherapy in the first- and second-line treatment of estrogen receptor-positive metastatic breast cancer Breast Cancer Res Treat. 2015 Jul;152(1):95-117. doi: 10.1007/s10549-015-3453-9. Epub 2015 Jun 5.
1064. Huang X, Xu B. Efficacy and Safety of Tacrolimus versus Pimecrolimus for the Treatment of Atopic Dermatitis in Children: A Network Meta-Analysis Dermatology. 2015;231(1):41-9. doi: 10.1159/000381948. Epub 2015 May 30.
1065. Zhou X, Hetrick SE, Cuijpers P, Qin B, Barth J, Whittington CJ, Cohen D, Del Giovane C, Liu Y, Michael KD, Zhang Y, Weisz JR, Xie P. Comparative efficacy and acceptability of psychotherapies for depression in children and adolescents: A systematic review and network meta-analysis World Psychiatry. 2015 Jun;14(2):207-22. doi: 10.1002/wps.20217.
1066. Tolley K, Hutchinson M, You X, Wang P, Sperling B, Taneja A, Siddiqui MK, Kinter E. A Network Meta-Analysis of Efficacy and Evaluation of Safety of Subcutaneous Pegylated Interferon Beta-1a versus Other Injectable Therapies for the Treatment of Relapsing-Remitting Multiple Sclerosis PLoS One. 2015 Jun 3;10(6):e0127960. doi: 10.1371/journal.pone.0127960. eCollection 2015.
1067. Thom H, Thompson JC, Scott DA, Halfpenny N, Sulham K, Corey GR. Comparative efficacy of antibiotics for the treatment of acute bacterial skin and skin structure infections (ABSSSI): a systematic review and network meta-analysis Curr Med Res Opin. 2015 Aug;31(8):1539-51. doi: 10.1185/03007995.2015.1058248. Epub 2015 Jun 30.
1068. Mei WQ, Hu HZ, Liu Y, Li ZC, Wang WG. Infliximab is superior to other biological agents for treatment of active ulcerative colitis: A meta-analysis World J Gastroenterol. 2015 May 21;21(19):6044-51. doi: 10.3748/wjg.v21.i19.6044.
1069. Mayor S. No blood pressure lowering agents prolong survival in diabetes and kidney disease, review shows BMJ. 2015 May 25;350:h2824. doi: 10.1136/bmj.h2824.
1070. Lee YH, Bae SC, Song GG. Comparative efficacy and safety of tofacitinib, with or without methotrexate, in patients with active rheumatoid arthritis: a Bayesian network meta-analysis of randomized controlled trials Rheumatol Int. 2015 Dec;35(12):1965-74. doi: 10.1007/s00296-015-3291-4. Epub 2015 May 21.
1071. Yu Q, Zhu Z, Liu Y, Zhang J, Li K. Efficacy and Safety of HER2-Targeted Agents for Breast Cancer with HER2-Overexpression: A Network Meta-Analysis PLoS One. 2015 May 20;10(5):e0127404. doi: 10.1371/journal.pone.0127404. eCollection 2015.
1072. Liu M, Zhang H, Du BX, Xu FY, Zou Z, Sui B, Shi XY. Neurokinin-1 receptor antagonists in preventing postoperative nausea and vomiting: a systematic review and meta-analysis Medicine (Baltimore). 2015 May;94(19):e762. doi: 10.1097/MD.0000000000000762.
1073. Dixon WG. Rheumatoid arthritis: biological drugs and risk of infection Lancet. 2015 Jul 18;386(9990):224-5. doi: 10.1016/S0140-6736(14)61907-3. Epub 2015 May 11.
1074. Vemulapalli S, Dolor RJ, Hasselblad V, Subherwal S, Schmit KM, Heidenfelder BL, Patel MR, Schuyler Jones W. Comparative Effectiveness of Medical Therapy, Supervised Exercise, and Revascularization for Patients With Intermittent Claudication: A Network Meta-analysis Clin Cardiol. 2015 Jun;38(6):378-86. doi: 10.1002/clc.22406. Epub 2015 May 12.
1075. Gu S, Shi J, Tang Z, Sawhney M, Hu H, Shi L, Fonseca V, Dong H. Comparison of glucose lowering effect of metformin and acarbose in type 2 diabetes mellitus: a meta-analysis PLoS One. 2015 May 11;10(5):e0126704. doi: 10.1371/journal.pone.0126704. eCollection 2015.
1076. Zhang L, Pang Y, Shi Y, Xu M, Xu X, Zhang J, Ji L, Zhao D. Indirect comparison of teriparatide, denosumab, and oral bisphosphonates for the prevention of vertebral and nonvertebral fractures in postmenopausal women with osteoporosis Menopause. 2015 Sep;22(9):1021-5. doi: 10.1097/GME.0000000000000466.
1077. van Sloten TT, Stehouwer CD. Effects of RAS inhibitors on diabetic retinopathy - Authors' reply Lancet Diabetes Endocrinol. 2015 May;3(5):316. doi: 10.1016/S2213-8587(15)00084-4.
1078. Buckley F, Finckh A, Huizinga TW, Dejonckheere F, Jansen JP. Comparative Efficacy of Novel DMARDs as Monotherapy and in Combination with Methotrexate in Rheumatoid Arthritis Patients with Inadequate Response to Conventional DMARDs: A Network Meta-Analysis J Manag Care Spec Pharm. 2015 May;21(5):409-23. doi: 10.18553/jmcp.2015.21.5.409.
1079. Xu GY, Qiu Y, Mao HJ. A Network Meta-analysis of Outcomes of 7 Surgical Treatments for Distal Radius Fractures Am J Ther. 2016 Nov/Dec;23(6):e1320-e1328. doi: 10.1097/MJT.0000000000000228.
1080. Wang H, Zhang X, Bai Y, Duan Z, Lin Y, Wang G, Li F. Comparative efficacy and acceptability of five anti-tubercular drugs in treatment of multidrug resistant tuberculosis: a network meta-analysis J Clin Bioinforma. 2015 Apr 28;5:5. doi: 10.1186/s13336-015-0020-x. eCollection 2015.
1081. Bateman ED, Esser D, Chirila C, Fernandez M, Fowler A, Moroni-Zentgraf P, FitzGerald JM. Magnitude of effect of asthma treatments on Asthma Quality of Life Questionnaire and Asthma Control Questionnaire scores: Systematic review and network meta-analysis J Allergy Clin Immunol. 2015 Oct;136(4):914-22. doi: 10.1016/j.jaci.2015.03.023. Epub 2015 May 1.
1082. Taieb V, Pacou M, Ho S, Pettré S, Van Sanden S, Pisini M, Ustianowski A, Mehnert A. A network meta-analysis to compare simeprevir with boceprevir and telaprevir in combination with peginterferon-α and ribavirin in patients infected with genotype 1 Hepatitis C virus J Med Econ. 2015;18(10):787-96. doi: 10.3111/13696998.2015.1046880. Epub 2015 May 26.
1083. Loveman E, Copley VR, Scott DA, Colquitt JL, Clegg AJ, O'Reilly KM. Comparing new treatments for idiopathic pulmonary fibrosis--a network meta-analysis BMC Pulm Med. 2015 Apr 18;15:37. doi: 10.1186/s12890-015-0034-y.
1084. Kocsis JH. Antidepressant augmentation: good news, limitations, and stumbling blocks J Clin Psychiatry. 2015 Apr;76(4):e526-7. doi: 10.4088/JCP.14com09536.
1085. Govan L, Wu O, Xin Y, Hutchinson SJ, Hawkins N. Comparative effectiveness of antiviral treatment for hepatitis B: a systematic review and Bayesian network meta-analysis Eur J Gastroenterol Hepatol. 2015 Aug;27(8):882-94. doi: 10.1097/MEG.0000000000000376.
1086. Mearns ES, Sobieraj DM, White CM, Saulsberry WJ, Kohn CG, Doleh Y, Zaccaro E, Coleman CI. Comparative efficacy and safety of antidiabetic drug regimens added to metformin monotherapy in patients with type 2 diabetes: a network meta-analysis PLoS One. 2015 Apr 28;10(4):e0125879. doi: 10.1371/journal.pone.0125879. eCollection 2015.
1087. Rochwerg B, Alhazzani W, Gibson A, Ribic CM, Sindi A, Heels-Ansdell D, Thabane L, Fox-Robichaud A, Mbuagbaw L, Szczeklik W, Alshamsi F, Altayyar S, Ip W, Li G, Wang M, Włudarczyk A, Zhou Q, Annane D, Cook DJ, Jaeschke R, Guyatt GH; FISSH Group (Fluids in Sepsis and Septic Shock). Fluid type and the use of renal replacement therapy in sepsis: a systematic review and network meta-analysis Intensive Care Med. 2015 Sep;41(9):1561-71. doi: 10.1007/s00134-015-3794-1. Epub 2015 Apr 23.
1088. Dumville JC, McFarlane E, Edwards P, Lipp A, Holmes A, Liu Z. Preoperative skin antiseptics for preventing surgical wound infections after clean surgery Cochrane Database Syst Rev. 2015 Apr 21;2015(4):CD003949. doi: 10.1002/14651858.CD003949.pub4.
1089. Bow EJ, Vanness DJ, Slavin M, Cordonnier C, Cornely OA, Marks DI, Pagliuca A, Solano C, Cragin L, Shaul AJ, Sorensen S, Chambers R, Kantecki M, Weinstein D, Schlamm H. Systematic review and mixed treatment comparison meta-analysis of randomized clinical trials of primary oral antifungal prophylaxis in allogeneic hematopoietic cell transplant recipients BMC Infect Dis. 2015 Mar 17;15:128. doi: 10.1186/s12879-015-0855-6.
1090. Maxwell LJ, Zochling J, Boonen A, Singh JA, Veras MM, Tanjong Ghogomu E, Benkhalti Jandu M, Tugwell P, Wells GA. TNF-alpha inhibitors for ankylosing spondylitis Cochrane Database Syst Rev. 2015 Apr 18;(4):CD005468. doi: 10.1002/14651858.CD005468.pub2.
1091. Wang C, Guo L, Chi C, Wang X, Guo L, Wang W, Zhao N, Wang Y, Zhang Z, Li E. Mechanical ventilation modes for respiratory distress syndrome in infants: a systematic review and network meta-analysis Crit Care. 2015 Mar 20;19(1):108. doi: 10.1186/s13054-015-0843-7.
1092. van Walsem A, Pandhi S, Nixon RM, Guyot P, Karabis A, Moore RA. Relative benefit-risk comparing diclofenac to other traditional non-steroidal anti-inflammatory drugs and cyclooxygenase-2 inhibitors in patients with osteoarthritis or rheumatoid arthritis: a network meta-analysis Arthritis Res Ther. 2015 Mar 19;17(1):66. doi: 10.1186/s13075-015-0554-0.
1093. Xiao J, Wu WX, Ye YY, Lin WJ, Wang L. A Network Meta-analysis of Randomized Controlled Trials Focusing on Different Allergic Rhinitis Medications Am J Ther. 2016 Nov/Dec;23(6):e1568-e1578. doi: 10.1097/MJT.0000000000000242.
1094. Moja L, Danese S, Fiorino G, Del Giovane C, Bonovas S. Systematic review with network meta-analysis: comparative efficacy and safety of budesonide and mesalazine (mesalamine) for Crohn's disease Aliment Pharmacol Ther. 2015 Jun;41(11):1055-65. doi: 10.1111/apt.13190. Epub 2015 Apr 13.
1095. Stynes G, Svedsater H, Wex J, Lettis S, Leather D, Castelnuovo E, Detry M, Berry S. Once-daily fluticasone furoate/vilanterol 100/25 mcg versus twice daily combination therapies in COPD - mixed treatment comparisons of clinical efficacy Respir Res. 2015 Feb 15;16(1):25. doi: 10.1186/s12931-015-0184-8.
1096. Hüttner FJ, Tenckhoff S, Jensen K, Uhlmann L, Kulu Y, Büchler MW, Diener MK, Ulrich A. Meta-analysis of reconstruction techniques after low anterior resection for rectal cancer Br J Surg. 2015 Jun;102(7):735-45. doi: 10.1002/bjs.9782. Epub 2015 Mar 31.
1097. Galván-Banqueri M, Vega-Coca MD, Castillo-Muñoz MA, Beltrán Calvo C, Molina López T. Indirect comparison for Anti-TNF drugs in moderate to severe ulcerative colitis Farm Hosp. 2015 Mar 1;39(2):80-91. doi: 10.7399/fh.2015.39.2.8218.
1098. Sobieraj DM, Coleman CI, Pasupuleti V, Deshpande A, Kaw R, Hernandez AV. Comparative efficacy and safety of anticoagulants and aspirin for extended treatment of venous thromboembolism: A network meta-analysis Thromb Res. 2015 May;135(5):888-96. doi: 10.1016/j.thromres.2015.02.032. Epub 2015 Mar 4.
1099. Kunitomi T, Hashiguchi M, Mochizuki M. Effect of common comparators in indirect comparison analysis of the effectiveness of different inhaled corticosteroids in the treatment of asthma PLoS One. 2015 Mar 20;10(3):e0120836. doi: 10.1371/journal.pone.0120836. eCollection 2015.
1100. Zhu GQ, Shi KQ, Huang S, Huang GQ, Lin YQ, Zhou ZR, Braddock M, Chen YP, Zheng MH. Network meta-analysis of randomized controlled trials: efficacy and safety of UDCA-based therapies in primary biliary cirrhosis Medicine (Baltimore). 2015 Mar;94(11):e609. doi: 10.1097/MD.0000000000000609.
1101. Wang C, Zhao N, Wang W, Guo L, Guo L, Chi C, Wang X, Pi X, Cui Y, Li E. Intraoperative mechanical ventilation strategies for obese patients: a systematic review and network meta-analysis Obes Rev. 2015 Jun;16(6):508-17. doi: 10.1111/obr.12274. Epub 2015 Mar 18.
1102. Thorlund K, Druyts E, Toor K, Mills EJ. Comparative efficacy of golimumab, infliximab, and adalimumab for moderately to severely active ulcerative colitis: a network meta-analysis accounting for differences in trial designs Expert Rev Gastroenterol Hepatol. 2015 May;9(5):693-700. doi: 10.1586/17474124.2015.1024657. Epub 2015 Mar 12.
1103. Dong W, Goost H, Lin XB, Burger C, Paul C, Wang ZL, Zhang TY, Jiang ZC, Welle K, Kabir K. Treatments for shoulder impingement syndrome: a PRISMA systematic review and network meta-analysis Medicine (Baltimore). 2015 Mar;94(10):e510. doi: 10.1097/MD.0000000000000510.
1104. Wang Z, Qiao D, Lu Y, Curtis D, Wen X, Yao Y, Zhao H. Systematic literature review and network meta-analysis comparing bone-targeted agents for the prevention of skeletal-related events in cancer patients with bone metastasis Oncologist. 2015 Apr;20(4):440-9. doi: 10.1634/theoncologist.2014-0328. Epub 2015 Mar 2.
1105. Kotb A, Cameron C, Hsieh S, Wells G. Comparative effectiveness of different forms of telemedicine for individuals with heart failure (HF): a systematic review and network meta-analysis PLoS One. 2015 Feb 25;10(2):e0118681. doi: 10.1371/journal.pone.0118681. eCollection 2015.
1106. Sun F, Chai S, Li L, Yu K, Yang Z, Wu S, Zhang Y, Ji L, Zhan S. Effects of glucagon-like peptide-1 receptor agonists on weight loss in patients with type 2 diabetes: a systematic review and network meta-analysis J Diabetes Res. 2015;2015:157201. doi: 10.1155/2015/157201. Epub 2015 Jan 20.
1107. Zhu GQ, Shi KQ, Huang S, Wang LR, Lin YQ, Huang GQ, Chen YP, Braddock M, Zheng MH. Systematic review with network meta-analysis: the comparative effectiveness and safety of interventions in patients with overt hepatic encephalopathy Aliment Pharmacol Ther. 2015 Apr;41(7):624-35. doi: 10.1111/apt.13122. Epub 2015 Feb 13.
1108. Messori A, Fadda V, Maratea D, Trippoli S. First-line treatments for chronic lymphocytic leukaemia: interpreting efficacy data by network meta-analysis Ann Hematol. 2015 Jun;94(6):1003-9. doi: 10.1007/s00277-015-2310-6. Epub 2015 Feb 14.
1109. Pilkington G, Boland A, Brown T, Oyee J, Bagust A, Dickson R. A systematic review of the clinical effectiveness of first-line chemotherapy for adult patients with locally advanced or metastatic non-small cell lung cancer Thorax. 2015 Apr;70(4):359-67. doi: 10.1136/thoraxjnl-2014-205914. Epub 2015 Feb 6.
1110. Alfirevic Z, Keeney E, Dowswell T, Welton NJ, Dias S, Jones LV, Navaratnam K, Caldwell DM. Labour induction with prostaglandins: a systematic review and network meta-analysis BMJ. 2015 Feb 5;350:h217. doi: 10.1136/bmj.h217.
1111. Greco T, Calabrò MG, Covello RD, Greco M, Pasin L, Morelli A, Landoni G, Zangrillo A. A Bayesian network meta-analysis on the effect of inodilatory agents on mortality Br J Anaesth. 2015 May;114(5):746-56. doi: 10.1093/bja/aeu446. Epub 2015 Feb 4.
1112. Stevens JW, Khunti K, Harvey R, Johnson M, Preston L, Woods HB, Davies M, Goyder E. Preventing the progression to type 2 diabetes mellitus in adults at high risk: a systematic review and network meta-analysis of lifestyle, pharmacological and surgical interventions Diabetes Res Clin Pract. 2015 Mar;107(3):320-31. doi: 10.1016/j.diabres.2015.01.027. Epub 2015 Jan 21.
1113. Edwards SJ, Barton S, Thurgar E, Trevor N. Topotecan, pegylated liposomal doxorubicin hydrochloride, paclitaxel, trabectedin and gemcitabine for advanced recurrent or refractory ovarian cancer: a systematic review and economic evaluation Health Technol Assess. 2015 Jan;19(7):1-480. doi: 10.3310/hta19070.
1114. Nelson H, Cartier S, Allen-Ramey F, Lawton S, Calderon MA. Network meta-analysis shows commercialized subcutaneous and sublingual grass products have comparable efficacy J Allergy Clin Immunol Pract. 2015 Mar-Apr;3(2):256-266.e3. doi: 10.1016/j.jaip.2014.09.018. Epub 2014 Nov 20.
1115. Owen RK, Tincello DG, Keith RA. Network meta-analysis: development of a three-level hierarchical modeling approach incorporating dose-related constraints Value Health. 2015 Jan;18(1):116-26. doi: 10.1016/j.jval.2014.10.006.
1116. Oba Y, Lone NA. Comparative efficacy of long-acting muscarinic antagonists in preventing COPD exacerbations: a network meta-analysis and meta-regression Ther Adv Respir Dis. 2015 Feb;9(1):3-15. doi: 10.1177/1753465814565624.
1117. deGruy FV. Treatment of depression in primary care Ann Fam Med. 2015 Jan-Feb;13(1):3-5. doi: 10.1370/afm.1726.
1118. Bannuru RR, Schmid CH, Kent DM, Vaysbrot EE, Wong JB, McAlindon TE. Comparative effectiveness of pharmacologic interventions for knee osteoarthritis: a systematic review and network meta-analysis Ann Intern Med. 2015 Jan 6;162(1):46-54. doi: 10.7326/M14-1231.
1119. Bulluck H, Kwok CS, Ryding AD, Loke YK. Safety of short-term dual antiplatelet therapy after drug-eluting stents: An updated meta-analysis with direct and adjusted indirect comparison of randomized control trials Int J Cardiol. 2015 Feb 15;181:331-9. doi: 10.1016/j.ijcard.2014.12.037. Epub 2014 Dec 13.
1120. Sun F, Wu S, Wang J, Guo S, Chai S, Yang Z, Li L, Zhang Y, Ji L, Zhan S. Effect of glucagon-like peptide-1 receptor agonists on lipid profiles among type 2 diabetes: a systematic review and network meta-analysis Clin Ther. 2015 Jan 1;37(1):225-241.e8. doi: 10.1016/j.clinthera.2014.11.008. Epub 2014 Dec 29.
1121. Llorca PM, Lançon C, Brignone M, Painchault C, Rive B, Toumi M, François C. Can we well assess the relative efficacy and tolerability of a new drug versus others at the time of marketing authorization using mixed treatment comparisons? A detailed illustration with escitalopram J Mark Access Health Policy. 2015 Sep 24;3. doi: 10.3402/jmahp.v3.26776. eCollection 2015.
1122. Haspinger ER, Agustoni F, Torri V, Gelsomino F, Platania M, Zilembo N, Gallucci R, Garassino MC, Cinquini M. Is there evidence for different effects among EGFR-TKIs? Systematic review and meta-analysis of EGFR tyrosine kinase inhibitors (TKIs) versus chemotherapy as first-line treatment for patients harboring EGFR mutations Crit Rev Oncol Hematol. 2015 May;94(2):213-27. doi: 10.1016/j.critrevonc.2014.11.005. Epub 2014 Nov 22.
1123. Zeng C, Li H, Yang T, Deng ZH, Yang Y, Zhang Y, Lei GH. Electrical stimulation for pain relief in knee osteoarthritis: systematic review and network meta-analysis Osteoarthritis Cartilage. 2015 Feb;23(2):189-202. doi: 10.1016/j.joca.2014.11.014. Epub 2014 Nov 26.
1124. Popat S, Mellemgaard A, Fahrbach K, Martin A, Rizzo M, Kaiser R, Griebsch I, Reck M. Nintedanib plus docetaxel as second-line therapy in patients with non-small-cell lung cancer: a network meta-analysis Future Oncol. 2015;11(3):409-20. doi: 10.2217/fon.14.290. Epub 2014 Dec 5.
1125. Phan K, Xie A, Kumar N, Wong S, Medi C, La Meir M, Yan TD. Comparing energy sources for surgical ablation of atrial fibrillation: a Bayesian network meta-analysis of randomized, controlled trials Eur J Cardiothorac Surg. 2015 Aug;48(2):201-11. doi: 10.1093/ejcts/ezu408. Epub 2014 Nov 12.
1126. Lee SW, Chaiyakunapruk N, Chong HY, Liong ML. Comparative effectiveness and safety of various treatment procedures for lower pole renal calculi: a systematic review and network meta-analysis BJU Int. 2015 Aug;116(2):252-64. doi: 10.1111/bju.12983. Epub 2015 Mar 17.
1127. Sun F, Chai S, Yu K, Quan X, Yang Z, Wu S, Zhang Y, Ji L, Wang J, Shi L. Gastrointestinal adverse events of glucagon-like peptide-1 receptor agonists in patients with type 2 diabetes: a systematic review and network meta-analysis Diabetes Technol Ther. 2015 Jan;17(1):35-42. doi: 10.1089/dia.2014.0188.
1128. Messori A, Fadda V, Maratea D, Trippoli S, Marinai C. Testing the therapeutic equivalence of novel oral anticoagulants for thromboprophylaxis in orthopedic surgery and for prevention of stroke in atrial fibrillation Int J Clin Pharmacol Ther. 2015 Mar;53(3):211-9. doi: 10.5414/CP202183.
1129. Signorovitch JE, Betts KA, Yan YS, LeReun C, Sundaram M, Wu EQ, Mulani P. Comparative efficacy of biological treatments for moderate-to-severe psoriasis: a network meta-analysis adjusting for cross-trial differences in reference arm response Br J Dermatol. 2015 Feb;172(2):504-12. doi: 10.1111/bjd.13437. Epub 2015 Jan 21.
1130. Sun F, Wu S, Guo S, Yu K, Yang Z, Li L, Zhang Y, Ji L, Zhan S. Effect of GLP-1 receptor agonists on waist circumference among type 2 diabetes patients: a systematic review and network meta-analysis Endocrine. 2015 Apr;48(3):794-803. doi: 10.1007/s12020-014-0373-0. Epub 2014 Aug 13.
1131. Yildiz A, Nikodem M, Vieta E, Correll CU, Baldessarini RJ. A network meta-analysis on comparative efficacy and all-cause discontinuation of antimanic treatments in acute bipolar mania Psychol Med. 2015 Jan;45(2):299-317. doi: 10.1017/S0033291714001305. Epub 2014 Jul 18.
1132. Gupta AK, Daigle D, Paquet M. Therapies for onychomycosis a systematic review and network meta-analysis of mycological cure J Am Podiatr Med Assoc. 2015 Jul;105(4):357-66. doi: 10.7547/13-110.1. Epub 2014 Jul 17.
1133. Mantha S, Ansell J. Indirect comparison of dabigatran, rivaroxaban, apixaban and edoxaban for the treatment of acute venous thromboembolism J Thromb Thrombolysis. 2015 Feb;39(2):155-65. doi: 10.1007/s11239-014-1102-5.
1134. Haaland B, Tan PS, de Castro G Jr, Lopes G. Meta-analysis of first-line therapies in advanced non-small-cell lung cancer harboring EGFR-activating mutations J Thorac Oncol. 2014 Jun;9(6):805-11. doi: 10.1097/JTO.0000000000000156.
1135. Benedetto U, Raja SG, Albanese A, Amrani M, Biondi-Zoccai G, Frati G. Searching for the second best graft for coronary artery bypass surgery: a network meta-analysis of randomized controlled trials† Eur J Cardiothorac Surg. 2015 Jan;47(1):59-65; discussion 65. doi: 10.1093/ejcts/ezu111. Epub 2014 Mar 30.
1136. Reinecke H, Weber C, Lange K, Simon M, Stein C, Sorgatz H. Analgesic efficacy of opioids in chronic pain: recent meta-analyses Br J Pharmacol. 2015 Jan;172(2):324-33. doi: 10.1111/bph.12634. Epub 2014 Jul 1.
1137. Silva, M. A., Donovan, J. L., Durie, R., Ventura, D., Alspach, R. M., Kanaan, A. O., & Njoroge, M. (July 01, 2017). A Mixed Treatment Comparison Meta-Analysis of Pharmacotherapeutic Monotherapy and Placebo for Pulmonary Artery Hypertension. Clinical Pulmonary Medicine, 24, 4, 149-159.
1138. Zheng, M.-H., Xu, J.-G., Zhang, Y.-H., Yang, G., Huo, L.-M., Zheng, M.-H., Tian, J.-H., ... Sun, H.-T. (January 01, 2017). A network meta-analysis of treatment for newly diagnosed glioblastoma based on radiotherapy plus temozolomide. Neurology Asia, 22, 1, 49-58.
1139. Zhang, D., Chen, Y., Liu, H., & Zhou, D. (December 20, 2016). Anti-TNFα agents and interleukin-17A inhibitor secukinmuab have similar effects in improvement of ASAS20, ASAS40, and safety: A meta-analysis. International Journal of Clinical and Experimental Medicine, 9, 11, 20668-20673.
1140. Lipinski, M. J., Lee, R. C., Gaglia, M. A., Torguson, R., Garcia-Garcia, H. M., Pichard, A. D., Satler, L. F., ... Waksman, R. (December 01, 2016). Comparison of heparin, bivalirudin, and different glycoprotein IIb/IIIa inhibitor regimens for anticoagulation during percutaneous coronary intervention: A network meta-analysis. Cardiovascular Revascularization Medicine, 17, 8, 535-545.
1141. Wei, X., Zhu, L., Wang, H., Wang, C., Deng, Q., & Li, X. (April 01, 2017). Efficacy of Traditional Chinese Medicines in Preventing Oxaliplatin-induced Peripheral Neurotoxicity in Cancer Patients: A Network Meta-analysis. Chinese Herbal Medicines, 9, 2, 161-168.
1142. Doosti-Irani, A., Mansournia, M. A., Rahimi-Foroushani, A., Haddad, P., & Holakouie-Naieni, K. 2018. Local recurrence of esophageal squamous cell carcinoma after treatment: Comparison of frequentist and Bayesian network meta-analysis. Clinical Epidemiology and Global Health, 7, 2, 145-152.
1143. Cheng, Z., Hou, S., Sun, Y., Wu, Y., Liu, B., Yuan, M., & Wang, X. (June 30, 2017). Toxicity comparisons of eight chemotherapy regimens in the treatment of metastatic/advanced non-small-cell lung cancer: A network meta-analysis. International Journal of Clinical and Experimental Medicine, 10, 6, 8709-8719.
1144. Ribassin-Majed, L., Marguet, S., Lee, A. W. M., Ng, W. T., Ma, J., Chan, A. T. C., Huang, P. Y., ... Blanchard, P. (January 01, 2017). What Is the Best Treatment of Locally Advanced Nasopharyngeal Carcinoma? An Individual Patient Data Network Meta-Analysis. Journal of Clinical Oncology : Official Journal of the American Society of Clinical Oncology, 35, 5, 498-505.
